# LINC00174 is a novel prognostic factor in thymic epithelial tumors involved in cell migration and lipid metabolism

**DOI:** 10.1038/s41419-020-03171-9

**Published:** 2020-11-07

**Authors:** Claudia Tito, Federica Ganci, Andrea Sacconi, Silvia Masciarelli, Giulia Fontemaggi, Claudio Pulito, Enzo Gallo, Valentina Laquintana, Alessia Iaiza, Luciana De Angelis, Anna Benedetti, Jessica Cacciotti, Selenia Miglietta, Maria Bellenghi, Alessandra Carè, Alessandro Fatica, Daniele Diso, Marco Anile, Vincenzo Petrozza, Francesco Facciolo, Gabriele Alessandrini, Edoardo Pescarmona, Federico Venuta, Mirella Marino, Giovanni Blandino, Francesco Fazi

**Affiliations:** 1 Department of Anatomical, Histological, Forensic & Orthopedic Sciences, Section of Histology & Medical Embryology, Sapienza University of Rome, Laboratory Affiliated to Istituto Pasteur Italia-Fondazione Cenci Bolognetti, Rome, Italy; 2 Oncogenomic and Epigenetic Unit, IRCCS Regina Elena National Cancer Institute, Rome, Italy; 3 Istituto di Istologia ed Embriologia, Università Cattolica del Sacro Cuore, Rome, Italy; 4 Fondazione Policlinico Universitario “A. Gemelli”, IRCCS, Rome, Italy; 5 Molecular Chemoprevention Unit, “Regina Elena” National Cancer Institute - IFO, Rome, Italy; 6 Department of Pathology, IRCCS Regina Elena National Cancer Institute, Rome, Italy; 7 Pathology Unit, ICOT, Department of Medico-Surgical Sciences and Biotechnologies, Sapienza University of Rome, Latina, Italy; 8 Department of Anatomical, Histological, Forensic & Orthopedic Sciences, Section of Human Anatomy, Sapienza University of Rome, Rome, Italy; 9 Center for Gender-Specific Medicine, Oncology Unit-Istituto Superiore di Sanita’, Rome, Italy; 10 Department of Biology and Biotechnology ‘Charles Darwin’, Sapienza University of Rome, Rome, Italy; 11 Department of Thoracic Surgery, Sapienza University of Rome, Rome, Italy; 12 Thoracic Surgery, IRCCS Regina Elena National Cancer Institute, Rome, Italy

**Keywords:** Cancer therapy, Cell migration, Long non-coding RNAs

## Abstract

Long non-coding RNAs are emerging as new molecular players involved in many biological processes, such as proliferation, apoptosis, cell cycle, migration, and differentiation. Their aberrant expression has been reported in variety of diseases. The aim of this study is the identification and functional characterization of clinically relevant lncRNAs responsible for the inhibition of miR-145-5p, a key tumor suppressor in thymic epithelial tumors (TETs). Starting from gene expression analysis by microarray in a cohort of fresh frozen thymic tumors and normal tissues, we identified LINC00174 as upregulated in TET. Interestingly, LINC00174 expression is positively correlated with a 5-genes signature in TETs. Survival analyses, performed on the TCGA dataset, showed that LINC00174 and its associated 5-genes signature are prognostic in TETs. Specifically, we show that LINC00174 favors the expression of SYBU, FEM1B, and SCD5 genes by sponging miR-145-5p, a well-known tumor suppressor microRNA downregulated in a variety of tumors, included TETs. Functionally, LINC00174 impacts on cell migration and lipid metabolism. Specifically, SCD5, one of the LINC00174-associated genes, is implicated in the control of lipid metabolism and promotes thymic cancer cells migration. Our study highlights that LINC00174 and its associated gene signature are relevant prognostic indicators in TETs. Of note, we here show that a key controller of lipid metabolism, SCD5, augments the migration ability of TET cells, creating a link between lipids and motility, and highlighting these pathways as relevant targets for the development of novel therapeutic approaches for TET.

## Introduction

Long non coding RNAs (lncRNAs) represent a class of non-coding RNAs, usually longer than 200 nucleotides, which play functional role through different mechanisms of action, according to their localization^1^. Nuclear lncRNAs are involved in chromatin remodeling, transcription regulation and nuclear architecture^2–4^. Instead, cytoplasmic lncRNAs participate to post transcriptional regulation, mRNA turnover, protein stability and modulation of signaling pathways^5^. One of the well-known roles of lncRNAs is to act as sponges for microRNAs: lncRNAs sequester microRNAs, preventing them from binding target mRNAs, and promoting their degradation or repression of translation^6,7^. Through various mechanisms of action, lncRNAs participate to the regulation of a wide range of biological processes, such as cellular proliferation and differentiation, survival and apoptosis, invasion, migration, and metastasis formation^8^. All these phenotypes, together, contribute to cancer development and progression^9–11^. Indeed, deregulation of a large number of lncRNAs has been associated to different types of cancer tissues^12^, including breast cancer^13^, colorectal cancer^14^, urologic cancer^15^, hepatocellular carcinoma^16^, leukemia^17,18^, melanoma^19^, and possibly others.

Epithelial tumors of the thymus arise in the anterior mediastinum and include thymomas and thymic carcinomas (TCs)^20^. Based on the histological classification of the World Health Organization (WHO), thymomas, which usually present organotypic (thymus-like) features, have been classified into types A, AB, B1, B2, and B3 and rare other subtypes^21,22^. The malignant potential of thymomas may range from low to moderate, depending on tumor stage^23^. Thymomas in advanced stage, mostly B2 and B3 types, as thymic carcinomas (TC), are characterized by local relapse and/or pleural dissemination and lung metastases^24^. TCs resemble similarly termed carcinomas outside the mediastinum^25,26^.

In the present study, we aimed at identifying lncRNAs involved in the inhibition of the activity of miR-145-5p, a key tumor suppressor in TET. We observed an upregulation of LINC00174 in thymic tumor tissue compared to normal tissue, and high levels of LINC00174 associated to poor patients’ prognosis. The alteration of LINC00174 expression had been firstly reported in colorectal carcinoma^27^. Here, we studied the interaction between LINC00174, LINC00174-associated signature and miR-145-5p, one of the most significantly downregulated microRNAs in TET cells. Our results indicate a role for LINC00174 as sponge for miR-145-5p. Furthermore, we show the involvement of LINC00174 and SCD5, one of the five genes belonging to LINC00174-associated signature, in the regulation of migration and lipid metabolism in TC1889 thymic carcinoma cells. Our findings confirm a strong association between lipid synthesis and cell migration in TET, as previously reported in other malignancies^28–31^. Consequently, our results highlight new potential therapeutic targets and prognostic biomarkers clinically relevant for thymic epithelial tumors.

## Results

### Identification of a lncRNA with prognostic value in TETs

**Fig. 1:**
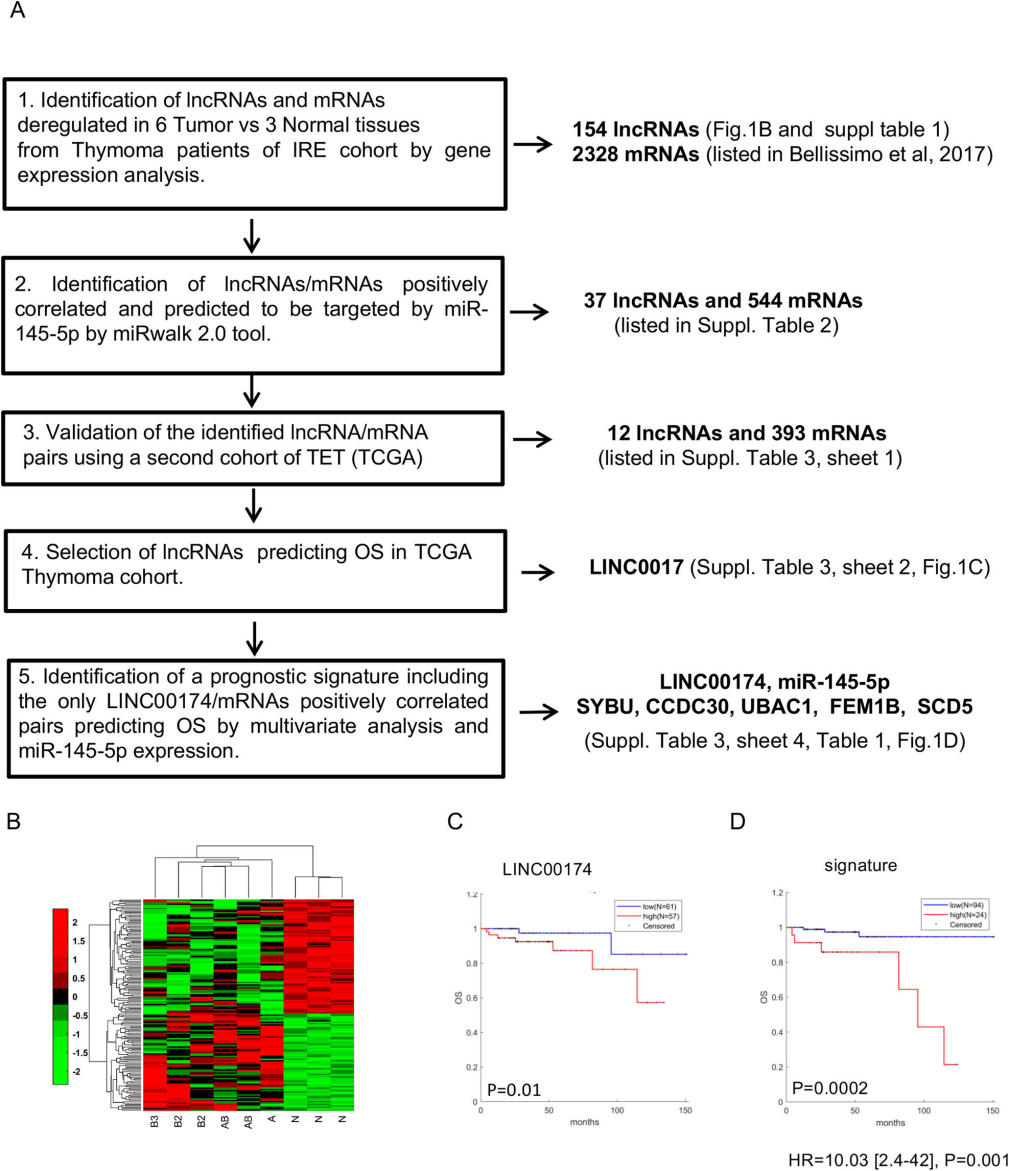
Identification of lncRNAs with prognostic value in Thymoma. **A** Workflow to identify lncRNAs and their associated signature with prognostic value in Thymoma patients. **B** Unsupervised clustering analysis representing the lncRNAs differentially expressed in T vs. N in Thymoma IRE (Istituto Regina Elena) cohort. *N* = normal samples; B2, B3, AB, A = TET histotype. **C**, **D** KM analysis representing the correlation between LINC00174 (**C**), the identified signature (**D**) expression and Overall Survival in TCGA Thymoma cohort. At the bottom of KM are indicated the HR values of multivariate analyses adjusted for histotype of Thymoma.

Gene expression analysis by microarray of a collection of fresh frozen tissues, including 6 TET specimens and 3 normal counterparts (peritumoral thymic tissue), highlighted 154 lncRNAs deregulated between tumor and normal samples (Fig. 1A and Supplementary Table 1). Unsupervised hierarchical clustering of these lncRNA in tumor and normal samples is shown in Fig. 1B. Integrated analysis of the expression of these lncRNAs with that of the mRNAs altered in the same samples, reported in our previous study^32^, allowed identifying pairs of correlated lncRNA/mRNA (Fig. 1A).

As miR-145-5p is a key tumor suppressor in TET^33^, where it is silenced by epigenetic regulation^32^, we here specifically focused on positively correlated lncRNA/mRNA pairs upregulated in TET and presenting a binding site for miR-145-5p (Supplementary Table 2). We selected only those lncRNA/mRNA pairs whose significant correlation was confirmed on a second cohort of TET (e.g., the TCGA cohort), finally obtaining 12 lncRNAs and 393 mRNAs positively correlated presenting a binding site for miR-145-5p (Supplementary Table 3, sheet 1). By using the TCGA dataset^34^, we investigated the relationship between expression of the identified lncRNAs and clinical outcome in TET. We observed that only lncRNA LINC00174 showed a significant prognostic power (Supplementary Table 3, sheet 2). High expression of LINC00174 was indeed associated with decreased overall survival (Fig. 1C). We next focused on the mRNAs positively correlated to LINC00174 (Supplementary Table 3, sheet 3) and we evidenced five pairs of LINC00174/mRNA, comprising UBAC1, FEM1B, CCDC30, SYBU, and SCD5 genes, whose high expression in TET predicted poor overall survival in uni- and multi-variable analysis, adjusting for TET histotypes (Supplementary Table 3, sheet 4 and Table 1). The prognosis of patients with high expression of both LINC00174 and LINC00174-related 5-genes signature was significantly poorer compared to patients with opposite condition (Fig. 1D and Table 1). Moreover, high expression of LINC00174/mRNA pairs maintained the prognostic power also when combined with low expression of miR-145-p (Supplementary Fig. 1 and Table 1).

**Table 1:** Multivariate analysis adjusted for TET histotypes showing the prognostic value of the mRNAs positively correlated to linc00174 in combination or not with LINC00174 and miR-145-5p expression.

				mRNAs-high\ LINC00174-high		mRNAs-high\ LINC00174-high\miR-145-5p-low	
mRNAs	HR [CI]	*p*	logrank	HR [CI]	*p*	HR [CI]	*p*
UBAC1	2.16 [1.12-4.14]	0.01	0.005	2.81 [1.29-6.1]	0.008	9.21 [2.20-38.49]	0.002
SCD5	1.57 [0.85-2.89]	0.14	0.18	2.04 [1.03-4.04]	0.03	8.91 [2.11-37.54]	0.002
CCDC30	2.47 [1.16-5.27]	0.01	0.20	3.21 [1.17-8.78]	0.02	7.10 [1.81-27.78//]	0.004
FEM1B	0.90 [0.46-1.77]	0.77	0.84	2.06 [1.07-3.96]	0.02	7.08 [1.84-27.21]	0.004
SYBU	1.98 [0.84-4.63]	0.11	0.05	7.17 [1.82-28.20]	0.005	13.46 [2.83-63.95]	0.001

### LINC00174 sponges miR-145-5p enabling expression of UBAC1, SYBU, FEM1B, and SCD5

To validate gene expression results, we first examined the levels of LINC00174 and LINC00174-related 5-genes signature (UBAC1, FEM1B, CCDC30, SYBU, and SCD5) in the fresh frozen thymoma and normal tissue samples used for microarrays, and in primary cell culture from normal (peritumoral thymic tissue) and neoplastic thymic tissues (Supplementary Fig. 2A, B). We confirmed that LINC00174 and LINC00174-related signature are upregulated in thymic tumor samples and primary cells compared to normal counterparts. CCDC30 presented very low expression level and couldn’t be reliably evaluated.

**Fig. 2:**
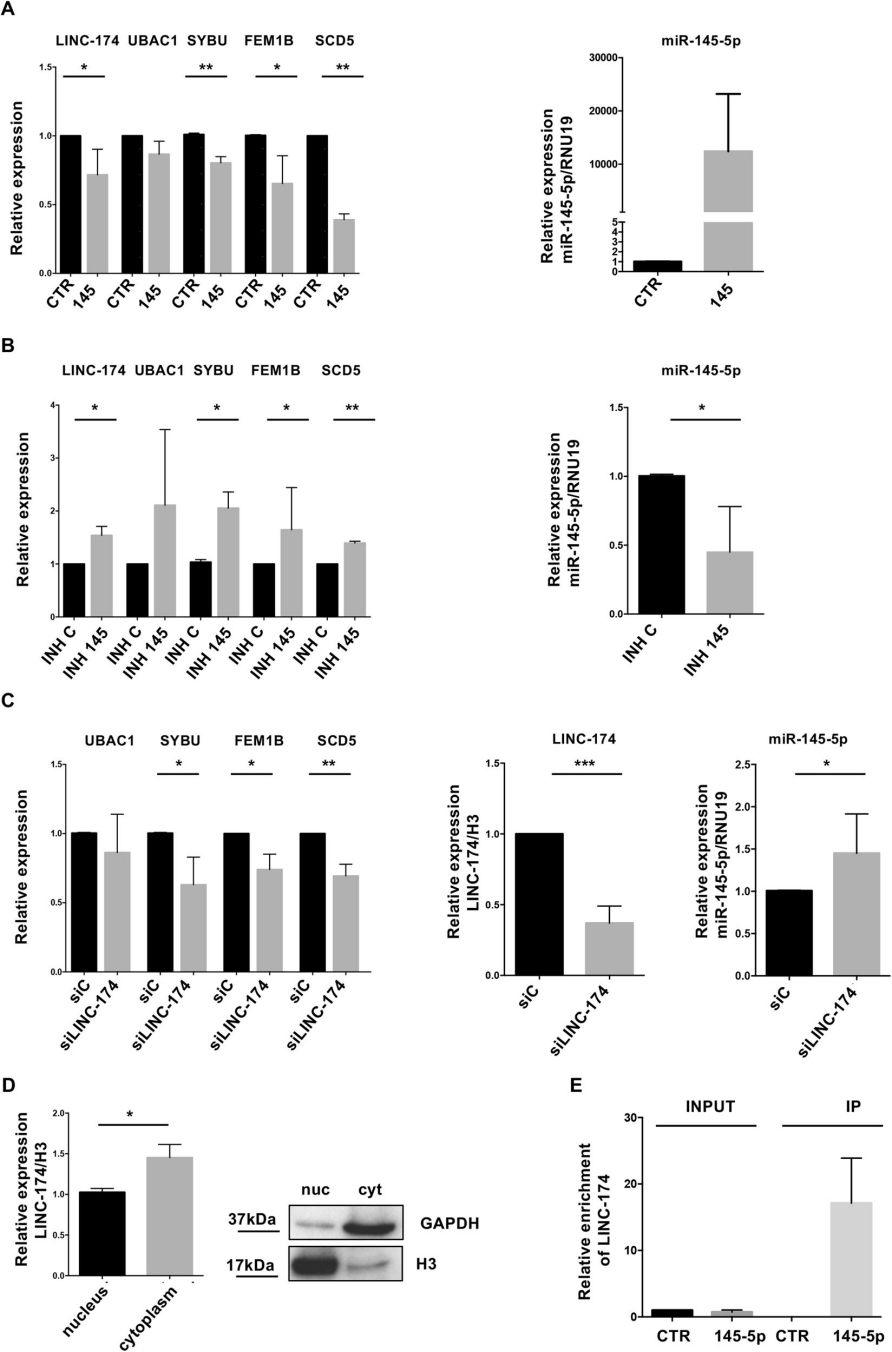
Analysis of the expression of LINC00174 and of its positively correlated mRNA signature. Quantitative reverse transcription-PCR (qRT-PCR) of LINC00174 and of its correlated genes upon overexpression (**A**) (CTR = control mimic oligonucleotide; 145 = miR-145-5p mimic oligonucleotide) or inhibition (**B**) (INH C = control oligonucleotide; INH 145 = miR-145-5p inhibitor oligonucleotide) of miR-145-5p (left panels). miR-145-5p level following its modulation (*n* = 3) is shown in right panels. **C** Expression of LINC00174-correlated genes after silencing of LINC00174 (si-LINC-174) (*n* = 4) analyzed by qRT-PCR. **D** qRT-PCR to evaluate expression of LINC00174 in nuclear and cytoplasmic fractions from TC1889 cells (graph, *n* = 3, *P* = 0.012). Analysis of H3 and GAPDH proteins by WB (right) was used as control of fractionation efficiency. **E** qRT-PCR of LINC00174 in a representative RNA pull-down assay in cells transfected with biotinylated miR-145-5p (145-5p) or control oligonucleotide (CTR). Significant *p*-values are indicated as *P < 0.05; **P < 0.005; ***P < 0.0005.

Based on our hypothesis, LINC00174 would act as a competing endogenous RNA (ceRNA) by sequestering miR-145-5p and releasing the expression of UBAC1, SYBU, FEM1B, and SCD5 mRNAs. To address this, we first overexpressed (Fig. 2A) or inhibited (Fig. 2B) miR-145-5p in thymic carcinoma TC1889 cells and observed, respectively, the downregulation (Fig. 2A) or the upregulation (Fig. 2B) of LINC00174 and LINC00174-related signature, confirming that they depend on miR-145-5p activity.

Next, we inhibited the expression of LINC00174 by using GapmeR oligonucleotides in TC1889 cells. As shown in Fig. 2C, LINC00174 depletion (siLINC-174) leads to the downregulation of SYBU, FEM1B, and SCD5 expression and releases miR-145-5p expression, supporting our hypothesis of LINC00174 acting as a ceRNA. The use of a second GapmeR oligonucleotide, si-LINC-174.3, confirmed these results (Supplementary Fig. 3). According to the ceRNA function of LINC00174, we observed that its levels are higher in the cytoplasm than in nucleus of TC1889 cells (Fig. 2D).

To further support a role for LINC00174 in sequestering miR-145-5p, we evaluated the interaction between LINC00174 and miR-145-5p. Importantly, overexpression of biotinylated miR-145-5p followed by recovery through streptavidin-coated beads evidenced enrichment for LINC00174 vs. control-scrambled oligonucleotide (Fig. 2E).

### LINC00174 favors migration of TC1889 cells

**Fig. 3:**
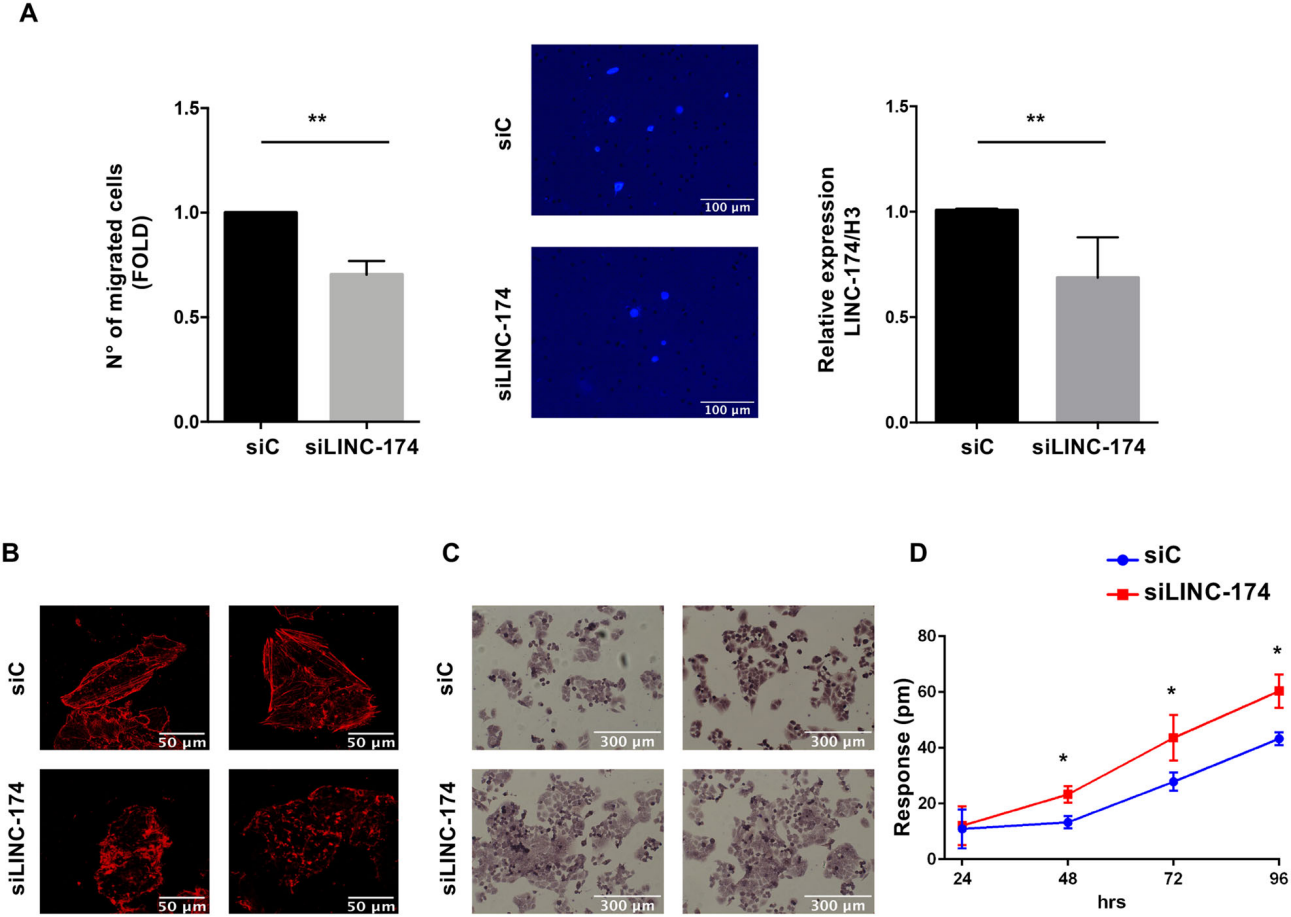
Impact of LINC00174 silencing on migration in TC1889 cell line. **A** TC1889 cells after silencing of LINC00174 (siLINC-174) for 72 h were used for Transwell migration assay (24 h of migration) (*n* = 3, *p*-value = 0.0148) (left, middle panels). Migration has been normalized over cell numbers in the indicated conditions. qRT-PCR analysis of LINC00174 is shown in right graph. **B** Confocal microscopy upon LINC00174 silencing for 72 h after staining with phalloidine. **C** Morphological analysis performed at 72 h showing morphological changes between siC and siLINC-174 samples. **D** Dynamic mass redistribution (DMR) label-free assay to observe the space occupied by cells TC1889 at 24 h, 48 h, 72 h, and 96 h of LINC00174 silencing. Significant *p*-values are indicated as *P < 0.05; **P < 0.005.

To explore the biological role of LINC00174 in thymic epithelial tumors, we first evaluated cell proliferation and migration ability after LINC00174 silencing. By trypan blue exclusion analysis and eFluor dye-based assay, we observed that inhibition of LINC00174 led to a mild reduction of cell growth at 72 h, compared to control samples (Supplementary Fig. 4A, B). Cell cycle and cell death, however, were not affected by LINC00174 silencing (Supplementary Fig. 4C, D). We next explored the effect of LINC00714 expression on cell migration. By transwell cell migration assay, we observed that silencing of LINC00174 significantly reduced cell migration in TC1889 cell line and in thymoma primary cells (Fig. 3A and Supplementary Fig. 5A). Motility of cells has been extensively shown to rely on changes in the expression of cell surface proteins controlling cell adhesion, as for example, the Cadherins (CDH) family. According to the reduced motility of cells, LINC00174-depleted cells presented reduced levels of N-cadherin and increased levels of E-cadherin proteins compared to control cells (Supplementary Fig. 6A, B).

To further support the migration result, we examined the actin cytoskeleton rearrangement, a major feature of migrating cells. We observed a lower actin polymerization and stress fibers formation in the samples silenced for LINC00174 (siLINC-174), compared to control (siC) (Fig. 3B). According to the morphological change of cells observed in siLINC-174 condition, Giemsa-stain showed that cells silenced for LINC00174 tended to form cell clusters while control cells were widespread and separated (Fig. 3C). DMR (Dynamic mass redistribution) assay confirmed that si-LINC00174 cells presented a significant mass redistribution, as shown in Fig. 3D. Cells depleted of LINC00174 occupied a larger area compared to control cells, further supporting what observed in Giemsa staining. No alteration of cell viability was observed in si-LINC00174 condition (Supplementary Fig. 4E).

### Involvement of LINC00174 in lipid metabolism

**Fig. 4:**
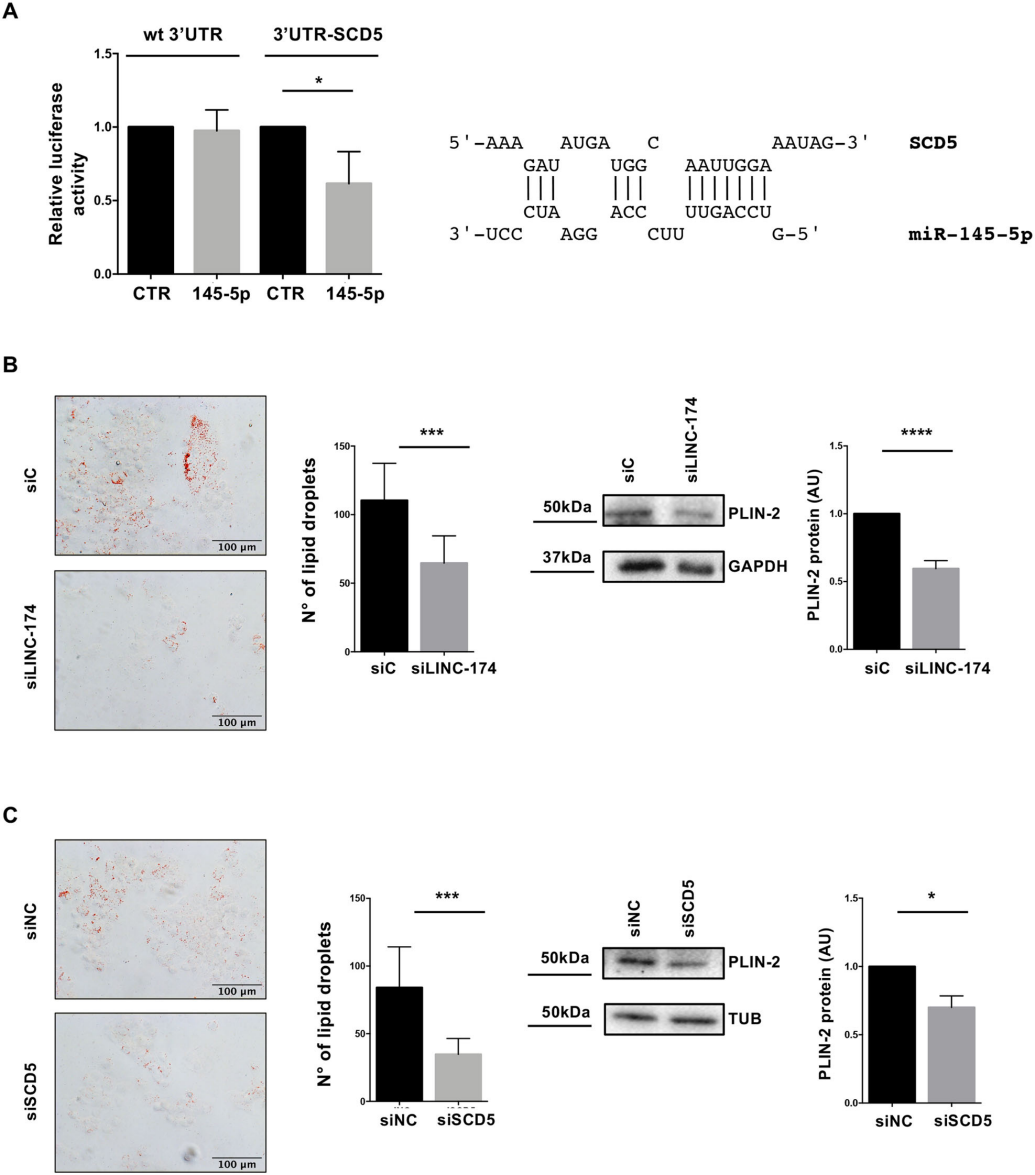
Involvement of LINC00174 and SCD5 in lipid metabolism. **A** Dual luciferase assays in TC1889 cells using a vector enclosing SCD5 3′UTR or control vector (wt-3′UTR) in presence of miR-145-5p mimic oligonucleotide or control mimic. **B**, **C** Oil red assay to count lipid droplets in TC1889 cells upon LINC00174 (**B**) or SCD5 (**C**) silencing for 72 h (*n* = 4) (left). Quantification of lipid droplets was performed by imageJ software. Western blot analysis of Perilipin-2 protein, with relative quantification (*n* = 3), in TC1889 cells upon LINC00174 (**B**) or SCD5 (**C**) silencing for 72 h is shown on the right. Significant p-values are indicated as *P < 0.05; ***P < 0.0005; ****P < 0.00005.

To explore the functional impact of LINC00174 expression in TET, we performed bioinformatic analysis, to highlight the pathways enriched among the genes positively correlated to LINC00174 in our TET cohort. This analysis revealed genes belonging to the lipid metabolism as the most enriched (Table 2). As an increasing amount of literature points to the key role exerted by lipids in the promotion of many features of malignancy, we further dissected this function. Of note, one of the genes positively correlated to LINC00174, presenting a binding site for miR-145-5p, and prognostic in TET, *SCD5*, was among the lipid metabolism-related genes. *SCD5* is a gene that belongs to Stearoyl-CoA desaturase and is involved in the production of unsaturated fatty acids from saturated fatty acids. We first assessed by luciferase assays that SCD5 3’-UTR is targeted by miR-145-5p (Fig. 4A). Next, to evaluate the involvement of LINC00174 and SCD5 in the control of lipids production in thymic carcinoma cells TC1889, we examined, by oil red assay, the amount of lipid droplets upon LINC00174 or SCD5 silencing. As shown in Fig. 4B, C, we observed a reduction of lipid droplets content in cells silenced for LINC00174 or SCD5, compared to control samples. Silencing of SCD5 is shown in Supplementary Fig. 7. According to the reduced lipid droplets content, cells depleted of LINC00174 or SCD5 also showed reduced protein levels of Perilipin-2 (PLIN-2), belonging to the perilipin family^35^ members of which coat intracellular lipid storage droplets (Fig. 4B, C). The ability of SCD5 to increase lipid droplets cell content in thymic carcinoma cells was also confirmed by overexpression experiments using an SCD5 expression vector (Supplementary Fig. 8A–C). In line with the hypothesized LINC00174/miR-145-5p/SCD5 model, we observed that also inhibition of miR-145-5p led to increased content of lipid droplets in TC1889 cells (Supplementary Fig. 8D, E).

**Table 2:** Enriched pathways among the 217 genes positively correlated to LINC00174 in Thymoma IRE cohort and predicted to be target of miR-145-5p; the analysis was performed using ConsensusPathDB tool.

Pathway	*p*-value	*q*-value	members_input_overlap
Metabolism of lipids	0.00032	0.0058	SGPL1; SLC44A3; RAB14; PLD2; PLA2G12A; PCCB; CDS1; CROT; SCD5; ELOVL6; SLC22A5; PCTP; AGPAT3; OSBPL3; OXCT1; ARV1; VAC14; SGPP2
Signaling by receptor tyrosine kinases	0.0024	0.0221	ATP6V0B; SPINT1; NTF4; EPS15L1; ITGA2; ESRP2; ESRP1; CLTC; WWOX; PDPK1; ATP6V1C1; AP2B1
Membrane trafficking	0.0048	0.0287	GPS1; GGA2; EPS15L1; COG5; AP2B1; CUX1; ANK3; AGPAT3; AP1S1; CLTC; CLINT1; DENND1A; STX6; RAB14
Transport of small molecules	0.0063	0.0287	ATP6V0B; FLVCR1; ATP2C1; PMPCB; WNK2; STEAP2; SLC22A5; SLC44A3; CLTC; MCOLN3; ATP6V1C1; TRPM7; SLC30A1; AP2B1; SLC46A1
Vesicle-mediated transport	0.0083	0.0302	GPS1; EPS15L1; COG5; AP2B1; DENND1A; CUX1; ANK3; AGPAT3; AP1S1; CLTC; CLINT1; GGA2; STX6; RAB14

These results confirmed that the LINC00174/miR-145-5p/SCD5 axis is involved in lipid metabolism pathway in thymic carcinoma.

### SCD5 impacts on the motility of thymic carcinoma cells

Although the biological activity of SCD5 is still poorly characterized, it has been reported as a pro-survival factor in breast cancer cells^36^, and implicated in the epithelial-mesenchymal reversion in advanced melanoma^37^. Moreover, lipids synthesis has been reported not only to increase cell proliferation, but also to promote migration and invasion^29,38^.

**Fig. 5:**
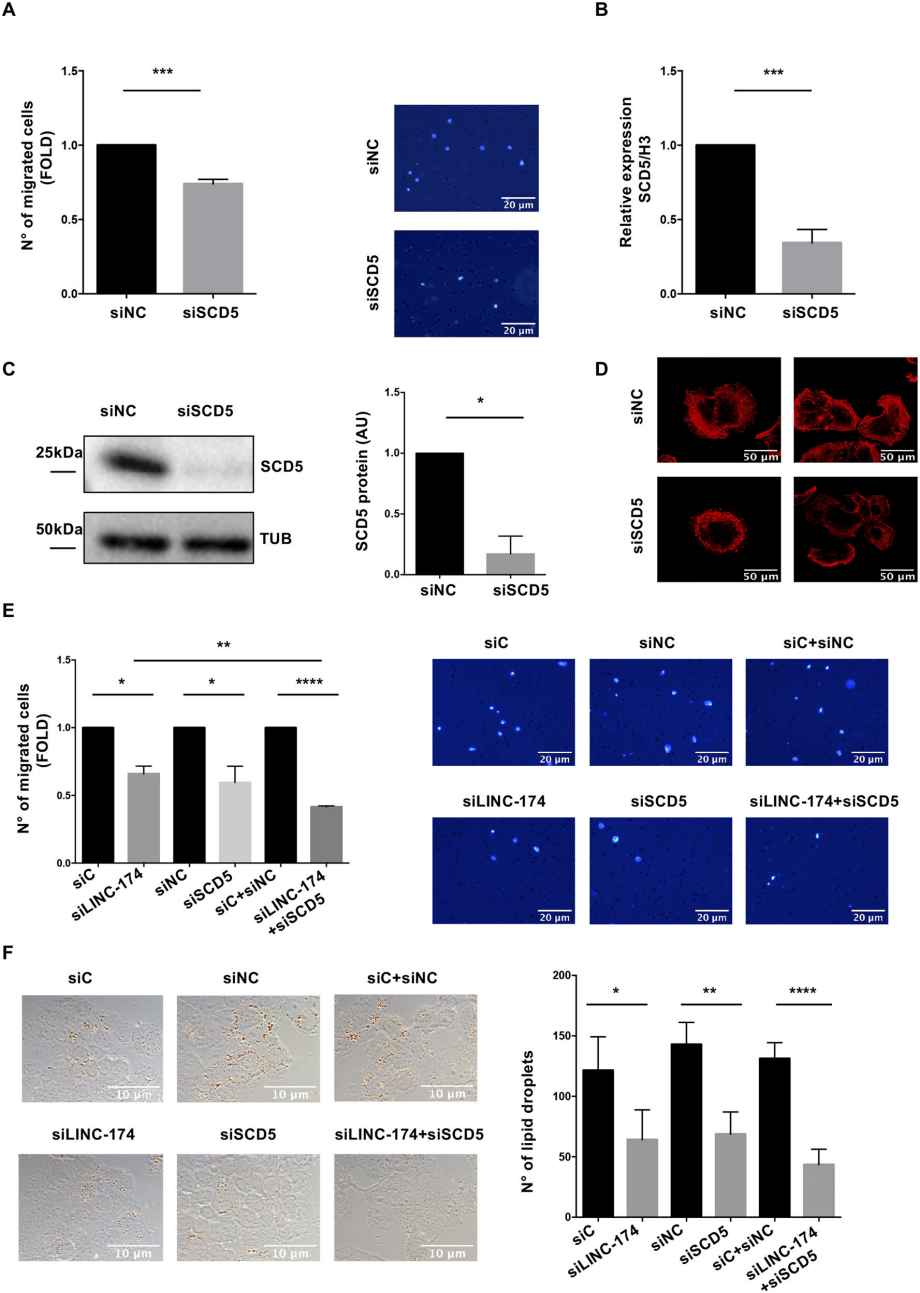
Silencing of SCD5 impacts on migration and lipid droplets content of TC1889 cells. **A** Transwell migration analysis to evaluate differences in motility upon SCD5 silencing (*n* = 3, *p* value = 0.0005) in TC1889 cells. Migration has been normalized over cell numbers in the indicated conditions. **B** RT-qPCR and **C** western blot analysis to verify silencing of SCD5 at transcript (*n* = 3, *p* value = 0.0003) and protein (*n* = 3, *p* value = 0.035) levels. **D** Confocal microscopy upon SCD5 silencing for 72 h after staining with phalloidine. **E** Transwell migration assay after 72 h of double or single depletion of LINC00174 and/or SCD5. Migration has been normalized over cell numbers in the indicated conditions. **F** Oil red assay for lipid droplets counting in the same conditions as in (**E**). Quantification of lipid droplets by imageJ software. Significant *p*-values are indicated as *P < 0.05; **P < 0.005; ***P < 0.0005; ****P < 0.00005.

On this basis, we evaluated if SCD5 is involved in tumor cell migration in TET. We observed that the depletion of SCD5 led to reduction of cell migration compared to control samples (Fig. 5A–C). This result was confirmed using a second siRNA for SCD5 (Supplementary Fig. 9A). To analyze the features of migratory cancer cells, we performed an immunofluorescence with phalloidine. As shown in Fig. 5D, SCD5 depletion decreased stress fibers formation and actin polymerization, supporting a role for SCD5 in the control of cell motility. On the contrary, SCD5 silencing didn’t impact cell growth (Supplementary Fig. 7A–C and Supplementary Fig. 9B) in TC1889 cells.

Given the similarity between the results obtained upon LINC00174 or SCD5 silencing, and their connection to lipid metabolism and cell migration in TC1889 cells, we next examined the effect of the combined depletion of these two factors and observed a much stronger and significant reduction of cell migration and lipid droplets cell content in the double-depleted compared to single-depleted cells (Fig. 5E, F and Supplementary Fig. 10). Moreover, reduction of migration after LINC00174 silencing was rescued by overexpression of SCD5 protein (Supplementary Fig. 11A, B).

Since our data highlighted the prognostic power of LINC00174 and of its associated signature, we next explored if any compound exists that impinges on LINC00174-associated signature as this approach may present therapeutic efficacy in TET. To this end, we used the 128-genes signature associated to LINC00174 (Supplementary Table 3, sheet 3) to interrogate Connectivity Map (https://clue.io) derived from the Library of Integrated Network-based Cellular Signatures (LINCS) database (http://lincs.hms.harvard.edu/) and containing an extensive catalogue of gene-expression profiles generated from several human cancer cells in response to more than 27,900 perturbations. This approach highlighted a number of drugs potentially able to downregulate the LINC00174-associated signature (Supplementary Table 3, sheet 6). Interestingly, we identified several compounds that could be considered for TET treatment. Of note, the drug showing highest score was catechin, a type of natural phenol and antioxidant, which is also involved in the inhibition of fatty acid synthase^39^. This result further supports that blocking of fatty acids synthesis pathway may represent a valuable approach for TET treatment that is worth exploring in future studies.

## Discussion

In this present study, starting from gene expression analysis of thymic tumors and normal tissues, we observed a network of positively correlated pairs of lncRNAs/mRNAs that shared a binding site for miR-145-5p, a well-known tumor suppressor downregulated in TETs. Among them, only LINC00174 and a 5-genes mRNAs signature showed a significant prognostic value in TET patients. Interestingly, an oncogenic role for LINC00174 has been previously reported in several cancer types^27,40,41^.

The positive correlation between LINC00174 and LINC00174-associated mRNA signature and their modulation of expression upon overexpression or inhibition of miR-145-5p, suggest a role for this lncRNA as a sponge for miR-145-5p in TET. Indeed, one of the mechanisms of action of lncRNAs is based on their interaction with microRNAs, through miRNA-binding sites, and their ability to function as competing endogenous RNAs (ceRNAs)^42^. Many authors demonstrated the potential role of lncRNAs as ceRNAs^43–48^. The observation that more than half of the amount of LINC00174 present in cells is localized in the cytoplasm and the assessment of the interaction between LINC00174 and miR-145-5p further supported the possible role of LINC00174 as an endogenous sponge for miR-145-5p. Previous studies had demonstrated the ability of LINC00174 to act as sponge for miR-152-3p in glioma^40^ and for miR-320 in hepatocellular carcinoma^41^, to promote tumorigenesis.

We previously reported that miR-145-5p is epigenetically downregulated in thymic tumors^32^. Data from this study suggest that additional layers of control to inhibit miR-145-5p are present in TET cells to ensure a more efficient block of miR-145-5p tumor suppressor activity.

It’s noteworthy that silencing of LINC00174 reduced cell proliferation, migration, and lipid droplets accumulation in TET cells. These data support the association between cell migration and the alteration of lipid metabolism. The regulation of actin dynamics and assembly/disassembly of focal adhesions drive cell migration^49^. A variety of studies have reported the involvement of lipid metabolism in tumorigenesis, revealing it as a predictive marker of cancer^29,50^. De novo fatty acids synthesis is an essential process for membrane production, cell growth and proliferation^51^. Many studies described that overexpression of enzymes involved in fatty acids synthesis stimulates migration and invasion of cancer cells, indicating a strong association between these two tumor pathways^29,31^. Of interest, among the 5 prognostic genes positively correlated to LINC00174, and predicted to be targeted by miR-145-5p, there was SCD5 gene, also involved in lipid metabolism. SCD5 is a steroyl-Co-desaturase (SCD) implicated in unsaturated fatty acids synthesis^36^. According to the association between lipid metabolism and cell migration, we observed that SCD5 in TC1889 cells increased the amount of lipid droplets as well as migration. We showed also that SCD5 exogenous expression is able to rescue the migration decrease observed upon LINC00174 silencing.

Altogether these data indicate that both genes could contribute to increase phenotype of cancer and as LINC00174’s role in cancer might be mediated by action of miR-145-5p on target genes, involved in different cellular pathway, as SCD5. It might represent an additional evidence of LINC00174’s activity as sponge for miR-145-5p. This study suggests as LINC00174 expression may become a valuable prognostic biomarker, opening the possibility to new potential therapeutic targets clinically relevant for treatment of thymic epithelial tumors.

## Materials and methods

### Cell culture and transfection

Human Thymic Carcinoma cell line TC1889^52^ was cultured in RPMI 1640 (Gibco® Thermo Fisher Scientific, Waltham, MA USA) containing 4.5 g/L glucose, 25 mM Hepes, 50 U/mL penicillin, 50 U/ml streptomycin and 10% heat-inactivated South-American Fetal Bovine Serum (FBS) (Gibco® Thermo Fisher Scientific, Waltham, MA, USA) at 37 °C in incubator with humidified 5% CO_2_ atmosphere.

For the overexpression and inhibition of miR-145-5p, Pre-miRNA-145 (#AM17100, Thermo Fisher Scientific, Waltham, MA USA), Pre-miRNA Precursor Negative Control (#AM17110, Thermo Fisher Scientific, Waltham, MA, USA), hsa-miR-145-5p mirVana miRNA inhibitor (#MH11480, Thermo Fisher Scientific, Waltham, MA, USA) and Negative Control #1 (#4464077, Thermo Fisher Scientific, Waltham, MA, USA) were transiently transfected at final concentration of 5 nM using Lipofectamine RNAiMAX (Gibco® Thermo Fisher Scientific, Waltham, MA, USA) according to the manufacturer’s instructions.

For the silencing of LINC00174 lncRNA, TC1889 cell line was transiently transfected with the following antisense LNA-GapmeR: for LINC-00174 ATCGTCGCTTGGAGAG (LG00197933-DDA) and CCGAACTGAGGAATTT (LG00197935-DDA); for Negative Control NC1 AACACGTCTATACGC (LG00000002-DDA) (Qiagen Chatsworth, CA) at final concentration of 20 nM.

For gene silencing, TC1889 cell line was transiently transfected with Dicer-substrate short interfering RNAs (DsiRNAs) for SCD5 (IDT, Belgium) or Negative Control (IDT, Belgium) at 500 pM using Lipofectamine RNAiMAX (Gibco® Thermo Fisher Scientific, Waltham, MA USA) according to the manufacturer’s instructions. The duplex sequences for SCD5.1: 5′-GGA GAA AGC UUG ACG UCA CUG ACC T-3′ and 3′-CCC CUC UUU CGA ACU GCA GUG ACU GGA-5′; for SCD5.2: 5′-AAG CUG CCU CUG AGG AUA UUU CUG G-3′ and 3′-GGU UCG ACG GAG ACU CCU AUA AAG ACC-5′.

For the overexpression of biotinylated miR-145-5p oligonucleotide, TC1889 cell line was transiently transfected with miRCURY LNA microRNA mimics, Premium, Biotin hsa-miR-145-5p (Exiquon, Denmark) and as a negative control miRCURY LNA microRNA mimics, Premium, Biotin hsa-miR-39-3p (Exiquon, Denmark) at 5 nM using Lipofectamine RNAiMAX (Gibco® Thermo Fisher Scientific, Waltham, MA USA) according to the manufacturer’s instructions.

### Total RNA extraction from tissues, labeling, and microarray

The following fresh frozen specimens were analyzed: four thymomas of different histotypes and two thymi derived from the Pathology Department of IRCCS Regina Elena National Cancer Institute, Rome, Italy; two thymomas and one thymus derived from “Policlinico Umberto I” Hospital, Rome, Italy. The thymomas frozen samples included: one type A, two type AB, two type B2, one type B3. The normal thymic counterparts included peritumoral thymic tissue of the same frozen series. This study was approved by the Institutional Review Board of “Policlinico Umberto I” Hospital (Rif 3262/26.06.2014 Prot. N° 815/14) and the Regina Elena National Cancer Institute (Rif 383/28.5.2013 Prot. N°5/13 and Rif 447/20.06.2013 Prot. N°7/13).

Fresh frozen samples were homogenized by gentleMACSdissociator (Miltenyi Biotec, Bologna, Italy) in 700 μl of Qiazol (Qiagen, Chatsworth, CA) and RNA was extracted using the miRNAeasy® kit (Qiagen, Chatsworth, CA) following the manufacturer’s instructions. Total RNA for each specimen was used for microarray analysis of mRNAs expression on Affymetrix platform. Specifically, 100 ng of total RNA was labeled and hybridized on Affymetrix® Human Gene 2.0 ST Arrays 2.0 (Affymetrix, Santa Clara, California). Scanning and image analysis were performed using the Affymetrix GeneChip 3000 Scanner according to the Affymetrix GeneChip WT Terminal Labeling and Hybridization User Manual.

### Bioinformatics analysis of expression data from TCGA and IRE (Istituto Regina Elena) Thymoma cohorts

Signals from gene expression profiling of 6 tumors and 3 normal samples from the IRE cohort were background adjusted and quantile normalized. A permutation test and a false discovery procedure (Storey, 2002) were used to identify most deregulated genes between tumoral and normal samples. Unsupervised hierarchical clustering was performed on deregulated features in order to discover groups of samples with different histotype. The entire signal processing analyses and statistical tests were performed by Matlab (The MathWorks Inc.). Raw data of gene expression profiling are available in GEO database (GSE158997).

Among deregulated genes, a Spearman correlation coefficient was evaluated for each LNC\gene pairs that resulted to be predicted as putative target of the miR-145-5p. Target prediction was assessed by the web server tool MirWalk2 (http://zmf.umm.uni-heidelberg.de/apps/zmf/mirwalk2/).

Positive correlated pairs were validated in TCGA thymoma cohort of 119 normalized samples.

Overall survival of selected features was evaluated by Kaplan–Meier method and multivariate Cox proportional hazard regression model. To obtain a symmetrical split of the patients we considered the median of the distribution. High and low expression value of each feature was established by positive and negative *z*-scores, respectively. The log-rank test was used to assess differences between curves. Significance was defined at the *p* < 0.05 level. The Hazard Risk (HR) and the 95% confidence intervals (95% CI) was estimated for each variable.

### Total RNA extraction from cells, cDNA reverse transcriptase, and RT-qPCR

Total RNA was extracted using Trizol Reagent (Invitrogen), following the manufacturer’s protocol and was reverse transcribed using High-Capacity RNA-to-cDNA Kit (Applied Biosystems). Quantification of gene expression was measured by Sybr Green assay (Applied Biosystems, Carlsbad, CA, USA) on Abi Prism 7500 (Applied Biosystems). cDNA reverse transcriptase of miR-145-5p was performed using miScript II RT kit (Qiagen, Chatsworth, CA). Quantification of miR-145-5p was carried out by miScript SYBR Green PCR kit (Qiagen, Chatsworth, CA), using miScript Primer Assay: Hs_miR-145_1 (miScript Primer Assay MS00003528 Qiagen, Chatsworth, CA), and Hs_RNU6_B (miScript Primer Assay MS00033740 Qiagen, Chatsworth, CA) as normalizer, on Abi Prism 7500 (Applied Biosystems). All reactions were performed in duplicates.

### Statistical analysis

The statistical analyses were performed using GraphPad Prism (GraphPad Software, San Diego, California, USA). Comparisons between two samples were analyzed through student’s *T* test (unpaired, two-tailed). Value of *P* ≤ 0.05 and *P* ≤ 0.01 was considered statistically significant.

## Supplementary information

SUPPLEMENTAL MATERIALS AND METHODS

Supplementary Figure 1

Supplementary Figure 2

Supplementary Figure 3

Supplementary Figure 4

Supplementary Figure 5

Supplementary Figure 6

Supplementary Figure 7

Supplementary Figure 8

Supplementary Figure 9

Supplementary Figure 10

Supplementary Figure 11

Supplementary Table 1

Supplementary Table 2

Supplementary Table 3_Sheet 1

Supplementary Table 3_Sheet 2

Supplementary Table 3_Sheet 3

Supplementary Table 3_Sheet 4

Supplementary Table 3_Sheet 5

Supplementary Table 3_Sheet 6
