# Supplementary material for: LINC00174 is a novel prognostic factor in thymic epithelial tumors involved in cell migration and lipid metabolism
Source: Cell Death Dis. 2020 Nov 7;11(11):959. doi: 10.1038/s41419-020-03171-9 (PMC7648846; doi:10.1038/s41419-020-03171-9)
Supplement: Supplementary file 1 — SUPPLEMENTAL MATERIALS AND METHODS [file 41419_2020_3171_MOESM1_ESM.docx]

**SUPPLEMENTAL MATERIAL AND METHODS**

**Cell culture and transfection**

Primary cell cultures from human thymoma and normal thymic counterpart (peritumoral thymic tissue) were established from thymus obtained by surgical resection from a patient with thymoma type AB. Specifically, the tumor or normal tissue was cut into small pieces, washed in PBS 1X (Gibco® Thermo Fisher Scientific, Waltham, MA USA) and placed in a 60 mm dish with EBM^TM^-2 Basal Medium (CC-3156, Lonza, Pharma & Biotech USA) and EGM^TM^-2 SingleQuots^TM^ Supplements (CC-4176, Lonza, Pharma & Biotech USA), supplemented with 10% FBS (Gibco® Thermo Fisher Scientific, Waltham, MA USA ), 1% penicillin and streptomycin (Gibco® Thermo Fisher Scientific, Waltham, MA USA ), at 37 °C in incubator with humidified 5% CO2 atmosphere. The medium was changed every 3 days. After 10 days the explants were removed, and the adherent cells were detached using a solution of PBS containing 0.05/0.02% trypsin/EDTA and subcultured in the same medium. Cytokeratin staining was performed to evaluate the proportion of epithelial cells in the culture. Specifically, cells grown to 70% confluence into a 35-mm dish containing coverglass were fixed with 4% formaldehyde, permeabilized and incubated with primary antibody to cytocheratin-19 (CK19+), diluted in PBS containing 5% BSA. CK19+ is a histological marker of thymic epithelial cells, expressed in all thymomas.

**Overexpression of SCD5**

For the overexpression of SCD5, TC1889 cell line was transiently transfected with a pCDNA3-SCD5 vector previously reported (Puglisi R et al., Oncotarget 2018; 9:7567-7581). Transfections were performed using Lipofectamine 3000 (Life Technologies, Carlsbad, CA, USA) according to the manufacturer’s instructions.

**Nucleocytoplasmic extracts preparation**

TC1889 cells were harvested and collected in RPMI and divided for RNA and protein extracts. For RNA extraction, cell lysates were prepared with Buffer A (20 mM Tris-Hcl pH 8.0, 10 mM NaCl, 3 mM MgCl2, 0,1% Nonidet P-40, 10% glicerolo, 0.2 mM EDTA, and fresh protease inhibitors plus 1mM DDT), and centrifuged at 300x g for 5 minutes to separate nuclei and cytosolic fraction. The supernatant (cytosolic fraction) was collected in a new tube, while pellet (nuclear fraction) was washed with PBS at 1200 ×rpm for 5 minutes. Total RNA from cytosolic and nuclear extracts was extracted with TRIzol Reagent (Invitrogen) according to the manufacturer’s instructions. For protein extracts, cell lysates were prepared with hypotonic buffer (50 mM HEPES; 150 mM NaCl; 2 mM EDTA; 10 mM MgCl2, 5 mM KCL; 0.5% Nonidet P-40; and fresh protease inhibitors) to obtain cytoplasmic fraction and 2% SDS buffer (2% SDS, 25 mM Tris-Hcl pH 7.5, 100 mM Nacl, 3 mM EDTA, 7% Glycerol and fresh protease inhibitors) for nuclear fraction. Mouse anti-GAPDH 6C5 (#sc32233, Santa Cruz Biotechnology) and rabbit polyclonal anti Histone H3 (#Abcam 1791) were used as markers for the cytoplasmic and nuclear fractions, respectively.

**Protein lysate preparation and immunoblotting analysis**

Cells were suspended in 2% SDS buffer (2% SDS, 25 mM Tris-Hcl pH 7.5, 100 mM Nacl, 3 mM EDTA, 7% Glycerol) and fresh protease inhibitors. Western blot analysis was carried out using the following primary antibodies: anti-GAPDH 6C5 (#sc32233, Santa Cruz Biotechnology); mouse monoclonal anti-Tubulin B512 (#T5168, Sigma-Aldrich, Milan, Italy); a specific polyclonal rabbit antibody was generated against a human SCD5 synthetic peptide (aa 313-327) (Eurogentec Group, Liege, Belgium); rabbit polyclonal anti-CDH2 (N-cadherin) (#ab18203, Abcam, Cambridge, UK); mouse monoclonal anti-CDH1 (E-cadherin) (#sc8426, Santa Cruz Biotechnology); mouse polyclonal anti-PLIN2 (#SAB4200452, Sigma-Aldrich, Milan, Italy). As secondary antibodies goat anti-mouse and anti-rabbit conjugated to horseradish peroxidase were used (Bethyl Laboratories). Protein signals were developed by ECL detection using a ChemiDoc-It Imaging System (UVP, Upland, CA) instrument.

**Immunocytochemistry and immunofluorescence analysis**

After 72h of transfection in 6-well plates, TC1889 cells were harvested, fixed with 4% formaldehyde and permeabilized with 0.01% Triton X-100 in PBS.

For immunocytochemistry analysis, cells were blocked for 30 minutes with 5% BSA and incubated overnight with the indicated primary antibodies diluted in 5% BSA/PBS. Cells were then incubated with peroxidase inhibitor conjugated to secondary antibody. Finally, slides were incubated with DAB (3,3'-diaminobenzidine) that produces enzymatic reaction in the presence of peroxidase, while nuclei were counterstained with hematoxylin.

For immunofluorescence analysis, cells were incubated with Rhodamine Phalloidin (#R415 Thermo Fisher Scientific, Waltham, MA USA) diluted in PBS (Thermo Scientific) for 1h at room temperature (RT). Then, nuclei were counterstained with Hoechst 33342 (Life Technologies).

**Cell cycle and cell death analysis**

For cell cycle analysis, 2x10^5^ cells were resuspended in 50% FCS, fixed in 70% ethanol for 24h, incubated with 50 μg/ml propidium iodide (Sigma-Aldrich) and 50 units/ml Dnase-free RNase A (Sigma-Aldrich) and analyzed after 3h (1x10^4^ events) using an Epics XL Cytometer (Beckman Coulter). For cell death analysis, cells were resuspended in propidium iodide (Sigma-Aldrich) and analyzed through Epics XL Cytometer (Beckman Coulter).

**Cell proliferation Efluor analysis**

Cell proliferation efluor assay was analyzed using a Dye Efluor 670 (Thermos Fisher), a red fluorescent dye that can be used to monitor individual cell division, following the manufacturer’s protocol. Analysis was performed using an Epics XL Cytometer (Beckman Coulter).

**Morphological analysis and dynamic mass redistribution label free assay**

Morphological analysis was performed by the Wright-Giemsa-staining according to the manufacturer’s instructions. TC1889 cells were seeded and transfected in specially designed 384-well plate with highly precise optical sensors able to measure changes in light refraction resulting from dynamic mass redistribution within the cell’s monolayer. Change in the light refraction was indicated by a shift in wavelength.

**ATPlite Luminescence Assay System**

ATPlite™ Luminescence Assay was performed by using a 96-well plate with 2x10^4^ cells/well and cell viability was evaluated following the manufacturer’s instructions. Luminescence was read by the EnSpire® Multimode Plate Reader (PerkinElmer, Whaltman, MA, USA).

**Transwell migration assay**

Migration assay was performed using a 24-well plate with a non-coated 8-μm pore size filter in the insert chamber (BD Falcon, Franklin Lakes, NJ, USA). After 72h from transfection, 4x10^4^ cells were resuspended in RPMI medium without FBS and seeded into the insert chamber. Cells were allowed to migrate for 24h into the bottom chamber containing 0.7 ml RPMI media without FBS containing EGF 30 ng/ml in a humidified incubator at 37°C in 5% CO2. Cells were fixed with 4% formaldehyde in PBS and were permeabilized with 0.01% Triton X-100 in PBS for 10 min. Staining with DAPI allowed the counter of cells migrated under the bottom of filter. Statistical analysis was performed by normalizing the number of migrated cells on cell number.

**Oil red staining**

Cells were seeded and transfected in 6-well plates for 72h. Then, TC1889 cells were fixed with 4% formaldehyde in PBS for 15 min at room temperature (RT) and washed with 60% isopropanol for 5 min at RT. Cells were let dry completely at RT. Oil red staining was performed by preparation of Oil Red O working solution and incubation at RT for 10 min. Cells were washed 4 times with ddH2O and images were acquired under the microscope for analysis.

**RNA-RNA interaction assay**

A total of 20x10^5^ cells were seeded and transfected with biotynilated miR-145-5p. After 48h, cells were harvested and resuspended in lysis buffer (500 mM NaCl, 50 mM Tris-Cl pH 7, 100mM EDTA, 1% SDS) supplemented with fresh Protease Inhibitor, PMSF and RNase inhibitor Superase In (Invitrogen). RNA recovery was performed using DynabeadsTM MyOneTM Streptavidin C1 Beads (#65002 Invitrogen). Total RNA was extracted using Trizol Reagent (Invitrogen) and was reverse transcribed using High-Capacity RNA-to-cDNA Kit (Applied Biosystems). Quantification of gene expression was measured by Sybr Green assay (Applied Biosystems, Carlsbad, CA, USA) on Abi Prism 7500 (Applied Biosystems)

**Plasmid construction and dual-luciferase reporter assay**

The luciferase reporter assay was performed by using a psiCHECK-2 vector (C8021; Promega, Madison, WI, USA) previously generated (Puglisi R et al., Oncotarget 2018; 9:7567-7581) containing a 551 bp fragment of 3'-UTR which encloses the binding site for miR-145-5p. Briefly, TC1889 were transfected with 40 ng of the vectors and 1 nM of Pre-miRNA-145 (#AM17100, Thermo Fisher Scientific, Waltham, MA USA) or Pre-miRNA Precursor Negative Control (#AM17110, Thermo Fisher Scientific, Waltham, MA USA) as indicated for 48h, using Lipofectamine 2000 (Gibco® Thermo Fisher Scientific, Waltham, MA USA) according to the manufacturer’s instructions. The Renilla and Firefly, as control, luciferase activity was measured by using the dual-luciferase assay system (E1910; Promega, Madison, WI, USA).

**Free fatty acids quantification**

After 72h of transfection in 6-well plates, a total of 1x10^6^ TC1889 cells were harvested and homogenized in 200 μL of a 1% (w/v) Triton X-100 in chloroform solution, following the manufacturer’s protocol. The concentration of fatty acids was determined by fluorometric assays, measuring fluorescence intensity (λex = 535/λem = 590 nm).

**Identification of potential inhibitors of LINC00174-associated expression signature**

The LINC00174-associated signature was used to interrogate the Library of Integrated Network-based Cellular Signatures (LINCS; http://www.lincsproject.org/) through Connectivity Map (data version 1.1.1.2 and software version 1.1.1.38; https://clue.io/) to identify compounds whose administration to cancer cells results an opposite expression profile of the 128-gene signature associated to LINC00174. Briefly, Connectivity Map generates a list of compounds rank-ordered by the similarity of differentially-expressed genes in treated cells to the query gene signature and characterized by a score that ranges from -100 to 100 (based on the overlap with up- or down-regulated genes after the perturbation and the strength of the enrichment). In particular, a negative score indicates that the compound and the query signatures are opposing, i.e. genes of query signature are decreased by treatment with the compound. The magnitude of the score corresponds to the magnitude of similarity or dissimilarity. Drugs with negative score (i.e. drugs that revert the signature expression levels) were filtered retaining those with a score lower than the 90th percentile of all scores.
