# Supplementary figures and images for: LINC00174 is a novel prognostic factor in thymic epithelial tumors involved in cell migration and lipid metabolism
Source: Cell Death Dis. 2020 Nov 7;11(11):959. doi: 10.1038/s41419-020-03171-9 (PMC7648846; doi:10.1038/s41419-020-03171-9)

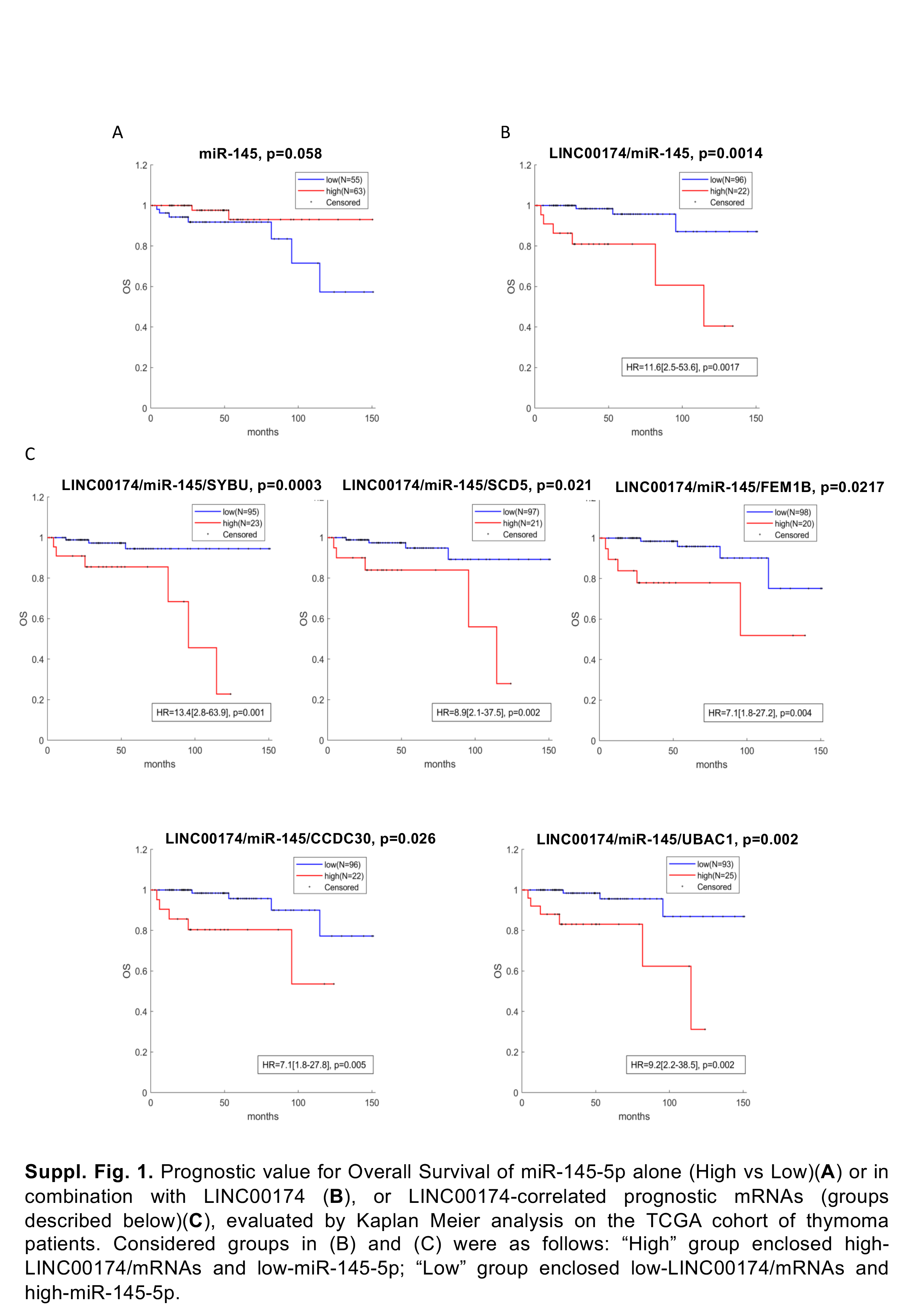

Supplement: Supplementary file 2 — Supplementary Figure 1 [file 41419_2020_3171_MOESM2_ESM.png]

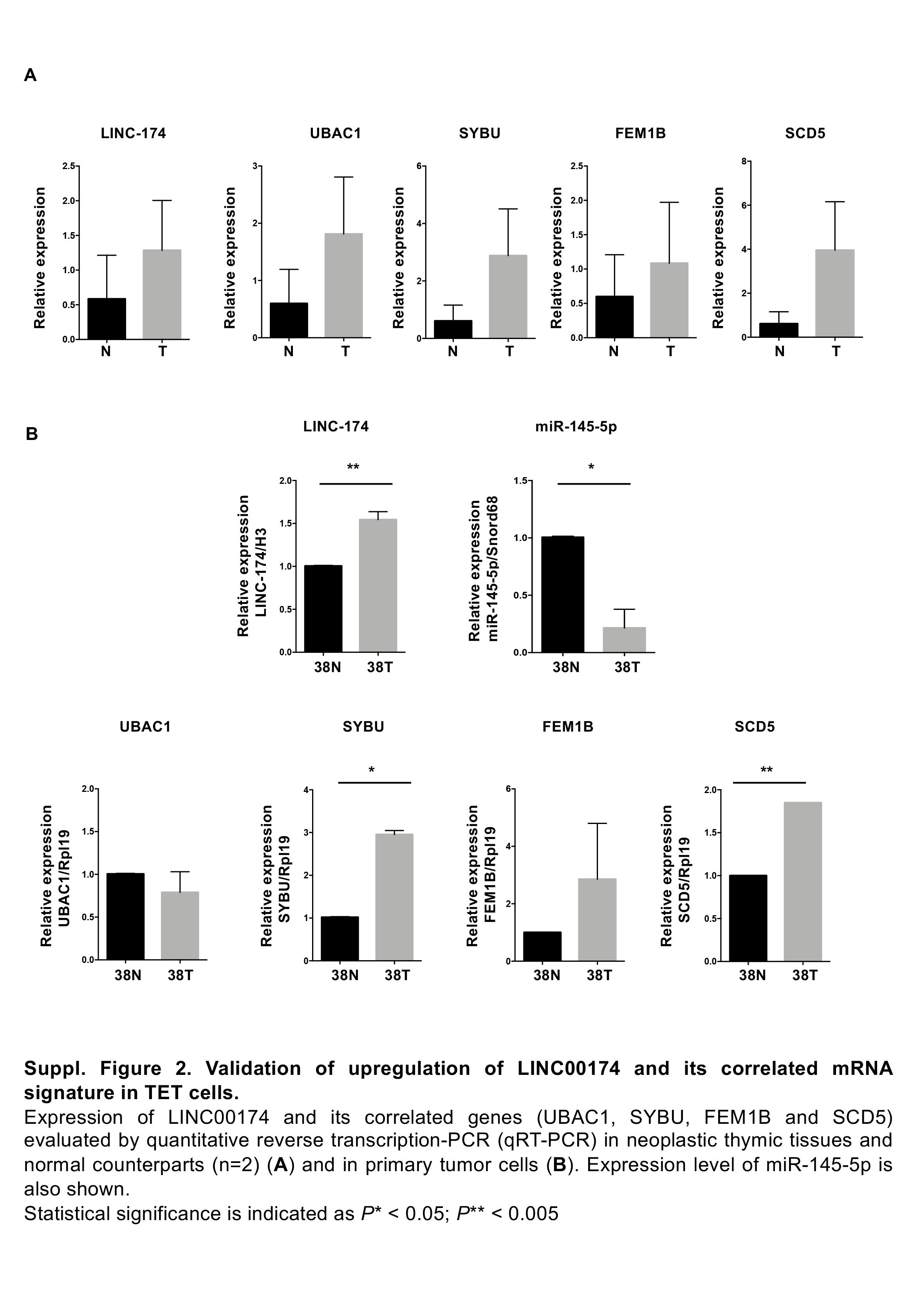

Supplement: Supplementary file 3 — Supplementary Figure 2 [file 41419_2020_3171_MOESM3_ESM.png]

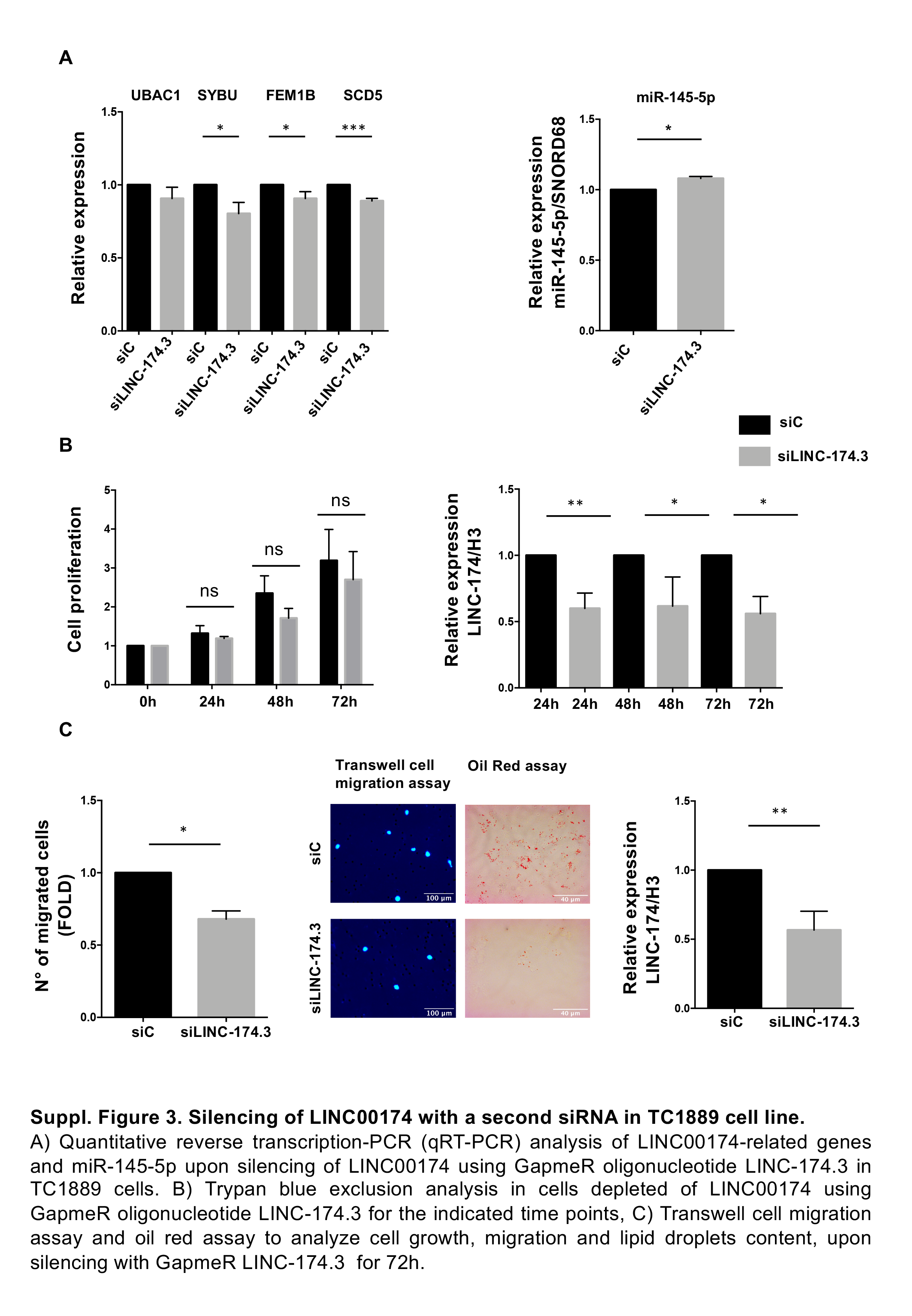

Supplement: Supplementary file 4 — Supplementary Figure 3 [file 41419_2020_3171_MOESM4_ESM.png]

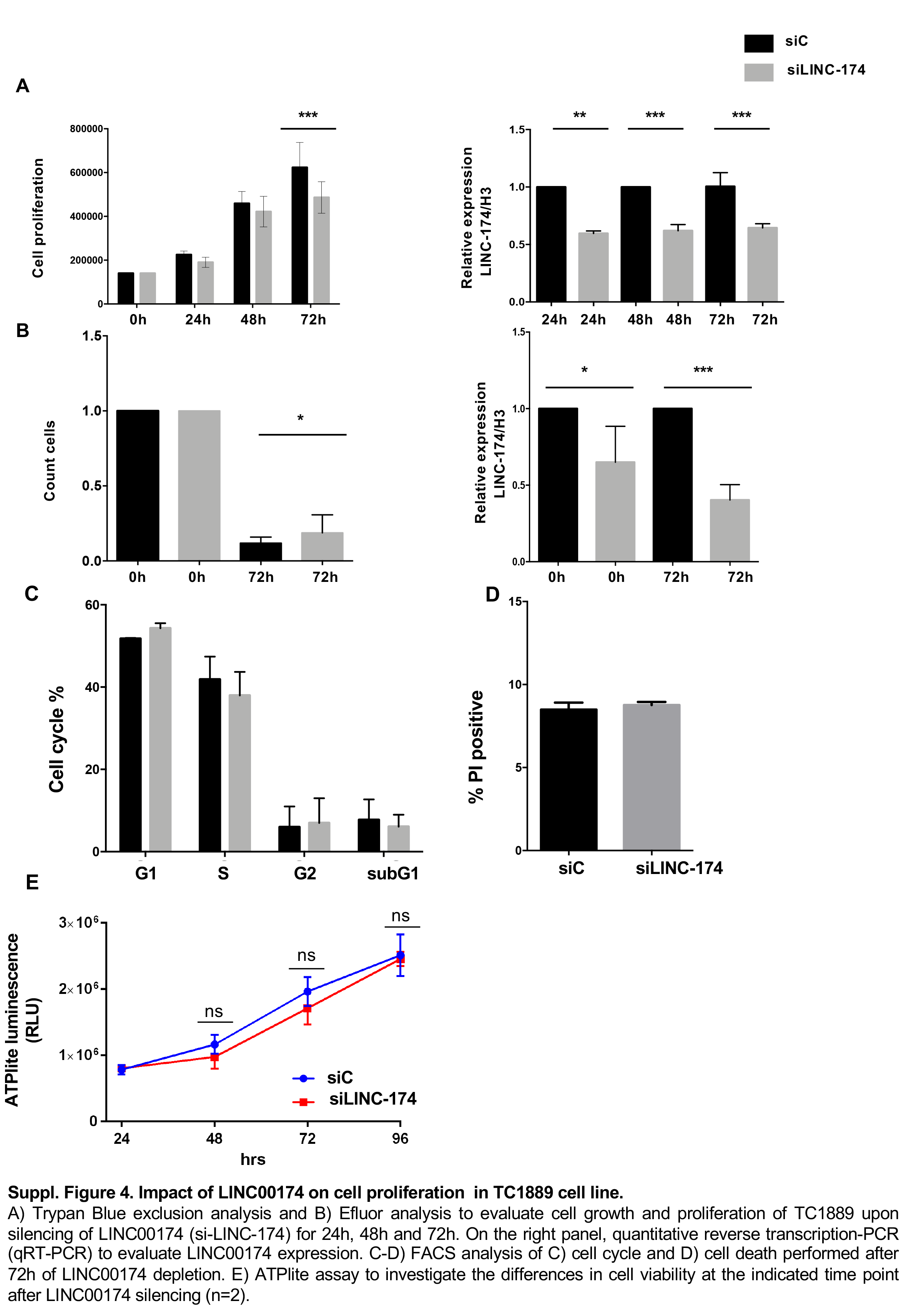

Supplement: Supplementary file 5 — Supplementary Figure 4 [file 41419_2020_3171_MOESM5_ESM.png]

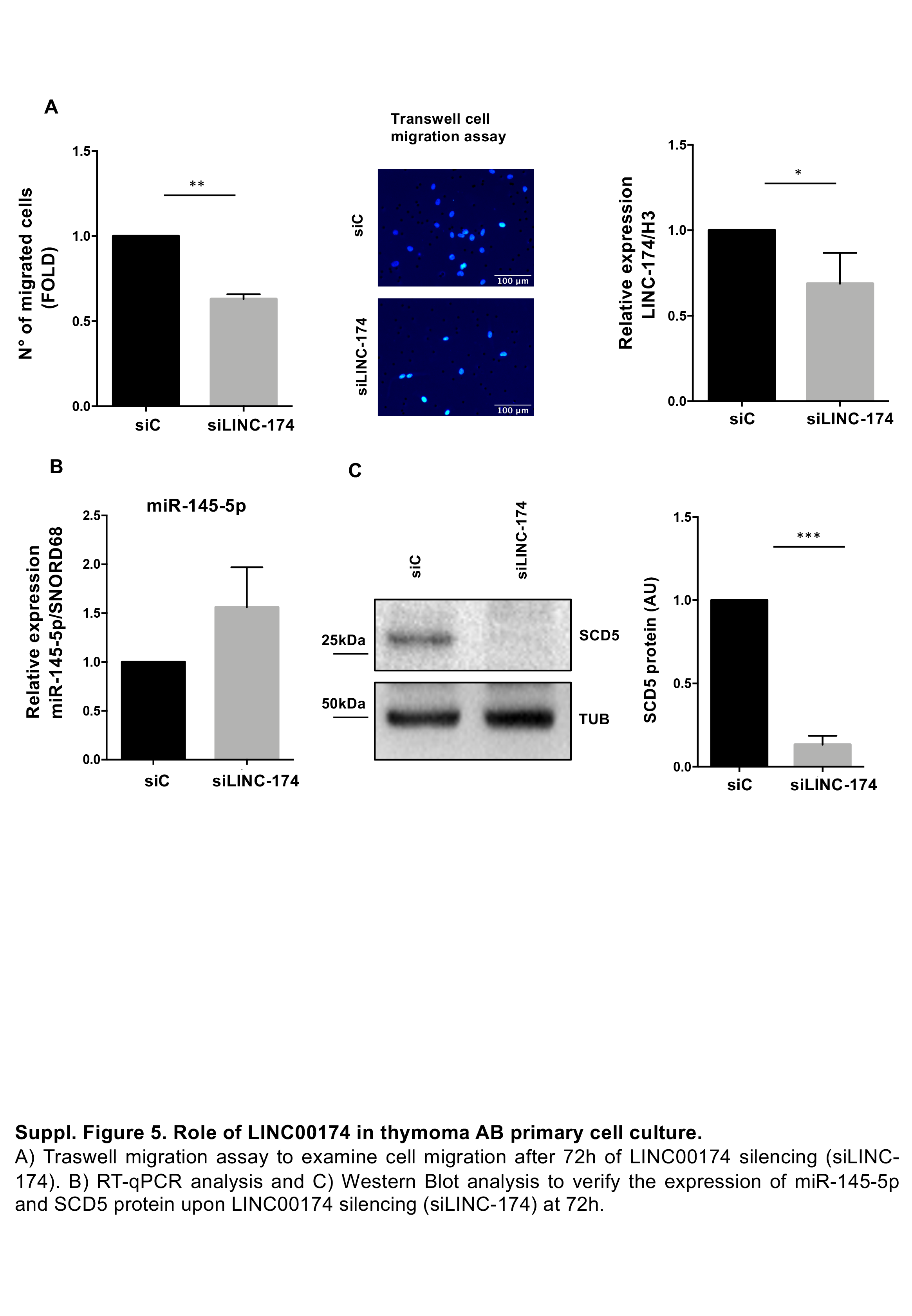

Supplement: Supplementary file 6 — Supplementary Figure 5 [file 41419_2020_3171_MOESM6_ESM.png]

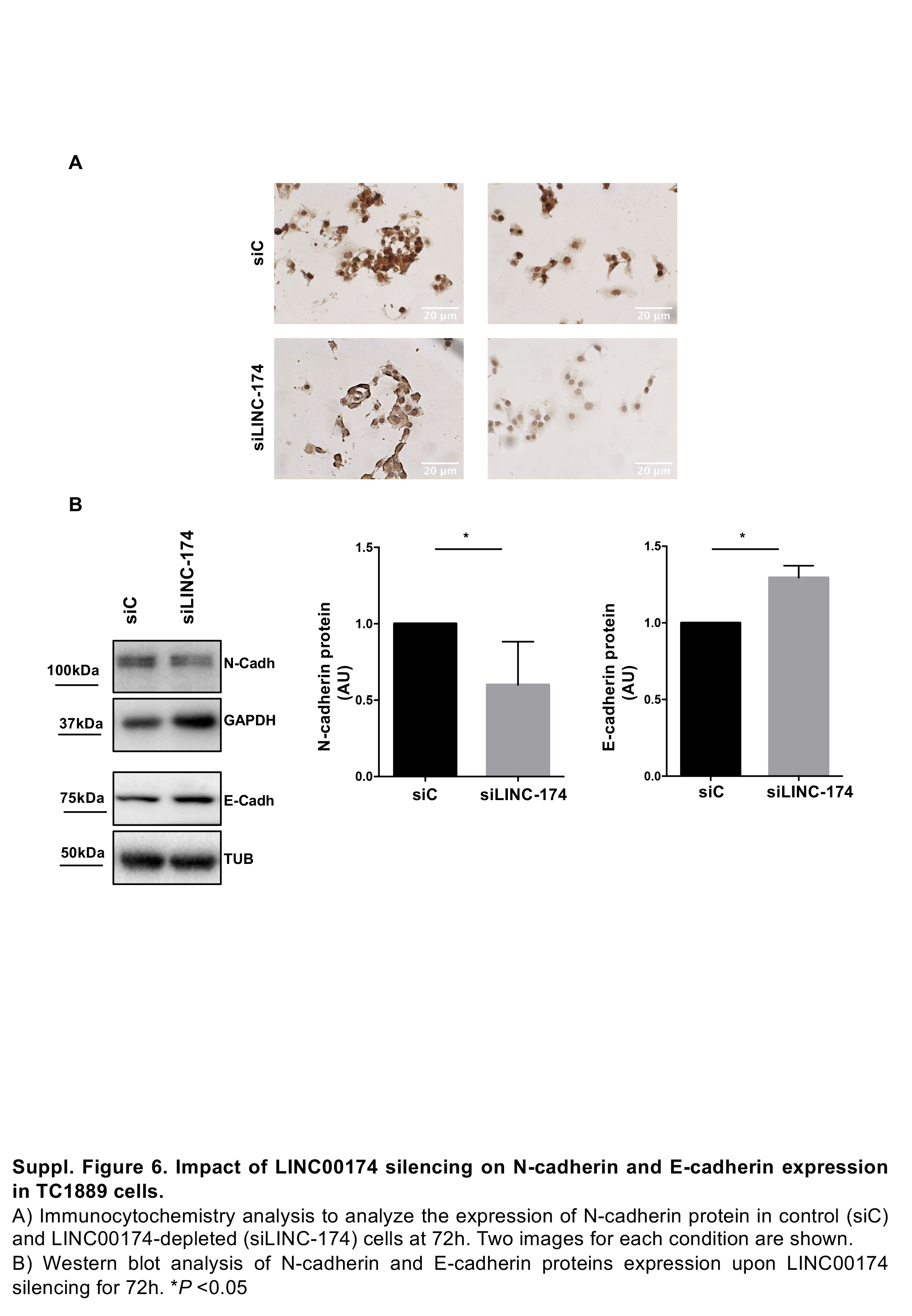

Supplement: Supplementary file 7 — Supplementary Figure 6 [file 41419_2020_3171_MOESM7_ESM.png]

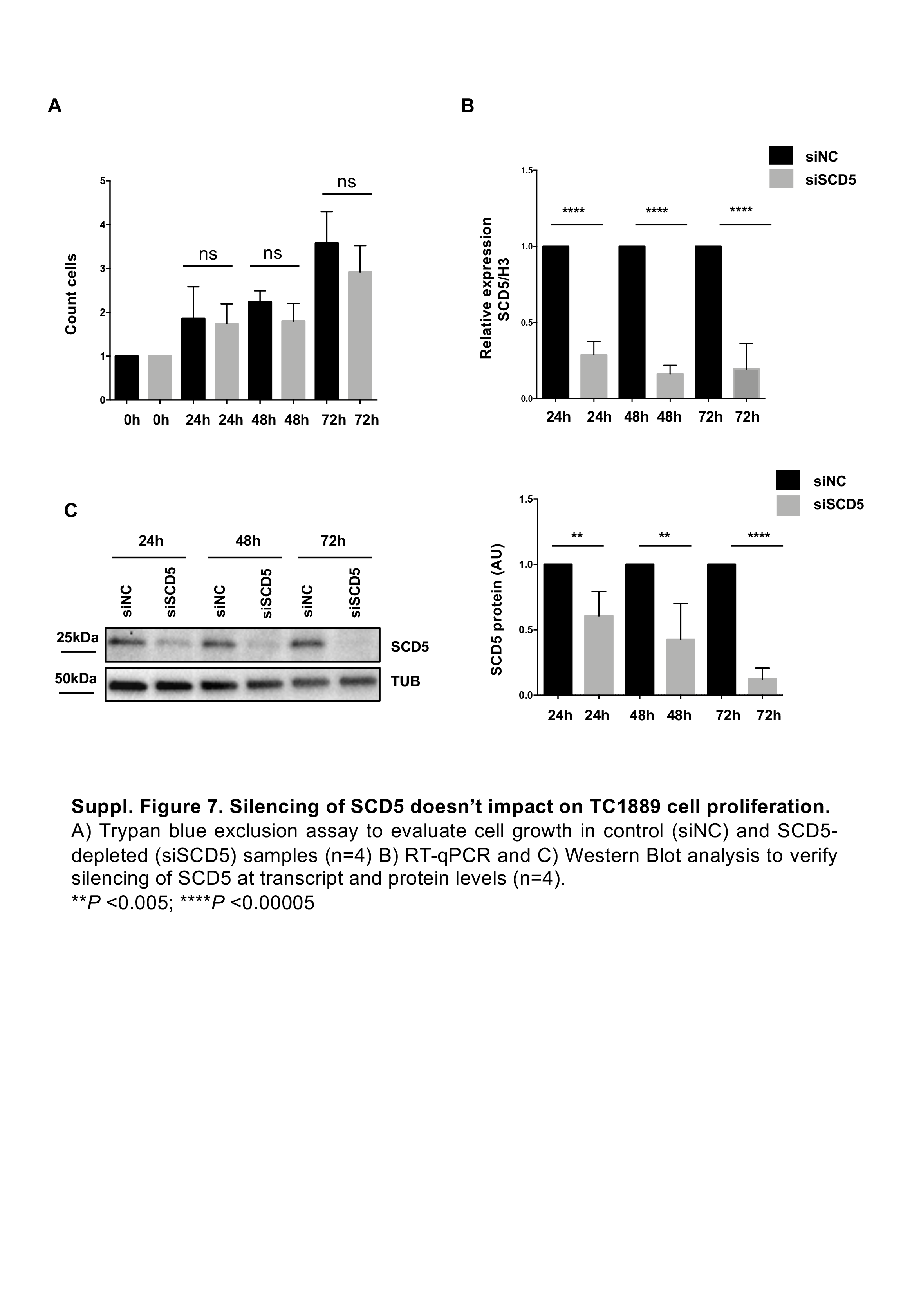

Supplement: Supplementary file 8 — Supplementary Figure 7 [file 41419_2020_3171_MOESM8_ESM.png]

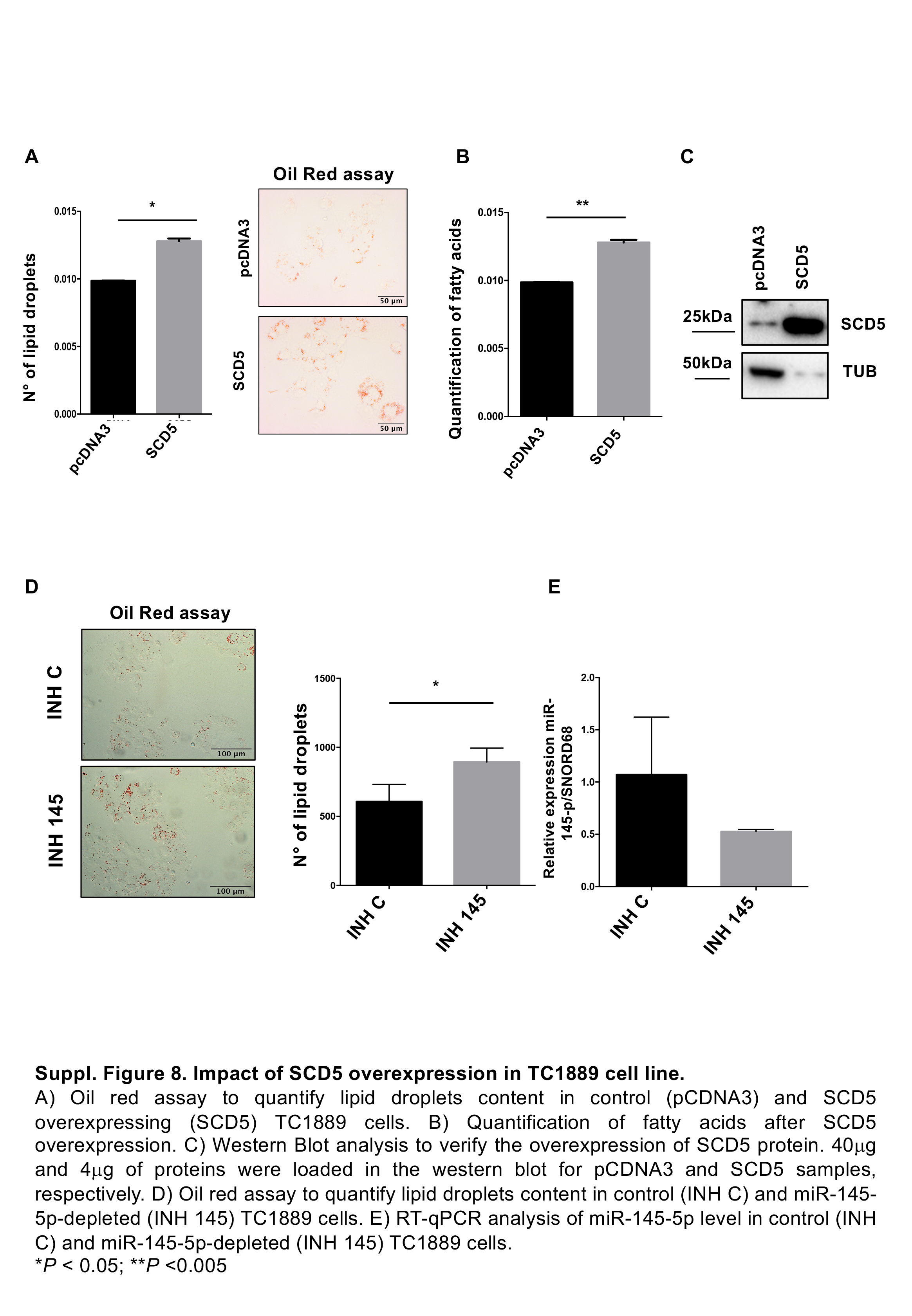

Supplement: Supplementary file 9 — Supplementary Figure 8 [file 41419_2020_3171_MOESM9_ESM.png]

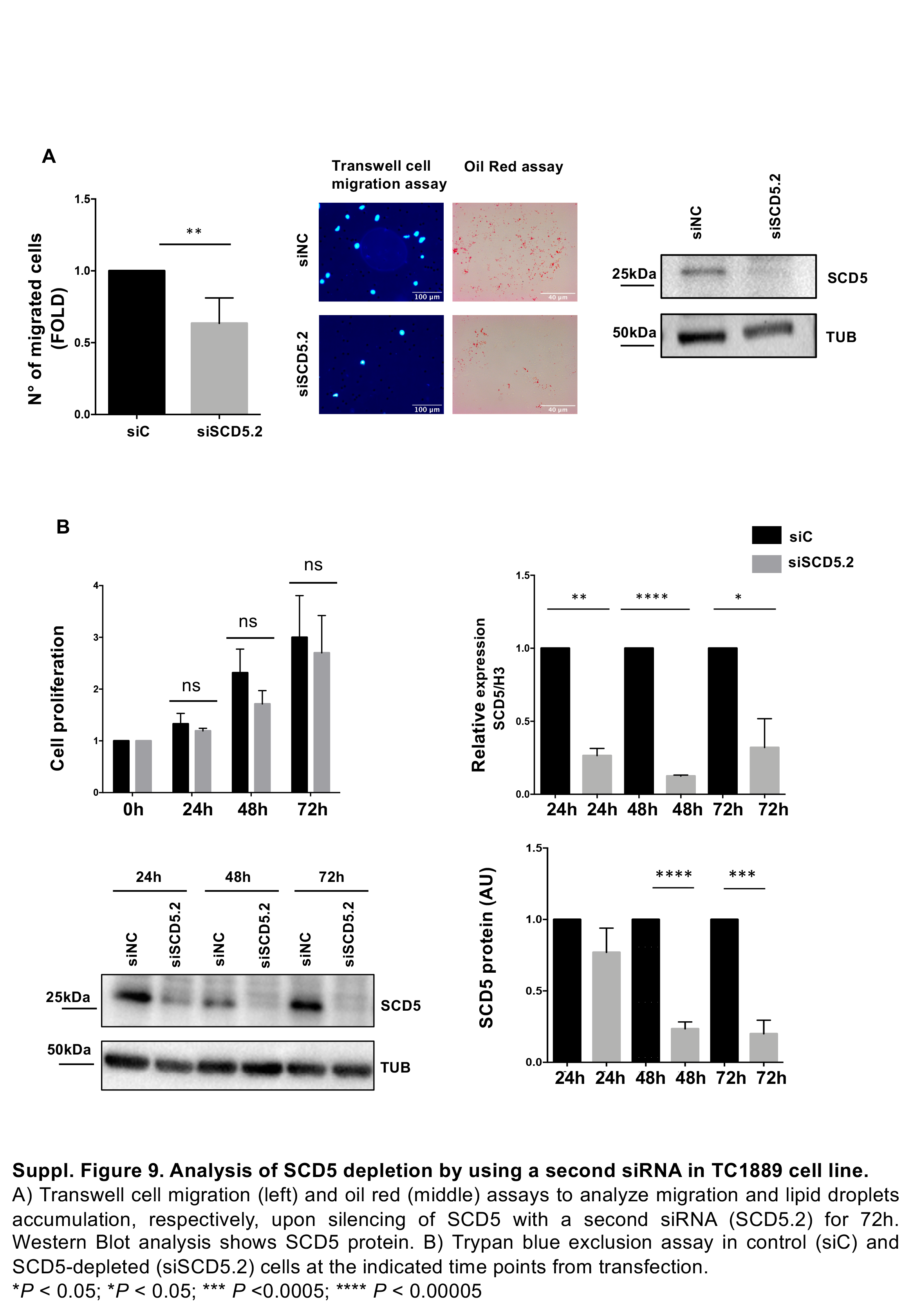

Supplement: Supplementary file 10 — Supplementary Figure 9 [file 41419_2020_3171_MOESM10_ESM.png]

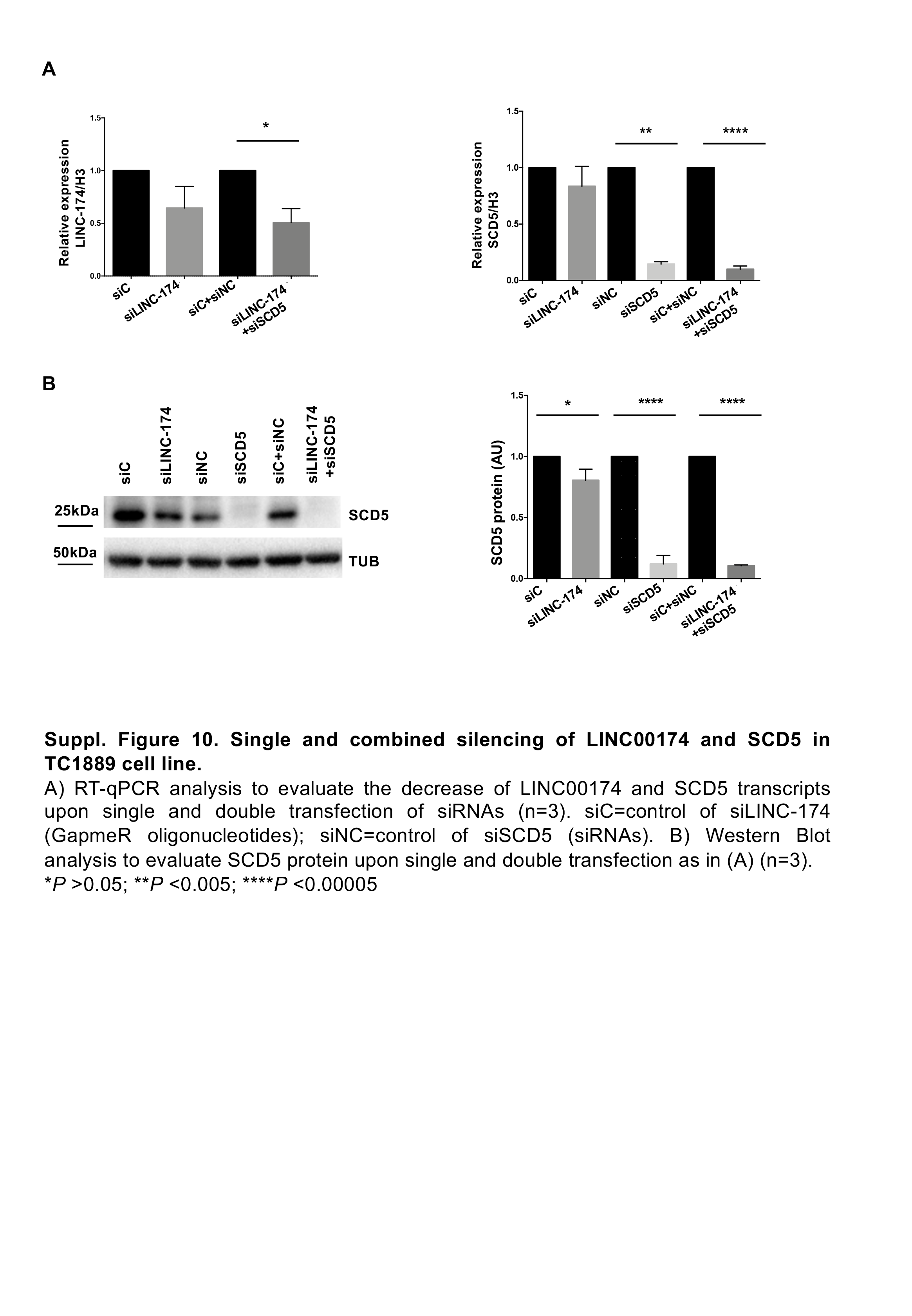

Supplement: Supplementary file 11 — Supplementary Figure 10 [file 41419_2020_3171_MOESM11_ESM.png]

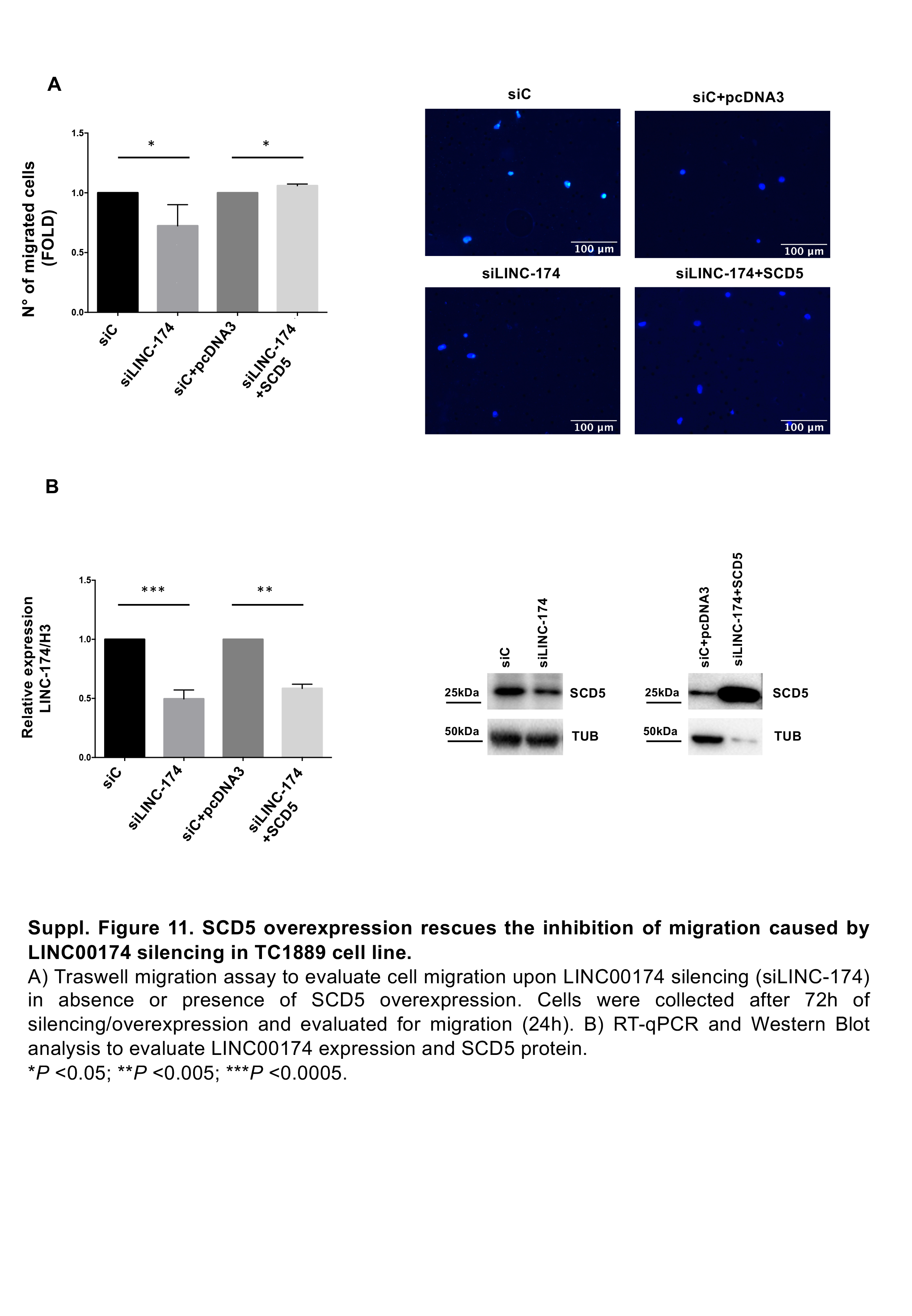

Supplement: Supplementary file 12 — Supplementary Figure 11 [file 41419_2020_3171_MOESM12_ESM.png]
