# Supplementary material for: LINC00174 is a novel prognostic factor in thymic epithelial tumors involved in cell migration and lipid metabolism
Source: Cell Death Dis. 2020 Nov 7;11(11):959. doi: 10.1038/s41419-020-03171-9 (PMC7648846; doi:10.1038/s41419-020-03171-9)
Supplement: Supplementary file 13 — Supplementary Table 1 [file 41419_2020_3171_MOESM13_ESM.pdf]

lncRNAs deregulated in 6 Tumor versus 3 Normal tissues of patients affected by Thymoma from IRE cohort (See Bellissimo et al, 2017 for the description of the cohort)

Up-regulated T vs N			
**lncRNA**	**permutation test**	**t-test**	**log2fold**
OTTHUMG00000164547	0,02313772	0,031191	2,713387
OTTHUMG00000172467	0,027556809	0,034686	2,61711
LINC00491	0,0084816	0,013202	2,172396
OTTHUMG00000015101	0,033507042	0,045558	1,686779
OTTHUMG00000020335	0,017898947	0,017911	1,407693
OTTHUMG00000172035	0,030045759	0,037708	1,403797
OTTHUMG00000166987	0,013295872	0,017352	1,369417
LINC00263	0,011503806	0,012383	1,263002
OTTHUMG00000162559	0,028245328	0,03384	1,237226
LINC00669	0,022795172	0,024541	1,106992
OTTHUMG00000164644	0,011696436	0,011328	1,051994
OTTHUMG00000018883	0,004114105	0,004275	1,023734
OTTHUMG00000022365	0,025730882	0,035119	0,893605
OTTHUMG00000013379	0,012148774	0,012591	0,891369
OTTHUMG00000182863	0,01976628	0,023868	0,877445
OTTHUMG00000169048	0,011134809	0,01489	0,857132
OTTHUMG00000154935	0,025404008	0,033315	0,832516
OTTHUMG00000170462	0,00890299	0,031358	0,813676
OTTHUMG00000168228	0,045810062	0,046167	0,804428
LINC00889	0,049434204	0,049511	0,798252
OTTHUMG00000154733	0,025144026	0,03074	0,789618
OTTHUMG00000160994	0,033000812	0,034643	0,739351
OTTHUMG00000021104	0,010508185	0,010957	0,71144
MGC16142	0,024991602	0,037253	0,692336
OTTHUMG00000013230	0,020922473	0,023696	0,689487
LINC00515	0,005567852	0,004522	0,675677
OTTHUMG00000182110	0,026072422	0,027559	0,669527
OTTHUMG00000179318	0,013310345	0,015859	0,628129
OTTHUMG00000165905	0,036819823	0,041337	0,613825
LINC00174	0,005759511	0,005446	0,605546
LINC00630	0,0205166	0,02086	0,51887
OTTHUMG00000017165	0,011195295	0,012355	0,489944
OTTHUMG00000170253	0,038748425	0,043156	0,482376
OTTHUMG00000155365	0,005324224	0,004559	0,475502
OTTHUMG00000041437	0,021663386	0,02335	0,473984
OTTHUMG00000170339	0,025944056	0,029217	0,459455
OTTHUMG00000166985	0,032485895	0,033413	0,420852
OTTHUMG00000167225	0,012762165	0,027148	0,403681
OTTHUMG00000168693	0,001297991	0,001676	0,400133
OTTHUMG00000151447	0,018006112	0,018105	0,395594
OTTHUMG00000151405	0,046323474	0,046633	0,377753
OTTHUMG00000175754	0,030040067	0,032865	0,363258
OTTHUMG00000155315	0,030487087	0,033869	0,357845
OTTHUMG00000163333	0,022079719	0,023704	0,332795
OTTHUMG00000162408	0,046199594	0,049006	0,329568
OTTHUMG00000178589	0,022212034	0,022726	0,329017
OTTHUMG00000151845	0,001102662	0,002156	0,320839
OTTHUMG00000151374	0,003622961	0,003623	0,310256
OTTHUMG00000154724	0,020654609	0,020639	0,307492
LINC00894	0,006205996	0,005156	0,302026
OTTHUMG00000164289	0,017892184	0,023365	0,292332
OTTHUMG00000163069	0,049412783	0,049657	0,290011
LINC00882	0,020361496	0,02311	0,265031
OTTHUMG00000153617	0,022928439	0,028456	0,261646
LINC00449	0,012731023	0,012323	0,260442
OTTHUMG00000152702	0,019073999	0,019612	0,253236
OTTHUMG00000015931	0,034222618	0,042027	0,235699
OTTHUMG00000017561	0,045666564	0,047925	0,23528
OTTHUMG00000154585	0,017676519	0,021251	0,224579
OTTHUMG00000161703	0,025411455	0,025985	0,210037
OTTHUMG00000163132	0,027328582	0,04429	0,188929
OTTHUMG00000168468	0,006531837	0,005646	0,174071
OTTHUMG00000160705	0,041188437	0,041823	0,163973
LINC00391	0,049110532	0,049507	0,163118
OTTHUMG00000159007	0,043185309	0,045339	0,153861
OTTHUMG00000017875	0,03106156	0,035526	0,146985
LINC00489	0,028545024	0,033118	0,107137
OTTHUMG00000161221	0,02122568	0,022798	0,101003
OTTHUMG00000162625	0,003520005	0,002876	0,080823
OTTHUMG00000032884	0,037115403	0,037975	0,051662

Down-regulated T vs N			
**lncRNA**	**permutation test**	**t-test**	**log2fold**
OTTHUMG00000154833	0,001647328	0,031859	-2,93332
LINC00312	0,006345716	0,030297	-1,91666
OTTHUMG00000164793	0,004328859	0,006806	-1,43703
OTTHUMG00000154838	0,023947598	0,033988	-1,42867
OTTHUMG00000012183	0,00219664	0,001712	-0,91373
LINC00926	0,002635721	0,00303	-0,75968
OTTHUMG00000171168	0,003763251	0,003222	-0,74685
OTTHUMG00000171168	0,003763251	0,003222	-0,74685
FLJ25363	0,005987977	0,005502	-0,7283
OTTHUMG00000184009	0,007096142	0,006544	-0,72104
OTTHUMG00000162005	0,004554282	0,003499	-0,60007
LINC00641	0,004771766	0,004904	-0,54932
OTTHUMG00000163048	0,018899739	0,030108	-0,52663
OTTHUMG00000017960	0,003510357	0,032539	-0,49027
OTTHUMG00000022248	0,004440458	0,005588	-0,48554
OTTHUMG00000152374	0,046200357	0,047689	-0,48294
OTTHUMG00000015353	0,001880106	0,002883	-0,46331
LINC00957	0,004951003	0,004996	-0,46291
LINC00310	0,003979918	0,003933	-0,45145
OTTHUMG00000165726	0,015094641	0,046773	-0,44941
LINC00189	0,001193605	0,001039	-0,41506
OTTHUMG00000151365	0,005397928	0,008206	-0,41048
OTTHUMG00000163006	0,00439967	0,004966	-0,3857
OTTHUMG00000169910	0,009968998	0,03436	-0,35978
OTTHUMG00000163345	0,002397299	0,002177	-0,35661
OTTHUMG00000034834	0,019933404	0,024664	-0,35637
OTTHUMG00000161869	0,013375599	0,027172	-0,35624
OTTHUMG00000162000	0,025129717	0,032229	-0,35147
OTTHUMG00000163630	0,008489053	0,019717	-0,33693
LINC00582	0,094923377	0,109095	-0,33369
OTTHUMG00000153047	0,01575003	0,01512	-0,29866
LINC00324	0,038554356	0,042943	-0,29643
OTTHUMG00000017965	0,009221169	0,008227	-0,29173
LINC00269	0,007081687	0,037883	-0,291
OTTHUMG00000171842	0,022853313	0,036461	-0,28655
OTTHUMG00000151543	0,031506434	0,044299	-0,28255
OTTHUMG00000161608	0,029742731	0,043485	-0,27378
OTTHUMG00000172383	0,008824392	0,007781	-0,2694
OTTHUMG00000008401	0,0205941	0,034281	-0,26558
OTTHUMG00000171853	0,002490998	0,002961	-0,26273
OTTHUMG00000177663	0,003059821	0,00227	-0,254
OTTHUMG00000168051	0,022142161	0,022848	-0,24495
OTTHUMG00000151805	0,020368027	0,049928	-0,24435
OTTHUMG00000086913	0,028530346	0,031394	-0,244
OTTHUMG00000017587	0,027338685	0,027866	-0,23812
OTTHUMG00000066367	0,024582969	0,045783	-0,23665
OTTHUMG00000078329	0,04071423	0,044547	-0,23545
OTTHUMG00000018322	0,015503393	0,014772	-0,23323
OTTHUMG00000160728	0,021831764	0,024504	-0,23255
LINC00508	0,008327852	0,007441	-0,23172
OTTHUMG00000152138	0,028447622	0,03954	-0,22635
OTTHUMG00000158359	0,00043007	0,001314	-0,22493
LINC00473	0,006761314	0,005638	-0,22419
OTTHUMG00000171195	0,038944073	0,04763	-0,21283
OTTHUMG00000163675	0,015599442	0,027199	-0,21212
OTTHUMG00000150946	0,036325637	0,040951	-0,2047
OTTHUMG00000168914	0,021285571	0,033093	-0,20251
OTTHUMG00000152023	0,038613919	0,043996	-0,20218
OTTHUMG00000163136	0,005704639	0,006081	-0,19073
OTTHUMG00000162339	0,018372199	0,022399	-0,1865
OTTHUMG00000154343	0,003267778	0,008982	-0,18559
OTTHUMG00000014989	0,040958721	0,046709	-0,18393
OTTHUMG00000004188	0,02250644	0,028749	-0,1818
OTTHUMG00000037511	0,038273316	0,03913	-0,1773
OTTHUMG00000037823	0,020935398	0,025862	-0,17611
LINC00517	0,021033693	0,024932	-0,17509
LINC00568	0,004701511	0,007222	-0,17389
OTTHUMG00000168958	0,040570755	0,041807	-0,17083
OTTHUMG00000162167	0,0234486	0,027667	-0,17047
OTTHUMG00000154465	0,031918582	0,0341	-0,16224
LINC00910	0,03319576	0,040917	-0,15149
OTTHUMG00000162998	0,010006315	0,010153	-0,14859
LINC00173	0,455432445	0,425941	-0,14328
OTTHUMG00000168706	0,036256723	0,037364	-0,14108
OTTHUMG00000020779	0,013122677	0,013913	-0,13945
OTTHUMG00000151627	0,012688516	0,011837	-0,13403
OTTHUMG00000151420	0,024299527	0,030711	-0,13355
OTTHUMG00000020459	0,01793237	0,017729	-0,13191
OTTHUMG00000171690	0,007412684	0,008215	-0,12787
OTTHUMG00000168454	0,045050588	0,046862	-0,12686
OTTHUMG00000177424	0,046534367	0,048233	-0,11062
OTTHUMG00000160932	0,007357656	0,029884	-0,10221
OTTHUMG00000170013	0,021837501	0,037759	-0,03977
OTTHUMG00000180596	0,933444262	0,927934	-0,0075
