# Supplementary material for: LINC00174 is a novel prognostic factor in thymic epithelial tumors involved in cell migration and lipid metabolism
Source: Cell Death Dis. 2020 Nov 7;11(11):959. doi: 10.1038/s41419-020-03171-9 (PMC7648846; doi:10.1038/s41419-020-03171-9)
Supplement: Supplementary file 14 — Supplementary Table 2 [file 41419_2020_3171_MOESM14_ESM.pdf]

Genes and lncRNAs positively correlated in IRE cohort and predicted to be target of miR145-5p by miRwalk 2.0 tool				
**lncRNA**	**gene(UP)**	**lncRNA\gene**	**R Spearman**	**pval**
C17orf102	AFG3L2	C17orf102\AFG3L2	0,74999994	0,025490521
C17orf102	ANKFY1	C17orf102\ANKFY1	0,883333266	0,003075397
C17orf102	ANO9	C17orf102\ANO9	0,883333266	0,003075397
C17orf102	AP2B1	C17orf102\AP2B1	0,783333302	0,017223325
C17orf102	APPBP2	C17orf102\APPBP2	0,866666615	0,004508378
C17orf102	ARL6IP1	C17orf102\ARL6IP1	0,699999928	0,043253969
C17orf102	ATHL1	C17orf102\ATHL1	0,699999928	0,043253969
C17orf102	ATP2C1	C17orf102\ATP2C1	0,699999928	0,043253969
C17orf102	ATP6V1A	C17orf102\ATP6V1A	0,783333302	0,017223325
C17orf102	ATP6V1C1	C17orf102\ATP6V1C1	0,73333329	0,031123236
C17orf102	ATRNL1	C17orf102\ATRNL1	0,849999964	0,006073633
C17orf102	BBS4	C17orf102\BBS4	0,716666639	0,036866181
C17orf102	C5orf15	C17orf102\C5orf15	0,783333302	0,017223325
C17orf102	CALML3	C17orf102\CALML3	0,833333313	0,008267196
C17orf102	CALR	C17orf102\CALR	0,699999928	0,043253969
C17orf102	CAMKK1	C17orf102\CAMKK1	0,849999964	0,006073633
C17orf102	CAMSAP1	C17orf102\CAMSAP1	0,833333313	0,008267196
C17orf102	CCL25	C17orf102\CCL25	0,866666615	0,004508378
C17orf102	CD274	C17orf102\CD274	0,783333302	0,017223325
C17orf102	CDKL5	C17orf102\CDKL5	0,766666651	0,021389991
C17orf102	CDS1	C17orf102\CDS1	0,73333329	0,031123236
C17orf102	CLINT1	C17orf102\CLINT1	0,699999928	0,043253969
C17orf102	CLTC	C17orf102\CLTC	0,799999952	0,013828263
C17orf102	CNTNAP3	C17orf102\CNTNAP3	0,866666615	0,004508378
C17orf102	CNTNAP3B	C17orf102\CNTNAP3B	0,699999928	0,043253969
C17orf102	CROT	C17orf102\CROT	0,716666639	0,036866181
C17orf102	CUX1	C17orf102\CUX1	0,766666651	0,021389991
C17orf102	CYP26B1	C17orf102\CYP26B1	0,916666567	0,001311728
C17orf102	DACT1	C17orf102\DACT1	0,783333302	0,017223325
C17orf102	DMRT2	C17orf102\DMRT2	0,699999928	0,043253969
C17orf102	DTNB	C17orf102\DTNB	0,73333329	0,031123236
C17orf102	EIF2AK1	C17orf102\EIF2AK1	0,866666615	0,004508378

C17orf102	ELOVL6	C17orf102\ELOVL6	0,816666603	0,010769401
C17orf102	ERAP1	C17orf102\ERAP1	0,74999994	0,025490521
C17orf102	ESRP1	C17orf102\ESRP1	0,699999928	0,043253969
C17orf102	ESRP2	C17orf102\ESRP2	0,73333329	0,031123236
C17orf102	FAM120A	C17orf102\FAM120A	0,716666639	0,036866181
C17orf102	FAM163A	C17orf102\FAM163A	0,783333302	0,017223325
C17orf102	FAM199X	C17orf102\FAM199X	0,74999994	0,025490521
C17orf102	FBXO2	C17orf102\FBXO2	0,811722577	0,010769401
C17orf102	FBXO22	C17orf102\FBXO22	0,711303294	0,037125219
C17orf102	FLVCR1	C17orf102\FLVCR1	0,699999928	0,043253969
C17orf102	FSTL4	C17orf102\FSTL4	0,833333313	0,008267196
C17orf102	GAA	C17orf102\GAA	0,816666603	0,010769401
C17orf102	GGA2	C17orf102\GGA2	0,783333302	0,017223325
C17orf102	GINS3	C17orf102\GINS3	0,799999952	0,013828263
C17orf102	GLI2	C17orf102\GLI2	0,74999994	0,025490521
C17orf102	GPHN	C17orf102\GPHN	0,816666603	0,010769401
C17orf102	GPR64	C17orf102\GPR64	0,699999928	0,043253969
C17orf102	GPS1	C17orf102\GPS1	0,866666615	0,004508378
C17orf102	HDAC4	C17orf102\HDAC4	0,853563964	0,005202822
C17orf102	IQCH	C17orf102\IQCH	0,833333313	0,008267196
C17orf102	KDM4B	C17orf102\KDM4B	0,699999928	0,043253969
C17orf102	KDM4D	C17orf102\KDM4D	0,719671547	0,033763226
C17orf102	KIAA1549	C17orf102\KIAA1549	0,73333329	0,031123236
C17orf102	LAMP2	C17orf102\LAMP2	0,836827397	0,00696649
C17orf102	LGI3	C17orf102\LGI3	0,74999994	0,025490521
C17orf102	LMBR1	C17orf102\LMBR1	0,833333313	0,008267196
C17orf102	LMOD3	C17orf102\LMOD3	0,762821436	0,022123016
C17orf102	M1AP	C17orf102\M1AP	0,699999928	0,043253969
C17orf102	MAST4	C17orf102\MAST4	0,70293504	0,040145501
C17orf102	MED21	C17orf102\MED21	0,769881189	0,018992504
C17orf102	METTL8	C17orf102\METTL8	0,816666603	0,010769401
C17orf102	MFSD3	C17orf102\MFSD3	0,799999952	0,013828263
C17orf102	MPP5	C17orf102\MPP5	0,73333329	0,031123236
C17orf102	MTPN	C17orf102\MTPN	0,845195651	0,006205908

C17orf102	NCS1	C17orf102\NCS1	0,699999928	0,043253969
C17orf102	NDUFA4	C17orf102\NDUFA4	0,716666639	0,036866181
C17orf102	NDUFB5	C17orf102\NDUFB5	0,716666639	0,036866181
C17orf102	NETO2	C17orf102\NETO2	0,699999928	0,043253969
C17orf102	NIF3L1	C17orf102\NIF3L1	0,849999964	0,006073633
C17orf102	NISCH	C17orf102\NISCH	0,716666639	0,036866181
C17orf102	NPTX1	C17orf102\NPTX1	0,74999994	0,025490521
C17orf102	NVL	C17orf102\NVL	0,716666639	0,036866181
C17orf102	OXCT1	C17orf102\OXCT1	0,833333313	0,008267196
C17orf102	PASK	C17orf102\PASK	0,849999964	0,006073633
C17orf102	PCCB	C17orf102\PCCB	0,716666639	0,036866181
C17orf102	PCTP	C17orf102\PCTP	0,870300472	0,003681658
C17orf102	PHLPP1	C17orf102\PHLPP1	0,866666615	0,004508378
C17orf102	PIFO	C17orf102\PIFO	0,716666639	0,036866181
C17orf102	PKP1	C17orf102\PKP1	0,766666651	0,021389991
C17orf102	PLCXD1	C17orf102\PLCXD1	0,73333329	0,031123236
C17orf102	PLD2	C17orf102\PLD2	0,699999928	0,043253969
C17orf102	PLEK2	C17orf102\PLEK2	0,899999917	0,002028219
C17orf102	PPIP5K1	C17orf102\PPIP5K1	0,799999952	0,013828263
C17orf102	PRSS8	C17orf102\PRSS8	0,699999928	0,043253969
C17orf102	PSMC2	C17orf102\PSMC2	0,816666603	0,010769401
C17orf102	PSMD12	C17orf102\PSMD12	0,716666639	0,036866181
C17orf102	PTPRT	C17orf102\PTPRT	0,833333313	0,008267196
C17orf102	RAB39A	C17orf102\RAB39A	0,73333329	0,031123236
C17orf102	RCC2	C17orf102\RCC2	0,699999928	0,043253969
C17orf102	RFESD	C17orf102\RFESD	0,816666603	0,010769401
C17orf102	RRAGD	C17orf102\RRAGD	0,833333313	0,008267196
C17orf102	S100A14	C17orf102\S100A14	0,849999964	0,006073633
C17orf102	SEC61A1	C17orf102\SEC61A1	0,699999928	0,043253969
C17orf102	SFXN5	C17orf102\SFXN5	0,853563964	0,005202822
C17orf102	SGPP2	C17orf102\SGPP2	0,799999952	0,013828263
C17orf102	SHROOM2	C17orf102\SHROOM2	0,766666651	0,021389991
C17orf102	SLC22A5	C17orf102\SLC22A5	0,73333329	0,031123236
C17orf102	SLCO5A1	C17orf102\SLCO5A1	0,866666615	0,004508378

C17orf102	SNX1	C17orf102\SNX1	0,883333266	0,003075397
C17orf102	SOGA2	C17orf102\SOGA2	0,73333329	0,031123236
C17orf102	SORCS2	C17orf102\SORCS2	0,716666639	0,036866181
C17orf102	SPRY3	C17orf102\SPRY3	0,699999928	0,043253969
C17orf102	STX6	C17orf102\STX6	0,866666615	0,004508378
C17orf102	SUSD4	C17orf102\SUSD4	0,883333266	0,003075397
C17orf102	SYBU	C17orf102\SYBU	0,766666651	0,021389991
C17orf102	TBC1D2B	C17orf102\TBC1D2B	0,766666651	0,021389991
C17orf102	TCEB3	C17orf102\TCEB3	0,783333302	0,017223325
C17orf102	TDGF1	C17orf102\TDGF1	0,73333329	0,031123236
C17orf102	THSD7B	C17orf102\THSD7B	0,783333302	0,017223325
C17orf102	TM7SF3	C17orf102\TM7SF3	0,73333329	0,031123236
C17orf102	TMEM129	C17orf102\TMEM129	0,728039861	0,031283069
C17orf102	TRIM52	C17orf102\TRIM52	0,766666651	0,021389991
C17orf102	TRPM7	C17orf102\TRPM7	0,766666651	0,021389991
C17orf102	TYK2	C17orf102\TYK2	0,716666639	0,036866181
C17orf102	UBAC1	C17orf102\UBAC1	0,716666639	0,036866181
C17orf102	UBFD1	C17orf102\UBFD1	0,716666639	0,036866181
C17orf102	UNG	C17orf102\UNG	0,849999964	0,006073633
C17orf102	USP40	C17orf102\USP40	0,74999994	0,025490521
C17orf102	WDR91	C17orf102\WDR91	0,799999952	0,013828263
C17orf102	WWOX	C17orf102\WWOX	0,719671547	0,033763226
C17orf102	XYLT2	C17orf102\XYLT2	0,833333313	0,008267196
C17orf102	ZNF132	C17orf102\ZNF132	0,699999928	0,043253969
C17orf102	ZNF554	C17orf102\ZNF554	0,711966693	0,038029101
CALML3-AS1	AJUBA	CALML3-AS1\AJUBA	0,916666567	0,001311728
CALML3-AS1	AKR1B1	CALML3-AS1\AKR1B1	0,74999994	0,025490521
CALML3-AS1	ALDH7A1	CALML3-AS1\ALDH7A1	0,833333313	0,008267196
CALML3-AS1	ALS2	CALML3-AS1\ALS2	0,74999994	0,025490521
CALML3-AS1	ANK3	CALML3-AS1\ANK3	0,799999952	0,013828263
CALML3-AS1	AP1S1	CALML3-AS1\AP1S1	0,849999964	0,006073633
CALML3-AS1	APOBEC2	CALML3-AS1\APOBEC2	0,699999928	0,043253969
CALML3-AS1	APP	CALML3-AS1\APP	0,849999964	0,006073633
CALML3-AS1	ARL1	CALML3-AS1\ARL1	0,816666603	0,010769401

CALML3-AS1	ATP6V0A1	CALML3-AS1\ATP6V0A1	0,915385723	0,001190476
CALML3-AS1	ATP6V0D1	CALML3-AS1\ATP6V0D1	0,783333302	0,017223325
CALML3-AS1	ATRNL1	CALML3-AS1\ATRNL1	0,716666639	0,036866181
CALML3-AS1	AUTS2	CALML3-AS1\AUTS2	0,916666567	0,001311728
CALML3-AS1	AZIN1	CALML3-AS1\AZIN1	0,816666603	0,010769401
CALML3-AS1	BAIAP2	CALML3-AS1\BAIAP2	0,883333266	0,003075397
CALML3-AS1	BCL11A	CALML3-AS1\BCL11A	0,716666639	0,036866181
CALML3-AS1	BRD1	CALML3-AS1\BRD1	0,816666603	0,010769401
CALML3-AS1	C14orf39	CALML3-AS1\C14orf39	0,849999964	0,006073633
CALML3-AS1	C19orf82	CALML3-AS1\C19orf82	0,766666651	0,021389991
CALML3-AS1	C4A	CALML3-AS1\C4A	0,833333313	0,008267196
CALML3-AS1	C4B	CALML3-AS1\C4B	0,833333313	0,008267196
CALML3-AS1	C5orf15	CALML3-AS1\C5orf15	0,699999928	0,043253969
CALML3-AS1	C5orf24	CALML3-AS1\C5orf24	0,716666639	0,036866181
CALML3-AS1	CACUL1	CALML3-AS1\CACUL1	0,766666651	0,021389991
CALML3-AS1	CALR	CALML3-AS1\CALR	0,766666651	0,021389991
CALML3-AS1	CCDC122	CALML3-AS1\CCDC122	0,916666567	0,001311728
CALML3-AS1	CCDC148	CALML3-AS1\CCDC148	0,816666603	0,010769401
CALML3-AS1	CCDC30	CALML3-AS1\CCDC30	0,866666615	0,004508378
CALML3-AS1	CCDC73	CALML3-AS1\CCDC73	0,878668785	0,003218695
CALML3-AS1	CCDC8	CALML3-AS1\CCDC8	0,883333266	0,003075397
CALML3-AS1	CDC42BPG	CALML3-AS1\CDC42BPG	0,79498601	0,013833774
CALML3-AS1	CDIP1	CALML3-AS1\CDIP1	0,799999952	0,013828263
CALML3-AS1	CDKL3	CALML3-AS1\CDKL3	0,699999928	0,043253969
CALML3-AS1	CDKL5	CALML3-AS1\CDKL5	0,74999994	0,025490521
CALML3-AS1	CDS1	CALML3-AS1\CDS1	0,74999994	0,025490521
CALML3-AS1	CETN3	CALML3-AS1\CETN3	0,716666639	0,036866181
CALML3-AS1	CHID1	CALML3-AS1\CHID1	0,899999917	0,002028219
CALML3-AS1	CLCA1	CALML3-AS1\CLCA1	0,922611535	0,000793651
CALML3-AS1	CLCN5	CALML3-AS1\CLCN5	0,73333329	0,031123236
CALML3-AS1	CLDN12	CALML3-AS1\CLDN12	0,716666639	0,036866181
CALML3-AS1	CLSTN1	CALML3-AS1\CLSTN1	0,933333278	0,000749559
CALML3-AS1	CNTNAP2	CALML3-AS1\CNTNAP2	0,783333302	0,017223325
CALML3-AS1	CNTNAP3	CALML3-AS1\CNTNAP3	0,73333329	0,031123236

CALML3-AS1	CNTNAP3B	CALML3-AS1\CNTNAP3B	0,73333329	0,031123236
CALML3-AS1	COL8A2	CALML3-AS1\COL8A2	0,916666567	0,001311728
CALML3-AS1	COX19	CALML3-AS1\COX19	0,933333278	0,000749559
CALML3-AS1	CRTC1	CALML3-AS1\CRTC1	0,916666567	0,001311728
CALML3-AS1	DACT1	CALML3-AS1\DACT1	0,74999994	0,025490521
CALML3-AS1	DCAKD	CALML3-AS1\DCAKD	0,799999952	0,013828263
CALML3-AS1	DDX31	CALML3-AS1\DDX31	0,816666603	0,010769401
CALML3-AS1	DET1	CALML3-AS1\DET1	0,899999917	0,002028219
CALML3-AS1	DHX32	CALML3-AS1\DHX32	0,816666603	0,010769401
CALML3-AS1	DMRT2	CALML3-AS1\DMRT2	0,73333329	0,031123236
CALML3-AS1	DNAJC16	CALML3-AS1\DNAJC16	0,716666639	0,036866181
CALML3-AS1	DNAJC19	CALML3-AS1\DNAJC19	0,883333266	0,003075397
CALML3-AS1	DNAJC21	CALML3-AS1\DNAJC21	0,899999917	0,002028219
CALML3-AS1	DNAL4	CALML3-AS1\DNAL4	0,74999994	0,025490521
CALML3-AS1	DPH6	CALML3-AS1\DPH6	0,73333329	0,031123236
CALML3-AS1	DSG2	CALML3-AS1\DSG2	0,866666615	0,004508378
CALML3-AS1	DUOXA1	CALML3-AS1\DUOXA1	0,916666567	0,001311728
CALML3-AS1	EDA2R	CALML3-AS1\EDA2R	0,916666567	0,001311728
CALML3-AS1	EFNB2	CALML3-AS1\EFNB2	0,883333266	0,003075397
CALML3-AS1	EIF2AK4	CALML3-AS1\EIF2AK4	0,766666651	0,021389991
CALML3-AS1	EMC1	CALML3-AS1\EMC1	0,73333329	0,031123236
CALML3-AS1	EMC10	CALML3-AS1\EMC10	0,945614994	0,00037478
CALML3-AS1	ENOSF1	CALML3-AS1\ENOSF1	0,73333329	0,031123236
CALML3-AS1	EPN3	CALML3-AS1\EPN3	0,799999952	0,013828263
CALML3-AS1	EPS15L1	CALML3-AS1\EPS15L1	0,716666639	0,036866181
CALML3-AS1	ESRP1	CALML3-AS1\ESRP1	0,766666651	0,021389991
CALML3-AS1	ESRP2	CALML3-AS1\ESRP2	0,799999952	0,013828263
CALML3-AS1	EVA1A	CALML3-AS1\EVA1A	0,833333313	0,008267196
CALML3-AS1	FAHD1	CALML3-AS1\FAHD1	0,73333329	0,031123236
CALML3-AS1	FAM160A1	CALML3-AS1\FAM160A1	0,883333266	0,003075397
CALML3-AS1	FAM218A	CALML3-AS1\FAM218A	0,849999964	0,006073633
CALML3-AS1	FAM83B	CALML3-AS1\FAM83B	0,916666567	0,001311728
CALML3-AS1	FBXW11	CALML3-AS1\FBXW11	0,711966693	0,038029101
CALML3-AS1	FEM1B	CALML3-AS1\FEM1B	0,899999917	0,002028219

CALML3-AS1	FNBP1L	CALML3-AS1\FNBP1L	0,766666651	0,021389991
CALML3-AS1	FRAS1	CALML3-AS1\FRAS1	0,766666651	0,021389991
CALML3-AS1	GGA1	CALML3-AS1\GGA1	0,833333313	0,008267196
CALML3-AS1	GNPDA1	CALML3-AS1\GNPDA1	0,899999917	0,002028219
CALML3-AS1	GOLM1	CALML3-AS1\GOLM1	0,783333302	0,017223325
CALML3-AS1	GPC4	CALML3-AS1\GPC4	0,899999917	0,002028219
CALML3-AS1	GPR107	CALML3-AS1\GPR107	0,830627799	0,007638889
CALML3-AS1	GPR64	CALML3-AS1\GPR64	0,716666639	0,036866181
CALML3-AS1	GRHL2	CALML3-AS1\GRHL2	0,816666603	0,010769401
CALML3-AS1	GRIP1	CALML3-AS1\GRIP1	0,699999928	0,043253969
CALML3-AS1	GRTP1	CALML3-AS1\GRTP1	0,866666615	0,004508378
CALML3-AS1	GTF3C4	CALML3-AS1\GTF3C4	0,766666651	0,021389991
CALML3-AS1	GXYLT1	CALML3-AS1\GXYLT1	0,849999964	0,006073633
CALML3-AS1	HDGFRP3	CALML3-AS1\HDGFRP3	0,833333313	0,008267196
CALML3-AS1	HN1L	CALML3-AS1\HN1L	0,74999994	0,025490521
CALML3-AS1	HOMER3	CALML3-AS1\HOMER3	0,883333266	0,003075397
CALML3-AS1	HPS5	CALML3-AS1\HPS5	0,966666639	0,000165344
CALML3-AS1	IGF1R	CALML3-AS1\IGF1R	0,833333313	0,008267196
CALML3-AS1	IGFBP2	CALML3-AS1\IGFBP2	0,799999952	0,013828263
CALML3-AS1	IGSF3	CALML3-AS1\IGSF3	0,766666651	0,021389991
CALML3-AS1	IKBIP	CALML3-AS1\IKBIP	0,883333266	0,003075397
CALML3-AS1	IRF2BP2	CALML3-AS1\IRF2BP2	0,699999928	0,043253969
CALML3-AS1	ITGA2	CALML3-AS1\ITGA2	0,866666615	0,004508378
CALML3-AS1	ITGAV	CALML3-AS1\ITGAV	0,799999952	0,013828263
CALML3-AS1	KAL1	CALML3-AS1\KAL1	0,833333313	0,008267196
CALML3-AS1	KCTD1	CALML3-AS1\KCTD1	0,883333266	0,003075397
CALML3-AS1	KDM4B	CALML3-AS1\KDM4B	0,699999928	0,043253969
CALML3-AS1	KDM4D	CALML3-AS1\KDM4D	0,686198473	0,046968695
CALML3-AS1	KIAA0319L	CALML3-AS1\KIAA0319L	0,916666567	0,001311728
CALML3-AS1	KRTAP5-8	CALML3-AS1\KRTAP5-8	0,849999964	0,006073633
CALML3-AS1	LACC1	CALML3-AS1\LACC1	0,803354323	0,012202381
CALML3-AS1	LAMP1	CALML3-AS1\LAMP1	0,878668785	0,003218695
CALML3-AS1	LAMP2	CALML3-AS1\LAMP2	0,711303294	0,037125219
CALML3-AS1	LAMP5	CALML3-AS1\LAMP5	0,883333266	0,003075397

CALML3-AS1	LEPREL1	CALML3-AS1\LEPREL1	0,799999952	0,013828263
CALML3-AS1	LGI3	CALML3-AS1\LGI3	0,699999928	0,043253969
CALML3-AS1	LPHN3	CALML3-AS1\LPHN3	0,849999964	0,006073633
CALML3-AS1	LPIN1	CALML3-AS1\LPIN1	0,916666567	0,001311728
CALML3-AS1	LRIG3	CALML3-AS1\LRIG3	0,849999964	0,006073633
CALML3-AS1	LRPAP1	CALML3-AS1\LRPAP1	0,786617756	0,015288801
CALML3-AS1	LRRK1	CALML3-AS1\LRRK1	0,783333302	0,017223325
CALML3-AS1	M1AP	CALML3-AS1\M1AP	0,699999928	0,043253969
CALML3-AS1	MAGEA3	CALML3-AS1\MAGEA3	0,73333329	0,031123236
CALML3-AS1	MARVELD2	CALML3-AS1\MARVELD2	0,766666651	0,021389991
CALML3-AS1	MAST4	CALML3-AS1\MAST4	0,895405293	0,002105379
CALML3-AS1	MCTP2	CALML3-AS1\MCTP2	0,799999952	0,013828263
CALML3-AS1	MED21	CALML3-AS1\MED21	0,686198473	0,046968695
CALML3-AS1	MED22	CALML3-AS1\MED22	0,864530981	0,004596561
CALML3-AS1	MLX	CALML3-AS1\MLX	0,853563964	0,005202822
CALML3-AS1	MOV10	CALML3-AS1\MOV10	0,816666603	0,010769401
CALML3-AS1	MST1L	CALML3-AS1\MST1L	0,783333302	0,017223325
CALML3-AS1	MYLK4	CALML3-AS1\MYLK4	0,833333313	0,008267196
CALML3-AS1	MYO10	CALML3-AS1\MYO10	0,799999952	0,013828263
CALML3-AS1	MYO5B	CALML3-AS1\MYO5B	0,79498601	0,013833774
CALML3-AS1	N4BP1	CALML3-AS1\N4BP1	0,799999952	0,013828263
CALML3-AS1	N4BP2L2	CALML3-AS1\N4BP2L2	0,849999964	0,006073633
CALML3-AS1	NAA25	CALML3-AS1\NAA25	0,73333329	0,031123236
CALML3-AS1	NBPF11	CALML3-AS1\NBPF11	0,716666639	0,036866181
CALML3-AS1	NBPF9	CALML3-AS1\NBPF9	0,716666639	0,036866181
CALML3-AS1	NCS1	CALML3-AS1\NCS1	0,73333329	0,031123236
CALML3-AS1	NDRG3	CALML3-AS1\NDRG3	0,883333266	0,003075397
CALML3-AS1	NDUFA4	CALML3-AS1\NDUFA4	0,73333329	0,031123236
CALML3-AS1	NEO1	CALML3-AS1\NEO1	0,883333266	0,003075397
CALML3-AS1	NET1	CALML3-AS1\NET1	0,866666615	0,004508378
CALML3-AS1	NETO2	CALML3-AS1\NETO2	0,699999928	0,043253969
CALML3-AS1	NHLRC3	CALML3-AS1\NHLRC3	0,916666567	0,001311728
CALML3-AS1	NISCH	CALML3-AS1\NISCH	0,74999994	0,025490521
CALML3-AS1	NPNT	CALML3-AS1\NPNT	0,883333266	0,003075397

CALML3-AS1	NRCAM	CALML3-AS1\NRCAM	0,74999994	0,025490521
CALML3-AS1	NTF4	CALML3-AS1\NTF4	0,766666651	0,021389991
CALML3-AS1	NUDT9	CALML3-AS1\NUDT9	0,849999964	0,006073633
CALML3-AS1	NXN	CALML3-AS1\NXN	0,895405293	0,002105379
CALML3-AS1	OAT	CALML3-AS1\OAT	0,699999928	0,043253969
CALML3-AS1	OSBPL3	CALML3-AS1\OSBPL3	0,799999952	0,013828263
CALML3-AS1	OXCT1	CALML3-AS1\OXCT1	0,699999928	0,043253969
CALML3-AS1	PAX1	CALML3-AS1\PAX1	0,799999952	0,013828263
CALML3-AS1	PAX9	CALML3-AS1\PAX9	0,799999952	0,013828263
CALML3-AS1	PCSK6	CALML3-AS1\PCSK6	0,816666603	0,010769401
CALML3-AS1	PDGFA	CALML3-AS1\PDGFA	0,899999917	0,002028219
CALML3-AS1	PDPK1	CALML3-AS1\PDPK1	0,766666651	0,021389991
CALML3-AS1	PGAP1	CALML3-AS1\PGAP1	0,766666651	0,021389991
CALML3-AS1	PIAS2	CALML3-AS1\PIAS2	0,883333266	0,003075397
CALML3-AS1	PIFO	CALML3-AS1\PIFO	0,883333266	0,003075397
CALML3-AS1	PKP1	CALML3-AS1\PKP1	0,849999964	0,006073633
CALML3-AS1	PKP4	CALML3-AS1\PKP4	0,73333329	0,031123236
CALML3-AS1	PLA2G12A	CALML3-AS1\PLA2G12A	0,73333329	0,031123236
CALML3-AS1	PLD2	CALML3-AS1\PLD2	0,73333329	0,031123236
CALML3-AS1	PMPCB	CALML3-AS1\PMPCB	0,73333329	0,031123236
CALML3-AS1	PNMAL1	CALML3-AS1\PNMAL1	0,699999928	0,043253969
CALML3-AS1	POGZ	CALML3-AS1\POGZ	0,799999952	0,013828263
CALML3-AS1	POMT2	CALML3-AS1\POMT2	0,899999917	0,002028219
CALML3-AS1	PRKAA2	CALML3-AS1\PRKAA2	0,899999917	0,002028219
CALML3-AS1	PRKAB1	CALML3-AS1\PRKAB1	0,849999964	0,006073633
CALML3-AS1	PRKAB2	CALML3-AS1\PRKAB2	0,816666603	0,010769401
CALML3-AS1	PROP1	CALML3-AS1\PROP1	0,883333266	0,003075397
CALML3-AS1	PRRG4	CALML3-AS1\PRRG4	0,966666639	0,000165344
CALML3-AS1	PSAT1	CALML3-AS1\PSAT1	0,966666639	0,000165344
CALML3-AS1	PSD3	CALML3-AS1\PSD3	0,833333313	0,008267196
CALML3-AS1	QSER1	CALML3-AS1\QSER1	0,816666603	0,010769401
CALML3-AS1	RAB14	CALML3-AS1\RAB14	0,74999994	0,025490521
CALML3-AS1	RAB23	CALML3-AS1\RAB23	0,766666651	0,021389991
CALML3-AS1	RABGAP1	CALML3-AS1\RABGAP1	0,933333278	0,000749559

CALML3-AS1	RANBP17	CALML3-AS1\RANBP17	0,916666567	0,001311728
CALML3-AS1	RASEF	CALML3-AS1\RASEF	0,816666603	0,010769401
CALML3-AS1	RAVER2	CALML3-AS1\RAVER2	0,699999928	0,043253969
CALML3-AS1	RGS9	CALML3-AS1\RGS9	0,783333302	0,017223325
CALML3-AS1	RHBDD2	CALML3-AS1\RHBDD2	0,883333266	0,003075397
CALML3-AS1	RMDN3	CALML3-AS1\RMDN3	0,803354323	0,012202381
CALML3-AS1	RNF144B	CALML3-AS1\RNF144B	0,783333302	0,017223325
CALML3-AS1	RNF170	CALML3-AS1\RNF170	0,816666603	0,010769401
CALML3-AS1	RNF212	CALML3-AS1\RNF212	0,783333302	0,017223325
CALML3-AS1	RPGRIP1L	CALML3-AS1\RPGRIP1L	0,716666639	0,036866181
CALML3-AS1	RPS6KA6	CALML3-AS1\RPS6KA6	0,74999994	0,025490521
CALML3-AS1	RRM2B	CALML3-AS1\RRM2B	0,816666603	0,010769401
CALML3-AS1	S100A14	CALML3-AS1\S100A14	0,716666639	0,036866181
CALML3-AS1	SARS2	CALML3-AS1\SARS2	0,694566727	0,043816138
CALML3-AS1	SCAMP1	CALML3-AS1\SCAMP1	0,949999988	0,000352734
CALML3-AS1	SCN4B	CALML3-AS1\SCN4B	0,716666639	0,036866181
CALML3-AS1	SDR42E1	CALML3-AS1\SDR42E1	0,816666603	0,010769401
CALML3-AS1	SEC61A1	CALML3-AS1\SEC61A1	0,849999964	0,006073633
CALML3-AS1	SERPINB5	CALML3-AS1\SERPINB5	0,933333278	0,000749559
CALML3-AS1	SGPL1	CALML3-AS1\SGPL1	0,933333278	0,000749559
CALML3-AS1	SIX1	CALML3-AS1\SIX1	0,883333266	0,003075397
CALML3-AS1	SIX4	CALML3-AS1\SIX4	0,933333278	0,000749559
CALML3-AS1	SLC22A23	CALML3-AS1\SLC22A23	0,699999928	0,043253969
CALML3-AS1	SLC44A5	CALML3-AS1\SLC44A5	0,74999994	0,025490521
CALML3-AS1	SLC45A4	CALML3-AS1\SLC45A4	0,716666639	0,036866181
CALML3-AS1	SLC46A1	CALML3-AS1\SLC46A1	0,883333266	0,003075397
CALML3-AS1	SLCO5A1	CALML3-AS1\SLCO5A1	0,699999928	0,043253969
CALML3-AS1	SMO	CALML3-AS1\SMO	0,74999994	0,025490521
CALML3-AS1	SOGA2	CALML3-AS1\SOGA2	0,74999994	0,025490521
CALML3-AS1	SPANXN2	CALML3-AS1\SPANXN2	0,716666639	0,036866181
CALML3-AS1	SPATA6	CALML3-AS1\SPATA6	0,74999994	0,025490521
CALML3-AS1	SPIN1	CALML3-AS1\SPIN1	0,883333266	0,003075397
CALML3-AS1	SPIN4	CALML3-AS1\SPIN4	0,74999994	0,025490521
CALML3-AS1	SPINT1	CALML3-AS1\SPINT1	0,866666615	0,004508378

CALML3-AS1	STARD7	CALML3-AS1\STARD7	0,816666603	0,010769401
CALML3-AS1	STON2	CALML3-AS1\STON2	0,916666567	0,001311728
CALML3-AS1	SUFU	CALML3-AS1\SUFU	0,73333329	0,031123236
CALML3-AS1	SUPT3H	CALML3-AS1\SUPT3H	0,828459144	0,008289241
CALML3-AS1	TAB3	CALML3-AS1\TAB3	0,933333278	0,000749559
CALML3-AS1	TCF24	CALML3-AS1\TCF24	0,716666639	0,036866181
CALML3-AS1	TM7SF3	CALML3-AS1\TM7SF3	0,883333266	0,003075397
CALML3-AS1	TMEM107	CALML3-AS1\TMEM107	0,816666603	0,010769401
CALML3-AS1	TMEM133	CALML3-AS1\TMEM133	0,949999988	0,000352734
CALML3-AS1	TMEM180	CALML3-AS1\TMEM180	0,864530981	0,004464286
CALML3-AS1	TMEM185A	CALML3-AS1\TMEM185A	0,899999917	0,002028219
CALML3-AS1	TMEM30B	CALML3-AS1\TMEM30B	0,949999988	0,000352734
CALML3-AS1	TNPO1	CALML3-AS1\TNPO1	0,883333266	0,003075397
CALML3-AS1	TOMM20	CALML3-AS1\TOMM20	0,866666615	0,004508378
CALML3-AS1	TOMM34	CALML3-AS1\TOMM34	0,74999994	0,025490521
CALML3-AS1	TP63	CALML3-AS1\TP63	0,699999928	0,043253969
CALML3-AS1	TRMT5	CALML3-AS1\TRMT5	0,799999952	0,013828263
CALML3-AS1	TRPM7	CALML3-AS1\TRPM7	0,73333329	0,031123236
CALML3-AS1	TSPAN13	CALML3-AS1\TSPAN13	0,766666651	0,021389991
CALML3-AS1	TTYH2	CALML3-AS1\TTYH2	0,849999964	0,006073633
CALML3-AS1	TXNL1	CALML3-AS1\TXNL1	0,799999952	0,013828263
CALML3-AS1	UBTD2	CALML3-AS1\UBTD2	0,828459144	0,008289241
CALML3-AS1	UEVLD	CALML3-AS1\UEVLD	0,830627799	0,007638889
CALML3-AS1	UMPS	CALML3-AS1\UMPS	0,745869875	0,026388889
CALML3-AS1	UNC5B	CALML3-AS1\UNC5B	0,833333313	0,008267196
CALML3-AS1	UPF1	CALML3-AS1\UPF1	0,716666639	0,036866181
CALML3-AS1	USP28	CALML3-AS1\USP28	0,883333266	0,003075397
CALML3-AS1	USP46	CALML3-AS1\USP46	0,783333302	0,017223325
CALML3-AS1	VPS35	CALML3-AS1\VPS35	0,766666651	0,021389991
CALML3-AS1	VWCE	CALML3-AS1\VWCE	0,816666603	0,010769401
CALML3-AS1	WASL	CALML3-AS1\WASL	0,916666567	0,001311728
CALML3-AS1	WDR61	CALML3-AS1\WDR61	0,694566727	0,043816138
CALML3-AS1	XPR1	CALML3-AS1\XPR1	0,816666603	0,010769401
CALML3-AS1	ZBTB41	CALML3-AS1\ZBTB41	0,783333302	0,017223325

CALML3-AS1	ZNF211	CALML3-AS1\ZNF211	0,849999964	0,006073633
CALML3-AS1	ZNF221	CALML3-AS1\ZNF221	0,883333266	0,003075397
CALML3-AS1	ZNF257	CALML3-AS1\ZNF257	0,699999928	0,043253969
CALML3-AS1	ZNF280B	CALML3-AS1\ZNF280B	0,74999994	0,025490521
CALML3-AS1	ZNF449	CALML3-AS1\ZNF449	0,761512935	0,021263227
CALML3-AS1	ZSCAN31	CALML3-AS1\ZSCAN31	0,716666639	0,036866181
CDKN2B-AS1	APPBP2	CDKN2B-AS1\APPBP2	0,699999928	0,043253969
CDKN2B-AS1	ARL1	CDKN2B-AS1\ARL1	0,799999952	0,013828263
CDKN2B-AS1	CCL25	CDKN2B-AS1\CCL25	0,716666639	0,036866181
CDKN2B-AS1	CDKL5	CDKN2B-AS1\CDKL5	0,866666615	0,004508378
CDKN2B-AS1	CYP26B1	CDKN2B-AS1\CYP26B1	0,799999952	0,013828263
CDKN2B-AS1	ERAP1	CDKN2B-AS1\ERAP1	0,74999994	0,025490521
CDKN2B-AS1	FAM163A	CDKN2B-AS1\FAM163A	0,849999964	0,006073633
CDKN2B-AS1	FRAS1	CDKN2B-AS1\FRAS1	0,716666639	0,036866181
CDKN2B-AS1	GPR64	CDKN2B-AS1\GPR64	0,799999952	0,013828263
CDKN2B-AS1	GXYLT1	CDKN2B-AS1\GXYLT1	0,716666639	0,036866181
CDKN2B-AS1	HDAC4	CDKN2B-AS1\HDAC4	0,719671547	0,033763226
CDKN2B-AS1	HDGFRP3	CDKN2B-AS1\HDGFRP3	0,699999928	0,043253969
CDKN2B-AS1	KCNIP3	CDKN2B-AS1\KCNIP3	0,849999964	0,006073633
CDKN2B-AS1	KIAA0319L	CDKN2B-AS1\KIAA0319L	0,716666639	0,036866181
CDKN2B-AS1	MAP10	CDKN2B-AS1\MAP10	0,783333302	0,017223325
CDKN2B-AS1	MAPK1	CDKN2B-AS1\MAPK1	0,778249502	0,017383156
CDKN2B-AS1	MED21	CDKN2B-AS1\MED21	0,769881189	0,018992504
CDKN2B-AS1	MLX	CDKN2B-AS1\MLX	0,728039861	0,031283069
CDKN2B-AS1	N4BP2L2	CDKN2B-AS1\N4BP2L2	0,73333329	0,031123236
CDKN2B-AS1	NIF3L1	CDKN2B-AS1\NIF3L1	0,783333302	0,017223325
CDKN2B-AS1	PLCXD1	CDKN2B-AS1\PLCXD1	0,73333329	0,031123236
CDKN2B-AS1	PNMAL1	CDKN2B-AS1\PNMAL1	0,766666651	0,021389991
CDKN2B-AS1	RASEF	CDKN2B-AS1\RASEF	0,799999952	0,013828263
CDKN2B-AS1	RCC2	CDKN2B-AS1\RCC2	0,799999952	0,013828263
CDKN2B-AS1	SCD5	CDKN2B-AS1\SCD5	0,699999928	0,043253969
CDKN2B-AS1	SFXN5	CDKN2B-AS1\SFXN5	0,719671547	0,033763226
CDKN2B-AS1	SLCO5A1	CDKN2B-AS1\SLCO5A1	0,816666603	0,010769401
CDKN2B-AS1	TMEM107	CDKN2B-AS1\TMEM107	0,716666639	0,036866181

CDKN2B-AS1	TRIM68	CDKN2B-AS1\TRIM68	0,74999994	0,025490521
CDKN2B-AS1	TYK2	CDKN2B-AS1\TYK2	0,716666639	0,036866181
CDKN2B-AS1	UNG	CDKN2B-AS1\UNG	0,849999964	0,006073633
CDKN2B-AS1	WDR61	CDKN2B-AS1\WDR61	0,736408114	0,02800926
CDKN2B-AS1	YY1AP1	CDKN2B-AS1\YY1AP1	0,74999994	0,025490521
CDKN2B-AS1	ZMAT3	CDKN2B-AS1\ZMAT3	0,899999917	0,002028219
CTBP1-AS2	ACAD10	CTBP1-AS2\ACAD10	0,686198473	0,047045857
CTBP1-AS2	AGPAT3	CTBP1-AS2\AGPAT3	0,783333302	0,017223325
CTBP1-AS2	AJUBA	CTBP1-AS2\AJUBA	0,766666651	0,021389991
CTBP1-AS2	ALDH7A1	CTBP1-AS2\ALDH7A1	0,816666603	0,010769401
CTBP1-AS2	ALS2	CTBP1-AS2\ALS2	0,74999994	0,025490521
CTBP1-AS2	ANK3	CTBP1-AS2\ANK3	0,933333278	0,000749559
CTBP1-AS2	ANKFY1	CTBP1-AS2\ANKFY1	0,833333313	0,008267196
CTBP1-AS2	AP1S1	CTBP1-AS2\AP1S1	0,799999952	0,013828263
CTBP1-AS2	AP2B1	CTBP1-AS2\AP2B1	0,783333302	0,017223325
CTBP1-AS2	APOBEC2	CTBP1-AS2\APOBEC2	0,916666567	0,001311728
CTBP1-AS2	APTX	CTBP1-AS2\APTX	0,799999952	0,013828263
CTBP1-AS2	ARL6IP1	CTBP1-AS2\ARL6IP1	0,783333302	0,017223325
CTBP1-AS2	ARV1	CTBP1-AS2\ARV1	0,799999952	0,013828263
CTBP1-AS2	ATHL1	CTBP1-AS2\ATHL1	0,699999928	0,043253969
CTBP1-AS2	ATP6V0B	CTBP1-AS2\ATP6V0B	0,716666639	0,036866181
CTBP1-AS2	ATP6V0D1	CTBP1-AS2\ATP6V0D1	0,73333329	0,031123236
CTBP1-AS2	ATP6V1C1	CTBP1-AS2\ATP6V1C1	0,783333302	0,017223325
CTBP1-AS2	ATPAF1	CTBP1-AS2\ATPAF1	0,73333329	0,031123236
CTBP1-AS2	ATRNL1	CTBP1-AS2\ATRNL1	0,799999952	0,013828263
CTBP1-AS2	AUTS2	CTBP1-AS2\AUTS2	0,766666651	0,021389991
CTBP1-AS2	BBS4	CTBP1-AS2\BBS4	0,766666651	0,021389991
CTBP1-AS2	BRD1	CTBP1-AS2\BRD1	0,883333266	0,003075397
CTBP1-AS2	C15orf41	CTBP1-AS2\C15orf41	0,933333278	0,000749559
CTBP1-AS2	C19orf54	CTBP1-AS2\C19orf54	0,716666639	0,036866181
CTBP1-AS2	C19orf82	CTBP1-AS2\C19orf82	0,966666639	0,000165344
CTBP1-AS2	C1orf109	CTBP1-AS2\C1orf109	0,783333302	0,017223325
CTBP1-AS2	C4A	CTBP1-AS2\C4A	0,716666639	0,036866181
CTBP1-AS2	C4B	CTBP1-AS2\C4B	0,716666639	0,036866181

CTBP1-AS2	CALML3	CTBP1-AS2\CALML3	0,699999928	0,043253969
CTBP1-AS2	CAMSAP1	CTBP1-AS2\CAMSAP1	0,933333278	0,000749559
CTBP1-AS2	CBLN3	CTBP1-AS2\CBLN3	0,716666639	0,036866181
CTBP1-AS2	CBS	CTBP1-AS2\CBS	0,816666603	0,010769401
CTBP1-AS2	CCDC30	CTBP1-AS2\CCDC30	0,799999952	0,013828263
CTBP1-AS2	CCDC8	CTBP1-AS2\CCDC8	0,883333266	0,003075397
CTBP1-AS2	CDC42BPG	CTBP1-AS2\CDC42BPG	0,728039861	0,031283069
CTBP1-AS2	CDH1	CTBP1-AS2\CDH1	0,74999994	0,025490521
CTBP1-AS2	CDKL3	CTBP1-AS2\CDKL3	0,849999964	0,006073633
CTBP1-AS2	CDS1	CTBP1-AS2\CDS1	0,783333302	0,017223325
CTBP1-AS2	CETN3	CTBP1-AS2\CETN3	0,766666651	0,021389991
CTBP1-AS2	CLCN5	CTBP1-AS2\CLCN5	0,799999952	0,013828263
CTBP1-AS2	CLDN12	CTBP1-AS2\CLDN12	0,866666615	0,004508378
CTBP1-AS2	CLHC1	CTBP1-AS2\CLHC1	0,73333329	0,031123236
CTBP1-AS2	CLINT1	CTBP1-AS2\CLINT1	0,73333329	0,031123236
CTBP1-AS2	CLTC	CTBP1-AS2\CLTC	0,74999994	0,025490521
CTBP1-AS2	CNTNAP3	CTBP1-AS2\CNTNAP3	0,716666639	0,036866181
CTBP1-AS2	CNTNAP3B	CTBP1-AS2\CNTNAP3B	0,766666651	0,021389991
CTBP1-AS2	COG5	CTBP1-AS2\COG5	0,73333329	0,031123236
CTBP1-AS2	COG8	CTBP1-AS2\COG8	0,694566727	0,043816138
CTBP1-AS2	CROT	CTBP1-AS2\CROT	0,966666639	0,000165344
CTBP1-AS2	CUX1	CTBP1-AS2\CUX1	0,766666651	0,021389991
CTBP1-AS2	DCAKD	CTBP1-AS2\DCAKD	0,73333329	0,031123236
CTBP1-AS2	DDX31	CTBP1-AS2\DDX31	0,866666615	0,004508378
CTBP1-AS2	DET1	CTBP1-AS2\DET1	0,766666651	0,021389991
CTBP1-AS2	DHTKD1	CTBP1-AS2\DHTKD1	0,883333266	0,003075397
CTBP1-AS2	DMRT2	CTBP1-AS2\DMRT2	0,766666651	0,021389991
CTBP1-AS2	DNAJC16	CTBP1-AS2\DNAJC16	0,99999994	5,51146E-06
CTBP1-AS2	DPH6	CTBP1-AS2\DPH6	0,883333266	0,003075397
CTBP1-AS2	DTNB	CTBP1-AS2\DTNB	0,883333266	0,003075397
CTBP1-AS2	DUOX1	CTBP1-AS2\DUOX1	0,895405293	0,002105379
CTBP1-AS2	DUOXA1	CTBP1-AS2\DUOXA1	0,799999952	0,013828263
CTBP1-AS2	ELOVL6	CTBP1-AS2\ELOVL6	0,799999952	0,013828263
CTBP1-AS2	EMC1	CTBP1-AS2\EMC1	0,74999994	0,025490521

CTBP1-AS2	EPN3	CTBP1-AS2\EPN3	0,916666567	0,001311728
CTBP1-AS2	EPS15L1	CTBP1-AS2\EPS15L1	0,816666603	0,010769401
CTBP1-AS2	ESRP1	CTBP1-AS2\ESRP1	0,916666567	0,001311728
CTBP1-AS2	ESRP2	CTBP1-AS2\ESRP2	0,883333266	0,003075397
CTBP1-AS2	EVA1A	CTBP1-AS2\EVA1A	0,73333329	0,031123236
CTBP1-AS2	EXOC7	CTBP1-AS2\EXOC7	0,830627799	0,007638889
CTBP1-AS2	EYA2	CTBP1-AS2\EYA2	0,799999952	0,013828263
CTBP1-AS2	FAHD1	CTBP1-AS2\FAHD1	0,783333302	0,017223325
CTBP1-AS2	FAM120A	CTBP1-AS2\FAM120A	0,699999928	0,043253969
CTBP1-AS2	FAM154B	CTBP1-AS2\FAM154B	0,796724617	0,013227513
CTBP1-AS2	FAM160A1	CTBP1-AS2\FAM160A1	0,699999928	0,043253969
CTBP1-AS2	FAM199X	CTBP1-AS2\FAM199X	0,74999994	0,025490521
CTBP1-AS2	FBXO22	CTBP1-AS2\FBXO22	0,686198473	0,046968695
CTBP1-AS2	FBXW11	CTBP1-AS2\FBXW11	0,881482542	0,003009259
CTBP1-AS2	FEM1B	CTBP1-AS2\FEM1B	0,899999917	0,002028219
CTBP1-AS2	FKTN	CTBP1-AS2\FKTN	0,799999952	0,013828263
CTBP1-AS2	FLVCR1	CTBP1-AS2\FLVCR1	0,816666603	0,010769401
CTBP1-AS2	FOXE1	CTBP1-AS2\FOXE1	0,799999952	0,013828263
CTBP1-AS2	FSTL4	CTBP1-AS2\FSTL4	0,783333302	0,017223325
CTBP1-AS2	GGA1	CTBP1-AS2\GGA1	0,699999928	0,043253969
CTBP1-AS2	GGA2	CTBP1-AS2\GGA2	0,966666639	0,000165344
CTBP1-AS2	GGCT	CTBP1-AS2\GGCT	0,833333313	0,008267196
CTBP1-AS2	GINS3	CTBP1-AS2\GINS3	0,73333329	0,031123236
CTBP1-AS2	GLI2	CTBP1-AS2\GLI2	0,883333266	0,003075397
CTBP1-AS2	GPHN	CTBP1-AS2\GPHN	0,716666639	0,036866181
CTBP1-AS2	GPS1	CTBP1-AS2\GPS1	0,866666615	0,004508378
CTBP1-AS2	GRHL2	CTBP1-AS2\GRHL2	0,849999964	0,006073633
CTBP1-AS2	GRIP1	CTBP1-AS2\GRIP1	0,74999994	0,025490521
CTBP1-AS2	GRTP1	CTBP1-AS2\GRTP1	0,816666603	0,010769401
CTBP1-AS2	GTF2H4	CTBP1-AS2\GTF2H4	0,74999994	0,025490521
CTBP1-AS2	GTF2I	CTBP1-AS2\GTF2I	0,766666651	0,021389991
CTBP1-AS2	GTF3C4	CTBP1-AS2\GTF3C4	0,816666603	0,010769401
CTBP1-AS2	HIST2H2BF	CTBP1-AS2\HIST2H2BF	0,883333266	0,003075397
CTBP1-AS2	HN1L	CTBP1-AS2\HN1L	0,899999917	0,002028219

CTBP1-AS2	HOMER2	CTBP1-AS2\HOMER2	0,816666603	0,010769401
CTBP1-AS2	HOMER3	CTBP1-AS2\HOMER3	0,699999928	0,043253969
CTBP1-AS2	HPS5	CTBP1-AS2\HPS5	0,783333302	0,017223325
CTBP1-AS2	HPSE	CTBP1-AS2\HPSE	0,833333313	0,008267196
CTBP1-AS2	IFT140	CTBP1-AS2\IFT140	0,74999994	0,025490521
CTBP1-AS2	IGFBP2	CTBP1-AS2\IGFBP2	0,716666639	0,036866181
CTBP1-AS2	IGSF3	CTBP1-AS2\IGSF3	0,699999928	0,043253969
CTBP1-AS2	IQCE	CTBP1-AS2\IQCE	0,716666639	0,036866181
CTBP1-AS2	IQCH	CTBP1-AS2\IQCH	0,783333302	0,017223325
CTBP1-AS2	ITGA2	CTBP1-AS2\ITGA2	0,816666603	0,010769401
CTBP1-AS2	KDM4D	CTBP1-AS2\KDM4D	0,928878427	0,000815697
CTBP1-AS2	KIAA1549	CTBP1-AS2\KIAA1549	0,783333302	0,017223325
CTBP1-AS2	KLHDC10	CTBP1-AS2\KLHDC10	0,799999952	0,013828263
CTBP1-AS2	KRTAP5-8	CTBP1-AS2\KRTAP5-8	0,833333313	0,008267196
CTBP1-AS2	LAMP1	CTBP1-AS2\LAMP1	0,744776368	0,026047178
CTBP1-AS2	LAMP2	CTBP1-AS2\LAMP2	0,778249502	0,017383156
CTBP1-AS2	LARS	CTBP1-AS2\LARS	0,699999928	0,043253969
CTBP1-AS2	LIMK1	CTBP1-AS2\LIMK1	0,845195651	0,006205908
CTBP1-AS2	LMBR1	CTBP1-AS2\LMBR1	0,783333302	0,017223325
CTBP1-AS2	LRIG3	CTBP1-AS2\LRIG3	0,816666603	0,010769401
CTBP1-AS2	LRPPRC	CTBP1-AS2\LRPPRC	0,833333313	0,008267196
CTBP1-AS2	LRRC27	CTBP1-AS2\LRRC27	0,74999994	0,025490521
CTBP1-AS2	M1AP	CTBP1-AS2\M1AP	0,866666615	0,004508378
CTBP1-AS2	MAML3	CTBP1-AS2\MAML3	0,98333329	4,96032E-05
CTBP1-AS2	MAP3K2	CTBP1-AS2\MAP3K2	0,799999952	0,013828263
CTBP1-AS2	MARVELD2	CTBP1-AS2\MARVELD2	0,816666603	0,010769401
CTBP1-AS2	MAST4	CTBP1-AS2\MAST4	0,70293504	0,040145501
CTBP1-AS2	MCOLN3	CTBP1-AS2\MCOLN3	0,849999964	0,006073633
CTBP1-AS2	MCTP2	CTBP1-AS2\MCTP2	0,849999964	0,006073633
CTBP1-AS2	MED22	CTBP1-AS2\MED22	0,695015132	0,044808201
CTBP1-AS2	METTL8	CTBP1-AS2\METTL8	0,866666615	0,004508378
CTBP1-AS2	MFSD3	CTBP1-AS2\MFSD3	0,916666567	0,001311728
CTBP1-AS2	MPP5	CTBP1-AS2\MPP5	0,783333302	0,017223325
CTBP1-AS2	MYO10	CTBP1-AS2\MYO10	0,866666615	0,004508378

CTBP1-AS2	N4BP1	CTBP1-AS2\N4BP1	0,866666615	0,004508378
CTBP1-AS2	NAA50	CTBP1-AS2\NAA50	0,799999952	0,013828263
CTBP1-AS2	NAGK	CTBP1-AS2\NAGK	0,883333266	0,003075397
CTBP1-AS2	NCS1	CTBP1-AS2\NCS1	0,799999952	0,013828263
CTBP1-AS2	NDUFA4	CTBP1-AS2\NDUFA4	0,766666651	0,021389991
CTBP1-AS2	NDUFB5	CTBP1-AS2\NDUFB5	0,699999928	0,043253969
CTBP1-AS2	NEO1	CTBP1-AS2\NEO1	0,699999928	0,043253969
CTBP1-AS2	NETO2	CTBP1-AS2\NETO2	0,799999952	0,013828263
CTBP1-AS2	NHLRC3	CTBP1-AS2\NHLRC3	0,766666651	0,021389991
CTBP1-AS2	NIPSNAP1	CTBP1-AS2\NIPSNAP1	0,816666603	0,010769401
CTBP1-AS2	NPNT	CTBP1-AS2\NPNT	0,73333329	0,031123236
CTBP1-AS2	NRCAM	CTBP1-AS2\NRCAM	0,866666615	0,004508378
CTBP1-AS2	NUDT12	CTBP1-AS2\NUDT12	0,887037039	0,002469136
CTBP1-AS2	NXN	CTBP1-AS2\NXN	0,694566727	0,043816138
CTBP1-AS2	OSBPL3	CTBP1-AS2\OSBPL3	0,933333278	0,000749559
CTBP1-AS2	OXCT1	CTBP1-AS2\OXCT1	0,883333266	0,003075397
CTBP1-AS2	PABPC4L	CTBP1-AS2\PABPC4L	0,816666603	0,010769401
CTBP1-AS2	PANK1	CTBP1-AS2\PANK1	0,816666603	0,010769401
CTBP1-AS2	PASK	CTBP1-AS2\PASK	0,849999964	0,006073633
CTBP1-AS2	PAX1	CTBP1-AS2\PAX1	0,783333302	0,017223325
CTBP1-AS2	PAX9	CTBP1-AS2\PAX9	0,933333278	0,000749559
CTBP1-AS2	PFN4	CTBP1-AS2\PFN4	0,699999928	0,043253969
CTBP1-AS2	PGAP1	CTBP1-AS2\PGAP1	0,833333313	0,008267196
CTBP1-AS2	PIAS2	CTBP1-AS2\PIAS2	0,699999928	0,043253969
CTBP1-AS2	PIFO	CTBP1-AS2\PIFO	0,883333266	0,003075397
CTBP1-AS2	PKP1	CTBP1-AS2\PKP1	0,766666651	0,021389991
CTBP1-AS2	PKP4	CTBP1-AS2\PKP4	0,849999964	0,006073633
CTBP1-AS2	PLA2G12A	CTBP1-AS2\PLA2G12A	0,866666615	0,004508378
CTBP1-AS2	PLD2	CTBP1-AS2\PLD2	0,799999952	0,013828263
CTBP1-AS2	PLEK2	CTBP1-AS2\PLEK2	0,74999994	0,025490521
CTBP1-AS2	PMPCB	CTBP1-AS2\PMPCB	0,949999988	0,000352734
CTBP1-AS2	POMT2	CTBP1-AS2\POMT2	0,716666639	0,036866181
CTBP1-AS2	PPP5C	CTBP1-AS2\PPP5C	0,766666651	0,021389991
CTBP1-AS2	PRKAB1	CTBP1-AS2\PRKAB1	0,699999928	0,043253969

CTBP1-AS2	PRRC1	CTBP1-AS2\PRRC1	0,849999964	0,006073633
CTBP1-AS2	PRRG4	CTBP1-AS2\PRRG4	0,73333329	0,031123236
CTBP1-AS2	PRSS8	CTBP1-AS2\PRSS8	0,916666567	0,001311728
CTBP1-AS2	PSD3	CTBP1-AS2\PSD3	0,949999988	0,000352734
CTBP1-AS2	PSMD12	CTBP1-AS2\PSMD12	0,766666651	0,021389991
CTBP1-AS2	PTPRK	CTBP1-AS2\PTPRK	0,73333329	0,031123236
CTBP1-AS2	PTPRT	CTBP1-AS2\PTPRT	0,74999994	0,025490521
CTBP1-AS2	RAB14	CTBP1-AS2\RAB14	0,799999952	0,013828263
CTBP1-AS2	RAB23	CTBP1-AS2\RAB23	0,716666639	0,036866181
CTBP1-AS2	RAB40C	CTBP1-AS2\RAB40C	0,933333278	0,000749559
CTBP1-AS2	RABGAP1	CTBP1-AS2\RABGAP1	0,783333302	0,017223325
CTBP1-AS2	RAD50	CTBP1-AS2\RAD50	0,799999952	0,013828263
CTBP1-AS2	RANBP17	CTBP1-AS2\RANBP17	0,73333329	0,031123236
CTBP1-AS2	RASL10A	CTBP1-AS2\RASL10A	0,82009083	0,009281305
CTBP1-AS2	RFESD	CTBP1-AS2\RFESD	0,799999952	0,013828263
CTBP1-AS2	RMDN3	CTBP1-AS2\RMDN3	0,970719814	8,81834E-05
CTBP1-AS2	RNF170	CTBP1-AS2\RNF170	0,74999994	0,025490521
CTBP1-AS2	RNF212	CTBP1-AS2\RNF212	0,699999928	0,043253969
CTBP1-AS2	RNF32	CTBP1-AS2\RNF32	0,74999994	0,025490521
CTBP1-AS2	RPGRIP1L	CTBP1-AS2\RPGRIP1L	0,833333313	0,008267196
CTBP1-AS2	RPS6KA6	CTBP1-AS2\RPS6KA6	0,833333313	0,008267196
CTBP1-AS2	RRAGB	CTBP1-AS2\RRAGB	0,799999952	0,013828263
CTBP1-AS2	RTF1	CTBP1-AS2\RTF1	0,766666651	0,021389991
CTBP1-AS2	S100A14	CTBP1-AS2\S100A14	0,799999952	0,013828263
CTBP1-AS2	SARS2	CTBP1-AS2\SARS2	0,694566727	0,043816138
CTBP1-AS2	SDR42E1	CTBP1-AS2\SDR42E1	0,899999917	0,002028219
CTBP1-AS2	SGPL1	CTBP1-AS2\SGPL1	0,816666603	0,010769401
CTBP1-AS2	SGPP2	CTBP1-AS2\SGPP2	0,699999928	0,043253969
CTBP1-AS2	SIX1	CTBP1-AS2\SIX1	0,699999928	0,043253969
CTBP1-AS2	SIX4	CTBP1-AS2\SIX4	0,783333302	0,017223325
CTBP1-AS2	SLC22A23	CTBP1-AS2\SLC22A23	0,73333329	0,031123236
CTBP1-AS2	SLC22A5	CTBP1-AS2\SLC22A5	0,74999994	0,025490521
CTBP1-AS2	SLC30A1	CTBP1-AS2\SLC30A1	0,766666651	0,021389991
CTBP1-AS2	SLC30A6	CTBP1-AS2\SLC30A6	0,74999994	0,025490521

CTBP1-AS2	SLC44A3	CTBP1-AS2\SLC44A3	0,799999952	0,013828263
CTBP1-AS2	SLC46A1	CTBP1-AS2\SLC46A1	0,883333266	0,003075397
CTBP1-AS2	SMO	CTBP1-AS2\SMO	0,833333313	0,008267196
CTBP1-AS2	SNX1	CTBP1-AS2\SNX1	0,783333302	0,017223325
CTBP1-AS2	SOGA2	CTBP1-AS2\SOGA2	0,866666615	0,004508378
CTBP1-AS2	SORBS2	CTBP1-AS2\SORBS2	0,849999964	0,006073633
CTBP1-AS2	SORCS2	CTBP1-AS2\SORCS2	0,73333329	0,031123236
CTBP1-AS2	SPANXN2	CTBP1-AS2\SPANXN2	0,766666651	0,021389991
CTBP1-AS2	SPATA6	CTBP1-AS2\SPATA6	0,816666603	0,010769401
CTBP1-AS2	SPIN1	CTBP1-AS2\SPIN1	0,73333329	0,031123236
CTBP1-AS2	SPINT1	CTBP1-AS2\SPINT1	0,816666603	0,010769401
CTBP1-AS2	SPIRE2	CTBP1-AS2\SPIRE2	0,811722577	0,010769401
CTBP1-AS2	STARD7	CTBP1-AS2\STARD7	0,916666567	0,001311728
CTBP1-AS2	STEAP2	CTBP1-AS2\STEAP2	0,73333329	0,031123236
CTBP1-AS2	STX6	CTBP1-AS2\STX6	0,766666651	0,021389991
CTBP1-AS2	SUPT3H	CTBP1-AS2\SUPT3H	0,79498601	0,013833774
CTBP1-AS2	SYBU	CTBP1-AS2\SYBU	0,849999964	0,006073633
CTBP1-AS2	SYT1	CTBP1-AS2\SYT1	0,74999994	0,025490521
CTBP1-AS2	TM7SF3	CTBP1-AS2\TM7SF3	0,783333302	0,017223325
CTBP1-AS2	TMEM107	CTBP1-AS2\TMEM107	0,716666639	0,036866181
CTBP1-AS2	TMEM180	CTBP1-AS2\TMEM180	0,898434162	0,002050265
CTBP1-AS2	TMEM185A	CTBP1-AS2\TMEM185A	0,849999964	0,006073633
CTBP1-AS2	TNPO1	CTBP1-AS2\TNPO1	0,699999928	0,043253969
CTBP1-AS2	TP63	CTBP1-AS2\TP63	0,783333302	0,017223325
CTBP1-AS2	TRIM7	CTBP1-AS2\TRIM7	0,74999994	0,025490521
CTBP1-AS2	TRMT10B	CTBP1-AS2\TRMT10B	0,783333302	0,017223325
CTBP1-AS2	TRMT5	CTBP1-AS2\TRMT5	0,73333329	0,031123236
CTBP1-AS2	TRPM7	CTBP1-AS2\TRPM7	0,899999917	0,002028219
CTBP1-AS2	TRUB1	CTBP1-AS2\TRUB1	0,716666639	0,036866181
CTBP1-AS2	TSPAN13	CTBP1-AS2\TSPAN13	0,74999994	0,025490521
CTBP1-AS2	TTC22	CTBP1-AS2\TTC22	0,833333313	0,008267196
CTBP1-AS2	UBAC1	CTBP1-AS2\UBAC1	0,799999952	0,013828263
CTBP1-AS2	UBAP2	CTBP1-AS2\UBAP2	0,74999994	0,025490521
CTBP1-AS2	UBE2V2	CTBP1-AS2\UBE2V2	0,761512935	0,021814374

CTBP1-AS2	UBFD1	CTBP1-AS2\UBFD1	0,816666603	0,010769401
CTBP1-AS2	UEVLD	CTBP1-AS2\UEVLD	0,796724617	0,013227513
CTBP1-AS2	UNC5B	CTBP1-AS2\UNC5B	0,833333313	0,008267196
CTBP1-AS2	UPF1	CTBP1-AS2\UPF1	0,866666615	0,004508378
CTBP1-AS2	USP40	CTBP1-AS2\USP40	0,833333313	0,008267196
CTBP1-AS2	VAC14	CTBP1-AS2\VAC14	0,916666567	0,001311728
CTBP1-AS2	VMA21	CTBP1-AS2\VMA21	0,766666651	0,021389991
CTBP1-AS2	WASL	CTBP1-AS2\WASL	0,699999928	0,043253969
CTBP1-AS2	WDR91	CTBP1-AS2\WDR91	0,73333329	0,031123236
CTBP1-AS2	WNK2	CTBP1-AS2\WNK2	0,816666603	0,010769401
CTBP1-AS2	WWOX	CTBP1-AS2\WWOX	0,761512935	0,021263227
CTBP1-AS2	XPR1	CTBP1-AS2\XPR1	0,766666651	0,021389991
CTBP1-AS2	XYLT2	CTBP1-AS2\XYLT2	0,849999964	0,006073633
CTBP1-AS2	ZBTB41	CTBP1-AS2\ZBTB41	0,833333313	0,008267196
CTBP1-AS2	ZMYM3	CTBP1-AS2\ZMYM3	0,783333302	0,017223325
CTBP1-AS2	ZNF132	CTBP1-AS2\ZNF132	0,966666639	0,000165344
CTBP1-AS2	ZNF221	CTBP1-AS2\ZNF221	0,766666651	0,021389991
CTBP1-AS2	ZNF257	CTBP1-AS2\ZNF257	0,716666639	0,036866181
CTBP1-AS2	ZNF280B	CTBP1-AS2\ZNF280B	0,866666615	0,004508378
CTBP1-AS2	ZNF449	CTBP1-AS2\ZNF449	0,786617756	0,015288801
CTBP1-AS2	ZNF543	CTBP1-AS2\ZNF543	0,816666603	0,010769401
CTBP1-AS2	ZNF544	CTBP1-AS2\ZNF544	0,74999994	0,025490521
CTBP1-AS2	ZNF554	CTBP1-AS2\ZNF554	0,728918254	0,031349208
CTBP1-AS2	ZNF562	CTBP1-AS2\ZNF562	0,74999994	0,025490521
CTBP1-AS2	ZNF626	CTBP1-AS2\ZNF626	0,699999928	0,043253969
CTBP1-AS2	ZRANB3	CTBP1-AS2\ZRANB3	0,73333329	0,031123236
CTBP1-AS2	ZSCAN31	CTBP1-AS2\ZSCAN31	0,833333313	0,008267196
DKFZP434I0714	AKR1B1	DKFZP434I0714\AKR1B1	0,849999964	0,006073633
DKFZP434I0714	ALDH7A1	DKFZP434I0714\ALDH7A1	0,699999928	0,043253969
DKFZP434I0714	ALS2	DKFZP434I0714\ALS2	0,883333266	0,003075397
DKFZP434I0714	AP2B1	DKFZP434I0714\AP2B1	0,699999928	0,043253969
DKFZP434I0714	ARL6IP1	DKFZP434I0714\ARL6IP1	0,699999928	0,043253969
DKFZP434I0714	ARV1	DKFZP434I0714\ARV1	0,766666651	0,021389991
DKFZP434I0714	ATP2C1	DKFZP434I0714\ATP2C1	0,766666651	0,021389991

DKFZP434I0714	AUTS2	DKFZP434I0714\AUTS2	0,74999994	0,025490521
DKFZP434I0714	AZIN1	DKFZP434I0714\AZIN1	0,716666639	0,036866181
DKFZP434I0714	BAIAP2	DKFZP434I0714\BAIAP2	0,73333329	0,031123236
DKFZP434I0714	BBS4	DKFZP434I0714\BBS4	0,799999952	0,013828263
DKFZP434I0714	BRD1	DKFZP434I0714\BRD1	0,699999928	0,043253969
DKFZP434I0714	BTBD3	DKFZP434I0714\BTBD3	0,833333313	0,008267196
DKFZP434I0714	C14orf132	DKFZP434I0714\C14orf132	0,728039861	0,031283069
DKFZP434I0714	C14orf39	DKFZP434I0714\C14orf39	0,799999952	0,013828263
DKFZP434I0714	C1orf109	DKFZP434I0714\C1orf109	0,766666651	0,021389991
DKFZP434I0714	C4A	DKFZP434I0714\C4A	0,766666651	0,021389991
DKFZP434I0714	C4B	DKFZP434I0714\C4B	0,766666651	0,021389991
DKFZP434I0714	C5orf15	DKFZP434I0714\C5orf15	0,849999964	0,006073633
DKFZP434I0714	C5orf24	DKFZP434I0714\C5orf24	0,899999917	0,002028219
DKFZP434I0714	CACUL1	DKFZP434I0714\CACUL1	0,766666651	0,021389991
DKFZP434I0714	CALR	DKFZP434I0714\CALR	0,766666651	0,021389991
DKFZP434I0714	CBS	DKFZP434I0714\CBS	0,816666603	0,010769401
DKFZP434I0714	CCDC30	DKFZP434I0714\CCDC30	0,849999964	0,006073633
DKFZP434I0714	CCDC73	DKFZP434I0714\CCDC73	0,786617756	0,015288801
DKFZP434I0714	CCZ1	DKFZP434I0714\CCZ1	0,766666651	0,021389991
DKFZP434I0714	CDC42BPG	DKFZP434I0714\CDC42BPG	0,694566727	0,043816138
DKFZP434I0714	CDH2	DKFZP434I0714\CDH2	0,74999994	0,025490521
DKFZP434I0714	CDS1	DKFZP434I0714\CDS1	0,699999928	0,043253969
DKFZP434I0714	CETN3	DKFZP434I0714\CETN3	0,866666615	0,004508378
DKFZP434I0714	CLDN12	DKFZP434I0714\CLDN12	0,783333302	0,017223325
DKFZP434I0714	CLHC1	DKFZP434I0714\CLHC1	0,799999952	0,013828263
DKFZP434I0714	CLINT1	DKFZP434I0714\CLINT1	0,699999928	0,043253969
DKFZP434I0714	CLSTN1	DKFZP434I0714\CLSTN1	0,73333329	0,031123236
DKFZP434I0714	CLTC	DKFZP434I0714\CLTC	0,716666639	0,036866181
DKFZP434I0714	CNTNAP2	DKFZP434I0714\CNTNAP2	0,699999928	0,043253969
DKFZP434I0714	COG5	DKFZP434I0714\COG5	0,783333302	0,017223325
DKFZP434I0714	COG8	DKFZP434I0714\COG8	0,962351501	0,000165344
DKFZP434I0714	COX19	DKFZP434I0714\COX19	0,716666639	0,036866181
DKFZP434I0714	DDX31	DKFZP434I0714\DDX31	0,716666639	0,036866181
DKFZP434I0714	DET1	DKFZP434I0714\DET1	0,699999928	0,043253969

DKFZP434I0714	DNAI1	DKFZP434I0714\DNAI1	0,833333313	0,008267196
DKFZP434I0714	DNAJC19	DKFZP434I0714\DNAJC19	0,866666615	0,004508378
DKFZP434I0714	DNAJC21	DKFZP434I0714\DNAJC21	0,716666639	0,036866181
DKFZP434I0714	DNAL4	DKFZP434I0714\DNAL4	0,699999928	0,043253969
DKFZP434I0714	EDA2R	DKFZP434I0714\EDA2R	0,799999952	0,013828263
DKFZP434I0714	EIF2AK4	DKFZP434I0714\EIF2AK4	0,716666639	0,036866181
DKFZP434I0714	ELOVL6	DKFZP434I0714\ELOVL6	0,699999928	0,043253969
DKFZP434I0714	EMC1	DKFZP434I0714\EMC1	0,74999994	0,025490521
DKFZP434I0714	EMC10	DKFZP434I0714\EMC10	0,719671547	0,033763226
DKFZP434I0714	EXOSC10	DKFZP434I0714\EXOSC10	0,899999917	0,002028219
DKFZP434I0714	FAM199X	DKFZP434I0714\FAM199X	0,716666639	0,036866181
DKFZP434I0714	FAM218A	DKFZP434I0714\FAM218A	0,816666603	0,010769401
DKFZP434I0714	FAM45A	DKFZP434I0714\FAM45A	0,74999994	0,025490521
DKFZP434I0714	FEM1B	DKFZP434I0714\FEM1B	0,716666639	0,036866181
DKFZP434I0714	FKTN	DKFZP434I0714\FKTN	0,783333302	0,017223325
DKFZP434I0714	FLVCR1	DKFZP434I0714\FLVCR1	0,74999994	0,025490521
DKFZP434I0714	FRAS1	DKFZP434I0714\FRAS1	0,699999928	0,043253969
DKFZP434I0714	GGCT	DKFZP434I0714\GGCT	0,849999964	0,006073633
DKFZP434I0714	GINS3	DKFZP434I0714\GINS3	0,783333302	0,017223325
DKFZP434I0714	GNPDA2	DKFZP434I0714\GNPDA2	0,849999964	0,006073633
DKFZP434I0714	GOLM1	DKFZP434I0714\GOLM1	0,899999917	0,002028219
DKFZP434I0714	GPC4	DKFZP434I0714\GPC4	0,766666651	0,021389991
DKFZP434I0714	GTF2H3	DKFZP434I0714\GTF2H3	0,866666615	0,004508378
DKFZP434I0714	GTF2H4	DKFZP434I0714\GTF2H4	0,864530981	0,004596561
DKFZP434I0714	GTF2IRD2	DKFZP434I0714\GTF2IRD2	0,816666603	0,010769401
DKFZP434I0714	GTF3C4	DKFZP434I0714\GTF3C4	0,833333313	0,008267196
DKFZP434I0714	GXYLT1	DKFZP434I0714\GXYLT1	0,699999928	0,043253969
DKFZP434I0714	HDGFRP3	DKFZP434I0714\HDGFRP3	0,766666651	0,021389991
DKFZP434I0714	HN1L	DKFZP434I0714\HN1L	0,74999994	0,025490521
DKFZP434I0714	HOMER2	DKFZP434I0714\HOMER2	0,766666651	0,021389991
DKFZP434I0714	HPSE	DKFZP434I0714\HPSE	0,783333302	0,017223325
DKFZP434I0714	IFLTD1	DKFZP434I0714\IFLTD1	0,73333329	0,031123236
DKFZP434I0714	IGF1R	DKFZP434I0714\IGF1R	0,699999928	0,043253969
DKFZP434I0714	IGSF11	DKFZP434I0714\IGSF11	0,699999928	0,043253969

DKFZP434I0714	IGSF3	DKFZP434I0714\IGSF3	0,883333266	0,003075397
DKFZP434I0714	IKBIP	DKFZP434I0714\IKBIP	0,783333302	0,017223325
DKFZP434I0714	IKZF5	DKFZP434I0714\IKZF5	0,799999952	0,013828263
DKFZP434I0714	KAL1	DKFZP434I0714\KAL1	0,866666615	0,004508378
DKFZP434I0714	KDM4B	DKFZP434I0714\KDM4B	0,799999952	0,013828263
DKFZP434I0714	KIAA1644	DKFZP434I0714\KIAA1644	0,833333313	0,008267196
DKFZP434I0714	KIAA1841	DKFZP434I0714\KIAA1841	0,73333329	0,031123236
DKFZP434I0714	KIF3A	DKFZP434I0714\KIF3A	0,849999964	0,006073633
DKFZP434I0714	KLHDC10	DKFZP434I0714\KLHDC10	0,73333329	0,031123236
DKFZP434I0714	KRTAP5-8	DKFZP434I0714\KRTAP5-8	0,716666639	0,036866181
DKFZP434I0714	KTN1	DKFZP434I0714\KTN1	0,899999917	0,002028219
DKFZP434I0714	LARS	DKFZP434I0714\LARS	0,866666615	0,004508378
DKFZP434I0714	LEPREL1	DKFZP434I0714\LEPREL1	0,783333302	0,017223325
DKFZP434I0714	LGI3	DKFZP434I0714\LGI3	0,816666603	0,010769401
DKFZP434I0714	LPHN3	DKFZP434I0714\LPHN3	0,716666639	0,036866181
DKFZP434I0714	LRPPRC	DKFZP434I0714\LRPPRC	0,849999964	0,006073633
DKFZP434I0714	LRRK1	DKFZP434I0714\LRRK1	0,783333302	0,017223325
DKFZP434I0714	LZIC	DKFZP434I0714\LZIC	0,899999917	0,002028219
DKFZP434I0714	MAGEA3	DKFZP434I0714\MAGEA3	0,766666651	0,021389991
DKFZP434I0714	MAP10	DKFZP434I0714\MAP10	0,73333329	0,031123236
DKFZP434I0714	MAP3K2	DKFZP434I0714\MAP3K2	0,783333302	0,017223325
DKFZP434I0714	MBLAC2	DKFZP434I0714\MBLAC2	0,833333313	0,008267196
DKFZP434I0714	MCTP2	DKFZP434I0714\MCTP2	0,783333302	0,017223325
DKFZP434I0714	METTL10	DKFZP434I0714\METTL10	0,799999952	0,013828263
DKFZP434I0714	MPP5	DKFZP434I0714\MPP5	0,783333302	0,017223325
DKFZP434I0714	MRPL40	DKFZP434I0714\MRPL40	0,716666639	0,036866181
DKFZP434I0714	MST1L	DKFZP434I0714\MST1L	0,699999928	0,043253969
DKFZP434I0714	MTPAP	DKFZP434I0714\MTPAP	0,966666639	0,000165344
DKFZP434I0714	MYO5B	DKFZP434I0714\MYO5B	0,70293504	0,040145501
DKFZP434I0714	NAA25	DKFZP434I0714\NAA25	0,833333313	0,008267196
DKFZP434I0714	NDRG3	DKFZP434I0714\NDRG3	0,866666615	0,004508378
DKFZP434I0714	NDUFA4	DKFZP434I0714\NDUFA4	0,716666639	0,036866181
DKFZP434I0714	NETO2	DKFZP434I0714\NETO2	0,783333302	0,017223325
DKFZP434I0714	NHLRC3	DKFZP434I0714\NHLRC3	0,766666651	0,021389991

DKFZP434I0714	NPNT	DKFZP434I0714\NPNT	0,74999994	0,025490521
DKFZP434I0714	NSRP1	DKFZP434I0714\NSRP1	0,766666651	0,021389991
DKFZP434I0714	NUDT5	DKFZP434I0714\NUDT5	0,899999917	0,002028219
DKFZP434I0714	NUDT9	DKFZP434I0714\NUDT9	0,799999952	0,013828263
DKFZP434I0714	NVL	DKFZP434I0714\NVL	0,816666603	0,010769401
DKFZP434I0714	OPA1	DKFZP434I0714\OPA1	0,866666615	0,004508378
DKFZP434I0714	PARD6B	DKFZP434I0714\PARD6B	0,73333329	0,031123236
DKFZP434I0714	PCSK6	DKFZP434I0714\PCSK6	0,716666639	0,036866181
DKFZP434I0714	PDGFA	DKFZP434I0714\PDGFA	0,716666639	0,036866181
DKFZP434I0714	PDPK1	DKFZP434I0714\PDPK1	0,74999994	0,025490521
DKFZP434I0714	PFN4	DKFZP434I0714\PFN4	0,849999964	0,006073633
DKFZP434I0714	PGAP1	DKFZP434I0714\PGAP1	0,74999994	0,025490521
DKFZP434I0714	PLA2G12A	DKFZP434I0714\PLA2G12A	0,766666651	0,021389991
DKFZP434I0714	PNMAL1	DKFZP434I0714\PNMAL1	0,833333313	0,008267196
DKFZP434I0714	PNMAL2	DKFZP434I0714\PNMAL2	0,866666615	0,004508378
DKFZP434I0714	POGZ	DKFZP434I0714\POGZ	0,716666639	0,036866181
DKFZP434I0714	POMT2	DKFZP434I0714\POMT2	0,816666603	0,010769401
DKFZP434I0714	POTEE	DKFZP434I0714\POTEE	0,883333266	0,003075397
DKFZP434I0714	POTEF	DKFZP434I0714\POTEF	0,799999952	0,013828263
DKFZP434I0714	POTEM	DKFZP434I0714\POTEM	0,849999964	0,006073633
DKFZP434I0714	PRMT5	DKFZP434I0714\PRMT5	0,799999952	0,013828263
DKFZP434I0714	PRRC1	DKFZP434I0714\PRRC1	0,74999994	0,025490521
DKFZP434I0714	PSAT1	DKFZP434I0714\PSAT1	0,73333329	0,031123236
DKFZP434I0714	PSMC2	DKFZP434I0714\PSMC2	0,716666639	0,036866181
DKFZP434I0714	PSMD12	DKFZP434I0714\PSMD12	0,799999952	0,013828263
DKFZP434I0714	PTPRK	DKFZP434I0714\PTPRK	0,816666603	0,010769401
DKFZP434I0714	PTPRT	DKFZP434I0714\PTPRT	0,783333302	0,017223325
DKFZP434I0714	QSER1	DKFZP434I0714\QSER1	0,716666639	0,036866181
DKFZP434I0714	RAB23	DKFZP434I0714\RAB23	0,799999952	0,013828263
DKFZP434I0714	RAB40C	DKFZP434I0714\RAB40C	0,74999994	0,025490521
DKFZP434I0714	RABGAP1	DKFZP434I0714\RABGAP1	0,783333302	0,017223325
DKFZP434I0714	RAD50	DKFZP434I0714\RAD50	0,783333302	0,017223325
DKFZP434I0714	RAI1	DKFZP434I0714\RAI1	0,878668785	0,003218695
DKFZP434I0714	RANBP17	DKFZP434I0714\RANBP17	0,74999994	0,025490521

DKFZP434I0714	RAVER2	DKFZP434I0714\RAVER2	0,833333313	0,008267196
DKFZP434I0714	RBM23	DKFZP434I0714\RBM23	0,766666651	0,021389991
DKFZP434I0714	RFESD	DKFZP434I0714\RFESD	0,699999928	0,043253969
DKFZP434I0714	RGS9	DKFZP434I0714\RGS9	0,833333313	0,008267196
DKFZP434I0714	RNF144B	DKFZP434I0714\RNF144B	0,766666651	0,021389991
DKFZP434I0714	RNF212	DKFZP434I0714\RNF212	0,73333329	0,031123236
DKFZP434I0714	RNF32	DKFZP434I0714\RNF32	0,816666603	0,010769401
DKFZP434I0714	RPGRIP1L	DKFZP434I0714\RPGRIP1L	0,899999917	0,002028219
DKFZP434I0714	RPS6KA6	DKFZP434I0714\RPS6KA6	0,816666603	0,010769401
DKFZP434I0714	RRAGB	DKFZP434I0714\RRAGB	0,799999952	0,013828263
DKFZP434I0714	SARS2	DKFZP434I0714\SARS2	0,728039861	0,031283069
DKFZP434I0714	SCAMP1	DKFZP434I0714\SCAMP1	0,783333302	0,017223325
DKFZP434I0714	SCCPDH	DKFZP434I0714\SCCPDH	0,866666615	0,004508378
DKFZP434I0714	SDR42E1	DKFZP434I0714\SDR42E1	0,73333329	0,031123236
DKFZP434I0714	SEC61A1	DKFZP434I0714\SEC61A1	0,74999994	0,025490521
DKFZP434I0714	SERBP1	DKFZP434I0714\SERBP1	0,933333278	0,000749559
DKFZP434I0714	SF3B3	DKFZP434I0714\SF3B3	0,73333329	0,031123236
DKFZP434I0714	SIX4	DKFZP434I0714\SIX4	0,716666639	0,036866181
DKFZP434I0714	SLC15A1	DKFZP434I0714\SLC15A1	0,91214186	0,001322751
DKFZP434I0714	SLC30A6	DKFZP434I0714\SLC30A6	0,899999917	0,002028219
DKFZP434I0714	SLCO5A1	DKFZP434I0714\SLCO5A1	0,716666639	0,036866181
DKFZP434I0714	SMO	DKFZP434I0714\SMO	0,816666603	0,010769401
DKFZP434I0714	SNX1	DKFZP434I0714\SNX1	0,699999928	0,043253969
DKFZP434I0714	SOGA2	DKFZP434I0714\SOGA2	0,716666639	0,036866181
DKFZP434I0714	SORCS1	DKFZP434I0714\SORCS1	0,74999994	0,025490521
DKFZP434I0714	SPATA2	DKFZP434I0714\SPATA2	0,73333329	0,031123236
DKFZP434I0714	SPIN1	DKFZP434I0714\SPIN1	0,716666639	0,036866181
DKFZP434I0714	ST7L	DKFZP434I0714\ST7L	0,899999917	0,002028219
DKFZP434I0714	STK36	DKFZP434I0714\STK36	0,833333313	0,008267196
DKFZP434I0714	STX6	DKFZP434I0714\STX6	0,716666639	0,036866181
DKFZP434I0714	SUPT3H	DKFZP434I0714\SUPT3H	0,736408114	0,02800926
DKFZP434I0714	SUSD4	DKFZP434I0714\SUSD4	0,716666639	0,036866181
DKFZP434I0714	SYT1	DKFZP434I0714\SYT1	0,73333329	0,031123236
DKFZP434I0714	TCEB3	DKFZP434I0714\TCEB3	0,74999994	0,025490521

DKFZP434I0714	THAP9	DKFZP434I0714\THAP9	0,783333302	0,017223325
DKFZP434I0714	THSD7B	DKFZP434I0714\THSD7B	0,866666615	0,004508378
DKFZP434I0714	TMEM133	DKFZP434I0714\TMEM133	0,73333329	0,031123236
DKFZP434I0714	TMEM180	DKFZP434I0714\TMEM180	0,762821436	0,021329366
DKFZP434I0714	TMEM26	DKFZP434I0714\TMEM26	0,966666639	0,000165344
DKFZP434I0714	TOMM34	DKFZP434I0714\TOMM34	0,833333313	0,008267196
DKFZP434I0714	TRIM61	DKFZP434I0714\TRIM61	0,799999952	0,013828263
DKFZP434I0714	TRMT10B	DKFZP434I0714\TRMT10B	0,833333313	0,008267196
DKFZP434I0714	TRMT5	DKFZP434I0714\TRMT5	0,933333278	0,000749559
DKFZP434I0714	TRUB1	DKFZP434I0714\TRUB1	0,74999994	0,025490521
DKFZP434I0714	TTC26	DKFZP434I0714\TTC26	0,866666615	0,004508378
DKFZP434I0714	TTYH2	DKFZP434I0714\TTYH2	0,699999928	0,043253969
DKFZP434I0714	TXNL1	DKFZP434I0714\TXNL1	0,74999994	0,025490521
DKFZP434I0714	TYW5	DKFZP434I0714\TYW5	0,916666567	0,001311728
DKFZP434I0714	UBE3A	DKFZP434I0714\UBE3A	0,883333266	0,003075397
DKFZP434I0714	UBFD1	DKFZP434I0714\UBFD1	0,73333329	0,031123236
DKFZP434I0714	UBTD2	DKFZP434I0714\UBTD2	0,736408114	0,02800926
DKFZP434I0714	UEVLD	DKFZP434I0714\UEVLD	0,898434162	0,002050265
DKFZP434I0714	UMPS	DKFZP434I0714\UMPS	0,728918254	0,031679895
DKFZP434I0714	URB2	DKFZP434I0714\URB2	0,899999917	0,002028219
DKFZP434I0714	USP46	DKFZP434I0714\USP46	0,73333329	0,031123236
DKFZP434I0714	VPS35	DKFZP434I0714\VPS35	0,949999988	0,000352734
DKFZP434I0714	WDR35	DKFZP434I0714\WDR35	0,883333266	0,003075397
DKFZP434I0714	WDR91	DKFZP434I0714\WDR91	0,74999994	0,025490521
DKFZP434I0714	XPO7	DKFZP434I0714\XPO7	0,916666567	0,001311728
DKFZP434I0714	ZC3HC1	DKFZP434I0714\ZC3HC1	0,816666603	0,010769401
DKFZP434I0714	ZMYM3	DKFZP434I0714\ZMYM3	0,74999994	0,025490521
DKFZP434I0714	ZMYM6	DKFZP434I0714\ZMYM6	0,783333302	0,017223325
DKFZP434I0714	ZNF211	DKFZP434I0714\ZNF211	0,74999994	0,025490521
DKFZP434I0714	ZNF221	DKFZP434I0714\ZNF221	0,816666603	0,010769401
DKFZP434I0714	ZNF280B	DKFZP434I0714\ZNF280B	0,699999928	0,043253969
DKFZP434I0714	ZNF540	DKFZP434I0714\ZNF540	0,73333329	0,031123236
DKFZP434I0714	ZNF543	DKFZP434I0714\ZNF543	0,816666603	0,010769401
DKFZP434I0714	ZNF544	DKFZP434I0714\ZNF544	0,866666615	0,004508378

DKFZP434I0714	ZNF554	DKFZP434I0714\ZNF554	0,796724617	0,013227513
DKFZP434I0714	ZNF558	DKFZP434I0714\ZNF558	0,799999952	0,013828263
DKFZP434I0714	ZNF562	DKFZP434I0714\ZNF562	0,833333313	0,008267196
DKFZP434I0714	ZNF572	DKFZP434I0714\ZNF572	0,98333329	4,96032E-05
DKFZP434I0714	ZNF782	DKFZP434I0714\ZNF782	0,920510173	0,000981041
DKFZP434I0714	ZRANB3	DKFZP434I0714\ZRANB3	0,916666567	0,001311728
DKFZP434I0714	ZSCAN31	DKFZP434I0714\ZSCAN31	0,899999917	0,002028219
FAM66E	AJUBA	FAM66E\AJUBA	0,799999952	0,013828263
FAM66E	ALDH7A1	FAM66E\ALDH7A1	0,716666639	0,036866181
FAM66E	ALS2	FAM66E\ALS2	0,766666651	0,021389991
FAM66E	ANK3	FAM66E\ANK3	0,866666615	0,004508378
FAM66E	AP2B1	FAM66E\AP2B1	0,816666603	0,010769401
FAM66E	APOBEC2	FAM66E\APOBEC2	0,766666651	0,021389991
FAM66E	ARL6IP1	FAM66E\ARL6IP1	0,716666639	0,036866181
FAM66E	ATP6AP2	FAM66E\ATP6AP2	0,766666651	0,021389991
FAM66E	ATP6V0A1	FAM66E\ATP6V0A1	0,711966693	0,037896827
FAM66E	ATP6V0D1	FAM66E\ATP6V0D1	0,799999952	0,013828263
FAM66E	AUTS2	FAM66E\AUTS2	0,766666651	0,021389991
FAM66E	BBS4	FAM66E\BBS4	0,716666639	0,036866181
FAM66E	BRD1	FAM66E\BRD1	0,716666639	0,036866181
FAM66E	BTBD3	FAM66E\BTBD3	0,699999928	0,043253969
FAM66E	C15orf41	FAM66E\C15orf41	0,699999928	0,043253969
FAM66E	C4A	FAM66E\C4A	0,716666639	0,036866181
FAM66E	C4B	FAM66E\C4B	0,716666639	0,036866181
FAM66E	CBS	FAM66E\CBS	0,799999952	0,013828263
FAM66E	CCDC122	FAM66E\CCDC122	0,73333329	0,031123236
FAM66E	CCDC148	FAM66E\CCDC148	0,74999994	0,025490521
FAM66E	CCDC30	FAM66E\CCDC30	0,833333313	0,008267196
FAM66E	CCDC8	FAM66E\CCDC8	0,833333313	0,008267196
FAM66E	CDH1	FAM66E\CDH1	0,916666567	0,001311728
FAM66E	CDKL3	FAM66E\CDKL3	0,916666567	0,001311728
FAM66E	CDS1	FAM66E\CDS1	0,783333302	0,017223325
FAM66E	CETN3	FAM66E\CETN3	0,783333302	0,017223325
FAM66E	CLDN12	FAM66E\CLDN12	0,849999964	0,006073633

FAM66E	CLTC	FAM66E\CLTC	0,766666651	0,021389991
FAM66E	CNTNAP3	FAM66E\CNTNAP3	0,783333302	0,017223325
FAM66E	CNTNAP3B	FAM66E\CNTNAP3B	0,849999964	0,006073633
FAM66E	COG5	FAM66E\COG5	0,73333329	0,031123236
FAM66E	CYB5B	FAM66E\CYB5B	0,699999928	0,043253969
FAM66E	DDX31	FAM66E\DDX31	0,783333302	0,017223325
FAM66E	DENND1A	FAM66E\DENND1A	0,866666615	0,004508378
FAM66E	DET1	FAM66E\DET1	0,74999994	0,025490521
FAM66E	DHTKD1	FAM66E\DHTKD1	0,766666651	0,021389991
FAM66E	DHX32	FAM66E\DHX32	0,916666567	0,001311728
FAM66E	DMRT2	FAM66E\DMRT2	0,849999964	0,006073633
FAM66E	DMRTA1	FAM66E\DMRTA1	0,833333313	0,008267196
FAM66E	DNAJC16	FAM66E\DNAJC16	0,699999928	0,043253969
FAM66E	DNAL4	FAM66E\DNAL4	0,966666639	0,000165344
FAM66E	DPH6	FAM66E\DPH6	0,899999917	0,002028219
FAM66E	DUOX1	FAM66E\DUOX1	0,736408114	0,02800926
FAM66E	DUOXA1	FAM66E\DUOXA1	0,783333302	0,017223325
FAM66E	EDA2R	FAM66E\EDA2R	0,716666639	0,036866181
FAM66E	EIF2AK4	FAM66E\EIF2AK4	0,74999994	0,025490521
FAM66E	ENPEP	FAM66E\ENPEP	0,766666651	0,021389991
FAM66E	EPN3	FAM66E\EPN3	0,816666603	0,010769401
FAM66E	EPS15L1	FAM66E\EPS15L1	0,74999994	0,025490521
FAM66E	ESRP1	FAM66E\ESRP1	0,799999952	0,013828263
FAM66E	EYA2	FAM66E\EYA2	0,866666615	0,004508378
FAM66E	FAHD1	FAM66E\FAHD1	0,899999917	0,002028219
FAM66E	FAM154B	FAM66E\FAM154B	0,881482542	0,003009259
FAM66E	FAM160A1	FAM66E\FAM160A1	0,799999952	0,013828263
FAM66E	FAM199X	FAM66E\FAM199X	0,73333329	0,031123236
FAM66E	FAM218A	FAM66E\FAM218A	0,766666651	0,021389991
FAM66E	FBXL16	FAM66E\FBXL16	0,783333302	0,017223325
FAM66E	FEM1B	FAM66E\FEM1B	0,766666651	0,021389991
FAM66E	FKTN	FAM66E\FKTN	0,699999928	0,043253969
FAM66E	FNBP1L	FAM66E\FNBP1L	0,73333329	0,031123236
FAM66E	FOXE1	FAM66E\FOXE1	0,799999952	0,013828263

FAM66E	GGA1	FAM66E\GGA1	0,783333302	0,017223325
FAM66E	GGCT	FAM66E\GGCT	0,73333329	0,031123236
FAM66E	GLI2	FAM66E\GLI2	0,74999994	0,025490521
FAM66E	GNPDA1	FAM66E\GNPDA1	0,73333329	0,031123236
FAM66E	GRHL2	FAM66E\GRHL2	0,699999928	0,043253969
FAM66E	GRIP1	FAM66E\GRIP1	0,916666567	0,001311728
FAM66E	GRTP1	FAM66E\GRTP1	0,816666603	0,010769401
FAM66E	GTF3C4	FAM66E\GTF3C4	0,816666603	0,010769401
FAM66E	HIST2H2BF	FAM66E\HIST2H2BF	0,73333329	0,031123236
FAM66E	HN1L	FAM66E\HN1L	0,766666651	0,021389991
FAM66E	HOMER2	FAM66E\HOMER2	0,74999994	0,025490521
FAM66E	HOMER3	FAM66E\HOMER3	0,883333266	0,003075397
FAM66E	HPSE	FAM66E\HPSE	0,866666615	0,004508378
FAM66E	IFI44	FAM66E\IFI44	0,866666615	0,004508378
FAM66E	IGSF3	FAM66E\IGSF3	0,766666651	0,021389991
FAM66E	IQCH	FAM66E\IQCH	0,73333329	0,031123236
FAM66E	IRF2BP2	FAM66E\IRF2BP2	0,716666639	0,036866181
FAM66E	ITGA2	FAM66E\ITGA2	0,816666603	0,010769401
FAM66E	KDM4D	FAM66E\KDM4D	0,719671547	0,033763226
FAM66E	KLHDC10	FAM66E\KLHDC10	0,816666603	0,010769401
FAM66E	KRTAP5-8	FAM66E\KRTAP5-8	0,73333329	0,031123236
FAM66E	LACC1	FAM66E\LACC1	0,744776368	0,026047178
FAM66E	LARS	FAM66E\LARS	0,74999994	0,025490521
FAM66E	LEPREL1	FAM66E\LEPREL1	0,883333266	0,003075397
FAM66E	LIMK1	FAM66E\LIMK1	0,736408114	0,02800926
FAM66E	LRIG3	FAM66E\LRIG3	0,866666615	0,004508378
FAM66E	LRPPRC	FAM66E\LRPPRC	0,73333329	0,031123236
FAM66E	M1AP	FAM66E\M1AP	0,833333313	0,008267196
FAM66E	MAGEA3	FAM66E\MAGEA3	0,716666639	0,036866181
FAM66E	MAML3	FAM66E\MAML3	0,716666639	0,036866181
FAM66E	MAP3K2	FAM66E\MAP3K2	0,699999928	0,043253969
FAM66E	MARVELD2	FAM66E\MARVELD2	0,883333266	0,003075397
FAM66E	MCOLN3	FAM66E\MCOLN3	0,73333329	0,031123236
FAM66E	MCTP2	FAM66E\MCTP2	0,833333313	0,008267196

FAM66E	MFSD3	FAM66E\MFSD3	0,73333329	0,031123236
FAM66E	MPP5	FAM66E\MPP5	0,766666651	0,021389991
FAM66E	MRPL40	FAM66E\MRPL40	0,833333313	0,008267196
FAM66E	MYO10	FAM66E\MYO10	0,766666651	0,021389991
FAM66E	N4BP1	FAM66E\N4BP1	0,799999952	0,013828263
FAM66E	NAGK	FAM66E\NAGK	0,899999917	0,002028219
FAM66E	NCS1	FAM66E\NCS1	0,816666603	0,010769401
FAM66E	NDUFA4	FAM66E\NDUFA4	0,73333329	0,031123236
FAM66E	NEO1	FAM66E\NEO1	0,799999952	0,013828263
FAM66E	NETO2	FAM66E\NETO2	0,766666651	0,021389991
FAM66E	NHLRC3	FAM66E\NHLRC3	0,849999964	0,006073633
FAM66E	NPNT	FAM66E\NPNT	0,866666615	0,004508378
FAM66E	NRCAM	FAM66E\NRCAM	0,866666615	0,004508378
FAM66E	NUDT12	FAM66E\NUDT12	0,761512935	0,021263227
FAM66E	NXN	FAM66E\NXN	0,778249502	0,017383156
FAM66E	PABPC4L	FAM66E\PABPC4L	0,933333278	0,000749559
FAM66E	PANK1	FAM66E\PANK1	0,74999994	0,025490521
FAM66E	PAX1	FAM66E\PAX1	0,73333329	0,031123236
FAM66E	PAX9	FAM66E\PAX9	0,866666615	0,004508378
FAM66E	PDGFA	FAM66E\PDGFA	0,699999928	0,043253969
FAM66E	PGAP1	FAM66E\PGAP1	0,766666651	0,021389991
FAM66E	PKP4	FAM66E\PKP4	0,916666567	0,001311728
FAM66E	PLA2G12A	FAM66E\PLA2G12A	0,916666567	0,001311728
FAM66E	PLD2	FAM66E\PLD2	0,816666603	0,010769401
FAM66E	PMPCB	FAM66E\PMPCB	0,74999994	0,025490521
FAM66E	PNMAL2	FAM66E\PNMAL2	0,816666603	0,010769401
FAM66E	POMT2	FAM66E\POMT2	0,799999952	0,013828263
FAM66E	PRKAR1A	FAM66E\PRKAR1A	0,898434162	0,002050265
FAM66E	PRRC1	FAM66E\PRRC1	0,74999994	0,025490521
FAM66E	PRSS8	FAM66E\PRSS8	0,73333329	0,031123236
FAM66E	PSD3	FAM66E\PSD3	0,799999952	0,013828263
FAM66E	PSMD12	FAM66E\PSMD12	0,716666639	0,036866181
FAM66E	PTPRT	FAM66E\PTPRT	0,699999928	0,043253969
FAM66E	RAB14	FAM66E\RAB14	0,899999917	0,002028219

FAM66E	RAB23	FAM66E\RAB23	0,883333266	0,003075397
FAM66E	RABGAP1	FAM66E\RABGAP1	0,699999928	0,043253969
FAM66E	RAD50	FAM66E\RAD50	0,699999928	0,043253969
FAM66E	RANBP17	FAM66E\RANBP17	0,74999994	0,025490521
FAM66E	RMDN3	FAM66E\RMDN3	0,79498601	0,013833774
FAM66E	RNF170	FAM66E\RNF170	0,799999952	0,013828263
FAM66E	RPGRIP1L	FAM66E\RPGRIP1L	0,716666639	0,036866181
FAM66E	RPS6KA6	FAM66E\RPS6KA6	0,766666651	0,021389991
FAM66E	RRM2B	FAM66E\RRM2B	0,816666603	0,010769401
FAM66E	RTF1	FAM66E\RTF1	0,699999928	0,043253969
FAM66E	SDR42E1	FAM66E\SDR42E1	0,883333266	0,003075397
FAM66E	SGPL1	FAM66E\SGPL1	0,716666639	0,036866181
FAM66E	SIX1	FAM66E\SIX1	0,883333266	0,003075397
FAM66E	SIX4	FAM66E\SIX4	0,73333329	0,031123236
FAM66E	SLC22A5	FAM66E\SLC22A5	0,833333313	0,008267196
FAM66E	SLC30A1	FAM66E\SLC30A1	0,966666639	0,000165344
FAM66E	SLC46A1	FAM66E\SLC46A1	0,73333329	0,031123236
FAM66E	SMO	FAM66E\SMO	0,766666651	0,021389991
FAM66E	SNUPN	FAM66E\SNUPN	0,73333329	0,031123236
FAM66E	SORBS2	FAM66E\SORBS2	0,783333302	0,017223325
FAM66E	SORCS2	FAM66E\SORCS2	0,74999994	0,025490521
FAM66E	SPATA6	FAM66E\SPATA6	0,866666615	0,004508378
FAM66E	SPIN1	FAM66E\SPIN1	0,699999928	0,043253969
FAM66E	SPINT1	FAM66E\SPINT1	0,816666603	0,010769401
FAM66E	SPIRE2	FAM66E\SPIRE2	0,753144681	0,023533951
FAM66E	STARD7	FAM66E\STARD7	0,699999928	0,043253969
FAM66E	STEAP2	FAM66E\STEAP2	0,916666567	0,001311728
FAM66E	SUPT3H	FAM66E\SUPT3H	0,686198473	0,046968695
FAM66E	SYBU	FAM66E\SYBU	0,716666639	0,036866181
FAM66E	SYT1	FAM66E\SYT1	0,73333329	0,031123236
FAM66E	TDGF1	FAM66E\TDGF1	0,799999952	0,013828263
FAM66E	TMEM129	FAM66E\TMEM129	0,728039861	0,031283069
FAM66E	TMEM180	FAM66E\TMEM180	0,711966693	0,038029101
FAM66E	TMEM185A	FAM66E\TMEM185A	0,699999928	0,043253969

FAM66E	TMEM30B	FAM66E\TMEM30B	0,73333329	0,031123236
FAM66E	TP63	FAM66E\TP63	0,73333329	0,031123236
FAM66E	TRMT10B	FAM66E\TRMT10B	0,74999994	0,025490521
FAM66E	TRMT5	FAM66E\TRMT5	0,716666639	0,036866181
FAM66E	TRPM7	FAM66E\TRPM7	0,699999928	0,043253969
FAM66E	TSPAN13	FAM66E\TSPAN13	0,766666651	0,021389991
FAM66E	TTC22	FAM66E\TTC22	0,883333266	0,003075397
FAM66E	TYW5	FAM66E\TYW5	0,699999928	0,043253969
FAM66E	UBAC1	FAM66E\UBAC1	0,716666639	0,036866181
FAM66E	UBE2V2	FAM66E\UBE2V2	0,79498601	0,014252646
FAM66E	UBTD2	FAM66E\UBTD2	0,769881189	0,018992504
FAM66E	UEVLD	FAM66E\UEVLD	0,779773057	0,016765874
FAM66E	UNC5B	FAM66E\UNC5B	0,73333329	0,031123236
FAM66E	USP46	FAM66E\USP46	0,849999964	0,006073633
FAM66E	WASL	FAM66E\WASL	0,833333313	0,008267196
FAM66E	WDR91	FAM66E\WDR91	0,766666651	0,021389991
FAM66E	WTH3DI	FAM66E\WTH3DI	0,833333313	0,008267196
FAM66E	XPR1	FAM66E\XPR1	0,73333329	0,031123236
FAM66E	ZBTB41	FAM66E\ZBTB41	0,766666651	0,021389991
FAM66E	ZNF280B	FAM66E\ZNF280B	0,916666567	0,001311728
FAM66E	ZNF449	FAM66E\ZNF449	0,828459144	0,008289241
FAM66E	ZNF626	FAM66E\ZNF626	0,716666639	0,036866181
FAM66E	ZSCAN31	FAM66E\ZSCAN31	0,716666639	0,036866181
FAM95B1	ACAD10	FAM95B1\ACAD10	0,70293504	0,040343914
FAM95B1	AJUBA	FAM95B1\AJUBA	0,799999952	0,013828263
FAM95B1	ALDH7A1	FAM95B1\ALDH7A1	0,699999928	0,043253969
FAM95B1	ANK3	FAM95B1\ANK3	0,883333266	0,003075397
FAM95B1	ANKFY1	FAM95B1\ANKFY1	0,699999928	0,043253969
FAM95B1	AP1S1	FAM95B1\AP1S1	0,933333278	0,000749559
FAM95B1	AP2B1	FAM95B1\AP2B1	0,816666603	0,010769401
FAM95B1	APOBEC2	FAM95B1\APOBEC2	0,966666639	0,000165344
FAM95B1	APTX	FAM95B1\APTX	0,73333329	0,031123236
FAM95B1	ARL6IP1	FAM95B1\ARL6IP1	0,833333313	0,008267196
FAM95B1	ARV1	FAM95B1\ARV1	0,74999994	0,025490521

FAM95B1	ATP2C1	FAM95B1\ATP2C1	0,716666639	0,036866181
FAM95B1	ATP6AP2	FAM95B1\ATP6AP2	0,799999952	0,013828263
FAM95B1	ATP6V0B	FAM95B1\ATP6V0B	0,849999964	0,006073633
FAM95B1	ATP6V0D1	FAM95B1\ATP6V0D1	0,73333329	0,031123236
FAM95B1	ATP6V1A	FAM95B1\ATP6V1A	0,699999928	0,043253969
FAM95B1	ATP6V1C1	FAM95B1\ATP6V1C1	0,833333313	0,008267196
FAM95B1	ATRNL1	FAM95B1\ATRNL1	0,849999964	0,006073633
FAM95B1	AUTS2	FAM95B1\AUTS2	0,699999928	0,043253969
FAM95B1	BBS4	FAM95B1\BBS4	0,816666603	0,010769401
FAM95B1	BRD1	FAM95B1\BRD1	0,866666615	0,004508378
FAM95B1	C15orf41	FAM95B1\C15orf41	0,883333266	0,003075397
FAM95B1	C19orf54	FAM95B1\C19orf54	0,766666651	0,021389991
FAM95B1	C19orf82	FAM95B1\C19orf82	0,866666615	0,004508378
FAM95B1	C1orf109	FAM95B1\C1orf109	0,799999952	0,013828263
FAM95B1	CAMSAP1	FAM95B1\CAMSAP1	0,849999964	0,006073633
FAM95B1	CBLN3	FAM95B1\CBLN3	0,816666603	0,010769401
FAM95B1	CBS	FAM95B1\CBS	0,766666651	0,021389991
FAM95B1	CCDC30	FAM95B1\CCDC30	0,766666651	0,021389991
FAM95B1	CCDC8	FAM95B1\CCDC8	0,816666603	0,010769401
FAM95B1	CCL25	FAM95B1\CCL25	0,73333329	0,031123236
FAM95B1	CDH1	FAM95B1\CDH1	0,883333266	0,003075397
FAM95B1	CDIP1	FAM95B1\CDIP1	0,716666639	0,036866181
FAM95B1	CDKL3	FAM95B1\CDKL3	0,799999952	0,013828263
FAM95B1	CDS1	FAM95B1\CDS1	0,849999964	0,006073633
FAM95B1	CETN3	FAM95B1\CETN3	0,799999952	0,013828263
FAM95B1	CLDN12	FAM95B1\CLDN12	0,966666639	0,000165344
FAM95B1	CLINT1	FAM95B1\CLINT1	0,716666639	0,036866181
FAM95B1	CLTC	FAM95B1\CLTC	0,783333302	0,017223325
FAM95B1	CNTNAP3	FAM95B1\CNTNAP3	0,783333302	0,017223325
FAM95B1	CNTNAP3B	FAM95B1\CNTNAP3B	0,866666615	0,004508378
FAM95B1	COG5	FAM95B1\COG5	0,849999964	0,006073633
FAM95B1	CROT	FAM95B1\CROT	0,849999964	0,006073633
FAM95B1	CRTC1	FAM95B1\CRTC1	0,699999928	0,043253969
FAM95B1	CUX1	FAM95B1\CUX1	0,816666603	0,010769401

FAM95B1	DCAKD	FAM95B1\DCAKD	0,816666603	0,010769401
FAM95B1	DDX31	FAM95B1\DDX31	0,74999994	0,025490521
FAM95B1	DENND1A	FAM95B1\DENND1A	0,799999952	0,013828263
FAM95B1	DET1	FAM95B1\DET1	0,816666603	0,010769401
FAM95B1	DHTKD1	FAM95B1\DHTKD1	0,866666615	0,004508378
FAM95B1	DHX32	FAM95B1\DHX32	0,783333302	0,017223325
FAM95B1	DMRT2	FAM95B1\DMRT2	0,866666615	0,004508378
FAM95B1	DMRTA1	FAM95B1\DMRTA1	0,73333329	0,031123236
FAM95B1	DNAJC16	FAM95B1\DNAJC16	0,866666615	0,004508378
FAM95B1	DNAL4	FAM95B1\DNAL4	0,799999952	0,013828263
FAM95B1	DPH6	FAM95B1\DPH6	0,949999988	0,000352734
FAM95B1	DUOX1	FAM95B1\DUOX1	0,953983247	0,000253527
FAM95B1	DUOXA1	FAM95B1\DUOXA1	0,883333266	0,003075397
FAM95B1	ELOVL6	FAM95B1\ELOVL6	0,783333302	0,017223325
FAM95B1	ENPEP	FAM95B1\ENPEP	0,766666651	0,021389991
FAM95B1	EPN3	FAM95B1\EPN3	0,916666567	0,001311728
FAM95B1	EPS15L1	FAM95B1\EPS15L1	0,866666615	0,004508378
FAM95B1	ESRP1	FAM95B1\ESRP1	0,849999964	0,006073633
FAM95B1	ESRP2	FAM95B1\ESRP2	0,833333313	0,008267196
FAM95B1	EVA1A	FAM95B1\EVA1A	0,833333313	0,008267196
FAM95B1	EXOC7	FAM95B1\EXOC7	0,711966693	0,038029101
FAM95B1	EYA2	FAM95B1\EYA2	0,866666615	0,004508378
FAM95B1	FAHD1	FAM95B1\FAHD1	0,916666567	0,001311728
FAM95B1	FAM120A	FAM95B1\FAM120A	0,73333329	0,031123236
FAM95B1	FAM154B	FAM95B1\FAM154B	0,813676238	0,010449735
FAM95B1	FAM199X	FAM95B1\FAM199X	0,816666603	0,010769401
FAM95B1	FAM218A	FAM95B1\FAM218A	0,73333329	0,031123236
FAM95B1	FAM83B	FAM95B1\FAM83B	0,699999928	0,043253969
FAM95B1	FBXO22	FAM95B1\FBXO22	0,728039861	0,031283069
FAM95B1	FEM1B	FAM95B1\FEM1B	0,799999952	0,013828263
FAM95B1	FKTN	FAM95B1\FKTN	0,833333313	0,008267196
FAM95B1	FLVCR1	FAM95B1\FLVCR1	0,73333329	0,031123236
FAM95B1	FOXE1	FAM95B1\FOXE1	0,766666651	0,021389991
FAM95B1	FSTL4	FAM95B1\FSTL4	0,816666603	0,010769401

FAM95B1	GGA1	FAM95B1\GGA1	0,883333266	0,003075397
FAM95B1	GGA2	FAM95B1\GGA2	0,866666615	0,004508378
FAM95B1	GGCT	FAM95B1\GGCT	0,74999994	0,025490521
FAM95B1	GLI2	FAM95B1\GLI2	0,899999917	0,002028219
FAM95B1	GNPDA1	FAM95B1\GNPDA1	0,766666651	0,021389991
FAM95B1	GPHN	FAM95B1\GPHN	0,699999928	0,043253969
FAM95B1	GPR107	FAM95B1\GPR107	0,695015132	0,044576719
FAM95B1	GPS1	FAM95B1\GPS1	0,783333302	0,017223325
FAM95B1	GRHL2	FAM95B1\GRHL2	0,74999994	0,025490521
FAM95B1	GRIP1	FAM95B1\GRIP1	0,783333302	0,017223325
FAM95B1	GRTP1	FAM95B1\GRTP1	0,833333313	0,008267196
FAM95B1	GTF2I	FAM95B1\GTF2I	0,816666603	0,010769401
FAM95B1	GTF3C4	FAM95B1\GTF3C4	0,849999964	0,006073633
FAM95B1	HIST2H2BF	FAM95B1\HIST2H2BF	0,866666615	0,004508378
FAM95B1	HN1L	FAM95B1\HN1L	0,883333266	0,003075397
FAM95B1	HOMER2	FAM95B1\HOMER2	0,816666603	0,010769401
FAM95B1	HOMER3	FAM95B1\HOMER3	0,716666639	0,036866181
FAM95B1	HPS5	FAM95B1\HPS5	0,799999952	0,013828263
FAM95B1	HPSE	FAM95B1\HPSE	0,833333313	0,008267196
FAM95B1	IDH3G	FAM95B1\IDH3G	0,849999964	0,006073633
FAM95B1	IFI44	FAM95B1\IFI44	0,716666639	0,036866181
FAM95B1	IGSF3	FAM95B1\IGSF3	0,766666651	0,021389991
FAM95B1	IQCH	FAM95B1\IQCH	0,74999994	0,025490521
FAM95B1	ITGA2	FAM95B1\ITGA2	0,833333313	0,008267196
FAM95B1	KDM4D	FAM95B1\KDM4D	0,920510173	0,000981041
FAM95B1	KIAA0319L	FAM95B1\KIAA0319L	0,766666651	0,021389991
FAM95B1	KIAA1549	FAM95B1\KIAA1549	0,833333313	0,008267196
FAM95B1	KLHDC10	FAM95B1\KLHDC10	0,833333313	0,008267196
FAM95B1	KRTAP5-8	FAM95B1\KRTAP5-8	0,73333329	0,031123236
FAM95B1	LAMP1	FAM95B1\LAMP1	0,82009083	0,009281305
FAM95B1	LAMP2	FAM95B1\LAMP2	0,845195651	0,006205908
FAM95B1	LARS	FAM95B1\LARS	0,799999952	0,013828263
FAM95B1	LIMK1	FAM95B1\LIMK1	0,861932218	0,004376102
FAM95B1	LMBR1	FAM95B1\LMBR1	0,866666615	0,004508378

FAM95B1	LRIG3	FAM95B1\LRIG3	0,766666651	0,021389991
FAM95B1	LRPPRC	FAM95B1\LRPPRC	0,74999994	0,025490521
FAM95B1	LZIC	FAM95B1\LZIC	0,716666639	0,036866181
FAM95B1	M1AP	FAM95B1\M1AP	0,916666567	0,001311728
FAM95B1	MAML3	FAM95B1\MAML3	0,899999917	0,002028219
FAM95B1	MAP3K2	FAM95B1\MAP3K2	0,833333313	0,008267196
FAM95B1	MARVELD2	FAM95B1\MARVELD2	0,883333266	0,003075397
FAM95B1	MAST4	FAM95B1\MAST4	0,686198473	0,046968695
FAM95B1	MCOLN3	FAM95B1\MCOLN3	0,883333266	0,003075397
FAM95B1	MCTP2	FAM95B1\MCTP2	0,74999994	0,025490521
FAM95B1	MED22	FAM95B1\MED22	0,762821436	0,022123016
FAM95B1	MFSD3	FAM95B1\MFSD3	0,866666615	0,004508378
FAM95B1	MOV10	FAM95B1\MOV10	0,716666639	0,036866181
FAM95B1	MPP5	FAM95B1\MPP5	0,799999952	0,013828263
FAM95B1	MTPN	FAM95B1\MTPN	0,694566727	0,043816138
FAM95B1	MTSS1L	FAM95B1\MTSS1L	0,799999952	0,013828263
FAM95B1	MYO10	FAM95B1\MYO10	0,866666615	0,004508378
FAM95B1	N4BP1	FAM95B1\N4BP1	0,966666639	0,000165344
FAM95B1	NAA50	FAM95B1\NAA50	0,766666651	0,021389991
FAM95B1	NAGK	FAM95B1\NAGK	0,933333278	0,000749559
FAM95B1	NCS1	FAM95B1\NCS1	0,849999964	0,006073633
FAM95B1	NDUFA4	FAM95B1\NDUFA4	0,866666615	0,004508378
FAM95B1	NDUFB5	FAM95B1\NDUFB5	0,73333329	0,031123236
FAM95B1	NETO2	FAM95B1\NETO2	0,849999964	0,006073633
FAM95B1	NHLRC3	FAM95B1\NHLRC3	0,766666651	0,021389991
FAM95B1	NIPSNAP1	FAM95B1\NIPSNAP1	0,816666603	0,010769401
FAM95B1	NPNT	FAM95B1\NPNT	0,799999952	0,013828263
FAM95B1	NRCAM	FAM95B1\NRCAM	0,933333278	0,000749559
FAM95B1	NUDT12	FAM95B1\NUDT12	0,836827397	0,00696649
FAM95B1	NXN	FAM95B1\NXN	0,803354323	0,012202381
FAM95B1	OSBPL3	FAM95B1\OSBPL3	0,883333266	0,003075397
FAM95B1	OXCT1	FAM95B1\OXCT1	0,883333266	0,003075397
FAM95B1	PABPC4L	FAM95B1\PABPC4L	0,833333313	0,008267196
FAM95B1	PANK1	FAM95B1\PANK1	0,916666567	0,001311728

FAM95B1	PASK	FAM95B1\PASK	0,799999952	0,013828263
FAM95B1	PAX1	FAM95B1\PAX1	0,73333329	0,031123236
FAM95B1	PAX9	FAM95B1\PAX9	0,883333266	0,003075397
FAM95B1	PCCB	FAM95B1\PCCB	0,74999994	0,025490521
FAM95B1	PGAP1	FAM95B1\PGAP1	0,716666639	0,036866181
FAM95B1	PIAS2	FAM95B1\PIAS2	0,783333302	0,017223325
FAM95B1	PIFO	FAM95B1\PIFO	0,833333313	0,008267196
FAM95B1	PKP1	FAM95B1\PKP1	0,816666603	0,010769401
FAM95B1	PKP4	FAM95B1\PKP4	0,866666615	0,004508378
FAM95B1	PLA2G12A	FAM95B1\PLA2G12A	0,866666615	0,004508378
FAM95B1	PLD2	FAM95B1\PLD2	0,849999964	0,006073633
FAM95B1	PLEK2	FAM95B1\PLEK2	0,766666651	0,021389991
FAM95B1	PMPCB	FAM95B1\PMPCB	0,933333278	0,000749559
FAM95B1	PNMAL2	FAM95B1\PNMAL2	0,716666639	0,036866181
FAM95B1	PPP5C	FAM95B1\PPP5C	0,699999928	0,043253969
FAM95B1	PRKAR1A	FAM95B1\PRKAR1A	0,695015132	0,044576719
FAM95B1	PRRC1	FAM95B1\PRRC1	0,816666603	0,010769401
FAM95B1	PRRG4	FAM95B1\PRRG4	0,783333302	0,017223325
FAM95B1	PRSS8	FAM95B1\PRSS8	0,916666567	0,001311728
FAM95B1	PSAT1	FAM95B1\PSAT1	0,716666639	0,036866181
FAM95B1	PSD3	FAM95B1\PSD3	0,883333266	0,003075397
FAM95B1	PSMD12	FAM95B1\PSMD12	0,816666603	0,010769401
FAM95B1	PTPRT	FAM95B1\PTPRT	0,716666639	0,036866181
FAM95B1	RAB14	FAM95B1\RAB14	0,949999988	0,000352734
FAM95B1	RAB23	FAM95B1\RAB23	0,866666615	0,004508378
FAM95B1	RAB40C	FAM95B1\RAB40C	0,766666651	0,021389991
FAM95B1	RAD50	FAM95B1\RAD50	0,833333313	0,008267196
FAM95B1	RASL10A	FAM95B1\RASL10A	0,828459144	0,008289241
FAM95B1	RBM23	FAM95B1\RBM23	0,699999928	0,043253969
FAM95B1	RFESD	FAM95B1\RFESD	0,783333302	0,017223325
FAM95B1	RHBDD2	FAM95B1\RHBDD2	0,799999952	0,013828263
FAM95B1	RMDN3	FAM95B1\RMDN3	0,878668785	0,003218695
FAM95B1	RNF170	FAM95B1\RNF170	0,799999952	0,013828263
FAM95B1	RNF212	FAM95B1\RNF212	0,699999928	0,043253969

FAM95B1	RRAGB	FAM95B1\RRAGB	0,716666639	0,036866181
FAM95B1	RRM2B	FAM95B1\RRM2B	0,699999928	0,043253969
FAM95B1	RTF1	FAM95B1\RTF1	0,866666615	0,004508378
FAM95B1	S100A14	FAM95B1\S100A14	0,849999964	0,006073633
FAM95B1	SCD5	FAM95B1\SCD5	0,74999994	0,025490521
FAM95B1	SDR42E1	FAM95B1\SDR42E1	0,833333313	0,008267196
FAM95B1	SGPL1	FAM95B1\SGPL1	0,866666615	0,004508378
FAM95B1	SGPP2	FAM95B1\SGPP2	0,716666639	0,036866181
FAM95B1	SIX1	FAM95B1\SIX1	0,716666639	0,036866181
FAM95B1	SIX4	FAM95B1\SIX4	0,783333302	0,017223325
FAM95B1	SLC22A5	FAM95B1\SLC22A5	0,849999964	0,006073633
FAM95B1	SLC30A1	FAM95B1\SLC30A1	0,816666603	0,010769401
FAM95B1	SLC30A6	FAM95B1\SLC30A6	0,716666639	0,036866181
FAM95B1	SLC44A3	FAM95B1\SLC44A3	0,933333278	0,000749559
FAM95B1	SLC46A1	FAM95B1\SLC46A1	0,899999917	0,002028219
FAM95B1	SLC6A8	FAM95B1\SLC6A8	0,73333329	0,031123236
FAM95B1	SLCO5A1	FAM95B1\SLCO5A1	0,699999928	0,043253969
FAM95B1	SNX1	FAM95B1\SNX1	0,74999994	0,025490521
FAM95B1	SOGA2	FAM95B1\SOGA2	0,766666651	0,021389991
FAM95B1	SORBS2	FAM95B1\SORBS2	0,933333278	0,000749559
FAM95B1	SPATA6	FAM95B1\SPATA6	0,766666651	0,021389991
FAM95B1	SPIN1	FAM95B1\SPIN1	0,833333313	0,008267196
FAM95B1	SPINT1	FAM95B1\SPINT1	0,833333313	0,008267196
FAM95B1	SPIRE2	FAM95B1\SPIRE2	0,895405293	0,002105379
FAM95B1	STARD7	FAM95B1\STARD7	0,766666651	0,021389991
FAM95B1	STEAP2	FAM95B1\STEAP2	0,799999952	0,013828263
FAM95B1	STON2	FAM95B1\STON2	0,699999928	0,043253969
FAM95B1	STX6	FAM95B1\STX6	0,766666651	0,021389991
FAM95B1	SUPT3H	FAM95B1\SUPT3H	0,686198473	0,046968695
FAM95B1	SYBU	FAM95B1\SYBU	0,916666567	0,001311728
FAM95B1	TAB3	FAM95B1\TAB3	0,699999928	0,043253969
FAM95B1	TBC1D2B	FAM95B1\TBC1D2B	0,699999928	0,043253969
FAM95B1	TM7SF3	FAM95B1\TM7SF3	0,783333302	0,017223325
FAM95B1	TMEM107	FAM95B1\TMEM107	0,766666651	0,021389991

FAM95B1	TMEM129	FAM95B1\TMEM129	0,728039861	0,031283069
FAM95B1	TMEM180	FAM95B1\TMEM180	0,745869875	0,025859788
FAM95B1	TMEM185A	FAM95B1\TMEM185A	0,883333266	0,003075397
FAM95B1	TNPO1	FAM95B1\TNPO1	0,799999952	0,013828263
FAM95B1	TP63	FAM95B1\TP63	0,716666639	0,036866181
FAM95B1	TRIM61	FAM95B1\TRIM61	0,74999994	0,025490521
FAM95B1	TRIM7	FAM95B1\TRIM7	0,699999928	0,043253969
FAM95B1	TRMT10B	FAM95B1\TRMT10B	0,783333302	0,017223325
FAM95B1	TRPM7	FAM95B1\TRPM7	0,883333266	0,003075397
FAM95B1	TRUB1	FAM95B1\TRUB1	0,73333329	0,031123236
FAM95B1	TTC22	FAM95B1\TTC22	0,899999917	0,002028219
FAM95B1	UBAC1	FAM95B1\UBAC1	0,74999994	0,025490521
FAM95B1	UBAP2	FAM95B1\UBAP2	0,799999952	0,013828263
FAM95B1	UBE2V2	FAM95B1\UBE2V2	0,761512935	0,021814374
FAM95B1	UBFD1	FAM95B1\UBFD1	0,816666603	0,010769401
FAM95B1	UBTD2	FAM95B1\UBTD2	0,728039861	0,031283069
FAM95B1	UEVLD	FAM95B1\UEVLD	0,711966693	0,038029101
FAM95B1	UNC5B	FAM95B1\UNC5B	0,883333266	0,003075397
FAM95B1	UPF1	FAM95B1\UPF1	0,866666615	0,004508378
FAM95B1	VAC14	FAM95B1\VAC14	0,833333313	0,008267196
FAM95B1	VMA21	FAM95B1\VMA21	0,799999952	0,013828263
FAM95B1	WASL	FAM95B1\WASL	0,816666603	0,010769401
FAM95B1	WDR61	FAM95B1\WDR61	0,694566727	0,043816138
FAM95B1	WDR91	FAM95B1\WDR91	0,73333329	0,031123236
FAM95B1	WNK2	FAM95B1\WNK2	0,866666615	0,004508378
FAM95B1	WTH3DI	FAM95B1\WTH3DI	0,766666651	0,021389991
FAM95B1	WWOX	FAM95B1\WWOX	0,778249502	0,017383156
FAM95B1	XPR1	FAM95B1\XPR1	0,73333329	0,031123236
FAM95B1	XYLT2	FAM95B1\XYLT2	0,799999952	0,013828263
FAM95B1	ZBTB41	FAM95B1\ZBTB41	0,916666567	0,001311728
FAM95B1	ZNF132	FAM95B1\ZNF132	0,766666651	0,021389991
FAM95B1	ZNF280B	FAM95B1\ZNF280B	0,899999917	0,002028219
FAM95B1	ZNF449	FAM95B1\ZNF449	0,887037039	0,002469136
FAM95B1	ZNF543	FAM95B1\ZNF543	0,74999994	0,025490521

FAM95B1	ZNF544	FAM95B1\ZNF544	0,766666651	0,021389991
FGF13-AS1	AJUBA	FGF13-AS1\AJUBA	0,833333313	0,008267196
FGF13-AS1	ALS2	FGF13-AS1\ALS2	0,833333313	0,008267196
FGF13-AS1	AP1S1	FGF13-AS1\AP1S1	0,799999952	0,013828263
FGF13-AS1	APOBEC2	FGF13-AS1\APOBEC2	0,783333302	0,017223325
FGF13-AS1	APTX	FGF13-AS1\APTX	0,699999928	0,043253969
FGF13-AS1	AUTS2	FGF13-AS1\AUTS2	0,766666651	0,021389991
FGF13-AS1	BAIAP2	FGF13-AS1\BAIAP2	0,783333302	0,017223325
FGF13-AS1	BRD1	FGF13-AS1\BRD1	0,916666567	0,001311728
FGF13-AS1	C14orf39	FGF13-AS1\C14orf39	0,716666639	0,036866181
FGF13-AS1	C15orf41	FGF13-AS1\C15orf41	0,73333329	0,031123236
FGF13-AS1	C19orf44	FGF13-AS1\C19orf44	0,766666651	0,021389991
FGF13-AS1	C19orf82	FGF13-AS1\C19orf82	0,766666651	0,021389991
FGF13-AS1	C1orf109	FGF13-AS1\C1orf109	0,783333302	0,017223325
FGF13-AS1	C4A	FGF13-AS1\C4A	0,816666603	0,010769401
FGF13-AS1	C4B	FGF13-AS1\C4B	0,816666603	0,010769401
FGF13-AS1	CBS	FGF13-AS1\CBS	0,74999994	0,025490521
FGF13-AS1	CCDC30	FGF13-AS1\CCDC30	0,816666603	0,010769401
FGF13-AS1	CCDC73	FGF13-AS1\CCDC73	0,811722577	0,010769401
FGF13-AS1	CCDC8	FGF13-AS1\CCDC8	0,766666651	0,021389991
FGF13-AS1	CDC42BPG	FGF13-AS1\CDC42BPG	0,870300472	0,003681658
FGF13-AS1	CDKL3	FGF13-AS1\CDKL3	0,783333302	0,017223325
FGF13-AS1	CETN3	FGF13-AS1\CETN3	0,699999928	0,043253969
FGF13-AS1	CLCA1	FGF13-AS1\CLCA1	0,783349395	0,014021164
FGF13-AS1	CLCN5	FGF13-AS1\CLCN5	0,799999952	0,013828263
FGF13-AS1	CLDN12	FGF13-AS1\CLDN12	0,716666639	0,036866181
FGF13-AS1	CLHC1	FGF13-AS1\CLHC1	0,866666615	0,004508378
FGF13-AS1	CLSTN1	FGF13-AS1\CLSTN1	0,699999928	0,043253969
FGF13-AS1	CRTC1	FGF13-AS1\CRTC1	0,73333329	0,031123236
FGF13-AS1	DCAKD	FGF13-AS1\DCAKD	0,73333329	0,031123236
FGF13-AS1	DDX31	FGF13-AS1\DDX31	0,699999928	0,043253969
FGF13-AS1	DET1	FGF13-AS1\DET1	0,799999952	0,013828263
FGF13-AS1	DNAJC16	FGF13-AS1\DNAJC16	0,699999928	0,043253969
FGF13-AS1	DNAJC21	FGF13-AS1\DNAJC21	0,73333329	0,031123236

FGF13-AS1	DNAL4	FGF13-AS1\DNAL4	0,699999928	0,043253969
FGF13-AS1	EDA2R	FGF13-AS1\EDA2R	0,716666639	0,036866181
FGF13-AS1	EPN3	FGF13-AS1\EPN3	0,783333302	0,017223325
FGF13-AS1	EPS15L1	FGF13-AS1\EPS15L1	0,916666567	0,001311728
FGF13-AS1	EXOC7	FGF13-AS1\EXOC7	0,711966693	0,038029101
FGF13-AS1	FAM218A	FGF13-AS1\FAM218A	0,866666615	0,004508378
FGF13-AS1	FBXW11	FGF13-AS1\FBXW11	0,695015132	0,044576719
FGF13-AS1	FEM1B	FGF13-AS1\FEM1B	0,783333302	0,017223325
FGF13-AS1	FOXE1	FGF13-AS1\FOXE1	0,833333313	0,008267196
FGF13-AS1	GGA1	FGF13-AS1\GGA1	0,866666615	0,004508378
FGF13-AS1	GGCT	FGF13-AS1\GGCT	0,766666651	0,021389991
FGF13-AS1	GTF2H4	FGF13-AS1\GTF2H4	0,745869875	0,026388889
FGF13-AS1	GTF3C4	FGF13-AS1\GTF3C4	0,866666615	0,004508378
FGF13-AS1	HIST1H2BC	FGF13-AS1\HIST1H2BC	0,699999928	0,043253969
FGF13-AS1	HIST2H2BF	FGF13-AS1\HIST2H2BF	0,74999994	0,025490521
FGF13-AS1	HN1L	FGF13-AS1\HN1L	0,899999917	0,002028219
FGF13-AS1	HOMER3	FGF13-AS1\HOMER3	0,73333329	0,031123236
FGF13-AS1	HPS5	FGF13-AS1\HPS5	0,833333313	0,008267196
FGF13-AS1	HPSE	FGF13-AS1\HPSE	0,73333329	0,031123236
FGF13-AS1	IGSF3	FGF13-AS1\IGSF3	0,73333329	0,031123236
FGF13-AS1	KAL1	FGF13-AS1\KAL1	0,833333313	0,008267196
FGF13-AS1	KRTAP5-8	FGF13-AS1\KRTAP5-8	0,866666615	0,004508378
FGF13-AS1	LAMP5	FGF13-AS1\LAMP5	0,73333329	0,031123236
FGF13-AS1	LARS	FGF13-AS1\LARS	0,699999928	0,043253969
FGF13-AS1	LPHN3	FGF13-AS1\LPHN3	0,716666639	0,036866181
FGF13-AS1	LRPAP1	FGF13-AS1\LRPAP1	0,686198473	0,046968695
FGF13-AS1	LRPPRC	FGF13-AS1\LRPPRC	0,766666651	0,021389991
FGF13-AS1	LRRC27	FGF13-AS1\LRRC27	0,716666639	0,036866181
FGF13-AS1	LZIC	FGF13-AS1\LZIC	0,799999952	0,013828263
FGF13-AS1	MBLAC2	FGF13-AS1\MBLAC2	0,783333302	0,017223325
FGF13-AS1	MCTP2	FGF13-AS1\MCTP2	0,716666639	0,036866181
FGF13-AS1	METTL10	FGF13-AS1\METTL10	0,74999994	0,025490521
FGF13-AS1	MTPAP	FGF13-AS1\MTPAP	0,699999928	0,043253969
FGF13-AS1	MYLK4	FGF13-AS1\MYLK4	0,816666603	0,010769401

FGF13-AS1	N4BP1	FGF13-AS1\N4BP1	0,699999928	0,043253969
FGF13-AS1	NAA25	FGF13-AS1\NAA25	0,849999964	0,006073633
FGF13-AS1	NDRG3	FGF13-AS1\NDRG3	0,74999994	0,025490521
FGF13-AS1	NHLRC3	FGF13-AS1\NHLRC3	0,816666603	0,010769401
FGF13-AS1	NPNT	FGF13-AS1\NPNT	0,849999964	0,006073633
FGF13-AS1	NSRP1	FGF13-AS1\NSRP1	0,799999952	0,013828263
FGF13-AS1	NUDT12	FGF13-AS1\NUDT12	0,686198473	0,046968695
FGF13-AS1	OSBPL3	FGF13-AS1\OSBPL3	0,73333329	0,031123236
FGF13-AS1	PDGFA	FGF13-AS1\PDGFA	0,73333329	0,031123236
FGF13-AS1	PLA2G12A	FGF13-AS1\PLA2G12A	0,766666651	0,021389991
FGF13-AS1	PMPCB	FGF13-AS1\PMPCB	0,74999994	0,025490521
FGF13-AS1	POGZ	FGF13-AS1\POGZ	0,816666603	0,010769401
FGF13-AS1	POMT2	FGF13-AS1\POMT2	0,783333302	0,017223325
FGF13-AS1	POTEE	FGF13-AS1\POTEE	0,699999928	0,043253969
FGF13-AS1	POTEF	FGF13-AS1\POTEF	0,716666639	0,036866181
FGF13-AS1	PRKAB1	FGF13-AS1\PRKAB1	0,833333313	0,008267196
FGF13-AS1	PRKAR1A	FGF13-AS1\PRKAR1A	0,796724617	0,013227513
FGF13-AS1	PRRC1	FGF13-AS1\PRRC1	0,816666603	0,010769401
FGF13-AS1	PSAT1	FGF13-AS1\PSAT1	0,783333302	0,017223325
FGF13-AS1	PSD3	FGF13-AS1\PSD3	0,74999994	0,025490521
FGF13-AS1	PTPRK	FGF13-AS1\PTPRK	0,833333313	0,008267196
FGF13-AS1	RAB23	FGF13-AS1\RAB23	0,833333313	0,008267196
FGF13-AS1	RAB40C	FGF13-AS1\RAB40C	0,833333313	0,008267196
FGF13-AS1	RABGAP1	FGF13-AS1\RABGAP1	0,783333302	0,017223325
FGF13-AS1	RANBP17	FGF13-AS1\RANBP17	0,766666651	0,021389991
FGF13-AS1	RAVER2	FGF13-AS1\RAVER2	0,73333329	0,031123236
FGF13-AS1	RGS21	FGF13-AS1\RGS21	0,813676238	0,010449735
FGF13-AS1	RGS9	FGF13-AS1\RGS9	0,73333329	0,031123236
FGF13-AS1	RMDN3	FGF13-AS1\RMDN3	0,728039861	0,031283069
FGF13-AS1	RNF212	FGF13-AS1\RNF212	0,899999917	0,002028219
FGF13-AS1	RPGRIP1L	FGF13-AS1\RPGRIP1L	0,816666603	0,010769401
FGF13-AS1	RPL37	FGF13-AS1\RPL37	0,74999994	0,025490521
FGF13-AS1	RPS6KA6	FGF13-AS1\RPS6KA6	0,73333329	0,031123236
FGF13-AS1	RRAGB	FGF13-AS1\RRAGB	0,799999952	0,013828263

FGF13-AS1	SARS2	FGF13-AS1\SARS2	0,719671547	0,033763226
FGF13-AS1	SCAMP1	FGF13-AS1\SCAMP1	0,74999994	0,025490521
FGF13-AS1	SDR42E1	FGF13-AS1\SDR42E1	0,74999994	0,025490521
FGF13-AS1	SIX1	FGF13-AS1\SIX1	0,73333329	0,031123236
FGF13-AS1	SIX4	FGF13-AS1\SIX4	0,849999964	0,006073633
FGF13-AS1	SLC15A1	FGF13-AS1\SLC15A1	0,694566727	0,043816138
FGF13-AS1	SLC30A6	FGF13-AS1\SLC30A6	0,73333329	0,031123236
FGF13-AS1	SMO	FGF13-AS1\SMO	0,73333329	0,031123236
FGF13-AS1	SPIN1	FGF13-AS1\SPIN1	0,933333278	0,000749559
FGF13-AS1	SPIN4	FGF13-AS1\SPIN4	0,74999994	0,025490521
FGF13-AS1	STARD7	FGF13-AS1\STARD7	0,816666603	0,010769401
FGF13-AS1	STK36	FGF13-AS1\STK36	0,866666615	0,004508378
FGF13-AS1	SUPT3H	FGF13-AS1\SUPT3H	0,878668785	0,003218695
FGF13-AS1	SYT1	FGF13-AS1\SYT1	0,74999994	0,025490521
FGF13-AS1	TAB3	FGF13-AS1\TAB3	0,799999952	0,013828263
FGF13-AS1	TFCP2L1	FGF13-AS1\TFCP2L1	0,73333329	0,031123236
FGF13-AS1	TMEM180	FGF13-AS1\TMEM180	0,813676238	0,010449735
FGF13-AS1	TMEM185A	FGF13-AS1\TMEM185A	0,716666639	0,036866181
FGF13-AS1	TRMT10B	FGF13-AS1\TRMT10B	0,883333266	0,003075397
FGF13-AS1	TRMT5	FGF13-AS1\TRMT5	0,799999952	0,013828263
FGF13-AS1	TTC26	FGF13-AS1\TTC26	0,783333302	0,017223325
FGF13-AS1	TYW5	FGF13-AS1\TYW5	0,74999994	0,025490521
FGF13-AS1	UBE2V2	FGF13-AS1\UBE2V2	0,728039861	0,031988535
FGF13-AS1	UEVLD	FGF13-AS1\UEVLD	0,84757942	0,00598545
FGF13-AS1	VWCE	FGF13-AS1\VWCE	0,799999952	0,013828263
FGF13-AS1	WASL	FGF13-AS1\WASL	0,816666603	0,010769401
FGF13-AS1	XPO7	FGF13-AS1\XPO7	0,766666651	0,021389991
FGF13-AS1	XPR1	FGF13-AS1\XPR1	0,766666651	0,021389991
FGF13-AS1	ZNF211	FGF13-AS1\ZNF211	0,766666651	0,021389991
FGF13-AS1	ZNF221	FGF13-AS1\ZNF221	0,799999952	0,013828263
FGF13-AS1	ZNF257	FGF13-AS1\ZNF257	0,916666567	0,001311728
FGF13-AS1	ZNF449	FGF13-AS1\ZNF449	0,686198473	0,046968695
FGF13-AS1	ZNF540	FGF13-AS1\ZNF540	0,899999917	0,002028219
FGF13-AS1	ZNF543	FGF13-AS1\ZNF543	0,849999964	0,006073633

FGF13-AS1	ZNF544	FGF13-AS1\ZNF544	0,73333329	0,031123236
FGF13-AS1	ZNF562	FGF13-AS1\ZNF562	0,883333266	0,003075397
FGF13-AS1	ZNF605	FGF13-AS1\ZNF605	0,816666603	0,010769401
FGF13-AS1	ZRANB3	FGF13-AS1\ZRANB3	0,699999928	0,043253969
FGF13-AS1	ZSCAN31	FGF13-AS1\ZSCAN31	0,816666603	0,010769401
KCNQ1OT1	AJUBA	KCNQ1OT1\AJUBA	0,949999988	0,000352734
KCNQ1OT1	AKR1B1	KCNQ1OT1\AKR1B1	0,699999928	0,043253969
KCNQ1OT1	ALDH7A1	KCNQ1OT1\ALDH7A1	0,849999964	0,006073633
KCNQ1OT1	ALS2	KCNQ1OT1\ALS2	0,899999917	0,002028219
KCNQ1OT1	ANK3	KCNQ1OT1\ANK3	0,866666615	0,004508378
KCNQ1OT1	AP1S1	KCNQ1OT1\AP1S1	0,816666603	0,010769401
KCNQ1OT1	AP2B1	KCNQ1OT1\AP2B1	0,73333329	0,031123236
KCNQ1OT1	APOBEC2	KCNQ1OT1\APOBEC2	0,783333302	0,017223325
KCNQ1OT1	APP	KCNQ1OT1\APP	0,816666603	0,010769401
KCNQ1OT1	ATP6AP2	KCNQ1OT1\ATP6AP2	0,699999928	0,043253969
KCNQ1OT1	ATP6V0A1	KCNQ1OT1\ATP6V0A1	0,864530981	0,004596561
KCNQ1OT1	ATP6V0D1	KCNQ1OT1\ATP6V0D1	0,766666651	0,021389991
KCNQ1OT1	AUTS2	KCNQ1OT1\AUTS2	0,949999988	0,000352734
KCNQ1OT1	AZIN1	KCNQ1OT1\AZIN1	0,833333313	0,008267196
KCNQ1OT1	BAIAP2	KCNQ1OT1\BAIAP2	0,916666567	0,001311728
KCNQ1OT1	BRD1	KCNQ1OT1\BRD1	0,883333266	0,003075397
KCNQ1OT1	C14orf39	KCNQ1OT1\C14orf39	0,849999964	0,006073633
KCNQ1OT1	C15orf41	KCNQ1OT1\C15orf41	0,699999928	0,043253969
KCNQ1OT1	C19orf44	KCNQ1OT1\C19orf44	0,716666639	0,036866181
KCNQ1OT1	C19orf82	KCNQ1OT1\C19orf82	0,73333329	0,031123236
KCNQ1OT1	C1orf109	KCNQ1OT1\C1orf109	0,699999928	0,043253969
KCNQ1OT1	C4A	KCNQ1OT1\C4A	0,933333278	0,000749559
KCNQ1OT1	C4B	KCNQ1OT1\C4B	0,933333278	0,000749559
KCNQ1OT1	C5orf15	KCNQ1OT1\C5orf15	0,699999928	0,043253969
KCNQ1OT1	CALR	KCNQ1OT1\CALR	0,74999994	0,025490521
KCNQ1OT1	CBS	KCNQ1OT1\CBS	0,783333302	0,017223325
KCNQ1OT1	CCDC122	KCNQ1OT1\CCDC122	0,866666615	0,004508378
KCNQ1OT1	CCDC148	KCNQ1OT1\CCDC148	0,849999964	0,006073633
KCNQ1OT1	CCDC30	KCNQ1OT1\CCDC30	0,933333278	0,000749559

KCNQ1OT1	CCDC73	KCNQ1OT1\CCDC73	0,870300472	0,003681658
KCNQ1OT1	CCDC8	KCNQ1OT1\CCDC8	0,933333278	0,000749559
KCNQ1OT1	CDC42BPG	KCNQ1OT1\CDC42BPG	0,878668785	0,003218695
KCNQ1OT1	CDH1	KCNQ1OT1\CDH1	0,783333302	0,017223325
KCNQ1OT1	CDIP1	KCNQ1OT1\CDIP1	0,716666639	0,036866181
KCNQ1OT1	CDKL3	KCNQ1OT1\CDKL3	0,883333266	0,003075397
KCNQ1OT1	CDS1	KCNQ1OT1\CDS1	0,74999994	0,025490521
KCNQ1OT1	CETN3	KCNQ1OT1\CETN3	0,799999952	0,013828263
KCNQ1OT1	CHID1	KCNQ1OT1\CHID1	0,816666603	0,010769401
KCNQ1OT1	CLCA1	KCNQ1OT1\CLCA1	0,792053282	0,012566137
KCNQ1OT1	CLDN12	KCNQ1OT1\CLDN12	0,833333313	0,008267196
KCNQ1OT1	CLHC1	KCNQ1OT1\CLHC1	0,74999994	0,025490521
KCNQ1OT1	CLSTN1	KCNQ1OT1\CLSTN1	0,916666567	0,001311728
KCNQ1OT1	CNTNAP2	KCNQ1OT1\CNTNAP2	0,73333329	0,031123236
KCNQ1OT1	CNTNAP3	KCNQ1OT1\CNTNAP3	0,716666639	0,036866181
KCNQ1OT1	CNTNAP3B	KCNQ1OT1\CNTNAP3B	0,783333302	0,017223325
KCNQ1OT1	COG5	KCNQ1OT1\COG5	0,699999928	0,043253969
KCNQ1OT1	COG8	KCNQ1OT1\COG8	0,719671547	0,033763226
KCNQ1OT1	COL8A2	KCNQ1OT1\COL8A2	0,783333302	0,017223325
KCNQ1OT1	COX19	KCNQ1OT1\COX19	0,833333313	0,008267196
KCNQ1OT1	CRTC1	KCNQ1OT1\CRTC1	0,899999917	0,002028219
KCNQ1OT1	DCAKD	KCNQ1OT1\DCAKD	0,816666603	0,010769401
KCNQ1OT1	DDX31	KCNQ1OT1\DDX31	0,833333313	0,008267196
KCNQ1OT1	DENND1A	KCNQ1OT1\DENND1A	0,74999994	0,025490521
KCNQ1OT1	DET1	KCNQ1OT1\DET1	0,883333266	0,003075397
KCNQ1OT1	DHX32	KCNQ1OT1\DHX32	0,899999917	0,002028219
KCNQ1OT1	DMRT2	KCNQ1OT1\DMRT2	0,783333302	0,017223325
KCNQ1OT1	DMRTA1	KCNQ1OT1\DMRTA1	0,716666639	0,036866181
KCNQ1OT1	DNAJC16	KCNQ1OT1\DNAJC16	0,73333329	0,031123236
KCNQ1OT1	DNAJC19	KCNQ1OT1\DNAJC19	0,849999964	0,006073633
KCNQ1OT1	DNAJC21	KCNQ1OT1\DNAJC21	0,766666651	0,021389991
KCNQ1OT1	DNAL4	KCNQ1OT1\DNAL4	0,933333278	0,000749559
KCNQ1OT1	DPH6	KCNQ1OT1\DPH6	0,849999964	0,006073633
KCNQ1OT1	DSG2	KCNQ1OT1\DSG2	0,766666651	0,021389991

KCNQ1OT1	DUOX1	KCNQ1OT1\DUOX1	0,70293504	0,040145501
KCNQ1OT1	DUOXA1	KCNQ1OT1\DUOXA1	0,866666615	0,004508378
KCNQ1OT1	EDA2R	KCNQ1OT1\EDA2R	0,933333278	0,000749559
KCNQ1OT1	EFNB2	KCNQ1OT1\EFNB2	0,816666603	0,010769401
KCNQ1OT1	EIF2AK4	KCNQ1OT1\EIF2AK4	0,799999952	0,013828263
KCNQ1OT1	EMC1	KCNQ1OT1\EMC1	0,699999928	0,043253969
KCNQ1OT1	EMC10	KCNQ1OT1\EMC10	0,895405293	0,002105379
KCNQ1OT1	EPN3	KCNQ1OT1\EPN3	0,883333266	0,003075397
KCNQ1OT1	EPS15L1	KCNQ1OT1\EPS15L1	0,866666615	0,004508378
KCNQ1OT1	ESRP1	KCNQ1OT1\ESRP1	0,799999952	0,013828263
KCNQ1OT1	ESRP2	KCNQ1OT1\ESRP2	0,716666639	0,036866181
KCNQ1OT1	EVA1A	KCNQ1OT1\EVA1A	0,766666651	0,021389991
KCNQ1OT1	EXOC7	KCNQ1OT1\EXOC7	0,711966693	0,038029101
KCNQ1OT1	EYA2	KCNQ1OT1\EYA2	0,766666651	0,021389991
KCNQ1OT1	FAHD1	KCNQ1OT1\FAHD1	0,816666603	0,010769401
KCNQ1OT1	FAM154B	KCNQ1OT1\FAM154B	0,779773057	0,016765874
KCNQ1OT1	FAM160A1	KCNQ1OT1\FAM160A1	0,933333278	0,000749559
KCNQ1OT1	FAM218A	KCNQ1OT1\FAM218A	0,966666639	0,000165344
KCNQ1OT1	FAM83B	KCNQ1OT1\FAM83B	0,74999994	0,025490521
KCNQ1OT1	FBXL16	KCNQ1OT1\FBXL16	0,816666603	0,010769401
KCNQ1OT1	FEM1B	KCNQ1OT1\FEM1B	0,899999917	0,002028219
KCNQ1OT1	FNBP1L	KCNQ1OT1\FNBP1L	0,883333266	0,003075397
KCNQ1OT1	FOXE1	KCNQ1OT1\FOXE1	0,799999952	0,013828263
KCNQ1OT1	GGA1	KCNQ1OT1\GGA1	0,916666567	0,001311728
KCNQ1OT1	GGCT	KCNQ1OT1\GGCT	0,74999994	0,025490521
KCNQ1OT1	GLI2	KCNQ1OT1\GLI2	0,699999928	0,043253969
KCNQ1OT1	GNPDA1	KCNQ1OT1\GNPDA1	0,866666615	0,004508378
KCNQ1OT1	GOLM1	KCNQ1OT1\GOLM1	0,799999952	0,013828263
KCNQ1OT1	GPC4	KCNQ1OT1\GPC4	0,866666615	0,004508378
KCNQ1OT1	GPR107	KCNQ1OT1\GPR107	0,728918254	0,031349208
KCNQ1OT1	GRHL2	KCNQ1OT1\GRHL2	0,799999952	0,013828263
KCNQ1OT1	GRIP1	KCNQ1OT1\GRIP1	0,783333302	0,017223325
KCNQ1OT1	GRTP1	KCNQ1OT1\GRTP1	0,849999964	0,006073633
KCNQ1OT1	GTF2IRD2	KCNQ1OT1\GTF2IRD2	0,73333329	0,031123236

KCNQ1OT1	GTF3C4	KCNQ1OT1\GTF3C4	0,899999917	0,002028219
KCNQ1OT1	HDGFRP3	KCNQ1OT1\HDGFRP3	0,716666639	0,036866181
KCNQ1OT1	HIST2H2BF	KCNQ1OT1\HIST2H2BF	0,716666639	0,036866181
KCNQ1OT1	HN1L	KCNQ1OT1\HN1L	0,866666615	0,004508378
KCNQ1OT1	HOMER3	KCNQ1OT1\HOMER3	0,966666639	0,000165344
KCNQ1OT1	HPS5	KCNQ1OT1\HPS5	0,899999917	0,002028219
KCNQ1OT1	HPSE	KCNQ1OT1\HPSE	0,816666603	0,010769401
KCNQ1OT1	IFT140	KCNQ1OT1\IFT140	0,699999928	0,043253969
KCNQ1OT1	IGF1R	KCNQ1OT1\IGF1R	0,766666651	0,021389991
KCNQ1OT1	IGFBP2	KCNQ1OT1\IGFBP2	0,74999994	0,025490521
KCNQ1OT1	IGSF3	KCNQ1OT1\IGSF3	0,833333313	0,008267196
KCNQ1OT1	IKBIP	KCNQ1OT1\IKBIP	0,799999952	0,013828263
KCNQ1OT1	IQCE	KCNQ1OT1\IQCE	0,716666639	0,036866181
KCNQ1OT1	IQCH	KCNQ1OT1\IQCH	0,699999928	0,043253969
KCNQ1OT1	IRF2BP2	KCNQ1OT1\IRF2BP2	0,766666651	0,021389991
KCNQ1OT1	ITGA2	KCNQ1OT1\ITGA2	0,849999964	0,006073633
KCNQ1OT1	ITGAV	KCNQ1OT1\ITGAV	0,766666651	0,021389991
KCNQ1OT1	KAL1	KCNQ1OT1\KAL1	0,866666615	0,004508378
KCNQ1OT1	KCTD1	KCNQ1OT1\KCTD1	0,799999952	0,013828263
KCNQ1OT1	KDM4B	KCNQ1OT1\KDM4B	0,716666639	0,036866181
KCNQ1OT1	KDM4D	KCNQ1OT1\KDM4D	0,686198473	0,046968695
KCNQ1OT1	KIAA0319L	KCNQ1OT1\KIAA0319L	0,73333329	0,031123236
KCNQ1OT1	KLHDC10	KCNQ1OT1\KLHDC10	0,716666639	0,036866181
KCNQ1OT1	KRTAP5-8	KCNQ1OT1\KRTAP5-8	0,916666567	0,001311728
KCNQ1OT1	KTN1	KCNQ1OT1\KTN1	0,74999994	0,025490521
KCNQ1OT1	LACC1	KCNQ1OT1\LACC1	0,778249502	0,017383156
KCNQ1OT1	LAMP1	KCNQ1OT1\LAMP1	0,786617756	0,015288801
KCNQ1OT1	LAMP5	KCNQ1OT1\LAMP5	0,766666651	0,021389991
KCNQ1OT1	LARS	KCNQ1OT1\LARS	0,74999994	0,025490521
KCNQ1OT1	LEPREL1	KCNQ1OT1\LEPREL1	0,933333278	0,000749559
KCNQ1OT1	LPHN3	KCNQ1OT1\LPHN3	0,849999964	0,006073633
KCNQ1OT1	LPIN1	KCNQ1OT1\LPIN1	0,699999928	0,043253969
KCNQ1OT1	LRIG3	KCNQ1OT1\LRIG3	0,916666567	0,001311728
KCNQ1OT1	LRPAP1	KCNQ1OT1\LRPAP1	0,728039861	0,031283069

KCNQ1OT1	LRPPRC	KCNQ1OT1\LRPPRC	0,74999994	0,025490521
KCNQ1OT1	LRRK1	KCNQ1OT1\LRRK1	0,849999964	0,006073633
KCNQ1OT1	LZIC	KCNQ1OT1\LZIC	0,73333329	0,031123236
KCNQ1OT1	M1AP	KCNQ1OT1\M1AP	0,783333302	0,017223325
KCNQ1OT1	MAGEA3	KCNQ1OT1\MAGEA3	0,766666651	0,021389991
KCNQ1OT1	MAML3	KCNQ1OT1\MAML3	0,699999928	0,043253969
KCNQ1OT1	MARVELD2	KCNQ1OT1\MARVELD2	0,799999952	0,013828263
KCNQ1OT1	MAST4	KCNQ1OT1\MAST4	0,769881189	0,018992504
KCNQ1OT1	MBLAC2	KCNQ1OT1\MBLAC2	0,716666639	0,036866181
KCNQ1OT1	MCTP2	KCNQ1OT1\MCTP2	0,899999917	0,002028219
KCNQ1OT1	MED22	KCNQ1OT1\MED22	0,711966693	0,037896827
KCNQ1OT1	MPP5	KCNQ1OT1\MPP5	0,699999928	0,043253969
KCNQ1OT1	MRPL40	KCNQ1OT1\MRPL40	0,74999994	0,025490521
KCNQ1OT1	MST1L	KCNQ1OT1\MST1L	0,73333329	0,031123236
KCNQ1OT1	MTPAP	KCNQ1OT1\MTPAP	0,73333329	0,031123236
KCNQ1OT1	MYLK4	KCNQ1OT1\MYLK4	0,766666651	0,021389991
KCNQ1OT1	MYO10	KCNQ1OT1\MYO10	0,833333313	0,008267196
KCNQ1OT1	MYO5B	KCNQ1OT1\MYO5B	0,811722577	0,010769401
KCNQ1OT1	N4BP1	KCNQ1OT1\N4BP1	0,833333313	0,008267196
KCNQ1OT1	NAA25	KCNQ1OT1\NAA25	0,73333329	0,031123236
KCNQ1OT1	NAGK	KCNQ1OT1\NAGK	0,799999952	0,013828263
KCNQ1OT1	NCS1	KCNQ1OT1\NCS1	0,73333329	0,031123236
KCNQ1OT1	NDRG3	KCNQ1OT1\NDRG3	0,866666615	0,004508378
KCNQ1OT1	NDUFA4	KCNQ1OT1\NDUFA4	0,73333329	0,031123236
KCNQ1OT1	NEO1	KCNQ1OT1\NEO1	0,933333278	0,000749559
KCNQ1OT1	NET1	KCNQ1OT1\NET1	0,833333313	0,008267196
KCNQ1OT1	NETO2	KCNQ1OT1\NETO2	0,74999994	0,025490521
KCNQ1OT1	NHLRC3	KCNQ1OT1\NHLRC3	0,98333329	4,96032E-05
KCNQ1OT1	NPNT	KCNQ1OT1\NPNT	0,99999994	5,51146E-06
KCNQ1OT1	NRCAM	KCNQ1OT1\NRCAM	0,816666603	0,010769401
KCNQ1OT1	NTF4	KCNQ1OT1\NTF4	0,833333313	0,008267196
KCNQ1OT1	NUDT12	KCNQ1OT1\NUDT12	0,70293504	0,040145501
KCNQ1OT1	NUDT9	KCNQ1OT1\NUDT9	0,74999994	0,025490521
KCNQ1OT1	NXN	KCNQ1OT1\NXN	0,836827397	0,00696649

KCNQ1OT1	OSBPL3	KCNQ1OT1\OSBPL3	0,716666639	0,036866181
KCNQ1OT1	PABPC4L	KCNQ1OT1\PABPC4L	0,783333302	0,017223325
KCNQ1OT1	PARD6B	KCNQ1OT1\PARD6B	0,716666639	0,036866181
KCNQ1OT1	PAX1	KCNQ1OT1\PAX1	0,866666615	0,004508378
KCNQ1OT1	PAX9	KCNQ1OT1\PAX9	0,866666615	0,004508378
KCNQ1OT1	PCSK6	KCNQ1OT1\PCSK6	0,816666603	0,010769401
KCNQ1OT1	PDGFA	KCNQ1OT1\PDGFA	0,933333278	0,000749559
KCNQ1OT1	PDPK1	KCNQ1OT1\PDPK1	0,73333329	0,031123236
KCNQ1OT1	PGAP1	KCNQ1OT1\PGAP1	0,833333313	0,008267196
KCNQ1OT1	PIAS2	KCNQ1OT1\PIAS2	0,766666651	0,021389991
KCNQ1OT1	PIFO	KCNQ1OT1\PIFO	0,766666651	0,021389991
KCNQ1OT1	PKP1	KCNQ1OT1\PKP1	0,73333329	0,031123236
KCNQ1OT1	PKP4	KCNQ1OT1\PKP4	0,816666603	0,010769401
KCNQ1OT1	PLA2G12A	KCNQ1OT1\PLA2G12A	0,883333266	0,003075397
KCNQ1OT1	PLD2	KCNQ1OT1\PLD2	0,73333329	0,031123236
KCNQ1OT1	PMPCB	KCNQ1OT1\PMPCB	0,766666651	0,021389991
KCNQ1OT1	PNMAL2	KCNQ1OT1\PNMAL2	0,783333302	0,017223325
KCNQ1OT1	POGZ	KCNQ1OT1\POGZ	0,816666603	0,010769401
KCNQ1OT1	POMT2	KCNQ1OT1\POMT2	0,966666639	0,000165344
KCNQ1OT1	POTEE	KCNQ1OT1\POTEE	0,699999928	0,043253969
KCNQ1OT1	PRKAA2	KCNQ1OT1\PRKAA2	0,816666603	0,010769401
KCNQ1OT1	PRKAB1	KCNQ1OT1\PRKAB1	0,866666615	0,004508378
KCNQ1OT1	PRKAR1A	KCNQ1OT1\PRKAR1A	0,898434162	0,002050265
KCNQ1OT1	PRRC1	KCNQ1OT1\PRRC1	0,766666651	0,021389991
KCNQ1OT1	PRRG4	KCNQ1OT1\PRRG4	0,866666615	0,004508378
KCNQ1OT1	PSAT1	KCNQ1OT1\PSAT1	0,883333266	0,003075397
KCNQ1OT1	PSD3	KCNQ1OT1\PSD3	0,866666615	0,004508378
KCNQ1OT1	RAB14	KCNQ1OT1\RAB14	0,849999964	0,006073633
KCNQ1OT1	RAB23	KCNQ1OT1\RAB23	0,933333278	0,000749559
KCNQ1OT1	RAB40C	KCNQ1OT1\RAB40C	0,73333329	0,031123236
KCNQ1OT1	RABGAP1	KCNQ1OT1\RABGAP1	0,916666567	0,001311728
KCNQ1OT1	RAI1	KCNQ1OT1\RAI1	0,719671547	0,033763226
KCNQ1OT1	RANBP17	KCNQ1OT1\RANBP17	0,899999917	0,002028219
KCNQ1OT1	RAVER2	KCNQ1OT1\RAVER2	0,699999928	0,043253969

KCNQ1OT1	RGS9	KCNQ1OT1\RGS9	0,716666639	0,036866181
KCNQ1OT1	RHBDD2	KCNQ1OT1\RHBDD2	0,799999952	0,013828263
KCNQ1OT1	RMDN3	KCNQ1OT1\RMDN3	0,828459144	0,008289241
KCNQ1OT1	RNF170	KCNQ1OT1\RNF170	0,766666651	0,021389991
KCNQ1OT1	RNF212	KCNQ1OT1\RNF212	0,899999917	0,002028219
KCNQ1OT1	RPGRIP1L	KCNQ1OT1\RPGRIP1L	0,833333313	0,008267196
KCNQ1OT1	RPS6KA6	KCNQ1OT1\RPS6KA6	0,866666615	0,004508378
KCNQ1OT1	RRM2B	KCNQ1OT1\RRM2B	0,816666603	0,010769401
KCNQ1OT1	SCAMP1	KCNQ1OT1\SCAMP1	0,866666615	0,004508378
KCNQ1OT1	SDR42E1	KCNQ1OT1\SDR42E1	0,916666567	0,001311728
KCNQ1OT1	SEC61A1	KCNQ1OT1\SEC61A1	0,73333329	0,031123236
KCNQ1OT1	SERPINB5	KCNQ1OT1\SERPINB5	0,74999994	0,025490521
KCNQ1OT1	SGPL1	KCNQ1OT1\SGPL1	0,833333313	0,008267196
KCNQ1OT1	SHROOM2	KCNQ1OT1\SHROOM2	0,716666639	0,036866181
KCNQ1OT1	SIX1	KCNQ1OT1\SIX1	0,966666639	0,000165344
KCNQ1OT1	SIX4	KCNQ1OT1\SIX4	0,916666567	0,001311728
KCNQ1OT1	SLC15A1	KCNQ1OT1\SLC15A1	0,694566727	0,043816138
KCNQ1OT1	SLC22A5	KCNQ1OT1\SLC22A5	0,74999994	0,025490521
KCNQ1OT1	SLC30A1	KCNQ1OT1\SLC30A1	0,799999952	0,013828263
KCNQ1OT1	SLC45A4	KCNQ1OT1\SLC45A4	0,699999928	0,043253969
KCNQ1OT1	SLC46A1	KCNQ1OT1\SLC46A1	0,799999952	0,013828263
KCNQ1OT1	SLCO5A1	KCNQ1OT1\SLCO5A1	0,699999928	0,043253969
KCNQ1OT1	SMO	KCNQ1OT1\SMO	0,866666615	0,004508378
KCNQ1OT1	SOGA2	KCNQ1OT1\SOGA2	0,73333329	0,031123236
KCNQ1OT1	SORBS2	KCNQ1OT1\SORBS2	0,766666651	0,021389991
KCNQ1OT1	SORCS2	KCNQ1OT1\SORCS2	0,716666639	0,036866181
KCNQ1OT1	SPATA6	KCNQ1OT1\SPATA6	0,799999952	0,013828263
KCNQ1OT1	SPIN1	KCNQ1OT1\SPIN1	0,916666567	0,001311728
KCNQ1OT1	SPIN4	KCNQ1OT1\SPIN4	0,716666639	0,036866181
KCNQ1OT1	SPINT1	KCNQ1OT1\SPINT1	0,849999964	0,006073633
KCNQ1OT1	ST7L	KCNQ1OT1\ST7L	0,74999994	0,025490521
KCNQ1OT1	STARD7	KCNQ1OT1\STARD7	0,816666603	0,010769401
KCNQ1OT1	STEAP2	KCNQ1OT1\STEAP2	0,783333302	0,017223325
KCNQ1OT1	STK36	KCNQ1OT1\STK36	0,799999952	0,013828263

KCNQ1OT1	STON2	KCNQ1OT1\STON2	0,74999994	0,025490521
KCNQ1OT1	SUPT3H	KCNQ1OT1\SUPT3H	0,853563964	0,005202822
KCNQ1OT1	SYT1	KCNQ1OT1\SYT1	0,816666603	0,010769401
KCNQ1OT1	TAB3	KCNQ1OT1\TAB3	0,799999952	0,013828263
KCNQ1OT1	TDGF1	KCNQ1OT1\TDGF1	0,699999928	0,043253969
KCNQ1OT1	TM7SF3	KCNQ1OT1\TM7SF3	0,716666639	0,036866181
KCNQ1OT1	TMEM133	KCNQ1OT1\TMEM133	0,916666567	0,001311728
KCNQ1OT1	TMEM180	KCNQ1OT1\TMEM180	0,881482542	0,003009259
KCNQ1OT1	TMEM185A	KCNQ1OT1\TMEM185A	0,816666603	0,010769401
KCNQ1OT1	TMEM30B	KCNQ1OT1\TMEM30B	0,899999917	0,002028219
KCNQ1OT1	TNPO1	KCNQ1OT1\TNPO1	0,783333302	0,017223325
KCNQ1OT1	TOMM20	KCNQ1OT1\TOMM20	0,833333313	0,008267196
KCNQ1OT1	TP63	KCNQ1OT1\TP63	0,699999928	0,043253969
KCNQ1OT1	TRMT10B	KCNQ1OT1\TRMT10B	0,816666603	0,010769401
KCNQ1OT1	TRMT5	KCNQ1OT1\TRMT5	0,883333266	0,003075397
KCNQ1OT1	TRPM7	KCNQ1OT1\TRPM7	0,716666639	0,036866181
KCNQ1OT1	TSPAN13	KCNQ1OT1\TSPAN13	0,799999952	0,013828263
KCNQ1OT1	TTC22	KCNQ1OT1\TTC22	0,783333302	0,017223325
KCNQ1OT1	TTC26	KCNQ1OT1\TTC26	0,783333302	0,017223325
KCNQ1OT1	TXNL1	KCNQ1OT1\TXNL1	0,73333329	0,031123236
KCNQ1OT1	TYW5	KCNQ1OT1\TYW5	0,799999952	0,013828263
KCNQ1OT1	UBE2V2	KCNQ1OT1\UBE2V2	0,728039861	0,031988535
KCNQ1OT1	UBTD2	KCNQ1OT1\UBTD2	0,811722577	0,010769401
KCNQ1OT1	UEVLD	KCNQ1OT1\UEVLD	0,915385723	0,001322751
KCNQ1OT1	UMPS	KCNQ1OT1\UMPS	0,728918254	0,031679895
KCNQ1OT1	UNC5B	KCNQ1OT1\UNC5B	0,816666603	0,010769401
KCNQ1OT1	UPF1	KCNQ1OT1\UPF1	0,73333329	0,031123236
KCNQ1OT1	USP28	KCNQ1OT1\USP28	0,766666651	0,021389991
KCNQ1OT1	USP46	KCNQ1OT1\USP46	0,883333266	0,003075397
KCNQ1OT1	VPS35	KCNQ1OT1\VPS35	0,799999952	0,013828263
KCNQ1OT1	VWCE	KCNQ1OT1\VWCE	0,799999952	0,013828263
KCNQ1OT1	WASL	KCNQ1OT1\WASL	0,98333329	4,96032E-05
KCNQ1OT1	WDR91	KCNQ1OT1\WDR91	0,716666639	0,036866181
KCNQ1OT1	XPO7	KCNQ1OT1\XPO7	0,716666639	0,036866181

KCNQ1OT1	XPR1	KCNQ1OT1\XPR1	0,833333313	0,008267196
KCNQ1OT1	ZBTB41	KCNQ1OT1\ZBTB41	0,783333302	0,017223325
KCNQ1OT1	ZNF211	KCNQ1OT1\ZNF211	0,783333302	0,017223325
KCNQ1OT1	ZNF221	KCNQ1OT1\ZNF221	0,883333266	0,003075397
KCNQ1OT1	ZNF229	KCNQ1OT1\ZNF229	0,73333329	0,031123236
KCNQ1OT1	ZNF257	KCNQ1OT1\ZNF257	0,699999928	0,043253969
KCNQ1OT1	ZNF280B	KCNQ1OT1\ZNF280B	0,849999964	0,006073633
KCNQ1OT1	ZNF449	KCNQ1OT1\ZNF449	0,82009083	0,009281305
KCNQ1OT1	ZNF540	KCNQ1OT1\ZNF540	0,74999994	0,025490521
KCNQ1OT1	ZNF543	KCNQ1OT1\ZNF543	0,699999928	0,043253969
KCNQ1OT1	ZNF544	KCNQ1OT1\ZNF544	0,783333302	0,017223325
KCNQ1OT1	ZNF562	KCNQ1OT1\ZNF562	0,73333329	0,031123236
KCNQ1OT1	ZNF572	KCNQ1OT1\ZNF572	0,766666651	0,021389991
KCNQ1OT1	ZSCAN31	KCNQ1OT1\ZSCAN31	0,833333313	0,008267196
LINC00174	AGPAT3	LINC00174\AGPAT3	0,799999952	0,013828263
LINC00174	ALDH7A1	LINC00174\ALDH7A1	0,783333302	0,017223325
LINC00174	ANK3	LINC00174\ANK3	0,883333266	0,003075397
LINC00174	ANKFY1	LINC00174\ANKFY1	0,816666603	0,010769401
LINC00174	AP1S1	LINC00174\AP1S1	0,783333302	0,017223325
LINC00174	AP2B1	LINC00174\AP2B1	0,866666615	0,004508378
LINC00174	APOBEC2	LINC00174\APOBEC2	0,916666567	0,001311728
LINC00174	ARL6IP1	LINC00174\ARL6IP1	0,74999994	0,025490521
LINC00174	ARV1	LINC00174\ARV1	0,74999994	0,025490521
LINC00174	ATHL1	LINC00174\ATHL1	0,816666603	0,010769401
LINC00174	ATP2C1	LINC00174\ATP2C1	0,699999928	0,043253969
LINC00174	ATP6AP2	LINC00174\ATP6AP2	0,783333302	0,017223325
LINC00174	ATP6V0B	LINC00174\ATP6V0B	0,716666639	0,036866181
LINC00174	ATP6V1C1	LINC00174\ATP6V1C1	0,783333302	0,017223325
LINC00174	ATRNL1	LINC00174\ATRNL1	0,899999917	0,002028219
LINC00174	BBS4	LINC00174\BBS4	0,799999952	0,013828263
LINC00174	BRD1	LINC00174\BRD1	0,799999952	0,013828263
LINC00174	C15orf41	LINC00174\C15orf41	0,799999952	0,013828263
LINC00174	C19orf82	LINC00174\C19orf82	0,849999964	0,006073633
LINC00174	C1orf109	LINC00174\C1orf109	0,74999994	0,025490521

LINC00174	C5orf15	LINC00174\C5orf15	0,699999928	0,043253969
LINC00174	CALML3	LINC00174\CALML3	0,833333313	0,008267196
LINC00174	CAMKK1	LINC00174\CAMKK1	0,716666639	0,036866181
LINC00174	CAMSAP1	LINC00174\CAMSAP1	0,933333278	0,000749559
LINC00174	CBLN3	LINC00174\CBLN3	0,816666603	0,010769401
LINC00174	CCDC30	LINC00174\CCDC30	0,74999994	0,025490521
LINC00174	CCDC8	LINC00174\CCDC8	0,783333302	0,017223325
LINC00174	CCL25	LINC00174\CCL25	0,766666651	0,021389991
LINC00174	CDH1	LINC00174\CDH1	0,799999952	0,013828263
LINC00174	CDS1	LINC00174\CDS1	0,799999952	0,013828263
LINC00174	CETN3	LINC00174\CETN3	0,766666651	0,021389991
LINC00174	CLDN12	LINC00174\CLDN12	0,949999988	0,000352734
LINC00174	CLINT1	LINC00174\CLINT1	0,699999928	0,043253969
LINC00174	CLTC	LINC00174\CLTC	0,833333313	0,008267196
LINC00174	CNTNAP3	LINC00174\CNTNAP3	0,849999964	0,006073633
LINC00174	CNTNAP3B	LINC00174\CNTNAP3B	0,816666603	0,010769401
LINC00174	COG5	LINC00174\COG5	0,833333313	0,008267196
LINC00174	CROT	LINC00174\CROT	0,916666567	0,001311728
LINC00174	CUX1	LINC00174\CUX1	0,866666615	0,004508378
LINC00174	DACT1	LINC00174\DACT1	0,73333329	0,031123236
LINC00174	DDX31	LINC00174\DDX31	0,73333329	0,031123236
LINC00174	DENND1A	LINC00174\DENND1A	0,74999994	0,025490521
LINC00174	DHTKD1	LINC00174\DHTKD1	0,816666603	0,010769401
LINC00174	DMRT2	LINC00174\DMRT2	0,816666603	0,010769401
LINC00174	DNAI1	LINC00174\DNAI1	0,716666639	0,036866181
LINC00174	DNAJC16	LINC00174\DNAJC16	0,866666615	0,004508378
LINC00174	DPH6	LINC00174\DPH6	0,933333278	0,000749559
LINC00174	DTNB	LINC00174\DTNB	0,766666651	0,021389991
LINC00174	DUOX1	LINC00174\DUOX1	0,93724668	0,000485009
LINC00174	DUOXA1	LINC00174\DUOXA1	0,766666651	0,021389991
LINC00174	EIF2AK1	LINC00174\EIF2AK1	0,73333329	0,031123236
LINC00174	ELOVL6	LINC00174\ELOVL6	0,833333313	0,008267196
LINC00174	EPN3	LINC00174\EPN3	0,866666615	0,004508378
LINC00174	EPS15L1	LINC00174\EPS15L1	0,766666651	0,021389991

LINC00174	ESRP1	LINC00174\ESRP1	0,916666567	0,001311728
LINC00174	ESRP2	LINC00174\ESRP2	0,883333266	0,003075397
LINC00174	EVA1A	LINC00174\EVA1A	0,866666615	0,004508378
LINC00174	EXOC7	LINC00174\EXOC7	0,745869875	0,025859788
LINC00174	EYA2	LINC00174\EYA2	0,916666567	0,001311728
LINC00174	FAHD1	LINC00174\FAHD1	0,866666615	0,004508378
LINC00174	FAM154B	LINC00174\FAM154B	0,84757942	0,00598545
LINC00174	FAM199X	LINC00174\FAM199X	0,766666651	0,021389991
LINC00174	FBXO2	LINC00174\FBXO2	0,744776368	0,026047178
LINC00174	FBXW11	LINC00174\FBXW11	0,695015132	0,044576719
LINC00174	FEM1B	LINC00174\FEM1B	0,766666651	0,021389991
LINC00174	FKTN	LINC00174\FKTN	0,783333302	0,017223325
LINC00174	FLVCR1	LINC00174\FLVCR1	0,73333329	0,031123236
LINC00174	FSTL4	LINC00174\FSTL4	0,933333278	0,000749559
LINC00174	GGA2	LINC00174\GGA2	0,916666567	0,001311728
LINC00174	GINS3	LINC00174\GINS3	0,74999994	0,025490521
LINC00174	GLI2	LINC00174\GLI2	0,98333329	4,96032E-05
LINC00174	GPHN	LINC00174\GPHN	0,899999917	0,002028219
LINC00174	GPR107	LINC00174\GPR107	0,762821436	0,021329366
LINC00174	GPS1	LINC00174\GPS1	0,866666615	0,004508378
LINC00174	GRHL2	LINC00174\GRHL2	0,849999964	0,006073633
LINC00174	GRIP1	LINC00174\GRIP1	0,73333329	0,031123236
LINC00174	GRTP1	LINC00174\GRTP1	0,783333302	0,017223325
LINC00174	GTF2I	LINC00174\GTF2I	0,866666615	0,004508378
LINC00174	GTF3C4	LINC00174\GTF3C4	0,799999952	0,013828263
LINC00174	HDAC4	LINC00174\HDAC4	0,694566727	0,043816138
LINC00174	HIST2H2BF	LINC00174\HIST2H2BF	0,74999994	0,025490521
LINC00174	HN1L	LINC00174\HN1L	0,833333313	0,008267196
LINC00174	HOMER2	LINC00174\HOMER2	0,766666651	0,021389991
LINC00174	HPSE	LINC00174\HPSE	0,73333329	0,031123236
LINC00174	IDH3G	LINC00174\IDH3G	0,699999928	0,043253969
LINC00174	IFI44	LINC00174\IFI44	0,699999928	0,043253969
LINC00174	IQCH	LINC00174\IQCH	0,883333266	0,003075397
LINC00174	ITGA2	LINC00174\ITGA2	0,783333302	0,017223325

LINC00174	KDM4B	LINC00174\KDM4B	0,783333302	0,017223325
LINC00174	KDM4D	LINC00174\KDM4D	0,903773606	0,001598325
LINC00174	KIAA1549	LINC00174\KIAA1549	0,783333302	0,017223325
LINC00174	KLHDC10	LINC00174\KLHDC10	0,783333302	0,017223325
LINC00174	LAMP2	LINC00174\LAMP2	0,878668785	0,003218695
LINC00174	LARS	LINC00174\LARS	0,766666651	0,021389991
LINC00174	LIMK1	LINC00174\LIMK1	0,761512935	0,021263227
LINC00174	LMBR1	LINC00174\LMBR1	0,949999988	0,000352734
LINC00174	LMOD3	LINC00174\LMOD3	0,830627799	0,008300264
LINC00174	LRIG3	LINC00174\LRIG3	0,73333329	0,031123236
LINC00174	LRRC27	LINC00174\LRRC27	0,716666639	0,036866181
LINC00174	M1AP	LINC00174\M1AP	0,966666639	0,000165344
LINC00174	MAML3	LINC00174\MAML3	0,883333266	0,003075397
LINC00174	MAP3K2	LINC00174\MAP3K2	0,783333302	0,017223325
LINC00174	MARVELD2	LINC00174\MARVELD2	0,833333313	0,008267196
LINC00174	MAST4	LINC00174\MAST4	0,744776368	0,026047178
LINC00174	MCOLN3	LINC00174\MCOLN3	0,799999952	0,013828263
LINC00174	MCTP2	LINC00174\MCTP2	0,783333302	0,017223325
LINC00174	METTL8	LINC00174\METTL8	0,833333313	0,008267196
LINC00174	MFSD3	LINC00174\MFSD3	0,949999988	0,000352734
LINC00174	MOV10	LINC00174\MOV10	0,716666639	0,036866181
LINC00174	MPP5	LINC00174\MPP5	0,783333302	0,017223325
LINC00174	MTPN	LINC00174\MTPN	0,728039861	0,031283069
LINC00174	MTSS1L	LINC00174\MTSS1L	0,783333302	0,017223325
LINC00174	MYO10	LINC00174\MYO10	0,916666567	0,001311728
LINC00174	N4BP1	LINC00174\N4BP1	0,866666615	0,004508378
LINC00174	NAGK	LINC00174\NAGK	0,849999964	0,006073633
LINC00174	NCS1	LINC00174\NCS1	0,866666615	0,004508378
LINC00174	NDUFA4	LINC00174\NDUFA4	0,816666603	0,010769401
LINC00174	NETO2	LINC00174\NETO2	0,833333313	0,008267196
LINC00174	NIPSNAP1	LINC00174\NIPSNAP1	0,716666639	0,036866181
LINC00174	NRCAM	LINC00174\NRCAM	0,816666603	0,010769401
LINC00174	NTF4	LINC00174\NTF4	0,716666639	0,036866181
LINC00174	NUDT12	LINC00174\NUDT12	0,719671547	0,033763226

LINC00174	NXN	LINC00174\NXN	0,694566727	0,043816138
LINC00174	OSBPL3	LINC00174\OSBPL3	0,833333313	0,008267196
LINC00174	OXCT1	LINC00174\OXCT1	0,933333278	0,000749559
LINC00174	PABPC4L	LINC00174\PABPC4L	0,783333302	0,017223325
LINC00174	PANK1	LINC00174\PANK1	0,833333313	0,008267196
LINC00174	PASK	LINC00174\PASK	0,883333266	0,003075397
LINC00174	PAX1	LINC00174\PAX1	0,816666603	0,010769401
LINC00174	PAX9	LINC00174\PAX9	0,883333266	0,003075397
LINC00174	PCCB	LINC00174\PCCB	0,766666651	0,021389991
LINC00174	PCTP	LINC00174\PCTP	0,753144681	0,023533951
LINC00174	PDPK1	LINC00174\PDPK1	0,74999994	0,025490521
LINC00174	PGAP1	LINC00174\PGAP1	0,816666603	0,010769401
LINC00174	PIFO	LINC00174\PIFO	0,849999964	0,006073633
LINC00174	PKP1	LINC00174\PKP1	0,833333313	0,008267196
LINC00174	PKP4	LINC00174\PKP4	0,849999964	0,006073633
LINC00174	PLA2G12A	LINC00174\PLA2G12A	0,766666651	0,021389991
LINC00174	PLD2	LINC00174\PLD2	0,866666615	0,004508378
LINC00174	PLEK2	LINC00174\PLEK2	0,816666603	0,010769401
LINC00174	PMPCB	LINC00174\PMPCB	0,883333266	0,003075397
LINC00174	PNMAL2	LINC00174\PNMAL2	0,73333329	0,031123236
LINC00174	POLR1A	LINC00174\POLR1A	0,699999928	0,043253969
LINC00174	PPP5C	LINC00174\PPP5C	0,783333302	0,017223325
LINC00174	PRRG4	LINC00174\PRRG4	0,699999928	0,043253969
LINC00174	PRSS8	LINC00174\PRSS8	0,966666639	0,000165344
LINC00174	PSD3	LINC00174\PSD3	0,833333313	0,008267196
LINC00174	PSMC2	LINC00174\PSMC2	0,716666639	0,036866181
LINC00174	PSMD12	LINC00174\PSMD12	0,799999952	0,013828263
LINC00174	PTPRT	LINC00174\PTPRT	0,816666603	0,010769401
LINC00174	RAB14	LINC00174\RAB14	0,866666615	0,004508378
LINC00174	RAB23	LINC00174\RAB23	0,74999994	0,025490521
LINC00174	RAB40C	LINC00174\RAB40C	0,766666651	0,021389991
LINC00174	RAD50	LINC00174\RAD50	0,783333302	0,017223325
LINC00174	RASL10A	LINC00174\RASL10A	0,928878427	0,000815697
LINC00174	RFESD	LINC00174\RFESD	0,833333313	0,008267196

LINC00174	RMDN3	LINC00174\RMDN3	0,861932218	0,004376102
LINC00174	RNF170	LINC00174\RNF170	0,73333329	0,031123236
LINC00174	RPS6KA6	LINC00174\RPS6KA6	0,73333329	0,031123236
LINC00174	RRAGD	LINC00174\RRAGD	0,766666651	0,021389991
LINC00174	RTF1	LINC00174\RTF1	0,849999964	0,006073633
LINC00174	S100A14	LINC00174\S100A14	0,899999917	0,002028219
LINC00174	SCD5	LINC00174\SCD5	0,699999928	0,043253969
LINC00174	SDR42E1	LINC00174\SDR42E1	0,816666603	0,010769401
LINC00174	SGPL1	LINC00174\SGPL1	0,74999994	0,025490521
LINC00174	SGPP2	LINC00174\SGPP2	0,833333313	0,008267196
LINC00174	SHROOM2	LINC00174\SHROOM2	0,849999964	0,006073633
LINC00174	SLC22A5	LINC00174\SLC22A5	0,899999917	0,002028219
LINC00174	SLC30A1	LINC00174\SLC30A1	0,73333329	0,031123236
LINC00174	SLC44A3	LINC00174\SLC44A3	0,849999964	0,006073633
LINC00174	SLC46A1	LINC00174\SLC46A1	0,833333313	0,008267196
LINC00174	SLC6A8	LINC00174\SLC6A8	0,849999964	0,006073633
LINC00174	SLCO5A1	LINC00174\SLCO5A1	0,766666651	0,021389991
LINC00174	SMO	LINC00174\SMO	0,73333329	0,031123236
LINC00174	SNX1	LINC00174\SNX1	0,833333313	0,008267196
LINC00174	SOGA2	LINC00174\SOGA2	0,883333266	0,003075397
LINC00174	SORBS2	LINC00174\SORBS2	0,98333329	4,96032E-05
LINC00174	SORCS2	LINC00174\SORCS2	0,816666603	0,010769401
LINC00174	SPANXN2	LINC00174\SPANXN2	0,699999928	0,043253969
LINC00174	SPATA6	LINC00174\SPATA6	0,766666651	0,021389991
LINC00174	SPINT1	LINC00174\SPINT1	0,783333302	0,017223325
LINC00174	SPIRE2	LINC00174\SPIRE2	0,79498601	0,013833774
LINC00174	STEAP2	LINC00174\STEAP2	0,783333302	0,017223325
LINC00174	STX6	LINC00174\STX6	0,849999964	0,006073633
LINC00174	SUSD4	LINC00174\SUSD4	0,699999928	0,043253969
LINC00174	SYBU	LINC00174\SYBU	0,966666639	0,000165344
LINC00174	TBC1D2B	LINC00174\TBC1D2B	0,766666651	0,021389991
LINC00174	TDGF1	LINC00174\TDGF1	0,783333302	0,017223325
LINC00174	TM7SF3	LINC00174\TM7SF3	0,816666603	0,010769401
LINC00174	TMEM129	LINC00174\TMEM129	0,79498601	0,013833774

LINC00174	TMEM180	LINC00174\TMEM180	0,728918254	0,031349208
LINC00174	TMEM185A	LINC00174\TMEM185A	0,73333329	0,031123236
LINC00174	TP63	LINC00174\TP63	0,799999952	0,013828263
LINC00174	TRIM7	LINC00174\TRIM7	0,833333313	0,008267196
LINC00174	TRMT10B	LINC00174\TRMT10B	0,716666639	0,036866181
LINC00174	TRPM7	LINC00174\TRPM7	0,933333278	0,000749559
LINC00174	TTC22	LINC00174\TTC22	0,74999994	0,025490521
LINC00174	UBAC1	LINC00174\UBAC1	0,866666615	0,004508378
LINC00174	UBAP2	LINC00174\UBAP2	0,716666639	0,036866181
LINC00174	UBE3C	LINC00174\UBE3C	0,699999928	0,043253969
LINC00174	UBFD1	LINC00174\UBFD1	0,799999952	0,013828263
LINC00174	UNC5B	LINC00174\UNC5B	0,899999917	0,002028219
LINC00174	UPF1	LINC00174\UPF1	0,949999988	0,000352734
LINC00174	USP40	LINC00174\USP40	0,866666615	0,004508378
LINC00174	VAC14	LINC00174\VAC14	0,783333302	0,017223325
LINC00174	WDR91	LINC00174\WDR91	0,833333313	0,008267196
LINC00174	WNK2	LINC00174\WNK2	0,849999964	0,006073633
LINC00174	WTH3DI	LINC00174\WTH3DI	0,74999994	0,025490521
LINC00174	WWOX	LINC00174\WWOX	0,861932218	0,004376102
LINC00174	XYLT2	LINC00174\XYLT2	0,883333266	0,003075397
LINC00174	ZBTB41	LINC00174\ZBTB41	0,833333313	0,008267196
LINC00174	ZNF132	LINC00174\ZNF132	0,849999964	0,006073633
LINC00174	ZNF280B	LINC00174\ZNF280B	0,849999964	0,006073633
LINC00174	ZNF449	LINC00174\ZNF449	0,719671547	0,033763226
LINC00174	ZNF543	LINC00174\ZNF543	0,716666639	0,036866181
LINC00174	ZNF544	LINC00174\ZNF544	0,699999928	0,043253969
LINC00174	ZNF554	LINC00174\ZNF554	0,779773057	0,016765874
LINC00491	ABLIM2	LINC00491\ABLIM2	0,883333266	0,003075397
LINC00491	ALDH7A1	LINC00491\ALDH7A1	0,799999952	0,013828263
LINC00491	ALS2	LINC00491\ALS2	0,799999952	0,013828263
LINC00491	AUTS2	LINC00491\AUTS2	0,799999952	0,013828263
LINC00491	AZIN1	LINC00491\AZIN1	0,783333302	0,017223325
LINC00491	BAIAP2	LINC00491\BAIAP2	0,716666639	0,036866181
LINC00491	C14orf39	LINC00491\C14orf39	0,74999994	0,025490521

LINC00491	C4A	LINC00491\C4A	0,833333313	0,008267196
LINC00491	C4B	LINC00491\C4B	0,833333313	0,008267196
LINC00491	CCDC148	LINC00491\CCDC148	0,699999928	0,043253969
LINC00491	CCDC30	LINC00491\CCDC30	0,866666615	0,004508378
LINC00491	CCDC73	LINC00491\CCDC73	0,769881189	0,018992504
LINC00491	CCDC8	LINC00491\CCDC8	0,73333329	0,031123236
LINC00491	CDC42BPG	LINC00491\CDC42BPG	0,728039861	0,031283069
LINC00491	CLHC1	LINC00491\CLHC1	0,833333313	0,008267196
LINC00491	CLSTN1	LINC00491\CLSTN1	0,766666651	0,021389991
LINC00491	CNKSR3	LINC00491\CNKSR3	0,799999952	0,013828263
LINC00491	COX19	LINC00491\COX19	0,766666651	0,021389991
LINC00491	DDX31	LINC00491\DDX31	0,716666639	0,036866181
LINC00491	DNAJC19	LINC00491\DNAJC19	0,699999928	0,043253969
LINC00491	EDA2R	LINC00491\EDA2R	0,73333329	0,031123236
LINC00491	EMC1	LINC00491\EMC1	0,933333278	0,000749559
LINC00491	ENOSF1	LINC00491\ENOSF1	0,716666639	0,036866181
LINC00491	FAM160A1	LINC00491\FAM160A1	0,766666651	0,021389991
LINC00491	FAM218A	LINC00491\FAM218A	0,716666639	0,036866181
LINC00491	FBXL16	LINC00491\FBXL16	0,833333313	0,008267196
LINC00491	FBXW11	LINC00491\FBXW11	0,711966693	0,038029101
LINC00491	FEM1B	LINC00491\FEM1B	0,74999994	0,025490521
LINC00491	FNBP1L	LINC00491\FNBP1L	0,699999928	0,043253969
LINC00491	GRHL2	LINC00491\GRHL2	0,699999928	0,043253969
LINC00491	GTF2IRD2	LINC00491\GTF2IRD2	0,74999994	0,025490521
LINC00491	IFLTD1	LINC00491\IFLTD1	0,766666651	0,021389991
LINC00491	IFT140	LINC00491\IFT140	0,816666603	0,010769401
LINC00491	IGF1R	LINC00491\IGF1R	0,766666651	0,021389991
LINC00491	IGFBP2	LINC00491\IGFBP2	0,766666651	0,021389991
LINC00491	IGSF11	LINC00491\IGSF11	0,849999964	0,006073633
LINC00491	IKZF5	LINC00491\IKZF5	0,766666651	0,021389991
LINC00491	IQCE	LINC00491\IQCE	0,783333302	0,017223325
LINC00491	KAL1	LINC00491\KAL1	0,766666651	0,021389991
LINC00491	KIAA1644	LINC00491\KIAA1644	0,699999928	0,043253969
LINC00491	KRTAP5-8	LINC00491\KRTAP5-8	0,766666651	0,021389991

LINC00491	KTN1	LINC00491\KTN1	0,73333329	0,031123236
LINC00491	LEPREL1	LINC00491\LEPREL1	0,716666639	0,036866181
LINC00491	LRIG3	LINC00491\LRIG3	0,699999928	0,043253969
LINC00491	LRRC27	LINC00491\LRRC27	0,716666639	0,036866181
LINC00491	LRRK1	LINC00491\LRRK1	0,833333313	0,008267196
LINC00491	MBLAC2	LINC00491\MBLAC2	0,833333313	0,008267196
LINC00491	MCTP2	LINC00491\MCTP2	0,799999952	0,013828263
LINC00491	MYO5B	LINC00491\MYO5B	0,845195651	0,006205908
LINC00491	NAA25	LINC00491\NAA25	0,816666603	0,010769401
LINC00491	NDRG3	LINC00491\NDRG3	0,816666603	0,010769401
LINC00491	NEO1	LINC00491\NEO1	0,766666651	0,021389991
LINC00491	NET1	LINC00491\NET1	0,716666639	0,036866181
LINC00491	NSRP1	LINC00491\NSRP1	0,766666651	0,021389991
LINC00491	NTF4	LINC00491\NTF4	0,716666639	0,036866181
LINC00491	PARD6B	LINC00491\PARD6B	0,98333329	4,96032E-05
LINC00491	PAX1	LINC00491\PAX1	0,766666651	0,021389991
LINC00491	PDGFA	LINC00491\PDGFA	0,73333329	0,031123236
LINC00491	PGAP1	LINC00491\PGAP1	0,783333302	0,017223325
LINC00491	POMT2	LINC00491\POMT2	0,766666651	0,021389991
LINC00491	PRKAA2	LINC00491\PRKAA2	0,73333329	0,031123236
LINC00491	RAB40C	LINC00491\RAB40C	0,73333329	0,031123236
LINC00491	RABGAP1	LINC00491\RABGAP1	0,816666603	0,010769401
LINC00491	RAVER2	LINC00491\RAVER2	0,866666615	0,004508378
LINC00491	RGS9	LINC00491\RGS9	0,799999952	0,013828263
LINC00491	RNF144B	LINC00491\RNF144B	0,866666615	0,004508378
LINC00491	RNF32	LINC00491\RNF32	0,899999917	0,002028219
LINC00491	ROR2	LINC00491\ROR2	0,783333302	0,017223325
LINC00491	RPGRIP1L	LINC00491\RPGRIP1L	0,833333313	0,008267196
LINC00491	RPS6KA6	LINC00491\RPS6KA6	0,866666615	0,004508378
LINC00491	SARS2	LINC00491\SARS2	0,70293504	0,040145501
LINC00491	SDR42E1	LINC00491\SDR42E1	0,716666639	0,036866181
LINC00491	SERBP1	LINC00491\SERBP1	0,716666639	0,036866181
LINC00491	SLC18B1	LINC00491\SLC18B1	0,883333266	0,003075397
LINC00491	SLC22A23	LINC00491\SLC22A23	0,74999994	0,025490521

LINC00491	SLC45A4	LINC00491\SLC45A4	0,74999994	0,025490521
LINC00491	SMO	LINC00491\SMO	0,866666615	0,004508378
LINC00491	SRGAP3	LINC00491\SRGAP3	0,716666639	0,036866181
LINC00491	ST7L	LINC00491\ST7L	0,73333329	0,031123236
LINC00491	STARD7	LINC00491\STARD7	0,699999928	0,043253969
LINC00491	STK36	LINC00491\STK36	0,783333302	0,017223325
LINC00491	SUPT3H	LINC00491\SUPT3H	0,686198473	0,046968695
LINC00491	SYT1	LINC00491\SYT1	0,849999964	0,006073633
LINC00491	TFCP2L1	LINC00491\TFCP2L1	0,783333302	0,017223325
LINC00491	TMEM180	LINC00491\TMEM180	0,830627799	0,007638889
LINC00491	TRMT5	LINC00491\TRMT5	0,833333313	0,008267196
LINC00491	TSPAN13	LINC00491\TSPAN13	0,766666651	0,021389991
LINC00491	UEVLD	LINC00491\UEVLD	0,762821436	0,021329366
LINC00491	USP46	LINC00491\USP46	0,699999928	0,043253969
LINC00491	VPS35	LINC00491\VPS35	0,74999994	0,025490521
LINC00491	ZNF211	LINC00491\ZNF211	0,783333302	0,017223325
LINC00491	ZNF221	LINC00491\ZNF221	0,883333266	0,003075397
LINC00491	ZNF229	LINC00491\ZNF229	0,799999952	0,013828263
LINC00491	ZNF540	LINC00491\ZNF540	0,783333302	0,017223325
LINC00491	ZNF572	LINC00491\ZNF572	0,716666639	0,036866181
LINC00491	ZSCAN31	LINC00491\ZSCAN31	0,833333313	0,008267196
LINC00882	AJUBA	LINC00882\AJUBA	0,903773606	0,001598325
LINC00882	ALDH7A1	LINC00882\ALDH7A1	0,711303294	0,037125219
LINC00882	ANK3	LINC00882\ANK3	0,803354323	0,012202381
LINC00882	AP1S1	LINC00882\AP1S1	0,903773606	0,001598325
LINC00882	AP2B1	LINC00882\AP2B1	0,786617756	0,015288801
LINC00882	APOBEC2	LINC00882\APOBEC2	0,778249502	0,017383156
LINC00882	APP	LINC00882\APP	0,82009083	0,009281305
LINC00882	ARL1	LINC00882\ARL1	0,728039861	0,031283069
LINC00882	ARL6IP1	LINC00882\ARL6IP1	0,778249502	0,017383156
LINC00882	ATP6AP2	LINC00882\ATP6AP2	0,70293504	0,040145501
LINC00882	ATP6V0A1	LINC00882\ATP6V0A1	0,78304255	0,016468255
LINC00882	ATP6V0D1	LINC00882\ATP6V0D1	0,778249502	0,017383156
LINC00882	ATP6V1A	LINC00882\ATP6V1A	0,836827397	0,00696649

LINC00882	ATP6V1C1	LINC00882\ATP6V1C1	0,694566727	0,043816138
LINC00882	ATRNL1	LINC00882\ATRNL1	0,778249502	0,017383156
LINC00882	AUTS2	LINC00882\AUTS2	0,79498601	0,013833774
LINC00882	AZIN1	LINC00882\AZIN1	0,686198473	0,046968695
LINC00882	BAIAP2	LINC00882\BAIAP2	0,79498601	0,013833774
LINC00882	BBS4	LINC00882\BBS4	0,728039861	0,031283069
LINC00882	BRD1	LINC00882\BRD1	0,753144681	0,023533951
LINC00882	C14orf132	LINC00882\C14orf132	0,680672288	0,048820548
LINC00882	C14orf39	LINC00882\C14orf39	0,70293504	0,040145501
LINC00882	C15orf41	LINC00882\C15orf41	0,686198473	0,046968695
LINC00882	C19orf82	LINC00882\C19orf82	0,686198473	0,046968695
LINC00882	C4A	LINC00882\C4A	0,728039861	0,031283069
LINC00882	C4B	LINC00882\C4B	0,728039861	0,031283069
LINC00882	CALR	LINC00882\CALR	0,811722577	0,010769401
LINC00882	CAMSAP1	LINC00882\CAMSAP1	0,694566727	0,043816138
LINC00882	CBLN3	LINC00882\CBLN3	0,761512935	0,021263227
LINC00882	CCDC122	LINC00882\CCDC122	0,861932218	0,004376102
LINC00882	CCDC148	LINC00882\CCDC148	0,694566727	0,043816138
LINC00882	CCDC30	LINC00882\CCDC30	0,811722577	0,010769401
LINC00882	CCDC73	LINC00882\CCDC73	0,722689092	0,03210979
LINC00882	CCDC8	LINC00882\CCDC8	0,811722577	0,010769401
LINC00882	CCL25	LINC00882\CCL25	0,744776368	0,026047178
LINC00882	CDC42BPG	LINC00882\CDC42BPG	0,710084021	0,036342591
LINC00882	CDH1	LINC00882\CDH1	0,853563964	0,005202822
LINC00882	CDIP1	LINC00882\CDIP1	0,887037039	0,002469136
LINC00882	CDKL3	LINC00882\CDKL3	0,736408114	0,02800926
LINC00882	CDKL5	LINC00882\CDKL5	0,803354323	0,012202381
LINC00882	CDS1	LINC00882\CDS1	0,853563964	0,005202822
LINC00882	CETN3	LINC00882\CETN3	0,761512935	0,021263227
LINC00882	CHID1	LINC00882\CHID1	0,744776368	0,026047178
LINC00882	CLCA1	LINC00882\CLCA1	0,786633968	0,013888889
LINC00882	CLDN12	LINC00882\CLDN12	0,836827397	0,00696649
LINC00882	CLSTN1	LINC00882\CLSTN1	0,79498601	0,013833774
LINC00882	CLTC	LINC00882\CLTC	0,736408114	0,02800926

LINC00882	CNTNAP2	LINC00882\CNTNAP2	0,736408114	0,02800926
LINC00882	CNTNAP3	LINC00882\CNTNAP3	0,828459144	0,008289241
LINC00882	CNTNAP3B	LINC00882\CNTNAP3B	0,887037039	0,002469136
LINC00882	COG5	LINC00882\COG5	0,744776368	0,026047178
LINC00882	COL8A2	LINC00882\COL8A2	0,786617756	0,015288801
LINC00882	COX19	LINC00882\COX19	0,711303294	0,037125219
LINC00882	CRTC1	LINC00882\CRTC1	0,920510173	0,000981041
LINC00882	DACT1	LINC00882\DACT1	0,778249502	0,017383156
LINC00882	DCAKD	LINC00882\DCAKD	0,853563964	0,005202822
LINC00882	DDX31	LINC00882\DDX31	0,70293504	0,040145501
LINC00882	DENND1A	LINC00882\DENND1A	0,753144681	0,023533951
LINC00882	DET1	LINC00882\DET1	0,903773606	0,001598325
LINC00882	DHTKD1	LINC00882\DHTKD1	0,711303294	0,037125219
LINC00882	DHX32	LINC00882\DHX32	0,920510173	0,000981041
LINC00882	DMRT2	LINC00882\DMRT2	0,887037039	0,002469136
LINC00882	DMRTA1	LINC00882\DMRTA1	0,719671547	0,033763226
LINC00882	DNAJC19	LINC00882\DNAJC19	0,753144681	0,023533951
LINC00882	DNAJC21	LINC00882\DNAJC21	0,694566727	0,043816138
LINC00882	DNAL4	LINC00882\DNAL4	0,878668785	0,003218695
LINC00882	DPH6	LINC00882\DPH6	0,853563964	0,005202822
LINC00882	DSG2	LINC00882\DSG2	0,744776368	0,026047178
LINC00882	DUOX1	LINC00882\DUOX1	0,80252099	0,01151896
LINC00882	DUOXA1	LINC00882\DUOXA1	0,962351501	0,000165344
LINC00882	EDA2R	LINC00882\EDA2R	0,811722577	0,010769401
LINC00882	EFNB2	LINC00882\EFNB2	0,811722577	0,010769401
LINC00882	EIF2AK4	LINC00882\EIF2AK4	0,836827397	0,00696649
LINC00882	EMC10	LINC00882\EMC10	0,861344516	0,004332011
LINC00882	ENPEP	LINC00882\ENPEP	0,744776368	0,026047178
LINC00882	EPN3	LINC00882\EPN3	0,82009083	0,009281305
LINC00882	EPS15L1	LINC00882\EPS15L1	0,728039861	0,031283069
LINC00882	ESRP1	LINC00882\ESRP1	0,761512935	0,021263227
LINC00882	ESRP2	LINC00882\ESRP2	0,778249502	0,017383156
LINC00882	EVA1A	LINC00882\EVA1A	0,845195651	0,006205908
LINC00882	EYA2	LINC00882\EYA2	0,79498601	0,013833774

LINC00882	FAHD1	LINC00882\FAHD1	0,895405293	0,002105379
LINC00882	FAM154B	LINC00882\FAM154B	0,731974542	0,029298943
LINC00882	FAM160A1	LINC00882\FAM160A1	0,778249502	0,017383156
LINC00882	FAM163A	LINC00882\FAM163A	0,711303294	0,037125219
LINC00882	FAM199X	LINC00882\FAM199X	0,803354323	0,012202381
LINC00882	FAM218A	LINC00882\FAM218A	0,811722577	0,010769401
LINC00882	FAM83B	LINC00882\FAM83B	0,861932218	0,004376102
LINC00882	FBXO22	LINC00882\FBXO22	0,693277299	0,042779982
LINC00882	FEM1B	LINC00882\FEM1B	0,778249502	0,017383156
LINC00882	FKTN	LINC00882\FKTN	0,70293504	0,040145501
LINC00882	FNBP1L	LINC00882\FNBP1L	0,711303294	0,037125219
LINC00882	GGA1	LINC00882\GGA1	0,93724668	0,000485009
LINC00882	GLI2	LINC00882\GLI2	0,744776368	0,026047178
LINC00882	GNPDA1	LINC00882\GNPDA1	0,928878427	0,000815697
LINC00882	GOLM1	LINC00882\GOLM1	0,761512935	0,021263227
LINC00882	GPC4	LINC00882\GPC4	0,845195651	0,006205908
LINC00882	GPR107	LINC00882\GPR107	0,791553855	0,013194445
LINC00882	GPR64	LINC00882\GPR64	0,82009083	0,009281305
LINC00882	GRHL2	LINC00882\GRHL2	0,728039861	0,031283069
LINC00882	GRIP1	LINC00882\GRIP1	0,786617756	0,015288801
LINC00882	GRTP1	LINC00882\GRTP1	0,870300472	0,003681658
LINC00882	GTF3C4	LINC00882\GTF3C4	0,769881189	0,018992504
LINC00882	GXYLT1	LINC00882\GXYLT1	0,694566727	0,043816138
LINC00882	HDGFRP3	LINC00882\HDGFRP3	0,786617756	0,015288801
LINC00882	HIST2H2BF	LINC00882\HIST2H2BF	0,70293504	0,040145501
LINC00882	HN1L	LINC00882\HN1L	0,728039861	0,031283069
LINC00882	HOMER2	LINC00882\HOMER2	0,719671547	0,033763226
LINC00882	HOMER3	LINC00882\HOMER3	0,853563964	0,005202822
LINC00882	HPS5	LINC00882\HPS5	0,861932218	0,004376102
LINC00882	HPSE	LINC00882\HPSE	0,761512935	0,021263227
LINC00882	IDH3G	LINC00882\IDH3G	0,786617756	0,015288801
LINC00882	IFI44	LINC00882\IFI44	0,711303294	0,037125219
LINC00882	IGDCC3	LINC00882\IGDCC3	0,719671547	0,033763226
LINC00882	IGSF3	LINC00882\IGSF3	0,803354323	0,012202381

LINC00882	IKBIP	LINC00882\IKBIP	0,845195651	0,006205908
LINC00882	IQCH	LINC00882\IQCH	0,711303294	0,037125219
LINC00882	ITGA2	LINC00882\ITGA2	0,870300472	0,003681658
LINC00882	ITGAV	LINC00882\ITGAV	0,79498601	0,013833774
LINC00882	KAL1	LINC00882\KAL1	0,694566727	0,043816138
LINC00882	KCTD1	LINC00882\KCTD1	0,803354323	0,012202381
LINC00882	KDM4D	LINC00882\KDM4D	0,785714269	0,014925044
LINC00882	KIAA0319L	LINC00882\KIAA0319L	0,887037039	0,002469136
LINC00882	KIAA1549	LINC00882\KIAA1549	0,694566727	0,043816138
LINC00882	KLHDC10	LINC00882\KLHDC10	0,753144681	0,023533951
LINC00882	KRTAP5-8	LINC00882\KRTAP5-8	0,744776368	0,026047178
LINC00882	LACC1	LINC00882\LACC1	0,861344516	0,004354056
LINC00882	LAMP1	LINC00882\LAMP1	0,915966392	0,001069224
LINC00882	LAMP2	LINC00882\LAMP2	0,789915979	0,013712522
LINC00882	LAMP5	LINC00882\LAMP5	0,686198473	0,046968695
LINC00882	LARS	LINC00882\LARS	0,728039861	0,031283069
LINC00882	LEPREL1	LINC00882\LEPREL1	0,769881189	0,018992504
LINC00882	LGI3	LINC00882\LGI3	0,728039861	0,031283069
LINC00882	LIMK1	LINC00882\LIMK1	0,722689092	0,031856261
LINC00882	LMBR1	LINC00882\LMBR1	0,778249502	0,017383156
LINC00882	LPHN3	LINC00882\LPHN3	0,778249502	0,017383156
LINC00882	LPIN1	LINC00882\LPIN1	0,744776368	0,026047178
LINC00882	LRIG3	LINC00882\LRIG3	0,79498601	0,013833774
LINC00882	M1AP	LINC00882\M1AP	0,811722577	0,010769401
LINC00882	MAGEA3	LINC00882\MAGEA3	0,861932218	0,004376102
LINC00882	MAML3	LINC00882\MAML3	0,711303294	0,037125219
LINC00882	MAP3K2	LINC00882\MAP3K2	0,70293504	0,040145501
LINC00882	MAPK1	LINC00882\MAPK1	0,747899175	0,023919754
LINC00882	MARVELD2	LINC00882\MARVELD2	0,878668785	0,003218695
LINC00882	MAST4	LINC00882\MAST4	0,810924351	0,010306437
LINC00882	MCOLN3	LINC00882\MCOLN3	0,769881189	0,018992504
LINC00882	MCTP2	LINC00882\MCTP2	0,728039861	0,031283069
LINC00882	MED21	LINC00882\MED21	0,768907547	0,018628748
LINC00882	MED22	LINC00882\MED22	0,885178506	0,002777778

LINC00882	MFSD3	LINC00882\MFSD3	0,728039861	0,031283069
LINC00882	MLX	LINC00882\MLX	0,70588237	0,037985008
LINC00882	MOV10	LINC00882\MOV10	0,811722577	0,010769401
LINC00882	MPP5	LINC00882\MPP5	0,744776368	0,026047178
LINC00882	MRPL40	LINC00882\MRPL40	0,711303294	0,037125219
LINC00882	MST1L	LINC00882\MST1L	0,736408114	0,02800926
LINC00882	MYLK4	LINC00882\MYLK4	0,686198473	0,046968695
LINC00882	MYO10	LINC00882\MYO10	0,803354323	0,012202381
LINC00882	N4BP1	LINC00882\N4BP1	0,91214186	0,001322751
LINC00882	N4BP2L2	LINC00882\N4BP2L2	0,786617756	0,015288801
LINC00882	NAGK	LINC00882\NAGK	0,786617756	0,015288801
LINC00882	NCS1	LINC00882\NCS1	0,836827397	0,00696649
LINC00882	NDRG3	LINC00882\NDRG3	0,694566727	0,043816138
LINC00882	NDUFA4	LINC00882\NDUFA4	0,836827397	0,00696649
LINC00882	NEO1	LINC00882\NEO1	0,778249502	0,017383156
LINC00882	NET1	LINC00882\NET1	0,736408114	0,02800926
LINC00882	NETO2	LINC00882\NETO2	0,778249502	0,017383156
LINC00882	NHLRC3	LINC00882\NHLRC3	0,870300472	0,003681658
LINC00882	NIPSNAP1	LINC00882\NIPSNAP1	0,711303294	0,037125219
LINC00882	NISCH	LINC00882\NISCH	0,728039861	0,031283069
LINC00882	NPNT	LINC00882\NPNT	0,887037039	0,002469136
LINC00882	NRCAM	LINC00882\NRCAM	0,887037039	0,002469136
LINC00882	NTF4	LINC00882\NTF4	0,736408114	0,02800926
LINC00882	NUDT12	LINC00882\NUDT12	0,69747901	0,041347001
LINC00882	NUDT9	LINC00882\NUDT9	0,70293504	0,040145501
LINC00882	NXN	LINC00882\NXN	0,949579835	0,000275573
LINC00882	OAT	LINC00882\OAT	0,79498601	0,013833774
LINC00882	OSBPL3	LINC00882\OSBPL3	0,744776368	0,026047178
LINC00882	OXCT1	LINC00882\OXCT1	0,736408114	0,02800926
LINC00882	PABPC4L	LINC00882\PABPC4L	0,778249502	0,017383156
LINC00882	PANK1	LINC00882\PANK1	0,786617756	0,015288801
LINC00882	PAX1	LINC00882\PAX1	0,728039861	0,031283069
LINC00882	PAX9	LINC00882\PAX9	0,803354323	0,012202381
LINC00882	PCSK6	LINC00882\PCSK6	0,744776368	0,026047178

LINC00882	PDGFA	LINC00882\PDGFA	0,811722577	0,010769401
LINC00882	PGAP1	LINC00882\PGAP1	0,686198473	0,046968695
LINC00882	PIAS2	LINC00882\PIAS2	0,91214186	0,001322751
LINC00882	PIFO	LINC00882\PIFO	0,811722577	0,010769401
LINC00882	PKP1	LINC00882\PKP1	0,878668785	0,003218695
LINC00882	PKP4	LINC00882\PKP4	0,82009083	0,009281305
LINC00882	PLA2G12A	LINC00882\PLA2G12A	0,811722577	0,010769401
LINC00882	PLD2	LINC00882\PLD2	0,836827397	0,00696649
LINC00882	PLEK2	LINC00882\PLEK2	0,694566727	0,043816138
LINC00882	PMPCB	LINC00882\PMPCB	0,761512935	0,021263227
LINC00882	PNMAL1	LINC00882\PNMAL1	0,728039861	0,031283069
LINC00882	PNMAL2	LINC00882\PNMAL2	0,719671547	0,033763226
LINC00882	POGZ	LINC00882\POGZ	0,736408114	0,02800926
LINC00882	POMT2	LINC00882\POMT2	0,811722577	0,010769401
LINC00882	PRKAA2	LINC00882\PRKAA2	0,694566727	0,043816138
LINC00882	PRKAB1	LINC00882\PRKAB1	0,79498601	0,013833774
LINC00882	PRKAR1A	LINC00882\PRKAR1A	0,706440568	0,037334654
LINC00882	PROP1	LINC00882\PROP1	0,753144681	0,023533951
LINC00882	PRRC1	LINC00882\PRRC1	0,686198473	0,046968695
LINC00882	PRRG4	LINC00882\PRRG4	0,928878427	0,000815697
LINC00882	PRSS8	LINC00882\PRSS8	0,719671547	0,033763226
LINC00882	PSAT1	LINC00882\PSAT1	0,878668785	0,003218695
LINC00882	PSD3	LINC00882\PSD3	0,803354323	0,012202381
LINC00882	PSMD12	LINC00882\PSMD12	0,728039861	0,031283069
LINC00882	PTPRT	LINC00882\PTPRT	0,694566727	0,043816138
LINC00882	RAB14	LINC00882\RAB14	0,928878427	0,000815697
LINC00882	RAB23	LINC00882\RAB23	0,878668785	0,003218695
LINC00882	RABGAP1	LINC00882\RABGAP1	0,761512935	0,021263227
LINC00882	RAD50	LINC00882\RAD50	0,70293504	0,040145501
LINC00882	RANBP17	LINC00882\RANBP17	0,786617756	0,015288801
LINC00882	RASEF	LINC00882\RASEF	0,728039861	0,031283069
LINC00882	RHBDD2	LINC00882\RHBDD2	0,953983247	0,000253527
LINC00882	RMDN3	LINC00882\RMDN3	0,760504186	0,020712081
LINC00882	RNF170	LINC00882\RNF170	0,853563964	0,005202822

LINC00882	RNF212	LINC00882\RNF212	0,753144681	0,023533951
LINC00882	RP9	LINC00882\RP9	0,694566727	0,043816138
LINC00882	RRM2B	LINC00882\RRM2B	0,836827397	0,00696649
LINC00882	RTF1	LINC00882\RTF1	0,686198473	0,046968695
LINC00882	S100A14	LINC00882\S100A14	0,778249502	0,017383156
LINC00882	SCAMP1	LINC00882\SCAMP1	0,82009083	0,009281305
LINC00882	SCD5	LINC00882\SCD5	0,753144681	0,023533951
LINC00882	SCN4B	LINC00882\SCN4B	0,803354323	0,012202381
LINC00882	SDR42E1	LINC00882\SDR42E1	0,786617756	0,015288801
LINC00882	SEC61A1	LINC00882\SEC61A1	0,803354323	0,012202381
LINC00882	SERPINB5	LINC00882\SERPINB5	0,769881189	0,018992504
LINC00882	SGPL1	LINC00882\SGPL1	0,928878427	0,000815697
LINC00882	SHROOM2	LINC00882\SHROOM2	0,694566727	0,043816138
LINC00882	SIX1	LINC00882\SIX1	0,853563964	0,005202822
LINC00882	SIX4	LINC00882\SIX4	0,870300472	0,003681658
LINC00882	SLC22A5	LINC00882\SLC22A5	0,803354323	0,012202381
LINC00882	SLC30A1	LINC00882\SLC30A1	0,79498601	0,013833774
LINC00882	SLC44A3	LINC00882\SLC44A3	0,694566727	0,043816138
LINC00882	SLC46A1	LINC00882\SLC46A1	0,887037039	0,002469136
LINC00882	SLCO5A1	LINC00882\SLCO5A1	0,736408114	0,02800926
LINC00882	SORBS2	LINC00882\SORBS2	0,79498601	0,013833774
LINC00882	SORCS1	LINC00882\SORCS1	0,728039861	0,031283069
LINC00882	SPATA6	LINC00882\SPATA6	0,736408114	0,02800926
LINC00882	SPIN1	LINC00882\SPIN1	0,870300472	0,003681658
LINC00882	SPINT1	LINC00882\SPINT1	0,870300472	0,003681658
LINC00882	SPIRE2	LINC00882\SPIRE2	0,739495814	0,026587302
LINC00882	STEAP2	LINC00882\STEAP2	0,811722577	0,010769401
LINC00882	STON2	LINC00882\STON2	0,861932218	0,004376102
LINC00882	SUFU	LINC00882\SUFU	0,778249502	0,017383156
LINC00882	SUPT3H	LINC00882\SUPT3H	0,69747901	0,041347001
LINC00882	SYBU	LINC00882\SYBU	0,803354323	0,012202381
LINC00882	TAB3	LINC00882\TAB3	0,811722577	0,010769401
LINC00882	TCEA2	LINC00882\TCEA2	0,728039861	0,031283069
LINC00882	TDGF1	LINC00882\TDGF1	0,711303294	0,037125219

LINC00882	TM7SF3	LINC00882\TM7SF3	0,79498601	0,013833774
LINC00882	TMEM107	LINC00882\TMEM107	0,79498601	0,013833774
LINC00882	TMEM133	LINC00882\TMEM133	0,870300472	0,003681658
LINC00882	TMEM180	LINC00882\TMEM180	0,706440568	0,037334654
LINC00882	TMEM185A	LINC00882\TMEM185A	0,903773606	0,001598325
LINC00882	TMEM30B	LINC00882\TMEM30B	0,895405293	0,002105379
LINC00882	TNPO1	LINC00882\TNPO1	0,878668785	0,003218695
LINC00882	TOMM20	LINC00882\TOMM20	0,878668785	0,003218695
LINC00882	TP63	LINC00882\TP63	0,686198473	0,046968695
LINC00882	TRPM7	LINC00882\TRPM7	0,744776368	0,026047178
LINC00882	TTC22	LINC00882\TTC22	0,845195651	0,006205908
LINC00882	TXNL1	LINC00882\TXNL1	0,811722577	0,010769401
LINC00882	UBTD2	LINC00882\UBTD2	0,844537795	0,005919312
LINC00882	UEVLD	LINC00882\UEVLD	0,740485847	0,025892857
LINC00882	UMPS	LINC00882\UMPS	0,731974542	0,029464286
LINC00882	UNC5B	LINC00882\UNC5B	0,861932218	0,004376102
LINC00882	UNG	LINC00882\UNG	0,711303294	0,037125219
LINC00882	UPF1	LINC00882\UPF1	0,719671547	0,033763226
LINC00882	USP28	LINC00882\USP28	0,811722577	0,010769401
LINC00882	USP46	LINC00882\USP46	0,719671547	0,033763226
LINC00882	VMA21	LINC00882\VMA21	0,769881189	0,018992504
LINC00882	VWCE	LINC00882\VWCE	0,719671547	0,033763226
LINC00882	WASL	LINC00882\WASL	0,945614994	0,00037478
LINC00882	WDR61	LINC00882\WDR61	0,80252099	0,011761464
LINC00882	WDR91	LINC00882\WDR91	0,728039861	0,031283069
LINC00882	WNK2	LINC00882\WNK2	0,778249502	0,017383156
LINC00882	WTH3DI	LINC00882\WTH3DI	0,728039861	0,031283069
LINC00882	XPR1	LINC00882\XPR1	0,769881189	0,018992504
LINC00882	ZBTB41	LINC00882\ZBTB41	0,861932218	0,004376102
LINC00882	ZNF280B	LINC00882\ZNF280B	0,853563964	0,005202822
LINC00882	ZNF449	LINC00882\ZNF449	0,907563031	0,001311728
OIP5-AS1	ACAD10	OIP5-AS1\ACAD10	0,819327712	0,009336419
OIP5-AS1	AFG3L2	OIP5-AS1\AFG3L2	0,736408114	0,02800926
OIP5-AS1	AK3	OIP5-AS1\AK3	0,870300472	0,003681658

OIP5-AS1	ANKFY1	OIP5-AS1\ANKFY1	0,828459144	0,008289241
OIP5-AS1	ANKMY2	OIP5-AS1\ANKMY2	0,845195651	0,006205908
OIP5-AS1	APOBEC2	OIP5-AS1\APOBEC2	0,769881189	0,018992504
OIP5-AS1	APTX	OIP5-AS1\APTX	0,93724668	0,000485009
OIP5-AS1	ARL6IP1	OIP5-AS1\ARL6IP1	0,853563964	0,005202822
OIP5-AS1	ARV1	OIP5-AS1\ARV1	0,895405293	0,002105379
OIP5-AS1	ATP2C1	OIP5-AS1\ATP2C1	0,845195651	0,006205908
OIP5-AS1	ATP6V0B	OIP5-AS1\ATP6V0B	0,803354323	0,012202381
OIP5-AS1	ATP6V1C1	OIP5-AS1\ATP6V1C1	0,895405293	0,002105379
OIP5-AS1	ATPAF1	OIP5-AS1\ATPAF1	0,853563964	0,005202822
OIP5-AS1	ATPIF1	OIP5-AS1\ATPIF1	0,853563964	0,005202822
OIP5-AS1	ATRNL1	OIP5-AS1\ATRNL1	0,686198473	0,046968695
OIP5-AS1	BBS4	OIP5-AS1\BBS4	0,82009083	0,009281305
OIP5-AS1	BTBD3	OIP5-AS1\BTBD3	0,786617756	0,015288801
OIP5-AS1	C15orf41	OIP5-AS1\C15orf41	0,870300472	0,003681658
OIP5-AS1	C19orf82	OIP5-AS1\C19orf82	0,70293504	0,040145501
OIP5-AS1	C1orf109	OIP5-AS1\C1orf109	0,736408114	0,02800926
OIP5-AS1	C1orf27	OIP5-AS1\C1orf27	0,694566727	0,043816138
OIP5-AS1	CAMSAP1	OIP5-AS1\CAMSAP1	0,728039861	0,031283069
OIP5-AS1	CBS	OIP5-AS1\CBS	0,753144681	0,023533951
OIP5-AS1	CCDC30	OIP5-AS1\CCDC30	0,79831934	0,012301587
OIP5-AS1	CD274	OIP5-AS1\CD274	0,686198473	0,046968695
OIP5-AS1	CDS1	OIP5-AS1\CDS1	0,736408114	0,02800926
OIP5-AS1	CETN3	OIP5-AS1\CETN3	0,728039861	0,031283069
OIP5-AS1	CLDN12	OIP5-AS1\CLDN12	0,719671547	0,033763226
OIP5-AS1	CLINT1	OIP5-AS1\CLINT1	0,93724668	0,000485009
OIP5-AS1	CLTC	OIP5-AS1\CLTC	0,686198473	0,046968695
OIP5-AS1	CMPK2	OIP5-AS1\CMPK2	0,870300472	0,003681658
OIP5-AS1	COG5	OIP5-AS1\COG5	0,761512935	0,021263227
OIP5-AS1	CROT	OIP5-AS1\CROT	0,744776368	0,026047178
OIP5-AS1	CSRP2BP	OIP5-AS1\CSRP2BP	0,903773606	0,001598325
OIP5-AS1	CUX1	OIP5-AS1\CUX1	0,811722577	0,010769401
OIP5-AS1	DHTKD1	OIP5-AS1\DHTKD1	0,836827397	0,00696649
OIP5-AS1	DNAJC16	OIP5-AS1\DNAJC16	0,769881189	0,018992504

OIP5-AS1	EIF2AK1	OIP5-AS1\EIF2AK1	0,811722577	0,010769401
OIP5-AS1	ELOVL6	OIP5-AS1\ELOVL6	0,870300472	0,003681658
OIP5-AS1	ENPEP	OIP5-AS1\ENPEP	0,728039861	0,031283069
OIP5-AS1	FAF2	OIP5-AS1\FAF2	0,803354323	0,012202381
OIP5-AS1	FAM120A	OIP5-AS1\FAM120A	0,920510173	0,000981041
OIP5-AS1	FAM199X	OIP5-AS1\FAM199X	0,786617756	0,015288801
OIP5-AS1	FAM45A	OIP5-AS1\FAM45A	0,728039861	0,031283069
OIP5-AS1	FBXO22	OIP5-AS1\FBXO22	0,848739505	0,005169753
OIP5-AS1	FKTN	OIP5-AS1\FKTN	0,887037039	0,002469136
OIP5-AS1	FLVCR1	OIP5-AS1\FLVCR1	0,878668785	0,003218695
OIP5-AS1	GGA2	OIP5-AS1\GGA2	0,79498601	0,013833774
OIP5-AS1	GGCT	OIP5-AS1\GGCT	0,828459144	0,008289241
OIP5-AS1	GINS3	OIP5-AS1\GINS3	0,769881189	0,018992504
OIP5-AS1	GNPDA2	OIP5-AS1\GNPDA2	0,803354323	0,012202381
OIP5-AS1	GPS1	OIP5-AS1\GPS1	0,828459144	0,008289241
OIP5-AS1	GTF2H3	OIP5-AS1\GTF2H3	0,853563964	0,005202822
OIP5-AS1	GTF2H4	OIP5-AS1\GTF2H4	0,686198473	0,046968695
OIP5-AS1	GTF2I	OIP5-AS1\GTF2I	0,728039861	0,031283069
OIP5-AS1	HIST1H2BC	OIP5-AS1\HIST1H2BC	0,836827397	0,00696649
OIP5-AS1	HIST2H2BF	OIP5-AS1\HIST2H2BF	0,895405293	0,002105379
OIP5-AS1	HOMER2	OIP5-AS1\HOMER2	0,870300472	0,003681658
OIP5-AS1	HPSE	OIP5-AS1\HPSE	0,769881189	0,018992504
OIP5-AS1	IDH3G	OIP5-AS1\IDH3G	0,686198473	0,046968695
OIP5-AS1	KDM4A	OIP5-AS1\KDM4A	0,70293504	0,040145501
OIP5-AS1	KDM4D	OIP5-AS1\KDM4D	0,726890743	0,030500442
OIP5-AS1	KIAA1549	OIP5-AS1\KIAA1549	0,895405293	0,002105379
OIP5-AS1	KIF3A	OIP5-AS1\KIF3A	0,70293504	0,040145501
OIP5-AS1	KLHDC10	OIP5-AS1\KLHDC10	0,79498601	0,013833774
OIP5-AS1	LAMP2	OIP5-AS1\LAMP2	0,70168066	0,03957231
OIP5-AS1	LARS	OIP5-AS1\LARS	0,719671547	0,033763226
OIP5-AS1	LIMK1	OIP5-AS1\LIMK1	0,777310908	0,017063493
OIP5-AS1	LRPPRC	OIP5-AS1\LRPPRC	0,828459144	0,008289241
OIP5-AS1	LZIC	OIP5-AS1\LZIC	0,711303294	0,037125219
OIP5-AS1	MAML3	OIP5-AS1\MAML3	0,836827397	0,00696649

OIP5-AS1	MAP3K2	OIP5-AS1\MAP3K2	0,887037039	0,002469136
OIP5-AS1	MCOLN3	OIP5-AS1\MCOLN3	0,82009083	0,009281305
OIP5-AS1	MPP5	OIP5-AS1\MPP5	0,803354323	0,012202381
OIP5-AS1	MTPN	OIP5-AS1\MTPN	0,80252099	0,011761464
OIP5-AS1	MTSS1L	OIP5-AS1\MTSS1L	0,744776368	0,026047178
OIP5-AS1	NDUFA4	OIP5-AS1\NDUFA4	0,753144681	0,023533951
OIP5-AS1	NDUFB5	OIP5-AS1\NDUFB5	0,920510173	0,000981041
OIP5-AS1	NETO2	OIP5-AS1\NETO2	0,769881189	0,018992504
OIP5-AS1	NIF3L1	OIP5-AS1\NIF3L1	0,728039861	0,031283069
OIP5-AS1	NIPSNAP1	OIP5-AS1\NIPSNAP1	0,861932218	0,004376102
OIP5-AS1	NRCAM	OIP5-AS1\NRCAM	0,744776368	0,026047178
OIP5-AS1	NUDT12	OIP5-AS1\NUDT12	0,907563031	0,001311728
OIP5-AS1	OSBPL3	OIP5-AS1\OSBPL3	0,686198473	0,046968695
OIP5-AS1	OXCT1	OIP5-AS1\OXCT1	0,728039861	0,031283069
OIP5-AS1	PANK1	OIP5-AS1\PANK1	0,761512935	0,021263227
OIP5-AS1	PASK	OIP5-AS1\PASK	0,845195651	0,006205908
OIP5-AS1	PLA2G12A	OIP5-AS1\PLA2G12A	0,719671547	0,033763226
OIP5-AS1	PLEK2	OIP5-AS1\PLEK2	0,769881189	0,018992504
OIP5-AS1	PMPCB	OIP5-AS1\PMPCB	0,828459144	0,008289241
OIP5-AS1	PNPT1	OIP5-AS1\PNPT1	0,853563964	0,005202822
OIP5-AS1	PRMT5	OIP5-AS1\PRMT5	0,694566727	0,043816138
OIP5-AS1	PRRC1	OIP5-AS1\PRRC1	0,853563964	0,005202822
OIP5-AS1	PSMC2	OIP5-AS1\PSMC2	0,786617756	0,015288801
OIP5-AS1	PSMD12	OIP5-AS1\PSMD12	0,82009083	0,009281305
OIP5-AS1	PSMD5	OIP5-AS1\PSMD5	0,785714269	0,015531305
OIP5-AS1	PTPRK	OIP5-AS1\PTPRK	0,711303294	0,037125219
OIP5-AS1	RAB40C	OIP5-AS1\RAB40C	0,728039861	0,031283069
OIP5-AS1	RAD50	OIP5-AS1\RAD50	0,887037039	0,002469136
OIP5-AS1	RBM23	OIP5-AS1\RBM23	0,686198473	0,046968695
OIP5-AS1	RFESD	OIP5-AS1\RFESD	0,870300472	0,003681658
OIP5-AS1	RMDN3	OIP5-AS1\RMDN3	0,714285731	0,034887567
OIP5-AS1	RRAGB	OIP5-AS1\RRAGB	0,887037039	0,002469136
OIP5-AS1	RTF1	OIP5-AS1\RTF1	0,778249502	0,017383156
OIP5-AS1	S100A14	OIP5-AS1\S100A14	0,686198473	0,046968695

OIP5-AS1	SCCPDH	OIP5-AS1\SCCPDH	0,79498601	0,013833774
OIP5-AS1	SLC30A6	OIP5-AS1\SLC30A6	0,828459144	0,008289241
OIP5-AS1	SLC44A3	OIP5-AS1\SLC44A3	0,736408114	0,02800926
OIP5-AS1	SNUPN	OIP5-AS1\SNUPN	0,70293504	0,040145501
OIP5-AS1	SNX1	OIP5-AS1\SNX1	0,786617756	0,015288801
OIP5-AS1	SPIRE2	OIP5-AS1\SPIRE2	0,718487382	0,033664022
OIP5-AS1	SPRY3	OIP5-AS1\SPRY3	0,887037039	0,002469136
OIP5-AS1	STX6	OIP5-AS1\STX6	0,803354323	0,012202381
OIP5-AS1	SUSD4	OIP5-AS1\SUSD4	0,728039861	0,031283069
OIP5-AS1	TCEB3	OIP5-AS1\TCEB3	0,79498601	0,013833774
OIP5-AS1	TRIM14	OIP5-AS1\TRIM14	0,736408114	0,02800926
OIP5-AS1	TRIM52	OIP5-AS1\TRIM52	0,828459144	0,008289241
OIP5-AS1	TRIM61	OIP5-AS1\TRIM61	0,753144681	0,023533951
OIP5-AS1	TRPM7	OIP5-AS1\TRPM7	0,719671547	0,033763226
OIP5-AS1	TRUB1	OIP5-AS1\TRUB1	0,91214186	0,001322751
OIP5-AS1	TTC22	OIP5-AS1\TTC22	0,711303294	0,037125219
OIP5-AS1	UBAP2	OIP5-AS1\UBAP2	0,803354323	0,012202381
OIP5-AS1	UBE3A	OIP5-AS1\UBE3A	0,70293504	0,040145501
OIP5-AS1	UBE3C	OIP5-AS1\UBE3C	0,803354323	0,012202381
OIP5-AS1	UBFD1	OIP5-AS1\UBFD1	0,91214186	0,001322751
OIP5-AS1	URB2	OIP5-AS1\URB2	0,719671547	0,033763226
OIP5-AS1	VAC14	OIP5-AS1\VAC14	0,945614994	0,00037478
OIP5-AS1	VANGL1	OIP5-AS1\VANGL1	0,887037039	0,002469136
OIP5-AS1	VMA21	OIP5-AS1\VMA21	0,853563964	0,005202822
OIP5-AS1	WDR3	OIP5-AS1\WDR3	0,778249502	0,017383156
OIP5-AS1	WWOX	OIP5-AS1\WWOX	0,710084021	0,036342591
OIP5-AS1	XPO7	OIP5-AS1\XPO7	0,769881189	0,018992504
OIP5-AS1	XYLT2	OIP5-AS1\XYLT2	0,753144681	0,023533951
OIP5-AS1	ZBTB41	OIP5-AS1\ZBTB41	0,769881189	0,018992504
OIP5-AS1	ZMYM6	OIP5-AS1\ZMYM6	0,761512935	0,021263227
OIP5-AS1	ZNF132	OIP5-AS1\ZNF132	0,711303294	0,037125219
OIP5-AS1	ZNF280B	OIP5-AS1\ZNF280B	0,694566727	0,043816138
OIP5-AS1	ZNF449	OIP5-AS1\ZNF449	0,714285731	0,035008818
OIP5-AS1	ZNF543	OIP5-AS1\ZNF543	0,744776368	0,026047178

OIP5-AS1	ZNF544	OIP5-AS1\ZNF544	0,719671547	0,033763226
OIP5-AS1	ZNF558	OIP5-AS1\ZNF558	0,70293504	0,040145501
OIP5-AS1	ZNF562	OIP5-AS1\ZNF562	0,761512935	0,021263227
OIP5-AS1	ZNF782	OIP5-AS1\ZNF782	0,714285731	0,035008818
OIP5-AS1	ZRANB3	OIP5-AS1\ZRANB3	0,70293504	0,040145501
PARD6G-AS1	ACAD10	PARD6G-AS1\ACAD10	0,93724668	0,000485009
PARD6G-AS1	AFG3L2	PARD6G-AS1\AFG3L2	0,699999928	0,043253969
PARD6G-AS1	AK3	PARD6G-AS1\AK3	0,799999952	0,013828263
PARD6G-AS1	ANKFY1	PARD6G-AS1\ANKFY1	0,766666651	0,021389991
PARD6G-AS1	AP1S1	PARD6G-AS1\AP1S1	0,716666639	0,036866181
PARD6G-AS1	APOBEC2	PARD6G-AS1\APOBEC2	0,816666603	0,010769401
PARD6G-AS1	APTX	PARD6G-AS1\APTX	0,916666567	0,001311728
PARD6G-AS1	ARL6IP1	PARD6G-AS1\ARL6IP1	0,816666603	0,010769401
PARD6G-AS1	ARV1	PARD6G-AS1\ARV1	0,949999988	0,000352734
PARD6G-AS1	ATP2C1	PARD6G-AS1\ATP2C1	0,73333329	0,031123236
PARD6G-AS1	ATP6V0B	PARD6G-AS1\ATP6V0B	0,933333278	0,000749559
PARD6G-AS1	ATP6V1C1	PARD6G-AS1\ATP6V1C1	0,899999917	0,002028219
PARD6G-AS1	ATPAF1	PARD6G-AS1\ATPAF1	0,849999964	0,006073633
PARD6G-AS1	ATPIF1	PARD6G-AS1\ATPIF1	0,766666651	0,021389991
PARD6G-AS1	BBS4	PARD6G-AS1\BBS4	0,783333302	0,017223325
PARD6G-AS1	BRD1	PARD6G-AS1\BRD1	0,716666639	0,036866181
PARD6G-AS1	C15orf41	PARD6G-AS1\C15orf41	0,766666651	0,021389991
PARD6G-AS1	C19orf44	PARD6G-AS1\C19orf44	0,74999994	0,025490521
PARD6G-AS1	C19orf54	PARD6G-AS1\C19orf54	0,699999928	0,043253969
PARD6G-AS1	C19orf82	PARD6G-AS1\C19orf82	0,766666651	0,021389991
PARD6G-AS1	C1orf109	PARD6G-AS1\C1orf109	0,949999988	0,000352734
PARD6G-AS1	CAMSAP1	PARD6G-AS1\CAMSAP1	0,699999928	0,043253969
PARD6G-AS1	CBLN3	PARD6G-AS1\CBLN3	0,699999928	0,043253969
PARD6G-AS1	CBS	PARD6G-AS1\CBS	0,849999964	0,006073633
PARD6G-AS1	CCDC30	PARD6G-AS1\CCDC30	0,778249502	0,017383156
PARD6G-AS1	CCZ1	PARD6G-AS1\CCZ1	0,73333329	0,031123236
PARD6G-AS1	CETN3	PARD6G-AS1\CETN3	0,699999928	0,043253969
PARD6G-AS1	CLCN5	PARD6G-AS1\CLCN5	0,699999928	0,043253969
PARD6G-AS1	CLDN12	PARD6G-AS1\CLDN12	0,716666639	0,036866181

PARD6G-AS1	CLINT1	PARD6G-AS1\CLINT1	0,866666615	0,004508378
PARD6G-AS1	CLTC	PARD6G-AS1\CLTC	0,699999928	0,043253969
PARD6G-AS1	COG5	PARD6G-AS1\COG5	0,799999952	0,013828263
PARD6G-AS1	COG8	PARD6G-AS1\COG8	0,70293504	0,040145501
PARD6G-AS1	CROT	PARD6G-AS1\CROT	0,783333302	0,017223325
PARD6G-AS1	CSRP2BP	PARD6G-AS1\CSRP2BP	0,833333313	0,008267196
PARD6G-AS1	CUX1	PARD6G-AS1\CUX1	0,899999917	0,002028219
PARD6G-AS1	DHTKD1	PARD6G-AS1\DHTKD1	0,883333266	0,003075397
PARD6G-AS1	DNAI1	PARD6G-AS1\DNAI1	0,699999928	0,043253969
PARD6G-AS1	DNAJC16	PARD6G-AS1\DNAJC16	0,783333302	0,017223325
PARD6G-AS1	DUOX1	PARD6G-AS1\DUOX1	0,786617756	0,015288801
PARD6G-AS1	EIF2AK1	PARD6G-AS1\EIF2AK1	0,73333329	0,031123236
PARD6G-AS1	ELOVL6	PARD6G-AS1\ELOVL6	0,883333266	0,003075397
PARD6G-AS1	ENPEP	PARD6G-AS1\ENPEP	0,73333329	0,031123236
PARD6G-AS1	ERAP1	PARD6G-AS1\ERAP1	0,716666639	0,036866181
PARD6G-AS1	EXOC7	PARD6G-AS1\EXOC7	0,745869875	0,025859788
PARD6G-AS1	FAF2	PARD6G-AS1\FAF2	0,766666651	0,021389991
PARD6G-AS1	FAM120A	PARD6G-AS1\FAM120A	0,849999964	0,006073633
PARD6G-AS1	FAM163A	PARD6G-AS1\FAM163A	0,716666639	0,036866181
PARD6G-AS1	FAM199X	PARD6G-AS1\FAM199X	0,73333329	0,031123236
PARD6G-AS1	FAM45A	PARD6G-AS1\FAM45A	0,933333278	0,000749559
PARD6G-AS1	FBXO22	PARD6G-AS1\FBXO22	0,728039861	0,031283069
PARD6G-AS1	FBXW11	PARD6G-AS1\FBXW11	0,745869875	0,025859788
PARD6G-AS1	FKTN	PARD6G-AS1\FKTN	0,866666615	0,004508378
PARD6G-AS1	FLVCR1	PARD6G-AS1\FLVCR1	0,916666567	0,001311728
PARD6G-AS1	FSTL4	PARD6G-AS1\FSTL4	0,74999994	0,025490521
PARD6G-AS1	GGA1	PARD6G-AS1\GGA1	0,699999928	0,043253969
PARD6G-AS1	GGA2	PARD6G-AS1\GGA2	0,783333302	0,017223325
PARD6G-AS1	GGCT	PARD6G-AS1\GGCT	0,866666615	0,004508378
PARD6G-AS1	GINS3	PARD6G-AS1\GINS3	0,866666615	0,004508378
PARD6G-AS1	GLI2	PARD6G-AS1\GLI2	0,699999928	0,043253969
PARD6G-AS1	GNPDA2	PARD6G-AS1\GNPDA2	0,716666639	0,036866181
PARD6G-AS1	GPS1	PARD6G-AS1\GPS1	0,833333313	0,008267196
PARD6G-AS1	GTF2H3	PARD6G-AS1\GTF2H3	0,833333313	0,008267196

PARD6G-AS1	GTF2H4	PARD6G-AS1\GTF2H4	0,796724617	0,014087302
PARD6G-AS1	GTF2I	PARD6G-AS1\GTF2I	0,833333313	0,008267196
PARD6G-AS1	GTF3C4	PARD6G-AS1\GTF3C4	0,699999928	0,043253969
PARD6G-AS1	HDAC4	PARD6G-AS1\HDAC4	0,728039861	0,031283069
PARD6G-AS1	HIST1H2BC	PARD6G-AS1\HIST1H2BC	0,716666639	0,036866181
PARD6G-AS1	HIST2H2BF	PARD6G-AS1\HIST2H2BF	0,783333302	0,017223325
PARD6G-AS1	HN1L	PARD6G-AS1\HN1L	0,766666651	0,021389991
PARD6G-AS1	HOMER2	PARD6G-AS1\HOMER2	0,833333313	0,008267196
PARD6G-AS1	HPSE	PARD6G-AS1\HPSE	0,816666603	0,010769401
PARD6G-AS1	IDH3G	PARD6G-AS1\IDH3G	0,899999917	0,002028219
PARD6G-AS1	KDM4A	PARD6G-AS1\KDM4A	0,74999994	0,025490521
PARD6G-AS1	KDM4D	PARD6G-AS1\KDM4D	0,769881189	0,018992504
PARD6G-AS1	KIAA1549	PARD6G-AS1\KIAA1549	0,899999917	0,002028219
PARD6G-AS1	KIF3A	PARD6G-AS1\KIF3A	0,816666603	0,010769401
PARD6G-AS1	KLHDC10	PARD6G-AS1\KLHDC10	0,816666603	0,010769401
PARD6G-AS1	LARS	PARD6G-AS1\LARS	0,783333302	0,017223325
PARD6G-AS1	LIMK1	PARD6G-AS1\LIMK1	0,811722577	0,010769401
PARD6G-AS1	LOC285556	PARD6G-AS1\LOC285556	0,74999994	0,025490521
PARD6G-AS1	LRPPRC	PARD6G-AS1\LRPPRC	0,866666615	0,004508378
PARD6G-AS1	LRRC27	PARD6G-AS1\LRRC27	0,716666639	0,036866181
PARD6G-AS1	LZIC	PARD6G-AS1\LZIC	0,866666615	0,004508378
PARD6G-AS1	MAML3	PARD6G-AS1\MAML3	0,816666603	0,010769401
PARD6G-AS1	MAP10	PARD6G-AS1\MAP10	0,699999928	0,043253969
PARD6G-AS1	MAP3K2	PARD6G-AS1\MAP3K2	0,866666615	0,004508378
PARD6G-AS1	MCOLN3	PARD6G-AS1\MCOLN3	0,866666615	0,004508378
PARD6G-AS1	METTL10	PARD6G-AS1\METTL10	0,73333329	0,031123236
PARD6G-AS1	MPP5	PARD6G-AS1\MPP5	0,74999994	0,025490521
PARD6G-AS1	MTPN	PARD6G-AS1\MTPN	0,711303294	0,037125219
PARD6G-AS1	MTSS1L	PARD6G-AS1\MTSS1L	0,883333266	0,003075397
PARD6G-AS1	N4BP1	PARD6G-AS1\N4BP1	0,699999928	0,043253969
PARD6G-AS1	NAA50	PARD6G-AS1\NAA50	0,716666639	0,036866181
PARD6G-AS1	NAGK	PARD6G-AS1\NAGK	0,716666639	0,036866181
PARD6G-AS1	NDUFA4	PARD6G-AS1\NDUFA4	0,699999928	0,043253969
PARD6G-AS1	NDUFB5	PARD6G-AS1\NDUFB5	0,849999964	0,006073633

PARD6G-AS1	NETO2	PARD6G-AS1\NETO2	0,716666639	0,036866181
PARD6G-AS1	NIF3L1	PARD6G-AS1\NIF3L1	0,73333329	0,031123236
PARD6G-AS1	NIPSNAP1	PARD6G-AS1\NIPSNAP1	0,899999917	0,002028219
PARD6G-AS1	NPTX1	PARD6G-AS1\NPTX1	0,716666639	0,036866181
PARD6G-AS1	NRCAM	PARD6G-AS1\NRCAM	0,74999994	0,025490521
PARD6G-AS1	NUDT12	PARD6G-AS1\NUDT12	0,786617756	0,015288801
PARD6G-AS1	NUDT5	PARD6G-AS1\NUDT5	0,766666651	0,021389991
PARD6G-AS1	NVL	PARD6G-AS1\NVL	0,833333313	0,008267196
PARD6G-AS1	OSBPL3	PARD6G-AS1\OSBPL3	0,74999994	0,025490521
PARD6G-AS1	OXCT1	PARD6G-AS1\OXCT1	0,716666639	0,036866181
PARD6G-AS1	PANK1	PARD6G-AS1\PANK1	0,883333266	0,003075397
PARD6G-AS1	PASK	PARD6G-AS1\PASK	0,866666615	0,004508378
PARD6G-AS1	PCBD2	PARD6G-AS1\PCBD2	0,866666615	0,004508378
PARD6G-AS1	PCCB	PARD6G-AS1\PCCB	0,766666651	0,021389991
PARD6G-AS1	PFN4	PARD6G-AS1\PFN4	0,816666603	0,010769401
PARD6G-AS1	PLA2G12A	PARD6G-AS1\PLA2G12A	0,74999994	0,025490521
PARD6G-AS1	PLEK2	PARD6G-AS1\PLEK2	0,74999994	0,025490521
PARD6G-AS1	PMPCB	PARD6G-AS1\PMPCB	0,799999952	0,013828263
PARD6G-AS1	PNPT1	PARD6G-AS1\PNPT1	0,799999952	0,013828263
PARD6G-AS1	POLR1A	PARD6G-AS1\POLR1A	0,73333329	0,031123236
PARD6G-AS1	PPP5C	PARD6G-AS1\PPP5C	0,816666603	0,010769401
PARD6G-AS1	PRRC1	PARD6G-AS1\PRRC1	0,766666651	0,021389991
PARD6G-AS1	PRSS8	PARD6G-AS1\PRSS8	0,783333302	0,017223325
PARD6G-AS1	PSMD12	PARD6G-AS1\PSMD12	0,783333302	0,017223325
PARD6G-AS1	PSMD5	PARD6G-AS1\PSMD5	0,686198473	0,047167107
PARD6G-AS1	PTPRK	PARD6G-AS1\PTPRK	0,899999917	0,002028219
PARD6G-AS1	RAB40C	PARD6G-AS1\RAB40C	0,833333313	0,008267196
PARD6G-AS1	RAD50	PARD6G-AS1\RAD50	0,866666615	0,004508378
PARD6G-AS1	RBM23	PARD6G-AS1\RBM23	0,949999988	0,000352734
PARD6G-AS1	RCC2	PARD6G-AS1\RCC2	0,833333313	0,008267196
PARD6G-AS1	RFESD	PARD6G-AS1\RFESD	0,883333266	0,003075397
PARD6G-AS1	RGS21	PARD6G-AS1\RGS21	0,796724617	0,013227513
PARD6G-AS1	RMDN3	PARD6G-AS1\RMDN3	0,694566727	0,043816138
PARD6G-AS1	RNF212	PARD6G-AS1\RNF212	0,699999928	0,043253969

PARD6G-AS1	RP9	PARD6G-AS1\RP9	0,73333329	0,031123236
PARD6G-AS1	RPGRIP1L	PARD6G-AS1\RPGRIP1L	0,716666639	0,036866181
PARD6G-AS1	RRAGB	PARD6G-AS1\RRAGB	0,866666615	0,004508378
PARD6G-AS1	RTF1	PARD6G-AS1\RTF1	0,916666567	0,001311728
PARD6G-AS1	SARS2	PARD6G-AS1\SARS2	0,719671547	0,033763226
PARD6G-AS1	SCCPDH	PARD6G-AS1\SCCPDH	0,833333313	0,008267196
PARD6G-AS1	SCD5	PARD6G-AS1\SCD5	0,73333329	0,031123236
PARD6G-AS1	SFXN5	PARD6G-AS1\SFXN5	0,778249502	0,017383156
PARD6G-AS1	SLC30A6	PARD6G-AS1\SLC30A6	0,883333266	0,003075397
PARD6G-AS1	SLC44A3	PARD6G-AS1\SLC44A3	0,883333266	0,003075397
PARD6G-AS1	SLC6A8	PARD6G-AS1\SLC6A8	0,783333302	0,017223325
PARD6G-AS1	SNX1	PARD6G-AS1\SNX1	0,766666651	0,021389991
PARD6G-AS1	SORCS1	PARD6G-AS1\SORCS1	0,73333329	0,031123236
PARD6G-AS1	SPATA2	PARD6G-AS1\SPATA2	0,799999952	0,013828263
PARD6G-AS1	SPIRE2	PARD6G-AS1\SPIRE2	0,828459144	0,008289241
PARD6G-AS1	SPRY3	PARD6G-AS1\SPRY3	0,866666615	0,004508378
PARD6G-AS1	STX6	PARD6G-AS1\STX6	0,799999952	0,013828263
PARD6G-AS1	TCEB3	PARD6G-AS1\TCEB3	0,816666603	0,010769401
PARD6G-AS1	THSD7B	PARD6G-AS1\THSD7B	0,73333329	0,031123236
PARD6G-AS1	TMEM107	PARD6G-AS1\TMEM107	0,699999928	0,043253969
PARD6G-AS1	TRIM14	PARD6G-AS1\TRIM14	0,73333329	0,031123236
PARD6G-AS1	TRIM52	PARD6G-AS1\TRIM52	0,866666615	0,004508378
PARD6G-AS1	TRIM61	PARD6G-AS1\TRIM61	0,833333313	0,008267196
PARD6G-AS1	TRIM7	PARD6G-AS1\TRIM7	0,74999994	0,025490521
PARD6G-AS1	TRMT10B	PARD6G-AS1\TRMT10B	0,866666615	0,004508378
PARD6G-AS1	TRUB1	PARD6G-AS1\TRUB1	0,816666603	0,010769401
PARD6G-AS1	TTC22	PARD6G-AS1\TTC22	0,699999928	0,043253969
PARD6G-AS1	TTC26	PARD6G-AS1\TTC26	0,74999994	0,025490521
PARD6G-AS1	TYW5	PARD6G-AS1\TYW5	0,73333329	0,031123236
PARD6G-AS1	UBAP2	PARD6G-AS1\UBAP2	0,98333329	4,96032E-05
PARD6G-AS1	UBE2V2	PARD6G-AS1\UBE2V2	0,845195651	0,006404321
PARD6G-AS1	UBE3C	PARD6G-AS1\UBE3C	0,816666603	0,010769401
PARD6G-AS1	UBFD1	PARD6G-AS1\UBFD1	0,916666567	0,001311728
PARD6G-AS1	VAC14	PARD6G-AS1\VAC14	0,883333266	0,003075397

PARD6G-AS1	VANGL1	PARD6G-AS1\VANGL1	0,816666603	0,010769401
PARD6G-AS1	VMA21	PARD6G-AS1\VMA21	0,766666651	0,021389991
PARD6G-AS1	WDR3	PARD6G-AS1\WDR3	0,783333302	0,017223325
PARD6G-AS1	WDR61	PARD6G-AS1\WDR61	0,728039861	0,031283069
PARD6G-AS1	WNK2	PARD6G-AS1\WNK2	0,73333329	0,031123236
PARD6G-AS1	WWOX	PARD6G-AS1\WWOX	0,91214186	0,001322751
PARD6G-AS1	XPO7	PARD6G-AS1\XPO7	0,849999964	0,006073633
PARD6G-AS1	XYLT2	PARD6G-AS1\XYLT2	0,816666603	0,010769401
PARD6G-AS1	YY1AP1	PARD6G-AS1\YY1AP1	0,833333313	0,008267196
PARD6G-AS1	ZBTB41	PARD6G-AS1\ZBTB41	0,716666639	0,036866181
PARD6G-AS1	ZC3HC1	PARD6G-AS1\ZC3HC1	0,74999994	0,025490521
PARD6G-AS1	ZMYM3	PARD6G-AS1\ZMYM3	0,833333313	0,008267196
PARD6G-AS1	ZMYM6	PARD6G-AS1\ZMYM6	0,916666567	0,001311728
PARD6G-AS1	ZNF132	PARD6G-AS1\ZNF132	0,783333302	0,017223325
PARD6G-AS1	ZNF449	PARD6G-AS1\ZNF449	0,753144681	0,023533951
PARD6G-AS1	ZNF543	PARD6G-AS1\ZNF543	0,933333278	0,000749559
PARD6G-AS1	ZNF544	PARD6G-AS1\ZNF544	0,783333302	0,017223325
PARD6G-AS1	ZNF554	PARD6G-AS1\ZNF554	0,864530981	0,004464286
PARD6G-AS1	ZNF558	PARD6G-AS1\ZNF558	0,73333329	0,031123236
PARD6G-AS1	ZNF562	PARD6G-AS1\ZNF562	0,799999952	0,013828263
PARD6G-AS1	ZNF605	PARD6G-AS1\ZNF605	0,74999994	0,025490521
PARD6G-AS1	ZNF782	PARD6G-AS1\ZNF782	0,686198473	0,046968695
PARD6G-AS1	ZRANB3	PARD6G-AS1\ZRANB3	0,849999964	0,006073633
PARD6G-AS1	ZSCAN31	PARD6G-AS1\ZSCAN31	0,716666639	0,036866181
PTOV1-AS1	AJUBA	PTOV1-AS1\AJUBA	0,966666639	0,000165344
PTOV1-AS1	ALDH7A1	PTOV1-AS1\ALDH7A1	0,866666615	0,004508378
PTOV1-AS1	ALS2	PTOV1-AS1\ALS2	0,899999917	0,002028219
PTOV1-AS1	ANK3	PTOV1-AS1\ANK3	0,899999917	0,002028219
PTOV1-AS1	AP1S1	PTOV1-AS1\AP1S1	0,833333313	0,008267196
PTOV1-AS1	APOBEC2	PTOV1-AS1\APOBEC2	0,799999952	0,013828263
PTOV1-AS1	APP	PTOV1-AS1\APP	0,74999994	0,025490521
PTOV1-AS1	ATP6V0A1	PTOV1-AS1\ATP6V0A1	0,830627799	0,008300264
PTOV1-AS1	ATP6V0D1	PTOV1-AS1\ATP6V0D1	0,816666603	0,010769401
PTOV1-AS1	AUTS2	PTOV1-AS1\AUTS2	0,949999988	0,000352734

PTOV1-AS1	AZIN1	PTOV1-AS1\AZIN1	0,799999952	0,013828263
PTOV1-AS1	BAIAP2	PTOV1-AS1\BAIAP2	0,849999964	0,006073633
PTOV1-AS1	BRD1	PTOV1-AS1\BRD1	0,916666567	0,001311728
PTOV1-AS1	C14orf39	PTOV1-AS1\C14orf39	0,833333313	0,008267196
PTOV1-AS1	C15orf41	PTOV1-AS1\C15orf41	0,783333302	0,017223325
PTOV1-AS1	C19orf44	PTOV1-AS1\C19orf44	0,783333302	0,017223325
PTOV1-AS1	C19orf82	PTOV1-AS1\C19orf82	0,849999964	0,006073633
PTOV1-AS1	C1orf109	PTOV1-AS1\C1orf109	0,699999928	0,043253969
PTOV1-AS1	C4A	PTOV1-AS1\C4A	0,916666567	0,001311728
PTOV1-AS1	C4B	PTOV1-AS1\C4B	0,916666567	0,001311728
PTOV1-AS1	CAMSAP1	PTOV1-AS1\CAMSAP1	0,716666639	0,036866181
PTOV1-AS1	CBS	PTOV1-AS1\CBS	0,799999952	0,013828263
PTOV1-AS1	CCDC122	PTOV1-AS1\CCDC122	0,816666603	0,010769401
PTOV1-AS1	CCDC148	PTOV1-AS1\CCDC148	0,799999952	0,013828263
PTOV1-AS1	CCDC30	PTOV1-AS1\CCDC30	0,916666567	0,001311728
PTOV1-AS1	CCDC73	PTOV1-AS1\CCDC73	0,828459144	0,008289241
PTOV1-AS1	CCDC8	PTOV1-AS1\CCDC8	0,966666639	0,000165344
PTOV1-AS1	CDC42BPG	PTOV1-AS1\CDC42BPG	0,920510173	0,000981041
PTOV1-AS1	CDH1	PTOV1-AS1\CDH1	0,766666651	0,021389991
PTOV1-AS1	CDIP1	PTOV1-AS1\CDIP1	0,73333329	0,031123236
PTOV1-AS1	CDKL3	PTOV1-AS1\CDKL3	0,916666567	0,001311728
PTOV1-AS1	CDS1	PTOV1-AS1\CDS1	0,716666639	0,036866181
PTOV1-AS1	CETN3	PTOV1-AS1\CETN3	0,73333329	0,031123236
PTOV1-AS1	CHID1	PTOV1-AS1\CHID1	0,833333313	0,008267196
PTOV1-AS1	CLCA1	PTOV1-AS1\CLCA1	0,818164945	0,008333334
PTOV1-AS1	CLCN5	PTOV1-AS1\CLCN5	0,849999964	0,006073633
PTOV1-AS1	CLDN12	PTOV1-AS1\CLDN12	0,799999952	0,013828263
PTOV1-AS1	CLHC1	PTOV1-AS1\CLHC1	0,74999994	0,025490521
PTOV1-AS1	CLSTN1	PTOV1-AS1\CLSTN1	0,899999917	0,002028219
PTOV1-AS1	CNTNAP3B	PTOV1-AS1\CNTNAP3B	0,73333329	0,031123236
PTOV1-AS1	COL8A2	PTOV1-AS1\COL8A2	0,783333302	0,017223325
PTOV1-AS1	COX19	PTOV1-AS1\COX19	0,833333313	0,008267196
PTOV1-AS1	CROT	PTOV1-AS1\CROT	0,74999994	0,025490521
PTOV1-AS1	CRTC1	PTOV1-AS1\CRTC1	0,849999964	0,006073633

PTOV1-AS1	DCAKD	PTOV1-AS1\DCAKD	0,883333266	0,003075397
PTOV1-AS1	DDX31	PTOV1-AS1\DDX31	0,866666615	0,004508378
PTOV1-AS1	DET1	PTOV1-AS1\DET1	0,899999917	0,002028219
PTOV1-AS1	DHTKD1	PTOV1-AS1\DHTKD1	0,73333329	0,031123236
PTOV1-AS1	DHX32	PTOV1-AS1\DHX32	0,833333313	0,008267196
PTOV1-AS1	DMRT2	PTOV1-AS1\DMRT2	0,73333329	0,031123236
PTOV1-AS1	DNAJC16	PTOV1-AS1\DNAJC16	0,849999964	0,006073633
PTOV1-AS1	DNAJC19	PTOV1-AS1\DNAJC19	0,783333302	0,017223325
PTOV1-AS1	DNAJC21	PTOV1-AS1\DNAJC21	0,783333302	0,017223325
PTOV1-AS1	DNAL4	PTOV1-AS1\DNAL4	0,833333313	0,008267196
PTOV1-AS1	DPH6	PTOV1-AS1\DPH6	0,833333313	0,008267196
PTOV1-AS1	DUOX1	PTOV1-AS1\DUOX1	0,753144681	0,023533951
PTOV1-AS1	DUOXA1	PTOV1-AS1\DUOXA1	0,883333266	0,003075397
PTOV1-AS1	EDA2R	PTOV1-AS1\EDA2R	0,883333266	0,003075397
PTOV1-AS1	EFNB2	PTOV1-AS1\EFNB2	0,766666651	0,021389991
PTOV1-AS1	EMC1	PTOV1-AS1\EMC1	0,766666651	0,021389991
PTOV1-AS1	EMC10	PTOV1-AS1\EMC10	0,853563964	0,005202822
PTOV1-AS1	ENOSF1	PTOV1-AS1\ENOSF1	0,74999994	0,025490521
PTOV1-AS1	EPN3	PTOV1-AS1\EPN3	0,933333278	0,000749559
PTOV1-AS1	EPS15L1	PTOV1-AS1\EPS15L1	0,883333266	0,003075397
PTOV1-AS1	ESRP1	PTOV1-AS1\ESRP1	0,833333313	0,008267196
PTOV1-AS1	ESRP2	PTOV1-AS1\ESRP2	0,799999952	0,013828263
PTOV1-AS1	EVA1A	PTOV1-AS1\EVA1A	0,74999994	0,025490521
PTOV1-AS1	EXOC7	PTOV1-AS1\EXOC7	0,813676238	0,010449735
PTOV1-AS1	EYA2	PTOV1-AS1\EYA2	0,73333329	0,031123236
PTOV1-AS1	FAHD1	PTOV1-AS1\FAHD1	0,783333302	0,017223325
PTOV1-AS1	FAM154B	PTOV1-AS1\FAM154B	0,779773057	0,016765874
PTOV1-AS1	FAM160A1	PTOV1-AS1\FAM160A1	0,916666567	0,001311728
PTOV1-AS1	FAM218A	PTOV1-AS1\FAM218A	0,883333266	0,003075397
PTOV1-AS1	FAM83B	PTOV1-AS1\FAM83B	0,783333302	0,017223325
PTOV1-AS1	FBXL16	PTOV1-AS1\FBXL16	0,783333302	0,017223325
PTOV1-AS1	FBXW11	PTOV1-AS1\FBXW11	0,813676238	0,010449735
PTOV1-AS1	FEM1B	PTOV1-AS1\FEM1B	0,949999988	0,000352734
PTOV1-AS1	FNBP1L	PTOV1-AS1\FNBP1L	0,783333302	0,017223325

PTOV1-AS1	FOXE1	PTOV1-AS1\FOXE1	0,849999964	0,006073633
PTOV1-AS1	GGA1	PTOV1-AS1\GGA1	0,866666615	0,004508378
PTOV1-AS1	GGA2	PTOV1-AS1\GGA2	0,73333329	0,031123236
PTOV1-AS1	GGCT	PTOV1-AS1\GGCT	0,783333302	0,017223325
PTOV1-AS1	GLI2	PTOV1-AS1\GLI2	0,716666639	0,036866181
PTOV1-AS1	GNPDA1	PTOV1-AS1\GNPDA1	0,833333313	0,008267196
PTOV1-AS1	GOLM1	PTOV1-AS1\GOLM1	0,699999928	0,043253969
PTOV1-AS1	GPC4	PTOV1-AS1\GPC4	0,783333302	0,017223325
PTOV1-AS1	GPR107	PTOV1-AS1\GPR107	0,728918254	0,031349208
PTOV1-AS1	GRHL2	PTOV1-AS1\GRHL2	0,833333313	0,008267196
PTOV1-AS1	GRIP1	PTOV1-AS1\GRIP1	0,74999994	0,025490521
PTOV1-AS1	GRTP1	PTOV1-AS1\GRTP1	0,883333266	0,003075397
PTOV1-AS1	GTF3C4	PTOV1-AS1\GTF3C4	0,849999964	0,006073633
PTOV1-AS1	HIST2H2BF	PTOV1-AS1\HIST2H2BF	0,766666651	0,021389991
PTOV1-AS1	HN1L	PTOV1-AS1\HN1L	0,883333266	0,003075397
PTOV1-AS1	HOMER3	PTOV1-AS1\HOMER3	0,933333278	0,000749559
PTOV1-AS1	HPS5	PTOV1-AS1\HPS5	0,916666567	0,001311728
PTOV1-AS1	HPSE	PTOV1-AS1\HPSE	0,816666603	0,010769401
PTOV1-AS1	IFT140	PTOV1-AS1\IFT140	0,816666603	0,010769401
PTOV1-AS1	IGF1R	PTOV1-AS1\IGF1R	0,783333302	0,017223325
PTOV1-AS1	IGFBP2	PTOV1-AS1\IGFBP2	0,766666651	0,021389991
PTOV1-AS1	IGSF3	PTOV1-AS1\IGSF3	0,74999994	0,025490521
PTOV1-AS1	IKBIP	PTOV1-AS1\IKBIP	0,716666639	0,036866181
PTOV1-AS1	IQCE	PTOV1-AS1\IQCE	0,799999952	0,013828263
PTOV1-AS1	IRF2BP2	PTOV1-AS1\IRF2BP2	0,699999928	0,043253969
PTOV1-AS1	ITGA2	PTOV1-AS1\ITGA2	0,883333266	0,003075397
PTOV1-AS1	KAL1	PTOV1-AS1\KAL1	0,799999952	0,013828263
PTOV1-AS1	KCTD1	PTOV1-AS1\KCTD1	0,766666651	0,021389991
PTOV1-AS1	KDM4D	PTOV1-AS1\KDM4D	0,769881189	0,018992504
PTOV1-AS1	KIAA0319L	PTOV1-AS1\KIAA0319L	0,74999994	0,025490521
PTOV1-AS1	KLHDC10	PTOV1-AS1\KLHDC10	0,699999928	0,043253969
PTOV1-AS1	KRTAP5-8	PTOV1-AS1\KRTAP5-8	0,98333329	4,96032E-05
PTOV1-AS1	LAMP1	PTOV1-AS1\LAMP1	0,845195651	0,006205908
PTOV1-AS1	LAMP5	PTOV1-AS1\LAMP5	0,833333313	0,008267196

PTOV1-AS1	LEPREL1	PTOV1-AS1\LEPREL1	0,866666615	0,004508378
PTOV1-AS1	LIMK1	PTOV1-AS1\LIMK1	0,711303294	0,037125219
PTOV1-AS1	LPHN3	PTOV1-AS1\LPHN3	0,799999952	0,013828263
PTOV1-AS1	LPIN1	PTOV1-AS1\LPIN1	0,716666639	0,036866181
PTOV1-AS1	LRIG3	PTOV1-AS1\LRIG3	0,933333278	0,000749559
PTOV1-AS1	LRPAP1	PTOV1-AS1\LRPAP1	0,811722577	0,010769401
PTOV1-AS1	LRPPRC	PTOV1-AS1\LRPPRC	0,783333302	0,017223325
PTOV1-AS1	LRRC27	PTOV1-AS1\LRRC27	0,73333329	0,031123236
PTOV1-AS1	LRRK1	PTOV1-AS1\LRRK1	0,816666603	0,010769401
PTOV1-AS1	M1AP	PTOV1-AS1\M1AP	0,766666651	0,021389991
PTOV1-AS1	MAML3	PTOV1-AS1\MAML3	0,799999952	0,013828263
PTOV1-AS1	MARVELD2	PTOV1-AS1\MARVELD2	0,799999952	0,013828263
PTOV1-AS1	MAST4	PTOV1-AS1\MAST4	0,778249502	0,017383156
PTOV1-AS1	MBLAC2	PTOV1-AS1\MBLAC2	0,783333302	0,017223325
PTOV1-AS1	MCOLN3	PTOV1-AS1\MCOLN3	0,716666639	0,036866181
PTOV1-AS1	MCTP2	PTOV1-AS1\MCTP2	0,899999917	0,002028219
PTOV1-AS1	MED22	PTOV1-AS1\MED22	0,762821436	0,022123016
PTOV1-AS1	MFSD3	PTOV1-AS1\MFSD3	0,73333329	0,031123236
PTOV1-AS1	MOV10	PTOV1-AS1\MOV10	0,716666639	0,036866181
PTOV1-AS1	MYLK4	PTOV1-AS1\MYLK4	0,783333302	0,017223325
PTOV1-AS1	MYO10	PTOV1-AS1\MYO10	0,849999964	0,006073633
PTOV1-AS1	MYO5B	PTOV1-AS1\MYO5B	0,82009083	0,009281305
PTOV1-AS1	N4BP1	PTOV1-AS1\N4BP1	0,866666615	0,004508378
PTOV1-AS1	N4BP2L2	PTOV1-AS1\N4BP2L2	0,699999928	0,043253969
PTOV1-AS1	NAA25	PTOV1-AS1\NAA25	0,783333302	0,017223325
PTOV1-AS1	NAA50	PTOV1-AS1\NAA50	0,799999952	0,013828263
PTOV1-AS1	NAGK	PTOV1-AS1\NAGK	0,833333313	0,008267196
PTOV1-AS1	NCS1	PTOV1-AS1\NCS1	0,73333329	0,031123236
PTOV1-AS1	NDRG3	PTOV1-AS1\NDRG3	0,849999964	0,006073633
PTOV1-AS1	NEO1	PTOV1-AS1\NEO1	0,916666567	0,001311728
PTOV1-AS1	NET1	PTOV1-AS1\NET1	0,799999952	0,013828263
PTOV1-AS1	NETO2	PTOV1-AS1\NETO2	0,699999928	0,043253969
PTOV1-AS1	NHLRC3	PTOV1-AS1\NHLRC3	0,966666639	0,000165344
PTOV1-AS1	NPNT	PTOV1-AS1\NPNT	0,949999988	0,000352734

PTOV1-AS1	NRCAM	PTOV1-AS1\NRCAM	0,833333313	0,008267196
PTOV1-AS1	NTF4	PTOV1-AS1\NTF4	0,799999952	0,013828263
PTOV1-AS1	NUDT12	PTOV1-AS1\NUDT12	0,769881189	0,018992504
PTOV1-AS1	NUDT9	PTOV1-AS1\NUDT9	0,716666639	0,036866181
PTOV1-AS1	NXN	PTOV1-AS1\NXN	0,828459144	0,008289241
PTOV1-AS1	OSBPL3	PTOV1-AS1\OSBPL3	0,833333313	0,008267196
PTOV1-AS1	PABPC4L	PTOV1-AS1\PABPC4L	0,799999952	0,013828263
PTOV1-AS1	PANK1	PTOV1-AS1\PANK1	0,699999928	0,043253969
PTOV1-AS1	PARD6B	PTOV1-AS1\PARD6B	0,783333302	0,017223325
PTOV1-AS1	PAX1	PTOV1-AS1\PAX1	0,849999964	0,006073633
PTOV1-AS1	PAX9	PTOV1-AS1\PAX9	0,899999917	0,002028219
PTOV1-AS1	PCSK6	PTOV1-AS1\PCSK6	0,73333329	0,031123236
PTOV1-AS1	PDGFA	PTOV1-AS1\PDGFA	0,883333266	0,003075397
PTOV1-AS1	PGAP1	PTOV1-AS1\PGAP1	0,833333313	0,008267196
PTOV1-AS1	PIAS2	PTOV1-AS1\PIAS2	0,816666603	0,010769401
PTOV1-AS1	PIFO	PTOV1-AS1\PIFO	0,833333313	0,008267196
PTOV1-AS1	PKP1	PTOV1-AS1\PKP1	0,74999994	0,025490521
PTOV1-AS1	PKP4	PTOV1-AS1\PKP4	0,816666603	0,010769401
PTOV1-AS1	PLA2G12A	PTOV1-AS1\PLA2G12A	0,883333266	0,003075397
PTOV1-AS1	PLD2	PTOV1-AS1\PLD2	0,73333329	0,031123236
PTOV1-AS1	PMPCB	PTOV1-AS1\PMPCB	0,816666603	0,010769401
PTOV1-AS1	POGZ	PTOV1-AS1\POGZ	0,783333302	0,017223325
PTOV1-AS1	POMT2	PTOV1-AS1\POMT2	0,933333278	0,000749559
PTOV1-AS1	PRKAA2	PTOV1-AS1\PRKAA2	0,799999952	0,013828263
PTOV1-AS1	PRKAB1	PTOV1-AS1\PRKAB1	0,916666567	0,001311728
PTOV1-AS1	PRKAB2	PTOV1-AS1\PRKAB2	0,73333329	0,031123236
PTOV1-AS1	PRKAR1A	PTOV1-AS1\PRKAR1A	0,881482542	0,003009259
PTOV1-AS1	PROP1	PTOV1-AS1\PROP1	0,699999928	0,043253969
PTOV1-AS1	PRRC1	PTOV1-AS1\PRRC1	0,799999952	0,013828263
PTOV1-AS1	PRRG4	PTOV1-AS1\PRRG4	0,883333266	0,003075397
PTOV1-AS1	PRSS8	PTOV1-AS1\PRSS8	0,73333329	0,031123236
PTOV1-AS1	PSAT1	PTOV1-AS1\PSAT1	0,849999964	0,006073633
PTOV1-AS1	PSD3	PTOV1-AS1\PSD3	0,949999988	0,000352734
PTOV1-AS1	PTPRK	PTOV1-AS1\PTPRK	0,73333329	0,031123236

PTOV1-AS1	RAB14	PTOV1-AS1\RAB14	0,799999952	0,013828263
PTOV1-AS1	RAB23	PTOV1-AS1\RAB23	0,833333313	0,008267196
PTOV1-AS1	RAB40C	PTOV1-AS1\RAB40C	0,833333313	0,008267196
PTOV1-AS1	RABGAP1	PTOV1-AS1\RABGAP1	0,933333278	0,000749559
PTOV1-AS1	RANBP17	PTOV1-AS1\RANBP17	0,899999917	0,002028219
PTOV1-AS1	RGS21	PTOV1-AS1\RGS21	0,728918254	0,031349208
PTOV1-AS1	RGS9	PTOV1-AS1\RGS9	0,716666639	0,036866181
PTOV1-AS1	RHBDD2	PTOV1-AS1\RHBDD2	0,783333302	0,017223325
PTOV1-AS1	RMDN3	PTOV1-AS1\RMDN3	0,895405293	0,002105379
PTOV1-AS1	RNF144B	PTOV1-AS1\RNF144B	0,699999928	0,043253969
PTOV1-AS1	RNF170	PTOV1-AS1\RNF170	0,783333302	0,017223325
PTOV1-AS1	RNF212	PTOV1-AS1\RNF212	0,916666567	0,001311728
PTOV1-AS1	RNF32	PTOV1-AS1\RNF32	0,699999928	0,043253969
PTOV1-AS1	RP9	PTOV1-AS1\RP9	0,716666639	0,036866181
PTOV1-AS1	RPGRIP1L	PTOV1-AS1\RPGRIP1L	0,866666615	0,004508378
PTOV1-AS1	RPS6KA6	PTOV1-AS1\RPS6KA6	0,883333266	0,003075397
PTOV1-AS1	RRAGB	PTOV1-AS1\RRAGB	0,716666639	0,036866181
PTOV1-AS1	RRM2B	PTOV1-AS1\RRM2B	0,766666651	0,021389991
PTOV1-AS1	SARS2	PTOV1-AS1\SARS2	0,761512935	0,021263227
PTOV1-AS1	SCAMP1	PTOV1-AS1\SCAMP1	0,816666603	0,010769401
PTOV1-AS1	SDR42E1	PTOV1-AS1\SDR42E1	0,933333278	0,000749559
PTOV1-AS1	SERPINB5	PTOV1-AS1\SERPINB5	0,74999994	0,025490521
PTOV1-AS1	SGPL1	PTOV1-AS1\SGPL1	0,866666615	0,004508378
PTOV1-AS1	SIX1	PTOV1-AS1\SIX1	0,933333278	0,000749559
PTOV1-AS1	SIX4	PTOV1-AS1\SIX4	0,949999988	0,000352734
PTOV1-AS1	SLC22A23	PTOV1-AS1\SLC22A23	0,716666639	0,036866181
PTOV1-AS1	SLC30A1	PTOV1-AS1\SLC30A1	0,783333302	0,017223325
PTOV1-AS1	SLC46A1	PTOV1-AS1\SLC46A1	0,849999964	0,006073633
PTOV1-AS1	SMO	PTOV1-AS1\SMO	0,883333266	0,003075397
PTOV1-AS1	SOGA2	PTOV1-AS1\SOGA2	0,74999994	0,025490521
PTOV1-AS1	SORBS2	PTOV1-AS1\SORBS2	0,73333329	0,031123236
PTOV1-AS1	SPANXN2	PTOV1-AS1\SPANXN2	0,716666639	0,036866181
PTOV1-AS1	SPATA6	PTOV1-AS1\SPATA6	0,816666603	0,010769401
PTOV1-AS1	SPIN1	PTOV1-AS1\SPIN1	0,899999917	0,002028219

PTOV1-AS1	SPIN4	PTOV1-AS1\SPIN4	0,699999928	0,043253969
PTOV1-AS1	SPINT1	PTOV1-AS1\SPINT1	0,883333266	0,003075397
PTOV1-AS1	SPIRE2	PTOV1-AS1\SPIRE2	0,694566727	0,043816138
PTOV1-AS1	STARD7	PTOV1-AS1\STARD7	0,933333278	0,000749559
PTOV1-AS1	STEAP2	PTOV1-AS1\STEAP2	0,716666639	0,036866181
PTOV1-AS1	STK36	PTOV1-AS1\STK36	0,74999994	0,025490521
PTOV1-AS1	STON2	PTOV1-AS1\STON2	0,783333302	0,017223325
PTOV1-AS1	SUPT3H	PTOV1-AS1\SUPT3H	0,928878427	0,000815697
PTOV1-AS1	SYBU	PTOV1-AS1\SYBU	0,699999928	0,043253969
PTOV1-AS1	SYT1	PTOV1-AS1\SYT1	0,833333313	0,008267196
PTOV1-AS1	TAB3	PTOV1-AS1\TAB3	0,849999964	0,006073633
PTOV1-AS1	TM7SF3	PTOV1-AS1\TM7SF3	0,74999994	0,025490521
PTOV1-AS1	TMEM107	PTOV1-AS1\TMEM107	0,716666639	0,036866181
PTOV1-AS1	TMEM133	PTOV1-AS1\TMEM133	0,866666615	0,004508378
PTOV1-AS1	TMEM180	PTOV1-AS1\TMEM180	0,949288905	0,000363757
PTOV1-AS1	TMEM185A	PTOV1-AS1\TMEM185A	0,883333266	0,003075397
PTOV1-AS1	TMEM30B	PTOV1-AS1\TMEM30B	0,883333266	0,003075397
PTOV1-AS1	TNPO1	PTOV1-AS1\TNPO1	0,766666651	0,021389991
PTOV1-AS1	TOMM20	PTOV1-AS1\TOMM20	0,783333302	0,017223325
PTOV1-AS1	TP63	PTOV1-AS1\TP63	0,74999994	0,025490521
PTOV1-AS1	TRMT10B	PTOV1-AS1\TRMT10B	0,799999952	0,013828263
PTOV1-AS1	TRMT5	PTOV1-AS1\TRMT5	0,866666615	0,004508378
PTOV1-AS1	TRPM7	PTOV1-AS1\TRPM7	0,73333329	0,031123236
PTOV1-AS1	TSPAN13	PTOV1-AS1\TSPAN13	0,799999952	0,013828263
PTOV1-AS1	TTC22	PTOV1-AS1\TTC22	0,816666603	0,010769401
PTOV1-AS1	TTC26	PTOV1-AS1\TTC26	0,799999952	0,013828263
PTOV1-AS1	TYW5	PTOV1-AS1\TYW5	0,73333329	0,031123236
PTOV1-AS1	UBE2V2	PTOV1-AS1\UBE2V2	0,744776368	0,026708554
PTOV1-AS1	UBTD2	PTOV1-AS1\UBTD2	0,761512935	0,021263227
PTOV1-AS1	UEVLD	PTOV1-AS1\UEVLD	0,915385723	0,001322751
PTOV1-AS1	UMPS	PTOV1-AS1\UMPS	0,728918254	0,031679895
PTOV1-AS1	UNC5B	PTOV1-AS1\UNC5B	0,833333313	0,008267196
PTOV1-AS1	UPF1	PTOV1-AS1\UPF1	0,766666651	0,021389991
PTOV1-AS1	USP28	PTOV1-AS1\USP28	0,74999994	0,025490521

PTOV1-AS1	USP46	PTOV1-AS1\USP46	0,816666603	0,010769401
PTOV1-AS1	VMA21	PTOV1-AS1\VMA21	0,73333329	0,031123236
PTOV1-AS1	VPS35	PTOV1-AS1\VPS35	0,73333329	0,031123236
PTOV1-AS1	VWCE	PTOV1-AS1\VWCE	0,799999952	0,013828263
PTOV1-AS1	WASL	PTOV1-AS1\WASL	0,933333278	0,000749559
PTOV1-AS1	WNK2	PTOV1-AS1\WNK2	0,716666639	0,036866181
PTOV1-AS1	XPR1	PTOV1-AS1\XPR1	0,933333278	0,000749559
PTOV1-AS1	ZBTB41	PTOV1-AS1\ZBTB41	0,766666651	0,021389991
PTOV1-AS1	ZNF132	PTOV1-AS1\ZNF132	0,766666651	0,021389991
PTOV1-AS1	ZNF211	PTOV1-AS1\ZNF211	0,849999964	0,006073633
PTOV1-AS1	ZNF221	PTOV1-AS1\ZNF221	0,916666567	0,001311728
PTOV1-AS1	ZNF229	PTOV1-AS1\ZNF229	0,699999928	0,043253969
PTOV1-AS1	ZNF257	PTOV1-AS1\ZNF257	0,816666603	0,010769401
PTOV1-AS1	ZNF280B	PTOV1-AS1\ZNF280B	0,833333313	0,008267196
PTOV1-AS1	ZNF449	PTOV1-AS1\ZNF449	0,82009083	0,009281305
PTOV1-AS1	ZNF540	PTOV1-AS1\ZNF540	0,766666651	0,021389991
PTOV1-AS1	ZNF543	PTOV1-AS1\ZNF543	0,73333329	0,031123236
PTOV1-AS1	ZNF544	PTOV1-AS1\ZNF544	0,766666651	0,021389991
PTOV1-AS1	ZNF562	PTOV1-AS1\ZNF562	0,783333302	0,017223325
PTOV1-AS1	ZNF572	PTOV1-AS1\ZNF572	0,716666639	0,036866181
PTOV1-AS1	ZNF626	PTOV1-AS1\ZNF626	0,74999994	0,025490521
PTOV1-AS1	ZSCAN31	PTOV1-AS1\ZSCAN31	0,866666615	0,004508378
PVT1	AGPAT3	PVT1\AGPAT3	0,816666603	0,010769401
PVT1	AKR1B1	PVT1\AKR1B1	0,716666639	0,036866181
PVT1	ALDH7A1	PVT1\ALDH7A1	0,899999917	0,002028219
PVT1	ALS2	PVT1\ALS2	0,813676238	0,010449735
PVT1	ANK3	PVT1\ANK3	0,866666615	0,004508378
PVT1	AP2B1	PVT1\AP2B1	0,883333266	0,003075397
PVT1	APOBEC2	PVT1\APOBEC2	0,699999928	0,043253969
PVT1	APPBP2	PVT1\APPBP2	0,695015132	0,044576719
PVT1	ARL6IP1	PVT1\ARL6IP1	0,711966693	0,038029101
PVT1	ARV1	PVT1\ARV1	0,728918254	0,031349208
PVT1	ATHL1	PVT1\ATHL1	0,866666615	0,004508378
PVT1	ATP2C1	PVT1\ATP2C1	0,745869875	0,025859788

PVT1	ATP6AP2	PVT1\ATP6AP2	0,74999994	0,025490521
PVT1	ATP6V0D1	PVT1\ATP6V0D1	0,699999928	0,043253969
PVT1	ATP6V1A	PVT1\ATP6V1A	0,762821436	0,021329366
PVT1	ATRNL1	PVT1\ATRNL1	0,783333302	0,017223325
PVT1	AUTS2	PVT1\AUTS2	0,783333302	0,017223325
PVT1	AZIN1	PVT1\AZIN1	0,783333302	0,017223325
PVT1	BAIAP2	PVT1\BAIAP2	0,74999994	0,025490521
PVT1	BBS4	PVT1\BBS4	0,813676238	0,010449735
PVT1	BTBD3	PVT1\BTBD3	0,796724617	0,013227513
PVT1	C14orf132	PVT1\C14orf132	0,731974542	0,029298943
PVT1	C14orf39	PVT1\C14orf39	0,711966693	0,038029101
PVT1	C1orf109	PVT1\C1orf109	0,762821436	0,021329366
PVT1	C4A	PVT1\C4A	0,74999994	0,025490521
PVT1	C4B	PVT1\C4B	0,74999994	0,025490521
PVT1	C5orf15	PVT1\C5orf15	0,833333313	0,008267196
PVT1	C5orf24	PVT1\C5orf24	0,711966693	0,038029101
PVT1	CALML3	PVT1\CALML3	0,74999994	0,025490521
PVT1	CALR	PVT1\CALR	0,766666651	0,021389991
PVT1	CAMSAP1	PVT1\CAMSAP1	0,816666603	0,010769401
PVT1	CBS	PVT1\CBS	0,796724617	0,013227513
PVT1	CCDC148	PVT1\CCDC148	0,74999994	0,025490521
PVT1	CCDC30	PVT1\CCDC30	0,699999928	0,043253969
PVT1	CCDC73	PVT1\CCDC73	0,714951873	0,035218254
PVT1	CCDC8	PVT1\CCDC8	0,816666603	0,010769401
PVT1	CCL25	PVT1\CCL25	0,745869875	0,025859788
PVT1	CCZ1	PVT1\CCZ1	0,779773057	0,016765874
PVT1	CD274	PVT1\CD274	0,745869875	0,025859788
PVT1	CDH1	PVT1\CDH1	0,766666651	0,021389991
PVT1	CDKL3	PVT1\CDKL3	0,711966693	0,038029101
PVT1	CDS1	PVT1\CDS1	0,73333329	0,031123236
PVT1	CETN3	PVT1\CETN3	0,73333329	0,031123236
PVT1	CLDN12	PVT1\CLDN12	0,833333313	0,008267196
PVT1	CLHC1	PVT1\CLHC1	0,728918254	0,031349208
PVT1	CLSTN1	PVT1\CLSTN1	0,766666651	0,021389991

PVT1	CLTC	PVT1\CLTC	0,816666603	0,010769401
PVT1	CNKSR3	PVT1\CNKSR3	0,783333302	0,017223325
PVT1	CNTNAP2	PVT1\CNTNAP2	0,695015132	0,044576719
PVT1	CNTNAP3	PVT1\CNTNAP3	0,849999964	0,006073633
PVT1	CNTNAP3B	PVT1\CNTNAP3B	0,783333302	0,017223325
PVT1	COG5	PVT1\COG5	0,699999928	0,043253969
PVT1	COG8	PVT1\COG8	0,694566727	0,043816138
PVT1	CROT	PVT1\CROT	0,716666639	0,036866181
PVT1	CUX1	PVT1\CUX1	0,762821436	0,021329366
PVT1	CYB5B	PVT1\CYB5B	0,762821436	0,021329366
PVT1	CYP26B1	PVT1\CYP26B1	0,695015132	0,044576719
PVT1	DACT1	PVT1\DACT1	0,866666615	0,004508378
PVT1	DDX31	PVT1\DDX31	0,766666651	0,021389991
PVT1	DENND1A	PVT1\DENND1A	0,766666651	0,021389991
PVT1	DHTKD1	PVT1\DHTKD1	0,728918254	0,031349208
PVT1	DHX32	PVT1\DHX32	0,699999928	0,043253969
PVT1	DMRT2	PVT1\DMRT2	0,783333302	0,017223325
PVT1	DMRTA1	PVT1\DMRTA1	0,73333329	0,031123236
PVT1	DNAI1	PVT1\DNAI1	0,783333302	0,017223325
PVT1	DNAJC16	PVT1\DNAJC16	0,699999928	0,043253969
PVT1	DNAJC19	PVT1\DNAJC19	0,699999928	0,043253969
PVT1	DNAL4	PVT1\DNAL4	0,766666651	0,021389991
PVT1	DPH6	PVT1\DPH6	0,866666615	0,004508378
PVT1	DUOX1	PVT1\DUOX1	0,728039861	0,031283069
PVT1	DUOXA1	PVT1\DUOXA1	0,699999928	0,043253969
PVT1	EDA2R	PVT1\EDA2R	0,73333329	0,031123236
PVT1	EIF2AK4	PVT1\EIF2AK4	0,73333329	0,031123236
PVT1	EIF3B	PVT1\EIF3B	0,711966693	0,038029101
PVT1	ELOVL6	PVT1\ELOVL6	0,745869875	0,025859788
PVT1	EMC1	PVT1\EMC1	0,716666639	0,036866181
PVT1	EMC10	PVT1\EMC10	0,680906534	0,049537037
PVT1	ENPEP	PVT1\ENPEP	0,711966693	0,038029101
PVT1	EPN3	PVT1\EPN3	0,766666651	0,021389991
PVT1	ESRP1	PVT1\ESRP1	0,916666567	0,001311728

PVT1	ESRP2	PVT1\ESRP2	0,799999952	0,013828263
PVT1	EVA1A	PVT1\EVA1A	0,799999952	0,013828263
PVT1	EYA2	PVT1\EYA2	0,933333278	0,000749559
PVT1	FAHD1	PVT1\FAHD1	0,833333313	0,008267196
PVT1	FAM154B	PVT1\FAM154B	0,898434162	0,002050265
PVT1	FAM160A1	PVT1\FAM160A1	0,799999952	0,013828263
PVT1	FAM199X	PVT1\FAM199X	0,762821436	0,021329366
PVT1	FAM218A	PVT1\FAM218A	0,73333329	0,031123236
PVT1	FBXL16	PVT1\FBXL16	0,833333313	0,008267196
PVT1	FBXO2	PVT1\FBXO2	0,79498601	0,013833774
PVT1	FEM1B	PVT1\FEM1B	0,766666651	0,021389991
PVT1	FKTN	PVT1\FKTN	0,762821436	0,021329366
PVT1	FLVCR1	PVT1\FLVCR1	0,745869875	0,025859788
PVT1	FNBP1L	PVT1\FNBP1L	0,73333329	0,031123236
PVT1	FSTL4	PVT1\FSTL4	0,833333313	0,008267196
PVT1	GEMIN5	PVT1\GEMIN5	0,830627799	0,007638889
PVT1	GGA2	PVT1\GGA2	0,73333329	0,031123236
PVT1	GGCT	PVT1\GGCT	0,762821436	0,021329366
PVT1	GINS3	PVT1\GINS3	0,796724617	0,013227513
PVT1	GLI2	PVT1\GLI2	0,883333266	0,003075397
PVT1	GNPDA1	PVT1\GNPDA1	0,73333329	0,031123236
PVT1	GNPDA2	PVT1\GNPDA2	0,779773057	0,016765874
PVT1	GOLM1	PVT1\GOLM1	0,716666639	0,036866181
PVT1	GPC4	PVT1\GPC4	0,699999928	0,043253969
PVT1	GPHN	PVT1\GPHN	0,916666567	0,001311728
PVT1	GPR107	PVT1\GPR107	0,779773057	0,016765874
PVT1	GPS1	PVT1\GPS1	0,728918254	0,031349208
PVT1	GRHL2	PVT1\GRHL2	0,916666567	0,001311728
PVT1	GRIP1	PVT1\GRIP1	0,799999952	0,013828263
PVT1	GRTP1	PVT1\GRTP1	0,799999952	0,013828263
PVT1	GTF2H3	PVT1\GTF2H3	0,728918254	0,031349208
PVT1	GTF2H4	PVT1\GTF2H4	0,695015132	0,044576719
PVT1	GTF2IRD2	PVT1\GTF2IRD2	0,830627799	0,007638889
PVT1	GTF3C4	PVT1\GTF3C4	0,74999994	0,025490521

PVT1	HDAC4	PVT1\HDAC4	0,697929204	0,04153439
PVT1	HDGFRP3	PVT1\HDGFRP3	0,728918254	0,031349208
PVT1	HN1L	PVT1\HN1L	0,699999928	0,043253969
PVT1	HOMER2	PVT1\HOMER2	0,779773057	0,016765874
PVT1	HOMER3	PVT1\HOMER3	0,73333329	0,031123236
PVT1	HPSE	PVT1\HPSE	0,796724617	0,013227513
PVT1	IFI44	PVT1\IFI44	0,74999994	0,025490521
PVT1	IFLTD1	PVT1\IFLTD1	0,728918254	0,031349208
PVT1	IGF1R	PVT1\IGF1R	0,695015132	0,044576719
PVT1	IGFBP2	PVT1\IGFBP2	0,833333313	0,008267196
PVT1	IGSF11	PVT1\IGSF11	0,711966693	0,038029101
PVT1	IGSF3	PVT1\IGSF3	0,813676238	0,010449735
PVT1	IKBIP	PVT1\IKBIP	0,728918254	0,031349208
PVT1	IQCH	PVT1\IQCH	0,949999988	0,000352734
PVT1	IRF2BP2	PVT1\IRF2BP2	0,883333266	0,003075397
PVT1	ITGA2	PVT1\ITGA2	0,799999952	0,013828263
PVT1	KAL1	PVT1\KAL1	0,745869875	0,025859788
PVT1	KDM4B	PVT1\KDM4B	0,866666615	0,004508378
PVT1	KDM4D	PVT1\KDM4D	0,711303294	0,037125219
PVT1	KLHDC10	PVT1\KLHDC10	0,813676238	0,010449735
PVT1	KTN1	PVT1\KTN1	0,881482542	0,003009259
PVT1	LAMP2	PVT1\LAMP2	0,769881189	0,018992504
PVT1	LARS	PVT1\LARS	0,864530981	0,004464286
PVT1	LEPREL1	PVT1\LEPREL1	0,799999952	0,013828263
PVT1	LGI3	PVT1\LGI3	0,813676238	0,010449735
PVT1	LMBR1	PVT1\LMBR1	0,849999964	0,006073633
PVT1	LMOD3	PVT1\LMOD3	0,966240525	0,000165344
PVT1	LOC285556	PVT1\LOC285556	0,711966693	0,038029101
PVT1	LPIN1	PVT1\LPIN1	0,699999928	0,043253969
PVT1	LRIG3	PVT1\LRIG3	0,833333313	0,008267196
PVT1	LRPPRC	PVT1\LRPPRC	0,762821436	0,021329366
PVT1	LRRK1	PVT1\LRRK1	0,745869875	0,025859788
PVT1	LZIC	PVT1\LZIC	0,779773057	0,016765874
PVT1	M1AP	PVT1\M1AP	0,916666567	0,001311728

PVT1	MAGEA3	PVT1\MAGEA3	0,779773057	0,016765874
PVT1	MAP3K2	PVT1\MAP3K2	0,762821436	0,021329366
PVT1	MARVELD2	PVT1\MARVELD2	0,816666603	0,010769401
PVT1	MAST4	PVT1\MAST4	0,82009083	0,009281305
PVT1	MCTP2	PVT1\MCTP2	0,883333266	0,003075397
PVT1	MED21	PVT1\MED21	0,714951873	0,035218254
PVT1	METTL8	PVT1\METTL8	0,799999952	0,013828263
PVT1	MFSD3	PVT1\MFSD3	0,866666615	0,004508378
PVT1	MPP5	PVT1\MPP5	0,716666639	0,036866181
PVT1	MRPL40	PVT1\MRPL40	0,74999994	0,025490521
PVT1	MST1L	PVT1\MST1L	0,695015132	0,044576719
PVT1	MTPAP	PVT1\MTPAP	0,864530981	0,004464286
PVT1	MTPN	PVT1\MTPN	0,680906534	0,049537037
PVT1	MTSS1L	PVT1\MTSS1L	0,728918254	0,031349208
PVT1	MYO10	PVT1\MYO10	0,899999917	0,002028219
PVT1	MYO5B	PVT1\MYO5B	0,680906534	0,049537037
PVT1	N4BP1	PVT1\N4BP1	0,699999928	0,043253969
PVT1	NAGK	PVT1\NAGK	0,716666639	0,036866181
PVT1	NCS1	PVT1\NCS1	0,866666615	0,004508378
PVT1	NDRG3	PVT1\NDRG3	0,745869875	0,025859788
PVT1	NDUFA4	PVT1\NDUFA4	0,699999928	0,043253969
PVT1	NEO1	PVT1\NEO1	0,799999952	0,013828263
PVT1	NET1	PVT1\NET1	0,833333313	0,008267196
PVT1	NETO2	PVT1\NETO2	0,74999994	0,025490521
PVT1	NHLRC3	PVT1\NHLRC3	0,716666639	0,036866181
PVT1	NPNT	PVT1\NPNT	0,73333329	0,031123236
PVT1	NPTX1	PVT1\NPTX1	0,728918254	0,031349208
PVT1	NRCAM	PVT1\NRCAM	0,695015132	0,044576719
PVT1	NTF4	PVT1\NTF4	0,73333329	0,031123236
PVT1	NVL	PVT1\NVL	0,779773057	0,016765874
PVT1	NXN	PVT1\NXN	0,711303294	0,037125219
PVT1	OPA1	PVT1\OPA1	0,728918254	0,031349208
PVT1	OXCT1	PVT1\OXCT1	0,73333329	0,031123236
PVT1	PABPC4L	PVT1\PABPC4L	0,799999952	0,013828263

PVT1	PASK	PVT1\PASK	0,711966693	0,038029101
PVT1	PAX1	PVT1\PAX1	0,916666567	0,001311728
PVT1	PAX9	PVT1\PAX9	0,866666615	0,004508378
PVT1	PCBD2	PVT1\PCBD2	0,695015132	0,044576719
PVT1	PCCB	PVT1\PCCB	0,711966693	0,038029101
PVT1	PCSK6	PVT1\PCSK6	0,849999964	0,006073633
PVT1	PCTP	PVT1\PCTP	0,686198473	0,046968695
PVT1	PDGFA	PVT1\PDGFA	0,783333302	0,017223325
PVT1	PDPK1	PVT1\PDPK1	0,833333313	0,008267196
PVT1	PFN4	PVT1\PFN4	0,881482542	0,003009259
PVT1	PGAP1	PVT1\PGAP1	0,933333278	0,000749559
PVT1	PIFO	PVT1\PIFO	0,783333302	0,017223325
PVT1	PKP1	PVT1\PKP1	0,799999952	0,013828263
PVT1	PKP4	PVT1\PKP4	0,866666615	0,004508378
PVT1	PLA2G12A	PVT1\PLA2G12A	0,716666639	0,036866181
PVT1	PLD2	PVT1\PLD2	0,866666615	0,004508378
PVT1	PLEK2	PVT1\PLEK2	0,745869875	0,025859788
PVT1	PNMAL1	PVT1\PNMAL1	0,711966693	0,038029101
PVT1	PNMAL2	PVT1\PNMAL2	0,799999952	0,013828263
PVT1	POLR1A	PVT1\POLR1A	0,711966693	0,038029101
PVT1	POMT2	PVT1\POMT2	0,74999994	0,025490521
PVT1	POTEE	PVT1\POTEE	0,728918254	0,031349208
PVT1	PRKAA2	PVT1\PRKAA2	0,699999928	0,043253969
PVT1	PRMT5	PVT1\PRMT5	0,711966693	0,038029101
PVT1	PRRG4	PVT1\PRRG4	0,699999928	0,043253969
PVT1	PRSS8	PVT1\PRSS8	0,799999952	0,013828263
PVT1	PSD3	PVT1\PSD3	0,74999994	0,025490521
PVT1	PSMC2	PVT1\PSMC2	0,779773057	0,016765874
PVT1	PSMD12	PVT1\PSMD12	0,813676238	0,010449735
PVT1	PTPRT	PVT1\PTPRT	0,866666615	0,004508378
PVT1	RAB14	PVT1\RAB14	0,783333302	0,017223325
PVT1	RAB23	PVT1\RAB23	0,716666639	0,036866181
PVT1	RABGAP1	PVT1\RABGAP1	0,73333329	0,031123236
PVT1	RAD50	PVT1\RAD50	0,762821436	0,021329366

PVT1	RAI1	PVT1\RAI1	0,851133168	0,005224868
PVT1	RASL10A	PVT1\RASL10A	0,744776368	0,026047178
PVT1	RBM23	PVT1\RBM23	0,745869875	0,025859788
PVT1	RFESD	PVT1\RFESD	0,745869875	0,025859788
PVT1	RMDN3	PVT1\RMDN3	0,778249502	0,017383156
PVT1	RNF170	PVT1\RNF170	0,74999994	0,025490521
PVT1	RNF32	PVT1\RNF32	0,716666639	0,036866181
PVT1	RPGRIP1L	PVT1\RPGRIP1L	0,813676238	0,010449735
PVT1	RPS6KA6	PVT1\RPS6KA6	0,833333313	0,008267196
PVT1	RRAGD	PVT1\RRAGD	0,699999928	0,043253969
PVT1	RTF1	PVT1\RTF1	0,779773057	0,016765874
PVT1	S100A14	PVT1\S100A14	0,783333302	0,017223325
PVT1	SCCPDH	PVT1\SCCPDH	0,745869875	0,025859788
PVT1	SDR42E1	PVT1\SDR42E1	0,849999964	0,006073633
PVT1	SEC61A1	PVT1\SEC61A1	0,745869875	0,025859788
PVT1	SERBP1	PVT1\SERBP1	0,74999994	0,025490521
PVT1	SGPP2	PVT1\SGPP2	0,833333313	0,008267196
PVT1	SHROOM2	PVT1\SHROOM2	0,966666639	0,000165344
PVT1	SIX1	PVT1\SIX1	0,73333329	0,031123236
PVT1	SLC15A1	PVT1\SLC15A1	0,766019881	0,019146826
PVT1	SLC22A5	PVT1\SLC22A5	0,899999917	0,002028219
PVT1	SLC30A1	PVT1\SLC30A1	0,766666651	0,021389991
PVT1	SLC30A6	PVT1\SLC30A6	0,796724617	0,013227513
PVT1	SLC45A4	PVT1\SLC45A4	0,833333313	0,008267196
PVT1	SLC46A1	PVT1\SLC46A1	0,716666639	0,036866181
PVT1	SLCO5A1	PVT1\SLCO5A1	0,766666651	0,021389991
PVT1	SMO	PVT1\SMO	0,833333313	0,008267196
PVT1	SNUPN	PVT1\SNUPN	0,762821436	0,021329366
PVT1	SNX1	PVT1\SNX1	0,73333329	0,031123236
PVT1	SOGA2	PVT1\SOGA2	0,866666615	0,004508378
PVT1	SORBS2	PVT1\SORBS2	0,883333266	0,003075397
PVT1	SORCS2	PVT1\SORCS2	0,98333329	4,96032E-05
PVT1	SPATA2	PVT1\SPATA2	0,779773057	0,016765874
PVT1	SPATA6	PVT1\SPATA6	0,849999964	0,006073633

PVT1	SPINT1	PVT1\SPINT1	0,799999952	0,013828263
PVT1	ST7L	PVT1\ST7L	0,881482542	0,003009259
PVT1	STEAP2	PVT1\STEAP2	0,849999964	0,006073633
PVT1	STK36	PVT1\STK36	0,796724617	0,013227513
PVT1	STX6	PVT1\STX6	0,699999928	0,043253969
PVT1	SUSD4	PVT1\SUSD4	0,796724617	0,013227513
PVT1	SYBU	PVT1\SYBU	0,833333313	0,008267196
PVT1	SYT1	PVT1\SYT1	0,73333329	0,031123236
PVT1	TBC1D2B	PVT1\TBC1D2B	0,711966693	0,038029101
PVT1	TCEB3	PVT1\TCEB3	0,728918254	0,031349208
PVT1	TDGF1	PVT1\TDGF1	0,933333278	0,000749559
PVT1	THAP9	PVT1\THAP9	0,728918254	0,031349208
PVT1	THSD7B	PVT1\THSD7B	0,699999928	0,043253969
PVT1	TM7SF3	PVT1\TM7SF3	0,73333329	0,031123236
PVT1	TMEM129	PVT1\TMEM129	0,769881189	0,018992504
PVT1	TMEM133	PVT1\TMEM133	0,711966693	0,038029101
PVT1	TMEM180	PVT1\TMEM180	0,728918254	0,031349208
PVT1	TMEM26	PVT1\TMEM26	0,84757942	0,00598545
PVT1	TMEM30B	PVT1\TMEM30B	0,699999928	0,043253969
PVT1	TP63	PVT1\TP63	0,883333266	0,003075397
PVT1	TRIM61	PVT1\TRIM61	0,762821436	0,021329366
PVT1	TRMT10B	PVT1\TRMT10B	0,796724617	0,013227513
PVT1	TRMT5	PVT1\TRMT5	0,830627799	0,007638889
PVT1	TRPM7	PVT1\TRPM7	0,816666603	0,010769401
PVT1	TRUB1	PVT1\TRUB1	0,695015132	0,044576719
PVT1	TSPAN13	PVT1\TSPAN13	0,816666603	0,010769401
PVT1	TTC26	PVT1\TTC26	0,745869875	0,025859788
PVT1	TYW5	PVT1\TYW5	0,881482542	0,003009259
PVT1	UBAC1	PVT1\UBAC1	0,866666615	0,004508378
PVT1	UBE2V2	PVT1\UBE2V2	0,714951873	0,034854498
PVT1	UBE3A	PVT1\UBE3A	0,84757942	0,00598545
PVT1	UBFD1	PVT1\UBFD1	0,728918254	0,031349208
PVT1	UEVLD	PVT1\UEVLD	0,793103516	0,013095238
PVT1	UNC5B	PVT1\UNC5B	0,849999964	0,006073633

PVT1	UPF1	PVT1\UPF1	0,833333313	0,008267196
PVT1	URB2	PVT1\URB2	0,830627799	0,007638889
PVT1	USP28	PVT1\USP28	0,799999952	0,013828263
PVT1	USP40	PVT1\USP40	0,899999917	0,002028219
PVT1	USP46	PVT1\USP46	0,766666651	0,021389991
PVT1	VPS35	PVT1\VPS35	0,864530981	0,004464286
PVT1	WASL	PVT1\WASL	0,695015132	0,044576719
PVT1	WDR91	PVT1\WDR91	0,916666567	0,001311728
PVT1	WTH3DI	PVT1\WTH3DI	0,73333329	0,031123236
PVT1	WWOX	PVT1\WWOX	0,78304255	0,01547619
PVT1	XPO7	PVT1\XPO7	0,762821436	0,021329366
PVT1	XYLT2	PVT1\XYLT2	0,73333329	0,031123236
PVT1	ZBTB41	PVT1\ZBTB41	0,695015132	0,044576719
PVT1	ZC3HC1	PVT1\ZC3HC1	0,745869875	0,025859788
PVT1	ZMYM6	PVT1\ZMYM6	0,728918254	0,031349208
PVT1	ZNF132	PVT1\ZNF132	0,699999928	0,043253969
PVT1	ZNF221	PVT1\ZNF221	0,728918254	0,031349208
PVT1	ZNF280B	PVT1\ZNF280B	0,816666603	0,010769401
PVT1	ZNF543	PVT1\ZNF543	0,728918254	0,031349208
PVT1	ZNF544	PVT1\ZNF544	0,762821436	0,021329366
PVT1	ZNF554	PVT1\ZNF554	0,827586234	0,008134921
PVT1	ZNF572	PVT1\ZNF572	0,881482542	0,003009259
PVT1	ZNF782	PVT1\ZNF782	0,834110498	0,007208995
PVT1	ZRANB3	PVT1\ZRANB3	0,813676238	0,010449735
PVT1	ZSCAN31	PVT1\ZSCAN31	0,813676238	0,010449735
SCARNA15	AKR1B1	SCARNA15\AKR1B1	0,833333313	0,008267196
SCARNA15	ANKMY2	SCARNA15\ANKMY2	0,699999928	0,043253969
SCARNA15	AP2B1	SCARNA15\AP2B1	0,73333329	0,031123236
SCARNA15	APPBP2	SCARNA15\APPBP2	0,816666603	0,010769401
SCARNA15	ARL6IP1	SCARNA15\ARL6IP1	0,699999928	0,043253969
SCARNA15	ARV1	SCARNA15\ARV1	0,766666651	0,021389991
SCARNA15	ATP2C1	SCARNA15\ATP2C1	0,833333313	0,008267196
SCARNA15	ATP6V1A	SCARNA15\ATP6V1A	0,73333329	0,031123236
SCARNA15	ATP6V1C1	SCARNA15\ATP6V1C1	0,699999928	0,043253969

SCARNA15	ATPIF1	SCARNA15\ATPIF1	0,716666639	0,036866181
SCARNA15	BBS4	SCARNA15\BBS4	0,816666603	0,010769401
SCARNA15	BTBD3	SCARNA15\BTBD3	0,849999964	0,006073633
SCARNA15	C14orf132	SCARNA15\C14orf132	0,686198473	0,046968695
SCARNA15	C5orf15	SCARNA15\C5orf15	0,899999917	0,002028219
SCARNA15	C5orf24	SCARNA15\C5orf24	0,783333302	0,017223325
SCARNA15	CALR	SCARNA15\CALR	0,783333302	0,017223325
SCARNA15	CCDC30	SCARNA15\CCDC30	0,853563964	0,005202822
SCARNA15	CCL25	SCARNA15\CCL25	0,816666603	0,010769401
SCARNA15	CCZ1	SCARNA15\CCZ1	0,766666651	0,021389991
SCARNA15	CD274	SCARNA15\CD274	0,74999994	0,025490521
SCARNA15	CDS1	SCARNA15\CDS1	0,699999928	0,043253969
SCARNA15	CETN3	SCARNA15\CETN3	0,799999952	0,013828263
SCARNA15	CLDN12	SCARNA15\CLDN12	0,899999917	0,002028219
SCARNA15	CLINT1	SCARNA15\CLINT1	0,766666651	0,021389991
SCARNA15	CLTC	SCARNA15\CLTC	0,783333302	0,017223325
SCARNA15	COG5	SCARNA15\COG5	0,783333302	0,017223325
SCARNA15	COG8	SCARNA15\COG8	0,903773606	0,001598325
SCARNA15	CSRP2BP	SCARNA15\CSRP2BP	0,74999994	0,025490521
SCARNA15	CUX1	SCARNA15\CUX1	0,74999994	0,025490521
SCARNA15	CYB5B	SCARNA15\CYB5B	0,716666639	0,036866181
SCARNA15	CYP26B1	SCARNA15\CYP26B1	0,833333313	0,008267196
SCARNA15	DNAI1	SCARNA15\DNAI1	0,816666603	0,010769401
SCARNA15	DNAJC19	SCARNA15\DNAJC19	0,73333329	0,031123236
SCARNA15	EIF2AK1	SCARNA15\EIF2AK1	0,799999952	0,013828263
SCARNA15	EIF3B	SCARNA15\EIF3B	0,699999928	0,043253969
SCARNA15	ELOVL6	SCARNA15\ELOVL6	0,783333302	0,017223325
SCARNA15	ERAP1	SCARNA15\ERAP1	0,799999952	0,013828263
SCARNA15	EXOSC10	SCARNA15\EXOSC10	0,783333302	0,017223325
SCARNA15	FAM120A	SCARNA15\FAM120A	0,716666639	0,036866181
SCARNA15	FAM163A	SCARNA15\FAM163A	0,716666639	0,036866181
SCARNA15	FAM199X	SCARNA15\FAM199X	0,74999994	0,025490521
SCARNA15	FAM45A	SCARNA15\FAM45A	0,716666639	0,036866181
SCARNA15	FBXO22	SCARNA15\FBXO22	0,744776368	0,026047178

SCARNA15	FKTN	SCARNA15\FKTN	0,766666651	0,021389991
SCARNA15	FLVCR1	SCARNA15\FLVCR1	0,74999994	0,025490521
SCARNA15	GEMIN5	SCARNA15\GEMIN5	0,766666651	0,021389991
SCARNA15	GGCT	SCARNA15\GGCT	0,699999928	0,043253969
SCARNA15	GINS3	SCARNA15\GINS3	0,866666615	0,004508378
SCARNA15	GNPDA2	SCARNA15\GNPDA2	0,866666615	0,004508378
SCARNA15	GOLM1	SCARNA15\GOLM1	0,849999964	0,006073633
SCARNA15	GPR64	SCARNA15\GPR64	0,716666639	0,036866181
SCARNA15	GPS1	SCARNA15\GPS1	0,699999928	0,043253969
SCARNA15	GTF2H3	SCARNA15\GTF2H3	0,833333313	0,008267196
SCARNA15	GTF2H4	SCARNA15\GTF2H4	0,711966693	0,037896827
SCARNA15	HDAC4	SCARNA15\HDAC4	0,786617756	0,015288801
SCARNA15	HDGFRP3	SCARNA15\HDGFRP3	0,766666651	0,021389991
SCARNA15	HOMER2	SCARNA15\HOMER2	0,74999994	0,025490521
SCARNA15	IFLTD1	SCARNA15\IFLTD1	0,716666639	0,036866181
SCARNA15	IGSF3	SCARNA15\IGSF3	0,783333302	0,017223325
SCARNA15	IKBIP	SCARNA15\IKBIP	0,716666639	0,036866181
SCARNA15	IQCH	SCARNA15\IQCH	0,73333329	0,031123236
SCARNA15	KDM4B	SCARNA15\KDM4B	0,783333302	0,017223325
SCARNA15	KIAA1549	SCARNA15\KIAA1549	0,699999928	0,043253969
SCARNA15	KIAA1841	SCARNA15\KIAA1841	0,716666639	0,036866181
SCARNA15	KIF3A	SCARNA15\KIF3A	0,833333313	0,008267196
SCARNA15	KLHDC10	SCARNA15\KLHDC10	0,699999928	0,043253969
SCARNA15	KTN1	SCARNA15\KTN1	0,74999994	0,025490521
SCARNA15	LAMP2	SCARNA15\LAMP2	0,719671547	0,033763226
SCARNA15	LARS	SCARNA15\LARS	0,816666603	0,010769401
SCARNA15	LGI3	SCARNA15\LGI3	0,883333266	0,003075397
SCARNA15	LRPPRC	SCARNA15\LRPPRC	0,699999928	0,043253969
SCARNA15	LZIC	SCARNA15\LZIC	0,766666651	0,021389991
SCARNA15	MAGEA3	SCARNA15\MAGEA3	0,73333329	0,031123236
SCARNA15	MAP10	SCARNA15\MAP10	0,833333313	0,008267196
SCARNA15	MAP3K2	SCARNA15\MAP3K2	0,766666651	0,021389991
SCARNA15	MED21	SCARNA15\MED21	0,728039861	0,031283069
SCARNA15	MPP5	SCARNA15\MPP5	0,799999952	0,013828263

SCARNA15	MTPAP	SCARNA15\MTPAP	0,866666615	0,004508378
SCARNA15	MTPN	SCARNA15\MTPN	0,811722577	0,010769401
SCARNA15	NDUFA4	SCARNA15\NDUFA4	0,716666639	0,036866181
SCARNA15	NDUFB5	SCARNA15\NDUFB5	0,716666639	0,036866181
SCARNA15	NETO2	SCARNA15\NETO2	0,766666651	0,021389991
SCARNA15	NIF3L1	SCARNA15\NIF3L1	0,716666639	0,036866181
SCARNA15	NPTX1	SCARNA15\NPTX1	0,849999964	0,006073633
SCARNA15	NUDT5	SCARNA15\NUDT5	0,849999964	0,006073633
SCARNA15	NVL	SCARNA15\NVL	0,849999964	0,006073633
SCARNA15	OPA1	SCARNA15\OPA1	0,949999988	0,000352734
SCARNA15	PASK	SCARNA15\PASK	0,716666639	0,036866181
SCARNA15	PDPK1	SCARNA15\PDPK1	0,699999928	0,043253969
SCARNA15	PFN4	SCARNA15\PFN4	0,73333329	0,031123236
SCARNA15	PLEK2	SCARNA15\PLEK2	0,766666651	0,021389991
SCARNA15	PNMAL1	SCARNA15\PNMAL1	0,833333313	0,008267196
SCARNA15	PNMAL2	SCARNA15\PNMAL2	0,816666603	0,010769401
SCARNA15	PNPT1	SCARNA15\PNPT1	0,73333329	0,031123236
SCARNA15	POTEE	SCARNA15\POTEE	0,74999994	0,025490521
SCARNA15	POTEM	SCARNA15\POTEM	0,699999928	0,043253969
SCARNA15	PPIP5K1	SCARNA15\PPIP5K1	0,699999928	0,043253969
SCARNA15	PRMT5	SCARNA15\PRMT5	0,883333266	0,003075397
SCARNA15	PSMC2	SCARNA15\PSMC2	0,849999964	0,006073633
SCARNA15	PSMD12	SCARNA15\PSMD12	0,816666603	0,010769401
SCARNA15	PTPRT	SCARNA15\PTPRT	0,849999964	0,006073633
SCARNA15	RAB39A	SCARNA15\RAB39A	0,783333302	0,017223325
SCARNA15	RAD50	SCARNA15\RAD50	0,766666651	0,021389991
SCARNA15	RAI1	SCARNA15\RAI1	0,786617756	0,015288801
SCARNA15	RFESD	SCARNA15\RFESD	0,783333302	0,017223325
SCARNA15	RNF32	SCARNA15\RNF32	0,699999928	0,043253969
SCARNA15	SCCPDH	SCARNA15\SCCPDH	0,883333266	0,003075397
SCARNA15	SEC61A1	SCARNA15\SEC61A1	0,74999994	0,025490521
SCARNA15	SERBP1	SCARNA15\SERBP1	0,916666567	0,001311728
SCARNA15	SF3B3	SCARNA15\SF3B3	0,716666639	0,036866181
SCARNA15	SLC15A1	SCARNA15\SLC15A1	0,79498601	0,013833774

SCARNA15	SLC22A5	SCARNA15\SLC22A5	0,699999928	0,043253969
SCARNA15	SLC30A6	SCARNA15\SLC30A6	0,816666603	0,010769401
SCARNA15	SLCO5A1	SCARNA15\SLCO5A1	0,833333313	0,008267196
SCARNA15	SNUPN	SCARNA15\SNUPN	0,73333329	0,031123236
SCARNA15	SNX1	SCARNA15\SNX1	0,816666603	0,010769401
SCARNA15	SPATA2	SCARNA15\SPATA2	0,699999928	0,043253969
SCARNA15	ST7L	SCARNA15\ST7L	0,74999994	0,025490521
SCARNA15	STX6	SCARNA15\STX6	0,833333313	0,008267196
SCARNA15	SUSD4	SCARNA15\SUSD4	0,899999917	0,002028219
SCARNA15	TBC1D2B	SCARNA15\TBC1D2B	0,699999928	0,043253969
SCARNA15	TCEB3	SCARNA15\TCEB3	0,883333266	0,003075397
SCARNA15	TDGF1	SCARNA15\TDGF1	0,716666639	0,036866181
SCARNA15	THAP9	SCARNA15\THAP9	0,799999952	0,013828263
SCARNA15	THSD7B	SCARNA15\THSD7B	0,933333278	0,000749559
SCARNA15	TMEM129	SCARNA15\TMEM129	0,711303294	0,037125219
SCARNA15	TMEM26	SCARNA15\TMEM26	0,933333278	0,000749559
SCARNA15	TOMM34	SCARNA15\TOMM34	0,73333329	0,031123236
SCARNA15	TRIM52	SCARNA15\TRIM52	0,73333329	0,031123236
SCARNA15	TRIM61	SCARNA15\TRIM61	0,74999994	0,025490521
SCARNA15	TRMT5	SCARNA15\TRMT5	0,716666639	0,036866181
SCARNA15	TRUB1	SCARNA15\TRUB1	0,783333302	0,017223325
SCARNA15	TXNL1	SCARNA15\TXNL1	0,716666639	0,036866181
SCARNA15	TYW5	SCARNA15\TYW5	0,73333329	0,031123236
SCARNA15	UBE3A	SCARNA15\UBE3A	0,916666567	0,001311728
SCARNA15	UBE3C	SCARNA15\UBE3C	0,74999994	0,025490521
SCARNA15	UBFD1	SCARNA15\UBFD1	0,74999994	0,025490521
SCARNA15	UNG	SCARNA15\UNG	0,73333329	0,031123236
SCARNA15	URB2	SCARNA15\URB2	0,933333278	0,000749559
SCARNA15	VANGL1	SCARNA15\VANGL1	0,699999928	0,043253969
SCARNA15	VPS35	SCARNA15\VPS35	0,833333313	0,008267196
SCARNA15	WDR35	SCARNA15\WDR35	0,766666651	0,021389991
SCARNA15	WDR91	SCARNA15\WDR91	0,799999952	0,013828263
SCARNA15	WWOX	SCARNA15\WWOX	0,686198473	0,046968695
SCARNA15	XPO7	SCARNA15\XPO7	0,799999952	0,013828263

SCARNA15	ZC3HC1	SCARNA15\ZC3HC1	0,766666651	0,021389991
SCARNA15	ZMYM6	SCARNA15\ZMYM6	0,74999994	0,025490521
SCARNA15	ZNF554	SCARNA15\ZNF554	0,813676238	0,010449735
SCARNA15	ZNF558	SCARNA15\ZNF558	0,816666603	0,010769401
SCARNA15	ZNF572	SCARNA15\ZNF572	0,866666615	0,004508378
SCARNA15	ZNF782	SCARNA15\ZNF782	0,945614994	0,00037478
SCARNA15	ZRANB3	SCARNA15\ZRANB3	0,833333313	0,008267196
SMC5-AS1	ACAD10	SMC5-AS1\ACAD10	0,753144681	0,023743385
SMC5-AS1	AFG3L2	SMC5-AS1\AFG3L2	0,699999928	0,043253969
SMC5-AS1	AGPAT3	SMC5-AS1\AGPAT3	0,766666651	0,021389991
SMC5-AS1	AJUBA	SMC5-AS1\AJUBA	0,74999994	0,025490521
SMC5-AS1	ALDH7A1	SMC5-AS1\ALDH7A1	0,74999994	0,025490521
SMC5-AS1	ALS2	SMC5-AS1\ALS2	0,699999928	0,043253969
SMC5-AS1	ANK3	SMC5-AS1\ANK3	0,916666567	0,001311728
SMC5-AS1	ANKFY1	SMC5-AS1\ANKFY1	0,883333266	0,003075397
SMC5-AS1	ANKMY2	SMC5-AS1\ANKMY2	0,716666639	0,036866181
SMC5-AS1	AP1S1	SMC5-AS1\AP1S1	0,816666603	0,010769401
SMC5-AS1	AP2B1	SMC5-AS1\AP2B1	0,833333313	0,008267196
SMC5-AS1	APOBEC2	SMC5-AS1\APOBEC2	0,933333278	0,000749559
SMC5-AS1	APTX	SMC5-AS1\APTX	0,833333313	0,008267196
SMC5-AS1	ARL6IP1	SMC5-AS1\ARL6IP1	0,866666615	0,004508378
SMC5-AS1	ARV1	SMC5-AS1\ARV1	0,849999964	0,006073633
SMC5-AS1	ATP2C1	SMC5-AS1\ATP2C1	0,766666651	0,021389991
SMC5-AS1	ATP6AP2	SMC5-AS1\ATP6AP2	0,699999928	0,043253969
SMC5-AS1	ATP6V0B	SMC5-AS1\ATP6V0B	0,783333302	0,017223325
SMC5-AS1	ATP6V0D1	SMC5-AS1\ATP6V0D1	0,716666639	0,036866181
SMC5-AS1	ATP6V1C1	SMC5-AS1\ATP6V1C1	0,866666615	0,004508378
SMC5-AS1	ATPAF1	SMC5-AS1\ATPAF1	0,766666651	0,021389991
SMC5-AS1	ATRNL1	SMC5-AS1\ATRNL1	0,849999964	0,006073633
SMC5-AS1	AUTS2	SMC5-AS1\AUTS2	0,699999928	0,043253969
SMC5-AS1	BBS4	SMC5-AS1\BBS4	0,833333313	0,008267196
SMC5-AS1	BRD1	SMC5-AS1\BRD1	0,833333313	0,008267196
SMC5-AS1	BTBD3	SMC5-AS1\BTBD3	0,73333329	0,031123236
SMC5-AS1	C15orf41	SMC5-AS1\C15orf41	0,949999988	0,000352734

SMC5-AS1	C19orf54	SMC5-AS1\C19orf54	0,74999994	0,025490521
SMC5-AS1	C19orf82	SMC5-AS1\C19orf82	0,933333278	0,000749559
SMC5-AS1	C1orf109	SMC5-AS1\C1orf109	0,799999952	0,013828263
SMC5-AS1	CALML3	SMC5-AS1\CALML3	0,73333329	0,031123236
SMC5-AS1	CAMKK1	SMC5-AS1\CAMKK1	0,699999928	0,043253969
SMC5-AS1	CAMSAP1	SMC5-AS1\CAMSAP1	0,949999988	0,000352734
SMC5-AS1	CBLN3	SMC5-AS1\CBLN3	0,73333329	0,031123236
SMC5-AS1	CBS	SMC5-AS1\CBS	0,833333313	0,008267196
SMC5-AS1	CCDC30	SMC5-AS1\CCDC30	0,783333302	0,017223325
SMC5-AS1	CCDC8	SMC5-AS1\CCDC8	0,833333313	0,008267196
SMC5-AS1	CCL25	SMC5-AS1\CCL25	0,716666639	0,036866181
SMC5-AS1	CDH1	SMC5-AS1\CDH1	0,783333302	0,017223325
SMC5-AS1	CDKL3	SMC5-AS1\CDKL3	0,833333313	0,008267196
SMC5-AS1	CDS1	SMC5-AS1\CDS1	0,849999964	0,006073633
SMC5-AS1	CETN3	SMC5-AS1\CETN3	0,799999952	0,013828263
SMC5-AS1	CLCN5	SMC5-AS1\CLCN5	0,73333329	0,031123236
SMC5-AS1	CLDN12	SMC5-AS1\CLDN12	0,883333266	0,003075397
SMC5-AS1	CLINT1	SMC5-AS1\CLINT1	0,816666603	0,010769401
SMC5-AS1	CLTC	SMC5-AS1\CLTC	0,816666603	0,010769401
SMC5-AS1	CMPK2	SMC5-AS1\CMPK2	0,699999928	0,043253969
SMC5-AS1	CNTNAP3	SMC5-AS1\CNTNAP3	0,766666651	0,021389991
SMC5-AS1	CNTNAP3B	SMC5-AS1\CNTNAP3B	0,833333313	0,008267196
SMC5-AS1	COG5	SMC5-AS1\COG5	0,799999952	0,013828263
SMC5-AS1	COG8	SMC5-AS1\COG8	0,694566727	0,043816138
SMC5-AS1	CROT	SMC5-AS1\CROT	0,949999988	0,000352734
SMC5-AS1	CSRP2BP	SMC5-AS1\CSRP2BP	0,716666639	0,036866181
SMC5-AS1	CUX1	SMC5-AS1\CUX1	0,833333313	0,008267196
SMC5-AS1	DCAKD	SMC5-AS1\DCAKD	0,716666639	0,036866181
SMC5-AS1	DDX31	SMC5-AS1\DDX31	0,833333313	0,008267196
SMC5-AS1	DET1	SMC5-AS1\DET1	0,783333302	0,017223325
SMC5-AS1	DHTKD1	SMC5-AS1\DHTKD1	0,933333278	0,000749559
SMC5-AS1	DHX32	SMC5-AS1\DHX32	0,699999928	0,043253969
SMC5-AS1	DMRT2	SMC5-AS1\DMRT2	0,833333313	0,008267196
SMC5-AS1	DMRTA1	SMC5-AS1\DMRTA1	0,716666639	0,036866181

SMC5-AS1	DNAJC16	SMC5-AS1\DNAJC16	0,98333329	4,96032E-05
SMC5-AS1	DPH6	SMC5-AS1\DPH6	0,899999917	0,002028219
SMC5-AS1	DTNB	SMC5-AS1\DTNB	0,833333313	0,008267196
SMC5-AS1	DUOX1	SMC5-AS1\DUOX1	0,928878427	0,000815697
SMC5-AS1	DUOXA1	SMC5-AS1\DUOXA1	0,816666603	0,010769401
SMC5-AS1	EIF2AK1	SMC5-AS1\EIF2AK1	0,74999994	0,025490521
SMC5-AS1	ELOVL6	SMC5-AS1\ELOVL6	0,866666615	0,004508378
SMC5-AS1	ENPEP	SMC5-AS1\ENPEP	0,766666651	0,021389991
SMC5-AS1	EPN3	SMC5-AS1\EPN3	0,883333266	0,003075397
SMC5-AS1	EPS15L1	SMC5-AS1\EPS15L1	0,766666651	0,021389991
SMC5-AS1	ESRP1	SMC5-AS1\ESRP1	0,899999917	0,002028219
SMC5-AS1	ESRP2	SMC5-AS1\ESRP2	0,866666615	0,004508378
SMC5-AS1	EVA1A	SMC5-AS1\EVA1A	0,716666639	0,036866181
SMC5-AS1	EXOC7	SMC5-AS1\EXOC7	0,762821436	0,021329366
SMC5-AS1	EYA2	SMC5-AS1\EYA2	0,816666603	0,010769401
SMC5-AS1	FAHD1	SMC5-AS1\FAHD1	0,816666603	0,010769401
SMC5-AS1	FAM120A	SMC5-AS1\FAM120A	0,799999952	0,013828263
SMC5-AS1	FAM154B	SMC5-AS1\FAM154B	0,779773057	0,016765874
SMC5-AS1	FAM199X	SMC5-AS1\FAM199X	0,833333313	0,008267196
SMC5-AS1	FBXO22	SMC5-AS1\FBXO22	0,786617756	0,015288801
SMC5-AS1	FBXW11	SMC5-AS1\FBXW11	0,813676238	0,010449735
SMC5-AS1	FEM1B	SMC5-AS1\FEM1B	0,849999964	0,006073633
SMC5-AS1	FKTN	SMC5-AS1\FKTN	0,866666615	0,004508378
SMC5-AS1	FLVCR1	SMC5-AS1\FLVCR1	0,866666615	0,004508378
SMC5-AS1	FOXE1	SMC5-AS1\FOXE1	0,74999994	0,025490521
SMC5-AS1	FSTL4	SMC5-AS1\FSTL4	0,833333313	0,008267196
SMC5-AS1	GGA1	SMC5-AS1\GGA1	0,716666639	0,036866181
SMC5-AS1	GGA2	SMC5-AS1\GGA2	0,98333329	4,96032E-05
SMC5-AS1	GGCT	SMC5-AS1\GGCT	0,849999964	0,006073633
SMC5-AS1	GINS3	SMC5-AS1\GINS3	0,766666651	0,021389991
SMC5-AS1	GLI2	SMC5-AS1\GLI2	0,899999917	0,002028219
SMC5-AS1	GNPDA2	SMC5-AS1\GNPDA2	0,716666639	0,036866181
SMC5-AS1	GPHN	SMC5-AS1\GPHN	0,73333329	0,031123236
SMC5-AS1	GPS1	SMC5-AS1\GPS1	0,916666567	0,001311728

SMC5-AS1	GRHL2	SMC5-AS1\GRHL2	0,799999952	0,013828263
SMC5-AS1	GRIP1	SMC5-AS1\GRIP1	0,783333302	0,017223325
SMC5-AS1	GRTP1	SMC5-AS1\GRTP1	0,799999952	0,013828263
SMC5-AS1	GTF2H3	SMC5-AS1\GTF2H3	0,716666639	0,036866181
SMC5-AS1	GTF2H4	SMC5-AS1\GTF2H4	0,716666639	0,036866181
SMC5-AS1	GTF2I	SMC5-AS1\GTF2I	0,799999952	0,013828263
SMC5-AS1	GTF3C4	SMC5-AS1\GTF3C4	0,799999952	0,013828263
SMC5-AS1	HIST2H2BF	SMC5-AS1\HIST2H2BF	0,916666567	0,001311728
SMC5-AS1	HN1L	SMC5-AS1\HN1L	0,866666615	0,004508378
SMC5-AS1	HOMER2	SMC5-AS1\HOMER2	0,883333266	0,003075397
SMC5-AS1	HPS5	SMC5-AS1\HPS5	0,74999994	0,025490521
SMC5-AS1	HPSE	SMC5-AS1\HPSE	0,866666615	0,004508378
SMC5-AS1	IDH3G	SMC5-AS1\IDH3G	0,74999994	0,025490521
SMC5-AS1	IGSF3	SMC5-AS1\IGSF3	0,73333329	0,031123236
SMC5-AS1	IQCH	SMC5-AS1\IQCH	0,799999952	0,013828263
SMC5-AS1	ITGA2	SMC5-AS1\ITGA2	0,799999952	0,013828263
SMC5-AS1	KDM4D	SMC5-AS1\KDM4D	0,962351501	0,000165344
SMC5-AS1	KIAA1549	SMC5-AS1\KIAA1549	0,866666615	0,004508378
SMC5-AS1	KLHDC10	SMC5-AS1\KLHDC10	0,866666615	0,004508378
SMC5-AS1	KRTAP5-8	SMC5-AS1\KRTAP5-8	0,766666651	0,021389991
SMC5-AS1	LAMP1	SMC5-AS1\LAMP1	0,761512935	0,021263227
SMC5-AS1	LAMP2	SMC5-AS1\LAMP2	0,836827397	0,00696649
SMC5-AS1	LARS	SMC5-AS1\LARS	0,74999994	0,025490521
SMC5-AS1	LIMK1	SMC5-AS1\LIMK1	0,903773606	0,001598325
SMC5-AS1	LMBR1	SMC5-AS1\LMBR1	0,833333313	0,008267196
SMC5-AS1	LRIG3	SMC5-AS1\LRIG3	0,766666651	0,021389991
SMC5-AS1	LRPPRC	SMC5-AS1\LRPPRC	0,849999964	0,006073633
SMC5-AS1	M1AP	SMC5-AS1\M1AP	0,883333266	0,003075397
SMC5-AS1	MAML3	SMC5-AS1\MAML3	0,99999994	5,51146E-06
SMC5-AS1	MAP3K2	SMC5-AS1\MAP3K2	0,866666615	0,004508378
SMC5-AS1	MARVELD2	SMC5-AS1\MARVELD2	0,849999964	0,006073633
SMC5-AS1	MCOLN3	SMC5-AS1\MCOLN3	0,916666567	0,001311728
SMC5-AS1	MCTP2	SMC5-AS1\MCTP2	0,799999952	0,013828263
SMC5-AS1	MED22	SMC5-AS1\MED22	0,711966693	0,037896827

SMC5-AS1	METTL8	SMC5-AS1\METTL8	0,833333313	0,008267196
SMC5-AS1	MFSD3	SMC5-AS1\MFSD3	0,933333278	0,000749559
SMC5-AS1	MPP5	SMC5-AS1\MPP5	0,849999964	0,006073633
SMC5-AS1	MRPL40	SMC5-AS1\MRPL40	0,699999928	0,043253969
SMC5-AS1	MTPN	SMC5-AS1\MTPN	0,744776368	0,026047178
SMC5-AS1	MTSS1L	SMC5-AS1\MTSS1L	0,73333329	0,031123236
SMC5-AS1	MYO10	SMC5-AS1\MYO10	0,833333313	0,008267196
SMC5-AS1	N4BP1	SMC5-AS1\N4BP1	0,883333266	0,003075397
SMC5-AS1	NAA50	SMC5-AS1\NAA50	0,766666651	0,021389991
SMC5-AS1	NAGK	SMC5-AS1\NAGK	0,899999917	0,002028219
SMC5-AS1	NCS1	SMC5-AS1\NCS1	0,833333313	0,008267196
SMC5-AS1	NDUFA4	SMC5-AS1\NDUFA4	0,833333313	0,008267196
SMC5-AS1	NDUFB5	SMC5-AS1\NDUFB5	0,799999952	0,013828263
SMC5-AS1	NETO2	SMC5-AS1\NETO2	0,849999964	0,006073633
SMC5-AS1	NHLRC3	SMC5-AS1\NHLRC3	0,73333329	0,031123236
SMC5-AS1	NIPSNAP1	SMC5-AS1\NIPSNAP1	0,883333266	0,003075397
SMC5-AS1	NPNT	SMC5-AS1\NPNT	0,699999928	0,043253969
SMC5-AS1	NRCAM	SMC5-AS1\NRCAM	0,916666567	0,001311728
SMC5-AS1	NUDT12	SMC5-AS1\NUDT12	0,928878427	0,000815697
SMC5-AS1	NXN	SMC5-AS1\NXN	0,711303294	0,037125219
SMC5-AS1	OSBPL3	SMC5-AS1\OSBPL3	0,916666567	0,001311728
SMC5-AS1	OXCT1	SMC5-AS1\OXCT1	0,916666567	0,001311728
SMC5-AS1	PABPC4L	SMC5-AS1\PABPC4L	0,833333313	0,008267196
SMC5-AS1	PANK1	SMC5-AS1\PANK1	0,883333266	0,003075397
SMC5-AS1	PASK	SMC5-AS1\PASK	0,899999917	0,002028219
SMC5-AS1	PAX1	SMC5-AS1\PAX1	0,716666639	0,036866181
SMC5-AS1	PAX9	SMC5-AS1\PAX9	0,916666567	0,001311728
SMC5-AS1	PCCB	SMC5-AS1\PCCB	0,73333329	0,031123236
SMC5-AS1	PGAP1	SMC5-AS1\PGAP1	0,783333302	0,017223325
SMC5-AS1	PIAS2	SMC5-AS1\PIAS2	0,716666639	0,036866181
SMC5-AS1	PIFO	SMC5-AS1\PIFO	0,866666615	0,004508378
SMC5-AS1	PKP1	SMC5-AS1\PKP1	0,783333302	0,017223325
SMC5-AS1	PKP4	SMC5-AS1\PKP4	0,866666615	0,004508378
SMC5-AS1	PLA2G12A	SMC5-AS1\PLA2G12A	0,883333266	0,003075397

SMC5-AS1	PLD2	SMC5-AS1\PLD2	0,833333313	0,008267196
SMC5-AS1	PLEK2	SMC5-AS1\PLEK2	0,833333313	0,008267196
SMC5-AS1	PMPCB	SMC5-AS1\PMPCB	0,966666639	0,000165344
SMC5-AS1	PNPT1	SMC5-AS1\PNPT1	0,716666639	0,036866181
SMC5-AS1	PPP5C	SMC5-AS1\PPP5C	0,783333302	0,017223325
SMC5-AS1	PRRC1	SMC5-AS1\PRRC1	0,866666615	0,004508378
SMC5-AS1	PRRG4	SMC5-AS1\PRRG4	0,716666639	0,036866181
SMC5-AS1	PRSS8	SMC5-AS1\PRSS8	0,933333278	0,000749559
SMC5-AS1	PSD3	SMC5-AS1\PSD3	0,916666567	0,001311728
SMC5-AS1	PSMC2	SMC5-AS1\PSMC2	0,766666651	0,021389991
SMC5-AS1	PSMD12	SMC5-AS1\PSMD12	0,833333313	0,008267196
SMC5-AS1	PTPRK	SMC5-AS1\PTPRK	0,716666639	0,036866181
SMC5-AS1	PTPRT	SMC5-AS1\PTPRT	0,783333302	0,017223325
SMC5-AS1	RAB14	SMC5-AS1\RAB14	0,849999964	0,006073633
SMC5-AS1	RAB23	SMC5-AS1\RAB23	0,73333329	0,031123236
SMC5-AS1	RAB40C	SMC5-AS1\RAB40C	0,883333266	0,003075397
SMC5-AS1	RABGAP1	SMC5-AS1\RABGAP1	0,716666639	0,036866181
SMC5-AS1	RAD50	SMC5-AS1\RAD50	0,866666615	0,004508378
SMC5-AS1	RANBP17	SMC5-AS1\RANBP17	0,699999928	0,043253969
SMC5-AS1	RASL10A	SMC5-AS1\RASL10A	0,786617756	0,015288801
SMC5-AS1	RBM23	SMC5-AS1\RBM23	0,699999928	0,043253969
SMC5-AS1	RFESD	SMC5-AS1\RFESD	0,866666615	0,004508378
SMC5-AS1	RMDN3	SMC5-AS1\RMDN3	0,953983247	0,000253527
SMC5-AS1	RNF170	SMC5-AS1\RNF170	0,766666651	0,021389991
SMC5-AS1	RPGRIP1L	SMC5-AS1\RPGRIP1L	0,783333302	0,017223325
SMC5-AS1	RPS6KA6	SMC5-AS1\RPS6KA6	0,766666651	0,021389991
SMC5-AS1	RRAGB	SMC5-AS1\RRAGB	0,816666603	0,010769401
SMC5-AS1	RRAGD	SMC5-AS1\RRAGD	0,716666639	0,036866181
SMC5-AS1	RTF1	SMC5-AS1\RTF1	0,833333313	0,008267196
SMC5-AS1	S100A14	SMC5-AS1\S100A14	0,849999964	0,006073633
SMC5-AS1	SDR42E1	SMC5-AS1\SDR42E1	0,866666615	0,004508378
SMC5-AS1	SFXN5	SMC5-AS1\SFXN5	0,719671547	0,033763226
SMC5-AS1	SGPL1	SMC5-AS1\SGPL1	0,833333313	0,008267196
SMC5-AS1	SGPP2	SMC5-AS1\SGPP2	0,74999994	0,025490521

SMC5-AS1	SIX4	SMC5-AS1\SIX4	0,766666651	0,021389991
SMC5-AS1	SLC22A5	SMC5-AS1\SLC22A5	0,799999952	0,013828263
SMC5-AS1	SLC30A1	SMC5-AS1\SLC30A1	0,799999952	0,013828263
SMC5-AS1	SLC30A6	SMC5-AS1\SLC30A6	0,783333302	0,017223325
SMC5-AS1	SLC44A3	SMC5-AS1\SLC44A3	0,849999964	0,006073633
SMC5-AS1	SLC46A1	SMC5-AS1\SLC46A1	0,899999917	0,002028219
SMC5-AS1	SMO	SMC5-AS1\SMO	0,766666651	0,021389991
SMC5-AS1	SNX1	SMC5-AS1\SNX1	0,849999964	0,006073633
SMC5-AS1	SOGA2	SMC5-AS1\SOGA2	0,833333313	0,008267196
SMC5-AS1	SORBS2	SMC5-AS1\SORBS2	0,866666615	0,004508378
SMC5-AS1	SORCS2	SMC5-AS1\SORCS2	0,716666639	0,036866181
SMC5-AS1	SPANXN2	SMC5-AS1\SPANXN2	0,73333329	0,031123236
SMC5-AS1	SPATA6	SMC5-AS1\SPATA6	0,799999952	0,013828263
SMC5-AS1	SPIN1	SMC5-AS1\SPIN1	0,716666639	0,036866181
SMC5-AS1	SPINT1	SMC5-AS1\SPINT1	0,799999952	0,013828263
SMC5-AS1	SPIRE2	SMC5-AS1\SPIRE2	0,870300472	0,003681658
SMC5-AS1	SPRY3	SMC5-AS1\SPRY3	0,74999994	0,025490521
SMC5-AS1	STARD7	SMC5-AS1\STARD7	0,866666615	0,004508378
SMC5-AS1	STEAP2	SMC5-AS1\STEAP2	0,783333302	0,017223325
SMC5-AS1	STX6	SMC5-AS1\STX6	0,833333313	0,008267196
SMC5-AS1	SUPT3H	SMC5-AS1\SUPT3H	0,753144681	0,023533951
SMC5-AS1	SUSD4	SMC5-AS1\SUSD4	0,716666639	0,036866181
SMC5-AS1	SYBU	SMC5-AS1\SYBU	0,883333266	0,003075397
SMC5-AS1	TCEB3	SMC5-AS1\TCEB3	0,699999928	0,043253969
SMC5-AS1	TDGF1	SMC5-AS1\TDGF1	0,699999928	0,043253969
SMC5-AS1	THSD7B	SMC5-AS1\THSD7B	0,699999928	0,043253969
SMC5-AS1	TM7SF3	SMC5-AS1\TM7SF3	0,766666651	0,021389991
SMC5-AS1	TMEM107	SMC5-AS1\TMEM107	0,73333329	0,031123236
SMC5-AS1	TMEM129	SMC5-AS1\TMEM129	0,711303294	0,037125219
SMC5-AS1	TMEM180	SMC5-AS1\TMEM180	0,830627799	0,007638889
SMC5-AS1	TMEM185A	SMC5-AS1\TMEM185A	0,866666615	0,004508378
SMC5-AS1	TNPO1	SMC5-AS1\TNPO1	0,73333329	0,031123236
SMC5-AS1	TP63	SMC5-AS1\TP63	0,74999994	0,025490521
SMC5-AS1	TRIM52	SMC5-AS1\TRIM52	0,699999928	0,043253969

SMC5-AS1	TRIM7	SMC5-AS1\TRIM7	0,766666651	0,021389991
SMC5-AS1	TRMT10B	SMC5-AS1\TRMT10B	0,766666651	0,021389991
SMC5-AS1	TRPM7	SMC5-AS1\TRPM7	0,916666567	0,001311728
SMC5-AS1	TRUB1	SMC5-AS1\TRUB1	0,799999952	0,013828263
SMC5-AS1	TSPAN13	SMC5-AS1\TSPAN13	0,699999928	0,043253969
SMC5-AS1	TTC22	SMC5-AS1\TTC22	0,883333266	0,003075397
SMC5-AS1	UBAC1	SMC5-AS1\UBAC1	0,816666603	0,010769401
SMC5-AS1	UBAP2	SMC5-AS1\UBAP2	0,799999952	0,013828263
SMC5-AS1	UBE2V2	SMC5-AS1\UBE2V2	0,778249502	0,018000441
SMC5-AS1	UBFD1	SMC5-AS1\UBFD1	0,883333266	0,003075397
SMC5-AS1	UBTD2	SMC5-AS1\UBTD2	0,711303294	0,037125219
SMC5-AS1	UEVLD	SMC5-AS1\UEVLD	0,762821436	0,021329366
SMC5-AS1	UNC5B	SMC5-AS1\UNC5B	0,816666603	0,010769401
SMC5-AS1	UPF1	SMC5-AS1\UPF1	0,833333313	0,008267196
SMC5-AS1	USP40	SMC5-AS1\USP40	0,799999952	0,013828263
SMC5-AS1	VAC14	SMC5-AS1\VAC14	0,949999988	0,000352734
SMC5-AS1	VANGL1	SMC5-AS1\VANGL1	0,699999928	0,043253969
SMC5-AS1	VMA21	SMC5-AS1\VMA21	0,833333313	0,008267196
SMC5-AS1	WDR3	SMC5-AS1\WDR3	0,699999928	0,043253969
SMC5-AS1	WDR91	SMC5-AS1\WDR91	0,766666651	0,021389991
SMC5-AS1	WNK2	SMC5-AS1\WNK2	0,833333313	0,008267196
SMC5-AS1	WTH3DI	SMC5-AS1\WTH3DI	0,716666639	0,036866181
SMC5-AS1	WWOX	SMC5-AS1\WWOX	0,803354323	0,012202381
SMC5-AS1	XPO7	SMC5-AS1\XPO7	0,716666639	0,036866181
SMC5-AS1	XPR1	SMC5-AS1\XPR1	0,73333329	0,031123236
SMC5-AS1	XYLT2	SMC5-AS1\XYLT2	0,899999917	0,002028219
SMC5-AS1	ZBTB41	SMC5-AS1\ZBTB41	0,883333266	0,003075397
SMC5-AS1	ZMYM3	SMC5-AS1\ZMYM3	0,73333329	0,031123236
SMC5-AS1	ZNF132	SMC5-AS1\ZNF132	0,933333278	0,000749559
SMC5-AS1	ZNF280B	SMC5-AS1\ZNF280B	0,899999917	0,002028219
SMC5-AS1	ZNF449	SMC5-AS1\ZNF449	0,845195651	0,006205908
SMC5-AS1	ZNF543	SMC5-AS1\ZNF543	0,799999952	0,013828263
SMC5-AS1	ZNF544	SMC5-AS1\ZNF544	0,783333302	0,017223325
SMC5-AS1	ZNF554	SMC5-AS1\ZNF554	0,728918254	0,031349208

SMC5-AS1	ZNF562	SMC5-AS1\ZNF562	0,73333329	0,031123236
SMC5-AS1	ZRANB3	SMC5-AS1\ZRANB3	0,716666639	0,036866181
SMC5-AS1	ZSCAN31	SMC5-AS1\ZSCAN31	0,783333302	0,017223325
SPTY2D1-AS1	ACAD10	SPTY2D1-AS1\ACAD10	0,840336144	0,006602734
SPTY2D1-AS1	AJUBA	SPTY2D1-AS1\AJUBA	0,803354323	0,012202381
SPTY2D1-AS1	AK3	SPTY2D1-AS1\AK3	0,728039861	0,031283069
SPTY2D1-AS1	ALS2	SPTY2D1-AS1\ALS2	0,82009083	0,009281305
SPTY2D1-AS1	ANK3	SPTY2D1-AS1\ANK3	0,728039861	0,031283069
SPTY2D1-AS1	ANKMY2	SPTY2D1-AS1\ANKMY2	0,803354323	0,012202381
SPTY2D1-AS1	AP1S1	SPTY2D1-AS1\AP1S1	0,769881189	0,018992504
SPTY2D1-AS1	AP2B1	SPTY2D1-AS1\AP2B1	0,769881189	0,018992504
SPTY2D1-AS1	APOBEC2	SPTY2D1-AS1\APOBEC2	0,828459144	0,008289241
SPTY2D1-AS1	APPBP2	SPTY2D1-AS1\APPBP2	0,728039861	0,031283069
SPTY2D1-AS1	APTX	SPTY2D1-AS1\APTX	0,811722577	0,010769401
SPTY2D1-AS1	ARL6IP1	SPTY2D1-AS1\ARL6IP1	0,91214186	0,001322751
SPTY2D1-AS1	ARV1	SPTY2D1-AS1\ARV1	0,878668785	0,003218695
SPTY2D1-AS1	ATP2C1	SPTY2D1-AS1\ATP2C1	0,895405293	0,002105379
SPTY2D1-AS1	ATP6V0B	SPTY2D1-AS1\ATP6V0B	0,786617756	0,015288801
SPTY2D1-AS1	ATP6V1A	SPTY2D1-AS1\ATP6V1A	0,736408114	0,02800926
SPTY2D1-AS1	ATP6V1C1	SPTY2D1-AS1\ATP6V1C1	0,878668785	0,003218695
SPTY2D1-AS1	ATPAF1	SPTY2D1-AS1\ATPAF1	0,70293504	0,040145501
SPTY2D1-AS1	ATPIF1	SPTY2D1-AS1\ATPIF1	0,769881189	0,018992504
SPTY2D1-AS1	ATRNL1	SPTY2D1-AS1\ATRNL1	0,728039861	0,031283069
SPTY2D1-AS1	BBS4	SPTY2D1-AS1\BBS4	0,920510173	0,000981041
SPTY2D1-AS1	BRD1	SPTY2D1-AS1\BRD1	0,761512935	0,021263227
SPTY2D1-AS1	BTBD3	SPTY2D1-AS1\BTBD3	0,91214186	0,001322751
SPTY2D1-AS1	C15orf41	SPTY2D1-AS1\C15orf41	0,82009083	0,009281305
SPTY2D1-AS1	C19orf82	SPTY2D1-AS1\C19orf82	0,694566727	0,043816138
SPTY2D1-AS1	C1orf109	SPTY2D1-AS1\C1orf109	0,861932218	0,004376102
SPTY2D1-AS1	C1orf27	SPTY2D1-AS1\C1orf27	0,836827397	0,00696649
SPTY2D1-AS1	C5orf15	SPTY2D1-AS1\C5orf15	0,736408114	0,02800926
SPTY2D1-AS1	C5orf24	SPTY2D1-AS1\C5orf24	0,719671547	0,033763226
SPTY2D1-AS1	CALR	SPTY2D1-AS1\CALR	0,70293504	0,040145501
SPTY2D1-AS1	CBS	SPTY2D1-AS1\CBS	0,91214186	0,001322751

SPTY2D1-AS1	CCDC30	SPTY2D1-AS1\CCDC30	0,887037039	0,002469136
SPTY2D1-AS1	CCDC8	SPTY2D1-AS1\CCDC8	0,70293504	0,040145501
SPTY2D1-AS1	CCL25	SPTY2D1-AS1\CCL25	0,769881189	0,018992504
SPTY2D1-AS1	CDKL3	SPTY2D1-AS1\CDKL3	0,803354323	0,012202381
SPTY2D1-AS1	CDS1	SPTY2D1-AS1\CDS1	0,861932218	0,004376102
SPTY2D1-AS1	CETN3	SPTY2D1-AS1\CETN3	0,928878427	0,000815697
SPTY2D1-AS1	CLCA1	SPTY2D1-AS1\CLCA1	0,747302234	0,022089947
SPTY2D1-AS1	CLDN12	SPTY2D1-AS1\CLDN12	0,769881189	0,018992504
SPTY2D1-AS1	CLINT1	SPTY2D1-AS1\CLINT1	0,870300472	0,003681658
SPTY2D1-AS1	CLTC	SPTY2D1-AS1\CLTC	0,79498601	0,013833774
SPTY2D1-AS1	CMPK2	SPTY2D1-AS1\CMPK2	0,803354323	0,012202381
SPTY2D1-AS1	CNTNAP3	SPTY2D1-AS1\CNTNAP3	0,753144681	0,023533951
SPTY2D1-AS1	CNTNAP3B	SPTY2D1-AS1\CNTNAP3B	0,828459144	0,008289241
SPTY2D1-AS1	COG5	SPTY2D1-AS1\COG5	0,903773606	0,001598325
SPTY2D1-AS1	COG8	SPTY2D1-AS1\COG8	0,861344516	0,004354056
SPTY2D1-AS1	CSRP2BP	SPTY2D1-AS1\CSRP2BP	0,828459144	0,008289241
SPTY2D1-AS1	CUX1	SPTY2D1-AS1\CUX1	0,803354323	0,012202381
SPTY2D1-AS1	CYB5B	SPTY2D1-AS1\CYB5B	0,761512935	0,021263227
SPTY2D1-AS1	DDX31	SPTY2D1-AS1\DDX31	0,744776368	0,026047178
SPTY2D1-AS1	DET1	SPTY2D1-AS1\DET1	0,878668785	0,003218695
SPTY2D1-AS1	DHTKD1	SPTY2D1-AS1\DHTKD1	0,845195651	0,006205908
SPTY2D1-AS1	DHX32	SPTY2D1-AS1\DHX32	0,79498601	0,013833774
SPTY2D1-AS1	DMRT2	SPTY2D1-AS1\DMRT2	0,828459144	0,008289241
SPTY2D1-AS1	DNAJC16	SPTY2D1-AS1\DNAJC16	0,736408114	0,02800926
SPTY2D1-AS1	DNAJC19	SPTY2D1-AS1\DNAJC19	0,728039861	0,031283069
SPTY2D1-AS1	DNAL4	SPTY2D1-AS1\DNAL4	0,79498601	0,013833774
SPTY2D1-AS1	DPH6	SPTY2D1-AS1\DPH6	0,753144681	0,023533951
SPTY2D1-AS1	DUOXA1	SPTY2D1-AS1\DUOXA1	0,711303294	0,037125219
SPTY2D1-AS1	EDA2R	SPTY2D1-AS1\EDA2R	0,694566727	0,043816138
SPTY2D1-AS1	EIF2AK1	SPTY2D1-AS1\EIF2AK1	0,753144681	0,023533951
SPTY2D1-AS1	EIF2AK4	SPTY2D1-AS1\EIF2AK4	0,694566727	0,043816138
SPTY2D1-AS1	ELOVL6	SPTY2D1-AS1\ELOVL6	0,82009083	0,009281305
SPTY2D1-AS1	ENPEP	SPTY2D1-AS1\ENPEP	0,82009083	0,009281305
SPTY2D1-AS1	EPS15L1	SPTY2D1-AS1\EPS15L1	0,686198473	0,046968695

SPTY2D1-AS1	EXOSC10	SPTY2D1-AS1\EXOSC10	0,719671547	0,033763226
SPTY2D1-AS1	FAF2	SPTY2D1-AS1\FAF2	0,719671547	0,033763226
SPTY2D1-AS1	FAM120A	SPTY2D1-AS1\FAM120A	0,853563964	0,005202822
SPTY2D1-AS1	FAM163A	SPTY2D1-AS1\FAM163A	0,686198473	0,046968695
SPTY2D1-AS1	FAM199X	SPTY2D1-AS1\FAM199X	0,887037039	0,002469136
SPTY2D1-AS1	FAM218A	SPTY2D1-AS1\FAM218A	0,753144681	0,023533951
SPTY2D1-AS1	FAM45A	SPTY2D1-AS1\FAM45A	0,82009083	0,009281305
SPTY2D1-AS1	FBXO22	SPTY2D1-AS1\FBXO22	0,878151238	0,003053351
SPTY2D1-AS1	FEM1B	SPTY2D1-AS1\FEM1B	0,736408114	0,02800926
SPTY2D1-AS1	FKTN	SPTY2D1-AS1\FKTN	0,945614994	0,00037478
SPTY2D1-AS1	FLVCR1	SPTY2D1-AS1\FLVCR1	0,861932218	0,004376102
SPTY2D1-AS1	GGA1	SPTY2D1-AS1\GGA1	0,845195651	0,006205908
SPTY2D1-AS1	GGA2	SPTY2D1-AS1\GGA2	0,70293504	0,040145501
SPTY2D1-AS1	GGCT	SPTY2D1-AS1\GGCT	0,945614994	0,00037478
SPTY2D1-AS1	GINS3	SPTY2D1-AS1\GINS3	0,761512935	0,021263227
SPTY2D1-AS1	GNPDA2	SPTY2D1-AS1\GNPDA2	0,928878427	0,000815697
SPTY2D1-AS1	GOLM1	SPTY2D1-AS1\GOLM1	0,828459144	0,008289241
SPTY2D1-AS1	GPR64	SPTY2D1-AS1\GPR64	0,811722577	0,010769401
SPTY2D1-AS1	GPS1	SPTY2D1-AS1\GPS1	0,728039861	0,031283069
SPTY2D1-AS1	GRIP1	SPTY2D1-AS1\GRIP1	0,711303294	0,037125219
SPTY2D1-AS1	GTF2H3	SPTY2D1-AS1\GTF2H3	0,93724668	0,000485009
SPTY2D1-AS1	GTF2H4	SPTY2D1-AS1\GTF2H4	0,766019881	0,019708995
SPTY2D1-AS1	GTF3C4	SPTY2D1-AS1\GTF3C4	0,870300472	0,003681658
SPTY2D1-AS1	HDGFRP3	SPTY2D1-AS1\HDGFRP3	0,694566727	0,043816138
SPTY2D1-AS1	HIST1H2BC	SPTY2D1-AS1\HIST1H2BC	0,887037039	0,002469136
SPTY2D1-AS1	HIST2H2BF	SPTY2D1-AS1\HIST2H2BF	0,895405293	0,002105379
SPTY2D1-AS1	HN1L	SPTY2D1-AS1\HN1L	0,82009083	0,009281305
SPTY2D1-AS1	HOMER2	SPTY2D1-AS1\HOMER2	0,928878427	0,000815697
SPTY2D1-AS1	HOMER3	SPTY2D1-AS1\HOMER3	0,711303294	0,037125219
SPTY2D1-AS1	HPS5	SPTY2D1-AS1\HPS5	0,753144681	0,023533951
SPTY2D1-AS1	HPSE	SPTY2D1-AS1\HPSE	0,928878427	0,000815697
SPTY2D1-AS1	IDH3G	SPTY2D1-AS1\IDH3G	0,811722577	0,010769401
SPTY2D1-AS1	IFI44	SPTY2D1-AS1\IFI44	0,711303294	0,037125219
SPTY2D1-AS1	IGSF3	SPTY2D1-AS1\IGSF3	0,945614994	0,00037478

SPTY2D1-AS1	IKBIP	SPTY2D1-AS1\IKBIP	0,753144681	0,023533951
SPTY2D1-AS1	KAL1	SPTY2D1-AS1\KAL1	0,711303294	0,037125219
SPTY2D1-AS1	KDM4D	SPTY2D1-AS1\KDM4D	0,693277299	0,042779982
SPTY2D1-AS1	KIAA1549	SPTY2D1-AS1\KIAA1549	0,878668785	0,003218695
SPTY2D1-AS1	KIF3A	SPTY2D1-AS1\KIF3A	0,828459144	0,008289241
SPTY2D1-AS1	KLHDC10	SPTY2D1-AS1\KLHDC10	0,895405293	0,002105379
SPTY2D1-AS1	KRTAP5-8	SPTY2D1-AS1\KRTAP5-8	0,728039861	0,031283069
SPTY2D1-AS1	KTN1	SPTY2D1-AS1\KTN1	0,719671547	0,033763226
SPTY2D1-AS1	LAMP2	SPTY2D1-AS1\LAMP2	0,768907547	0,018628748
SPTY2D1-AS1	LARS	SPTY2D1-AS1\LARS	0,93724668	0,000485009
SPTY2D1-AS1	LEPREL1	SPTY2D1-AS1\LEPREL1	0,728039861	0,031283069
SPTY2D1-AS1	LGI3	SPTY2D1-AS1\LGI3	0,761512935	0,021263227
SPTY2D1-AS1	LIMK1	SPTY2D1-AS1\LIMK1	0,735294104	0,027854938
SPTY2D1-AS1	LRPPRC	SPTY2D1-AS1\LRPPRC	0,945614994	0,00037478
SPTY2D1-AS1	LZIC	SPTY2D1-AS1\LZIC	0,962351501	0,000165344
SPTY2D1-AS1	MAGEA3	SPTY2D1-AS1\MAGEA3	0,845195651	0,006205908
SPTY2D1-AS1	MAML3	SPTY2D1-AS1\MAML3	0,778249502	0,017383156
SPTY2D1-AS1	MAP3K2	SPTY2D1-AS1\MAP3K2	0,945614994	0,00037478
SPTY2D1-AS1	MBLAC2	SPTY2D1-AS1\MBLAC2	0,761512935	0,021263227
SPTY2D1-AS1	MCOLN3	SPTY2D1-AS1\MCOLN3	0,828459144	0,008289241
SPTY2D1-AS1	MCTP2	SPTY2D1-AS1\MCTP2	0,719671547	0,033763226
SPTY2D1-AS1	METTL10	SPTY2D1-AS1\METTL10	0,694566727	0,043816138
SPTY2D1-AS1	MPP5	SPTY2D1-AS1\MPP5	0,903773606	0,001598325
SPTY2D1-AS1	MRPL40	SPTY2D1-AS1\MRPL40	0,778249502	0,017383156
SPTY2D1-AS1	MTPAP	SPTY2D1-AS1\MTPAP	0,79498601	0,013833774
SPTY2D1-AS1	MTPN	SPTY2D1-AS1\MTPN	0,781512618	0,016005291
SPTY2D1-AS1	MTSS1L	SPTY2D1-AS1\MTSS1L	0,803354323	0,012202381
SPTY2D1-AS1	N4BP1	SPTY2D1-AS1\N4BP1	0,70293504	0,040145501
SPTY2D1-AS1	N4BP2L2	SPTY2D1-AS1\N4BP2L2	0,719671547	0,033763226
SPTY2D1-AS1	NAA25	SPTY2D1-AS1\NAA25	0,711303294	0,037125219
SPTY2D1-AS1	NAGK	SPTY2D1-AS1\NAGK	0,719671547	0,033763226
SPTY2D1-AS1	NDRG3	SPTY2D1-AS1\NDRG3	0,711303294	0,037125219
SPTY2D1-AS1	NDUFA4	SPTY2D1-AS1\NDUFA4	0,878668785	0,003218695
SPTY2D1-AS1	NDUFB5	SPTY2D1-AS1\NDUFB5	0,853563964	0,005202822

SPTY2D1-AS1	NETO2	SPTY2D1-AS1\NETO2	0,895405293	0,002105379
SPTY2D1-AS1	NHLRC3	SPTY2D1-AS1\NHLRC3	0,79498601	0,013833774
SPTY2D1-AS1	NIPSNAP1	SPTY2D1-AS1\NIPSNAP1	0,828459144	0,008289241
SPTY2D1-AS1	NPNT	SPTY2D1-AS1\NPNT	0,778249502	0,017383156
SPTY2D1-AS1	NRCAM	SPTY2D1-AS1\NRCAM	0,853563964	0,005202822
SPTY2D1-AS1	NUDT12	SPTY2D1-AS1\NUDT12	0,89495796	0,00199515
SPTY2D1-AS1	NUDT5	SPTY2D1-AS1\NUDT5	0,878668785	0,003218695
SPTY2D1-AS1	NVL	SPTY2D1-AS1\NVL	0,744776368	0,026047178
SPTY2D1-AS1	OPA1	SPTY2D1-AS1\OPA1	0,719671547	0,033763226
SPTY2D1-AS1	PANK1	SPTY2D1-AS1\PANK1	0,811722577	0,010769401
SPTY2D1-AS1	PASK	SPTY2D1-AS1\PASK	0,744776368	0,026047178
SPTY2D1-AS1	PAX9	SPTY2D1-AS1\PAX9	0,728039861	0,031283069
SPTY2D1-AS1	PCBD2	SPTY2D1-AS1\PCBD2	0,719671547	0,033763226
SPTY2D1-AS1	PFN4	SPTY2D1-AS1\PFN4	0,761512935	0,021263227
SPTY2D1-AS1	PHB	SPTY2D1-AS1\PHB	0,736408114	0,02800926
SPTY2D1-AS1	PKP4	SPTY2D1-AS1\PKP4	0,686198473	0,046968695
SPTY2D1-AS1	PLA2G12A	SPTY2D1-AS1\PLA2G12A	0,903773606	0,001598325
SPTY2D1-AS1	PLEK2	SPTY2D1-AS1\PLEK2	0,761512935	0,021263227
SPTY2D1-AS1	PMPCB	SPTY2D1-AS1\PMPCB	0,845195651	0,006205908
SPTY2D1-AS1	PNMAL1	SPTY2D1-AS1\PNMAL1	0,82009083	0,009281305
SPTY2D1-AS1	PNMAL2	SPTY2D1-AS1\PNMAL2	0,845195651	0,006205908
SPTY2D1-AS1	PNPT1	SPTY2D1-AS1\PNPT1	0,778249502	0,017383156
SPTY2D1-AS1	POMT2	SPTY2D1-AS1\POMT2	0,753144681	0,023533951
SPTY2D1-AS1	POTEE	SPTY2D1-AS1\POTEE	0,70293504	0,040145501
SPTY2D1-AS1	POTEF	SPTY2D1-AS1\POTEF	0,711303294	0,037125219
SPTY2D1-AS1	PRKAB1	SPTY2D1-AS1\PRKAB1	0,753144681	0,023533951
SPTY2D1-AS1	PRKAR1A	SPTY2D1-AS1\PRKAR1A	0,714951873	0,035218254
SPTY2D1-AS1	PRMT5	SPTY2D1-AS1\PRMT5	0,686198473	0,046968695
SPTY2D1-AS1	PRRC1	SPTY2D1-AS1\PRRC1	0,928878427	0,000815697
SPTY2D1-AS1	PSAT1	SPTY2D1-AS1\PSAT1	0,769881189	0,018992504
SPTY2D1-AS1	PSD3	SPTY2D1-AS1\PSD3	0,694566727	0,043816138
SPTY2D1-AS1	PSMC2	SPTY2D1-AS1\PSMC2	0,803354323	0,012202381
SPTY2D1-AS1	PSMD12	SPTY2D1-AS1\PSMD12	0,920510173	0,000981041
SPTY2D1-AS1	PSMD5	SPTY2D1-AS1\PSMD5	0,815126061	0,010207231

SPTY2D1-AS1	PTPRK	SPTY2D1-AS1\PTPRK	0,853563964	0,005202822
SPTY2D1-AS1	PTPRT	SPTY2D1-AS1\PTPRT	0,761512935	0,021263227
SPTY2D1-AS1	RAB14	SPTY2D1-AS1\RAB14	0,811722577	0,010769401
SPTY2D1-AS1	RAB23	SPTY2D1-AS1\RAB23	0,895405293	0,002105379
SPTY2D1-AS1	RAB40C	SPTY2D1-AS1\RAB40C	0,769881189	0,018992504
SPTY2D1-AS1	RABGAP1	SPTY2D1-AS1\RABGAP1	0,711303294	0,037125219
SPTY2D1-AS1	RAD50	SPTY2D1-AS1\RAD50	0,945614994	0,00037478
SPTY2D1-AS1	RAI1	SPTY2D1-AS1\RAI1	0,735294104	0,027954144
SPTY2D1-AS1	RANBP17	SPTY2D1-AS1\RANBP17	0,778249502	0,017383156
SPTY2D1-AS1	RBM23	SPTY2D1-AS1\RBM23	0,828459144	0,008289241
SPTY2D1-AS1	RCC2	SPTY2D1-AS1\RCC2	0,694566727	0,043816138
SPTY2D1-AS1	RFESD	SPTY2D1-AS1\RFESD	0,82009083	0,009281305
SPTY2D1-AS1	RMDN3	SPTY2D1-AS1\RMDN3	0,777310908	0,017063493
SPTY2D1-AS1	RNF212	SPTY2D1-AS1\RNF212	0,753144681	0,023533951
SPTY2D1-AS1	RPGRIP1L	SPTY2D1-AS1\RPGRIP1L	0,836827397	0,00696649
SPTY2D1-AS1	RPS6KA6	SPTY2D1-AS1\RPS6KA6	0,70293504	0,040145501
SPTY2D1-AS1	RRAGB	SPTY2D1-AS1\RRAGB	0,93724668	0,000485009
SPTY2D1-AS1	RRM2B	SPTY2D1-AS1\RRM2B	0,778249502	0,017383156
SPTY2D1-AS1	RTF1	SPTY2D1-AS1\RTF1	0,845195651	0,006205908
SPTY2D1-AS1	S100A14	SPTY2D1-AS1\S100A14	0,728039861	0,031283069
SPTY2D1-AS1	SCAMP1	SPTY2D1-AS1\SCAMP1	0,728039861	0,031283069
SPTY2D1-AS1	SCCPDH	SPTY2D1-AS1\SCCPDH	0,845195651	0,006205908
SPTY2D1-AS1	SDR42E1	SPTY2D1-AS1\SDR42E1	0,761512935	0,021263227
SPTY2D1-AS1	SERBP1	SPTY2D1-AS1\SERBP1	0,761512935	0,021263227
SPTY2D1-AS1	SGPL1	SPTY2D1-AS1\SGPL1	0,744776368	0,026047178
SPTY2D1-AS1	SIX1	SPTY2D1-AS1\SIX1	0,711303294	0,037125219
SPTY2D1-AS1	SIX4	SPTY2D1-AS1\SIX4	0,836827397	0,00696649
SPTY2D1-AS1	SLC15A1	SPTY2D1-AS1\SLC15A1	0,735294104	0,027854938
SPTY2D1-AS1	SLC22A5	SPTY2D1-AS1\SLC22A5	0,753144681	0,023533951
SPTY2D1-AS1	SLC30A1	SPTY2D1-AS1\SLC30A1	0,70293504	0,040145501
SPTY2D1-AS1	SLC30A6	SPTY2D1-AS1\SLC30A6	0,962351501	0,000165344
SPTY2D1-AS1	SLC44A3	SPTY2D1-AS1\SLC44A3	0,711303294	0,037125219
SPTY2D1-AS1	SLC46A1	SPTY2D1-AS1\SLC46A1	0,728039861	0,031283069
SPTY2D1-AS1	SLCO5A1	SPTY2D1-AS1\SLCO5A1	0,70293504	0,040145501

SPTY2D1-AS1	SMO	SPTY2D1-AS1\SMO	0,70293504	0,040145501
SPTY2D1-AS1	SNUPN	SPTY2D1-AS1\SNUPN	0,786617756	0,015288801
SPTY2D1-AS1	SNX1	SPTY2D1-AS1\SNX1	0,778249502	0,017383156
SPTY2D1-AS1	SORBS2	SPTY2D1-AS1\SORBS2	0,694566727	0,043816138
SPTY2D1-AS1	SORCS1	SPTY2D1-AS1\SORCS1	0,82009083	0,009281305
SPTY2D1-AS1	SPATA2	SPTY2D1-AS1\SPATA2	0,711303294	0,037125219
SPTY2D1-AS1	SPIN1	SPTY2D1-AS1\SPIN1	0,836827397	0,00696649
SPTY2D1-AS1	SPIRE2	SPTY2D1-AS1\SPIRE2	0,718487382	0,033278219
SPTY2D1-AS1	SPRY3	SPTY2D1-AS1\SPRY3	0,82009083	0,009281305
SPTY2D1-AS1	ST7L	SPTY2D1-AS1\ST7L	0,719671547	0,033763226
SPTY2D1-AS1	STARD7	SPTY2D1-AS1\STARD7	0,736408114	0,02800926
SPTY2D1-AS1	STEAP2	SPTY2D1-AS1\STEAP2	0,761512935	0,021263227
SPTY2D1-AS1	STK36	SPTY2D1-AS1\STK36	0,719671547	0,033763226
SPTY2D1-AS1	STX6	SPTY2D1-AS1\STX6	0,79498601	0,013833774
SPTY2D1-AS1	SUPT3H	SPTY2D1-AS1\SUPT3H	0,823529422	0,008377425
SPTY2D1-AS1	SUSD4	SPTY2D1-AS1\SUSD4	0,711303294	0,037125219
SPTY2D1-AS1	TAB3	SPTY2D1-AS1\TAB3	0,686198473	0,046968695
SPTY2D1-AS1	TCEB3	SPTY2D1-AS1\TCEB3	0,736408114	0,02800926
SPTY2D1-AS1	THAP9	SPTY2D1-AS1\THAP9	0,870300472	0,003681658
SPTY2D1-AS1	THSD7B	SPTY2D1-AS1\THSD7B	0,753144681	0,023533951
SPTY2D1-AS1	TMEM129	SPTY2D1-AS1\TMEM129	0,69747901	0,041369047
SPTY2D1-AS1	TMEM180	SPTY2D1-AS1\TMEM180	0,731974542	0,029298943
SPTY2D1-AS1	TMEM185A	SPTY2D1-AS1\TMEM185A	0,769881189	0,018992504
SPTY2D1-AS1	TMEM26	SPTY2D1-AS1\TMEM26	0,853563964	0,005202822
SPTY2D1-AS1	TNPO1	SPTY2D1-AS1\TNPO1	0,786617756	0,015288801
SPTY2D1-AS1	TOMM34	SPTY2D1-AS1\TOMM34	0,70293504	0,040145501
SPTY2D1-AS1	TRIM52	SPTY2D1-AS1\TRIM52	0,728039861	0,031283069
SPTY2D1-AS1	TRIM61	SPTY2D1-AS1\TRIM61	0,920510173	0,000981041
SPTY2D1-AS1	TRMT10B	SPTY2D1-AS1\TRMT10B	0,870300472	0,003681658
SPTY2D1-AS1	TRMT5	SPTY2D1-AS1\TRMT5	0,828459144	0,008289241
SPTY2D1-AS1	TRPM7	SPTY2D1-AS1\TRPM7	0,744776368	0,026047178
SPTY2D1-AS1	TRUB1	SPTY2D1-AS1\TRUB1	0,920510173	0,000981041
SPTY2D1-AS1	TTC22	SPTY2D1-AS1\TTC22	0,79498601	0,013833774
SPTY2D1-AS1	TTC26	SPTY2D1-AS1\TTC26	0,811722577	0,010769401

SPTY2D1-AS1	TXNL1	SPTY2D1-AS1\TXNL1	0,82009083	0,009281305
SPTY2D1-AS1	TYW5	SPTY2D1-AS1\TYW5	0,828459144	0,008289241
SPTY2D1-AS1	UBAP2	SPTY2D1-AS1\UBAP2	0,811722577	0,010769401
SPTY2D1-AS1	UBE2V2	SPTY2D1-AS1\UBE2V2	0,815126061	0,010207231
SPTY2D1-AS1	UBE3A	SPTY2D1-AS1\UBE3A	0,870300472	0,003681658
SPTY2D1-AS1	UBE3C	SPTY2D1-AS1\UBE3C	0,736408114	0,02800926
SPTY2D1-AS1	UBFD1	SPTY2D1-AS1\UBFD1	0,895405293	0,002105379
SPTY2D1-AS1	UBTD2	SPTY2D1-AS1\UBTD2	0,865546227	0,003935185
SPTY2D1-AS1	UEVLD	SPTY2D1-AS1\UEVLD	0,885178506	0,002810847
SPTY2D1-AS1	UMPS	SPTY2D1-AS1\UMPS	0,731974542	0,029298943
SPTY2D1-AS1	UNG	SPTY2D1-AS1\UNG	0,686198473	0,046968695
SPTY2D1-AS1	URB2	SPTY2D1-AS1\URB2	0,887037039	0,002469136
SPTY2D1-AS1	VAC14	SPTY2D1-AS1\VAC14	0,79498601	0,013833774
SPTY2D1-AS1	VANGL1	SPTY2D1-AS1\VANGL1	0,811722577	0,010769401
SPTY2D1-AS1	VMA21	SPTY2D1-AS1\VMA21	0,903773606	0,001598325
SPTY2D1-AS1	VPS35	SPTY2D1-AS1\VPS35	0,769881189	0,018992504
SPTY2D1-AS1	WASL	SPTY2D1-AS1\WASL	0,761512935	0,021263227
SPTY2D1-AS1	WDR3	SPTY2D1-AS1\WDR3	0,744776368	0,026047178
SPTY2D1-AS1	WDR61	SPTY2D1-AS1\WDR61	0,69747901	0,041369047
SPTY2D1-AS1	WDR91	SPTY2D1-AS1\WDR91	0,728039861	0,031283069
SPTY2D1-AS1	WTH3DI	SPTY2D1-AS1\WTH3DI	0,79498601	0,013833774
SPTY2D1-AS1	WWOX	SPTY2D1-AS1\WWOX	0,718487382	0,033664022
SPTY2D1-AS1	XPO7	SPTY2D1-AS1\XPO7	0,979088068	6,61376E-05
SPTY2D1-AS1	XYLT2	SPTY2D1-AS1\XYLT2	0,694566727	0,043816138
SPTY2D1-AS1	YY1AP1	SPTY2D1-AS1\YY1AP1	0,728039861	0,031283069
SPTY2D1-AS1	ZBTB41	SPTY2D1-AS1\ZBTB41	0,878668785	0,003218695
SPTY2D1-AS1	ZC3HC1	SPTY2D1-AS1\ZC3HC1	0,744776368	0,026047178
SPTY2D1-AS1	ZMYM6	SPTY2D1-AS1\ZMYM6	0,853563964	0,005202822
SPTY2D1-AS1	ZNF221	SPTY2D1-AS1\ZNF221	0,694566727	0,043816138
SPTY2D1-AS1	ZNF280B	SPTY2D1-AS1\ZNF280B	0,845195651	0,006205908
SPTY2D1-AS1	ZNF449	SPTY2D1-AS1\ZNF449	0,886554599	0,002391975
SPTY2D1-AS1	ZNF543	SPTY2D1-AS1\ZNF543	0,853563964	0,005202822
SPTY2D1-AS1	ZNF544	SPTY2D1-AS1\ZNF544	0,903773606	0,001598325
SPTY2D1-AS1	ZNF554	SPTY2D1-AS1\ZNF554	0,714951873	0,035218254

SPTY2D1-AS1	ZNF558	SPTY2D1-AS1\ZNF558	0,728039861	0,031283069
SPTY2D1-AS1	ZNF562	SPTY2D1-AS1\ZNF562	0,91214186	0,001322751
SPTY2D1-AS1	ZNF572	SPTY2D1-AS1\ZNF572	0,853563964	0,005202822
SPTY2D1-AS1	ZNF626	SPTY2D1-AS1\ZNF626	0,686198473	0,046968695
SPTY2D1-AS1	ZNF782	SPTY2D1-AS1\ZNF782	0,882352948	0,002700617
SPTY2D1-AS1	ZRANB3	SPTY2D1-AS1\ZRANB3	0,828459144	0,008289241
SPTY2D1-AS1	ZSCAN31	SPTY2D1-AS1\ZSCAN31	0,836827397	0,00696649
TTC28-AS1	ABLIM2	TTC28-AS1\ABLIM2	0,69631058	0,041137565
TTC28-AS1	AJUBA	TTC28-AS1\AJUBA	0,887795985	0,002248677
TTC28-AS1	AKR1B1	TTC28-AS1\AKR1B1	0,73112613	0,028174603
TTC28-AS1	ALDH7A1	TTC28-AS1\ALDH7A1	0,748533905	0,023015874
TTC28-AS1	ALS2	TTC28-AS1\ALS2	0,844276607	0,005026455
TTC28-AS1	ANK3	TTC28-AS1\ANK3	0,705014467	0,037433863
TTC28-AS1	AP1S1	TTC28-AS1\AP1S1	0,83557272	0,006084656
TTC28-AS1	APOBEC2	TTC28-AS1\APOBEC2	0,73112613	0,028174603
TTC28-AS1	APP	TTC28-AS1\APP	0,826868832	0,007142857
TTC28-AS1	ARL1	TTC28-AS1\ARL1	0,792053282	0,012566137
TTC28-AS1	ATP6V0A1	TTC28-AS1\ATP6V0A1	0,779035687	0,01521164
TTC28-AS1	AUTS2	TTC28-AS1\AUTS2	0,87038821	0,003174603
TTC28-AS1	AZIN1	TTC28-AS1\AZIN1	0,826868832	0,007142857
TTC28-AS1	BAIAP2	TTC28-AS1\BAIAP2	0,922611535	0,000793651
TTC28-AS1	BCL11A	TTC28-AS1\BCL11A	0,757237792	0,020634921
TTC28-AS1	BRD1	TTC28-AS1\BRD1	0,879092097	0,002645503
TTC28-AS1	C14orf132	TTC28-AS1\C14orf132	0,738561869	0,024867725
TTC28-AS1	C14orf39	TTC28-AS1\C14orf39	0,887795985	0,002248677
TTC28-AS1	C19orf44	TTC28-AS1\C19orf44	0,783349395	0,014021164
TTC28-AS1	C19orf82	TTC28-AS1\C19orf82	0,739830017	0,024735449
TTC28-AS1	C1orf109	TTC28-AS1\C1orf109	0,687606692	0,044841271
TTC28-AS1	C4A	TTC28-AS1\C4A	0,887795985	0,002248677
TTC28-AS1	C4B	TTC28-AS1\C4B	0,887795985	0,002248677
TTC28-AS1	C5orf24	TTC28-AS1\C5orf24	0,852980494	0,004761905
TTC28-AS1	CACUL1	TTC28-AS1\CACUL1	0,852980494	0,004761905
TTC28-AS1	CALR	TTC28-AS1\CALR	0,69631058	0,041137565
TTC28-AS1	CBS	TTC28-AS1\CBS	0,69631058	0,041137565

TTC28-AS1	CCDC122	TTC28-AS1\CCDC122	0,748533905	0,023015874
TTC28-AS1	CCDC148	TTC28-AS1\CCDC148	0,687606692	0,044841271
TTC28-AS1	CCDC30	TTC28-AS1\CCDC30	0,861684382	0,003835979
TTC28-AS1	CCDC73	TTC28-AS1\CCDC73	0,943960726	0,000529101
TTC28-AS1	CCDC8	TTC28-AS1\CCDC8	0,809461057	0,01005291
TTC28-AS1	CDC42BPG	TTC28-AS1\CDC42BPG	0,891518474	0,002116402
TTC28-AS1	CDH2	TTC28-AS1\CDH2	0,713718355	0,033465609
TTC28-AS1	CDIP1	TTC28-AS1\CDIP1	0,705014467	0,037433863
TTC28-AS1	CDKL3	TTC28-AS1\CDKL3	0,705014467	0,037433863
TTC28-AS1	CETN3	TTC28-AS1\CETN3	0,748533905	0,023015874
TTC28-AS1	CHID1	TTC28-AS1\CHID1	0,87038821	0,003174603
TTC28-AS1	CLCA1	TTC28-AS1\CLCA1	0,886363626	0,003174603
TTC28-AS1	CLCN5	TTC28-AS1\CLCN5	0,765941679	0,018518519
TTC28-AS1	CLDN12	TTC28-AS1\CLDN12	0,713718355	0,033465609
TTC28-AS1	CLHC1	TTC28-AS1\CLHC1	0,792053282	0,012566137
TTC28-AS1	CLSTN1	TTC28-AS1\CLSTN1	0,87038821	0,003174603
TTC28-AS1	CNTNAP2	TTC28-AS1\CNTNAP2	0,809461057	0,01005291
TTC28-AS1	COG8	TTC28-AS1\COG8	0,694859982	0,04021164
TTC28-AS1	COL8A2	TTC28-AS1\COL8A2	0,809461057	0,01005291
TTC28-AS1	COX19	TTC28-AS1\COX19	0,826868832	0,007142857
TTC28-AS1	CRTC1	TTC28-AS1\CRTC1	0,90520376	0,001322751
TTC28-AS1	DCAKD	TTC28-AS1\DCAKD	0,748533905	0,023015874
TTC28-AS1	DDX31	TTC28-AS1\DDX31	0,722422242	0,03042328
TTC28-AS1	DET1	TTC28-AS1\DET1	0,861684382	0,003835979
TTC28-AS1	DHX32	TTC28-AS1\DHX32	0,722422242	0,03042328
TTC28-AS1	DNAJC19	TTC28-AS1\DNAJC19	0,861684382	0,003835979
TTC28-AS1	DNAJC21	TTC28-AS1\DNAJC21	0,922611535	0,000793651
TTC28-AS1	DNAL4	TTC28-AS1\DNAL4	0,739830017	0,024735449
TTC28-AS1	DPH6	TTC28-AS1\DPH6	0,678902805	0,048412699
TTC28-AS1	DSG2	TTC28-AS1\DSG2	0,722422242	0,03042328
TTC28-AS1	DUOXA1	TTC28-AS1\DUOXA1	0,765941679	0,018518519
TTC28-AS1	EDA2R	TTC28-AS1\EDA2R	0,887795985	0,002248677
TTC28-AS1	EFNB2	TTC28-AS1\EFNB2	0,687606692	0,044841271
TTC28-AS1	EIF2AK4	TTC28-AS1\EIF2AK4	0,678902805	0,048412699

TTC28-AS1	EMC1	TTC28-AS1\EMC1	0,705014467	0,037433863
TTC28-AS1	EMC10	TTC28-AS1\EMC10	0,891518474	0,002116402
TTC28-AS1	ENOSF1	TTC28-AS1\ENOSF1	0,748533905	0,023015874
TTC28-AS1	EPN3	TTC28-AS1\EPN3	0,765941679	0,018518519
TTC28-AS1	EPS15L1	TTC28-AS1\EPS15L1	0,818164945	0,008333334
TTC28-AS1	EVA1A	TTC28-AS1\EVA1A	0,739830017	0,024735449
TTC28-AS1	EXOC7	TTC28-AS1\EXOC7	0,717066944	0,030952381
TTC28-AS1	EXOSC10	TTC28-AS1\EXOSC10	0,713718355	0,033465609
TTC28-AS1	FAM160A1	TTC28-AS1\FAM160A1	0,792053282	0,012566137
TTC28-AS1	FAM218A	TTC28-AS1\FAM218A	0,94001931	0,000529101
TTC28-AS1	FAM83B	TTC28-AS1\FAM83B	0,748533905	0,023015874
TTC28-AS1	FBXW11	TTC28-AS1\FBXW11	0,717066944	0,030952381
TTC28-AS1	FEM1B	TTC28-AS1\FEM1B	0,826868832	0,007142857
TTC28-AS1	FNBP1L	TTC28-AS1\FNBP1L	0,748533905	0,023015874
TTC28-AS1	FRAS1	TTC28-AS1\FRAS1	0,774645507	0,015740741
TTC28-AS1	GGA1	TTC28-AS1\GGA1	0,896499872	0,001851852
TTC28-AS1	GGCT	TTC28-AS1\GGCT	0,713718355	0,033465609
TTC28-AS1	GNPDA1	TTC28-AS1\GNPDA1	0,748533905	0,023015874
TTC28-AS1	GOLM1	TTC28-AS1\GOLM1	0,774645507	0,015740741
TTC28-AS1	GPC4	TTC28-AS1\GPC4	0,844276607	0,005026455
TTC28-AS1	GPR107	TTC28-AS1\GPR107	0,717066944	0,030952381
TTC28-AS1	GPR35	TTC28-AS1\GPR35	0,703600347	0,037169311
TTC28-AS1	GRHL2	TTC28-AS1\GRHL2	0,687606692	0,044841271
TTC28-AS1	GTF2H4	TTC28-AS1\GTF2H4	0,805593729	0,011507937
TTC28-AS1	GTF2IRD2	TTC28-AS1\GTF2IRD2	0,69631058	0,041137565
TTC28-AS1	GTF3C4	TTC28-AS1\GTF3C4	0,852980494	0,004761905
TTC28-AS1	GXYLT1	TTC28-AS1\GXYLT1	0,83557272	0,006084656
TTC28-AS1	HDGFRP3	TTC28-AS1\HDGFRP3	0,792053282	0,012566137
TTC28-AS1	HN1L	TTC28-AS1\HN1L	0,83557272	0,006084656
TTC28-AS1	HOMER3	TTC28-AS1\HOMER3	0,809461057	0,01005291
TTC28-AS1	HPS5	TTC28-AS1\HPS5	0,922611535	0,000793651
TTC28-AS1	HPSE	TTC28-AS1\HPSE	0,69631058	0,041137565
TTC28-AS1	IDH3G	TTC28-AS1\IDH3G	0,687606692	0,044841271
TTC28-AS1	IGF1R	TTC28-AS1\IGF1R	0,809461057	0,01005291

TTC28-AS1	IGSF3	TTC28-AS1\IGSF3	0,809461057	0,01005291
TTC28-AS1	IKBIP	TTC28-AS1\IKBIP	0,818164945	0,008333334
TTC28-AS1	ITGAV	TTC28-AS1\ITGAV	0,783349395	0,014021164
TTC28-AS1	KAL1	TTC28-AS1\KAL1	0,957427084	0,000132275
TTC28-AS1	KCTD1	TTC28-AS1\KCTD1	0,809461057	0,01005291
TTC28-AS1	KDM4B	TTC28-AS1\KDM4B	0,73112613	0,028174603
TTC28-AS1	KIAA0319L	TTC28-AS1\KIAA0319L	0,818164945	0,008333334
TTC28-AS1	KRTAP5-8	TTC28-AS1\KRTAP5-8	0,87038821	0,003174603
TTC28-AS1	KTN1	TTC28-AS1\KTN1	0,678902805	0,048412699
TTC28-AS1	LAMP1	TTC28-AS1\LAMP1	0,769153178	0,016798941
TTC28-AS1	LAMP5	TTC28-AS1\LAMP5	0,887795985	0,002248677
TTC28-AS1	LARS	TTC28-AS1\LARS	0,722422242	0,03042328
TTC28-AS1	LEPREL1	TTC28-AS1\LEPREL1	0,765941679	0,018518519
TTC28-AS1	LPHN3	TTC28-AS1\LPHN3	0,90520376	0,001322751
TTC28-AS1	LPIN1	TTC28-AS1\LPIN1	0,705014467	0,037433863
TTC28-AS1	LRIG3	TTC28-AS1\LRIG3	0,73112613	0,028174603
TTC28-AS1	LRPAP1	TTC28-AS1\LRPAP1	0,812855065	0,008465609
TTC28-AS1	LRPPRC	TTC28-AS1\LRPPRC	0,713718355	0,033465609
TTC28-AS1	LRRC27	TTC28-AS1\LRRC27	0,739830017	0,024735449
TTC28-AS1	LRRC41	TTC28-AS1\LRRC41	0,722422242	0,03042328
TTC28-AS1	LRRK1	TTC28-AS1\LRRK1	0,809461057	0,01005291
TTC28-AS1	LZIC	TTC28-AS1\LZIC	0,783349395	0,014021164
TTC28-AS1	MAGEA3	TTC28-AS1\MAGEA3	0,713718355	0,033465609
TTC28-AS1	MAST4	TTC28-AS1\MAST4	0,721081138	0,03042328
TTC28-AS1	MBLAC2	TTC28-AS1\MBLAC2	0,739830017	0,024735449
TTC28-AS1	MCTP2	TTC28-AS1\MCTP2	0,765941679	0,018518519
TTC28-AS1	MED22	TTC28-AS1\MED22	0,717066944	0,032010581
TTC28-AS1	METTL10	TTC28-AS1\METTL10	0,83557272	0,006084656
TTC28-AS1	MLX	TTC28-AS1\MLX	0,712340772	0,034523811
TTC28-AS1	MOV10	TTC28-AS1\MOV10	0,705014467	0,037433863
TTC28-AS1	MST1L	TTC28-AS1\MST1L	0,809461057	0,01005291
TTC28-AS1	MTPAP	TTC28-AS1\MTPAP	0,826868832	0,007142857
TTC28-AS1	MYLK4	TTC28-AS1\MYLK4	0,94001931	0,000529101
TTC28-AS1	MYO10	TTC28-AS1\MYO10	0,705014467	0,037433863

TTC28-AS1	MYO5B	TTC28-AS1\MYO5B	0,795374334	0,011111111
TTC28-AS1	N4BP1	TTC28-AS1\N4BP1	0,722422242	0,03042328
TTC28-AS1	N4BP2L2	TTC28-AS1\N4BP2L2	0,83557272	0,006084656
TTC28-AS1	NAA25	TTC28-AS1\NAA25	0,879092097	0,002645503
TTC28-AS1	NDRG3	TTC28-AS1\NDRG3	0,90520376	0,001322751
TTC28-AS1	NDUFA4	TTC28-AS1\NDUFA4	0,687606692	0,044841271
TTC28-AS1	NEO1	TTC28-AS1\NEO1	0,792053282	0,012566137
TTC28-AS1	NET1	TTC28-AS1\NET1	0,73112613	0,028174603
TTC28-AS1	NETO2	TTC28-AS1\NETO2	0,687606692	0,044841271
TTC28-AS1	NHLRC3	TTC28-AS1\NHLRC3	0,887795985	0,002248677
TTC28-AS1	NPNT	TTC28-AS1\NPNT	0,90520376	0,001322751
TTC28-AS1	NRCAM	TTC28-AS1\NRCAM	0,678902805	0,048412699
TTC28-AS1	NSRP1	TTC28-AS1\NSRP1	0,852980494	0,004761905
TTC28-AS1	NTF4	TTC28-AS1\NTF4	0,73112613	0,028174603
TTC28-AS1	NUDT9	TTC28-AS1\NUDT9	0,861684382	0,003835979
TTC28-AS1	NXN	TTC28-AS1\NXN	0,690489829	0,040079366
TTC28-AS1	OAT	TTC28-AS1\OAT	0,678902805	0,048412699
TTC28-AS1	OSBPL3	TTC28-AS1\OSBPL3	0,739830017	0,024735449
TTC28-AS1	PARD6B	TTC28-AS1\PARD6B	0,687606692	0,044841271
TTC28-AS1	PAX1	TTC28-AS1\PAX1	0,765941679	0,018518519
TTC28-AS1	PAX9	TTC28-AS1\PAX9	0,705014467	0,037433863
TTC28-AS1	PCSK6	TTC28-AS1\PCSK6	0,783349395	0,014021164
TTC28-AS1	PDGFA	TTC28-AS1\PDGFA	0,887795985	0,002248677
TTC28-AS1	PDPK1	TTC28-AS1\PDPK1	0,774645507	0,015740741
TTC28-AS1	PGAP1	TTC28-AS1\PGAP1	0,705014467	0,037433863
TTC28-AS1	PIAS2	TTC28-AS1\PIAS2	0,765941679	0,018518519
TTC28-AS1	PIFO	TTC28-AS1\PIFO	0,705014467	0,037433863
TTC28-AS1	PLA2G12A	TTC28-AS1\PLA2G12A	0,739830017	0,024735449
TTC28-AS1	PMPCB	TTC28-AS1\PMPCB	0,713718355	0,033465609
TTC28-AS1	PNMAL1	TTC28-AS1\PNMAL1	0,765941679	0,018518519
TTC28-AS1	POGZ	TTC28-AS1\POGZ	0,94001931	0,000529101
TTC28-AS1	POMT2	TTC28-AS1\POMT2	0,887795985	0,002248677
TTC28-AS1	POTEE	TTC28-AS1\POTEE	0,87038821	0,003174603
TTC28-AS1	POTEF	TTC28-AS1\POTEF	0,87038821	0,003174603

TTC28-AS1	POTEH	TTC28-AS1\POTEH	0,687606692	0,044841271
TTC28-AS1	POTEM	TTC28-AS1\POTEM	0,826868832	0,007142857
TTC28-AS1	PRKAA2	TTC28-AS1\PRKAA2	0,748533905	0,023015874
TTC28-AS1	PRKAB1	TTC28-AS1\PRKAB1	0,887795985	0,002248677
TTC28-AS1	PRKAB2	TTC28-AS1\PRKAB2	0,678902805	0,048412699
TTC28-AS1	PRKAR1A	TTC28-AS1\PRKAR1A	0,717066944	0,030952381
TTC28-AS1	PROP1	TTC28-AS1\PROP1	0,765941679	0,018518519
TTC28-AS1	PRRC1	TTC28-AS1\PRRC1	0,73112613	0,028174603
TTC28-AS1	PRRG4	TTC28-AS1\PRRG4	0,809461057	0,01005291
TTC28-AS1	PSAT1	TTC28-AS1\PSAT1	0,94001931	0,000529101
TTC28-AS1	PSD3	TTC28-AS1\PSD3	0,748533905	0,023015874
TTC28-AS1	PTPRK	TTC28-AS1\PTPRK	0,792053282	0,012566137
TTC28-AS1	QSER1	TTC28-AS1\QSER1	0,80075717	0,011243386
TTC28-AS1	RAB14	TTC28-AS1\RAB14	0,69631058	0,041137565
TTC28-AS1	RAB23	TTC28-AS1\RAB23	0,852980494	0,004761905
TTC28-AS1	RAB40C	TTC28-AS1\RAB40C	0,757237792	0,020634921
TTC28-AS1	RABGAP1	TTC28-AS1\RABGAP1	0,887795985	0,002248677
TTC28-AS1	RAI1	TTC28-AS1\RAI1	0,756042659	0,02010582
TTC28-AS1	RANBP17	TTC28-AS1\RANBP17	0,844276607	0,005026455
TTC28-AS1	RASEF	TTC28-AS1\RASEF	0,792053282	0,012566137
TTC28-AS1	RAVER2	TTC28-AS1\RAVER2	0,83557272	0,006084656
TTC28-AS1	RGS21	TTC28-AS1\RGS21	0,849857092	0,00489418
TTC28-AS1	RGS9	TTC28-AS1\RGS9	0,879092097	0,002645503
TTC28-AS1	RHBDD2	TTC28-AS1\RHBDD2	0,783349395	0,014021164
TTC28-AS1	RMDN3	TTC28-AS1\RMDN3	0,725451291	0,029629629
TTC28-AS1	RNF144B	TTC28-AS1\RNF144B	0,80075717	0,011243386
TTC28-AS1	RNF212	TTC28-AS1\RNF212	0,90520376	0,001322751
TTC28-AS1	RNF32	TTC28-AS1\RNF32	0,69631058	0,041137565
TTC28-AS1	ROR2	TTC28-AS1\ROR2	0,705014467	0,037433863
TTC28-AS1	RP9	TTC28-AS1\RP9	0,722422242	0,03042328
TTC28-AS1	RPGRIP1L	TTC28-AS1\RPGRIP1L	0,80075717	0,011243386
TTC28-AS1	RPL37	TTC28-AS1\RPL37	0,87038821	0,003174603
TTC28-AS1	RPS6KA6	TTC28-AS1\RPS6KA6	0,765941679	0,018518519
TTC28-AS1	RRAGB	TTC28-AS1\RRAGB	0,687606692	0,044841271

TTC28-AS1	RRM2B	TTC28-AS1\RRM2B	0,678902805	0,048412699
TTC28-AS1	RUNX2	TTC28-AS1\RUNX2	0,69631058	0,041137565
TTC28-AS1	SARS2	TTC28-AS1\SARS2	0,786633968	0,013624338
TTC28-AS1	SCAMP1	TTC28-AS1\SCAMP1	0,94001931	0,000529101
TTC28-AS1	SDR42E1	TTC28-AS1\SDR42E1	0,765941679	0,018518519
TTC28-AS1	SEC61A1	TTC28-AS1\SEC61A1	0,757237792	0,020634921
TTC28-AS1	SERPINB5	TTC28-AS1\SERPINB5	0,783349395	0,014021164
TTC28-AS1	SGPL1	TTC28-AS1\SGPL1	0,783349395	0,014021164
TTC28-AS1	SIX1	TTC28-AS1\SIX1	0,809461057	0,01005291
TTC28-AS1	SIX4	TTC28-AS1\SIX4	0,90520376	0,001322751
TTC28-AS1	SLC15A1	TTC28-AS1\SLC15A1	0,852186799	0,004497354
TTC28-AS1	SLC18B1	TTC28-AS1\SLC18B1	0,739830017	0,024735449
TTC28-AS1	SLC22A23	TTC28-AS1\SLC22A23	0,705014467	0,037433863
TTC28-AS1	SLC30A6	TTC28-AS1\SLC30A6	0,705014467	0,037433863
TTC28-AS1	SLC44A5	TTC28-AS1\SLC44A5	0,774645507	0,015740741
TTC28-AS1	SLC46A1	TTC28-AS1\SLC46A1	0,722422242	0,03042328
TTC28-AS1	SLCO5A1	TTC28-AS1\SLCO5A1	0,792053282	0,012566137
TTC28-AS1	SMO	TTC28-AS1\SMO	0,765941679	0,018518519
TTC28-AS1	SOGA2	TTC28-AS1\SOGA2	0,69631058	0,041137565
TTC28-AS1	SORCS1	TTC28-AS1\SORCS1	0,713718355	0,033465609
TTC28-AS1	SPIN1	TTC28-AS1\SPIN1	0,957427084	0,000132275
TTC28-AS1	SPIN4	TTC28-AS1\SPIN4	0,879092097	0,002645503
TTC28-AS1	ST7L	TTC28-AS1\ST7L	0,678902805	0,048412699
TTC28-AS1	STARD7	TTC28-AS1\STARD7	0,765941679	0,018518519
TTC28-AS1	STK36	TTC28-AS1\STK36	0,792053282	0,012566137
TTC28-AS1	STON2	TTC28-AS1\STON2	0,748533905	0,023015874
TTC28-AS1	SUFU	TTC28-AS1\SUFU	0,678902805	0,048412699
TTC28-AS1	SUPT3H	TTC28-AS1\SUPT3H	0,847816586	0,004761905
TTC28-AS1	SYT1	TTC28-AS1\SYT1	0,705014467	0,037433863
TTC28-AS1	TAB3	TTC28-AS1\TAB3	0,922611535	0,000793651
TTC28-AS1	TCF24	TTC28-AS1\TCF24	0,705014467	0,037433863
TTC28-AS1	TFCP2L1	TTC28-AS1\TFCP2L1	0,678902805	0,048412699
TTC28-AS1	TM7SF3	TTC28-AS1\TM7SF3	0,739830017	0,024735449
TTC28-AS1	TMEM107	TTC28-AS1\TMEM107	0,757237792	0,020634921

TTC28-AS1	TMEM133	TTC28-AS1\TMEM133	0,887795985	0,002248677
TTC28-AS1	TMEM180	TTC28-AS1\TMEM180	0,841004431	0,005952381
TTC28-AS1	TMEM185A	TTC28-AS1\TMEM185A	0,783349395	0,014021164
TTC28-AS1	TMEM26	TTC28-AS1\TMEM26	0,765941679	0,018518519
TTC28-AS1	TMEM30B	TTC28-AS1\TMEM30B	0,792053282	0,012566137
TTC28-AS1	TNPO1	TTC28-AS1\TNPO1	0,774645507	0,015740741
TTC28-AS1	TOMM20	TTC28-AS1\TOMM20	0,826868832	0,007142857
TTC28-AS1	TOMM34	TTC28-AS1\TOMM34	0,852980494	0,004761905
TTC28-AS1	TOMM40	TTC28-AS1\TOMM40	0,748533905	0,023015874
TTC28-AS1	TRMT10B	TTC28-AS1\TRMT10B	0,792053282	0,012566137
TTC28-AS1	TRMT5	TTC28-AS1\TRMT5	0,861684382	0,003835979
TTC28-AS1	TTC26	TTC28-AS1\TTC26	0,80075717	0,011243386
TTC28-AS1	TTYH2	TTC28-AS1\TTYH2	0,879092097	0,002645503
TTC28-AS1	TXNL1	TTC28-AS1\TXNL1	0,748533905	0,023015874
TTC28-AS1	TYW5	TTC28-AS1\TYW5	0,774645507	0,015740741
TTC28-AS1	UBTD2	TTC28-AS1\UBTD2	0,738561869	0,024867725
TTC28-AS1	UEVLD	TTC28-AS1\UEVLD	0,876415133	0,003042328
TTC28-AS1	UMPS	TTC28-AS1\UMPS	0,81444639	0,00978836
TTC28-AS1	UNC5B	TTC28-AS1\UNC5B	0,705014467	0,037433863
TTC28-AS1	UPF1	TTC28-AS1\UPF1	0,678902805	0,048412699
TTC28-AS1	USP46	TTC28-AS1\USP46	0,705014467	0,037433863
TTC28-AS1	VPS35	TTC28-AS1\VPS35	0,818164945	0,008333334
TTC28-AS1	VWCE	TTC28-AS1\VWCE	0,922611535	0,000793651
TTC28-AS1	WASL	TTC28-AS1\WASL	0,90520376	0,001322751
TTC28-AS1	WDR35	TTC28-AS1\WDR35	0,792053282	0,012566137
TTC28-AS1	XPO7	TTC28-AS1\XPO7	0,765941679	0,018518519
TTC28-AS1	XPR1	TTC28-AS1\XPR1	0,73112613	0,028174603
TTC28-AS1	ZBTB41	TTC28-AS1\ZBTB41	0,73112613	0,028174603
TTC28-AS1	ZNF211	TTC28-AS1\ZNF211	0,887795985	0,002248677
TTC28-AS1	ZNF221	TTC28-AS1\ZNF221	0,887795985	0,002248677
TTC28-AS1	ZNF229	TTC28-AS1\ZNF229	0,765941679	0,018518519
TTC28-AS1	ZNF257	TTC28-AS1\ZNF257	0,818164945	0,008333334
TTC28-AS1	ZNF280B	TTC28-AS1\ZNF280B	0,678902805	0,048412699
TTC28-AS1	ZNF449	TTC28-AS1\ZNF449	0,721081138	0,03042328

TTC28-AS1	ZNF540	TTC28-AS1\ZNF540	0,896499872	0,001851852
TTC28-AS1	ZNF543	TTC28-AS1\ZNF543	0,748533905	0,023015874
TTC28-AS1	ZNF544	TTC28-AS1\ZNF544	0,722422242	0,03042328
TTC28-AS1	ZNF562	TTC28-AS1\ZNF562	0,792053282	0,012566137
TTC28-AS1	ZNF572	TTC28-AS1\ZNF572	0,774645507	0,015740741
TTC28-AS1	ZNF605	TTC28-AS1\ZNF605	0,765941679	0,018518519
TTC28-AS1	ZRANB3	TTC28-AS1\ZRANB3	0,687606692	0,044841271
TTC28-AS1	ZSCAN31	TTC28-AS1\ZSCAN31	0,80075717	0,011243386
WAC-AS1	ACAD10	WAC-AS1\ACAD10	0,728039861	0,031481482
WAC-AS1	AFG3L2	WAC-AS1\AFG3L2	0,816666603	0,010769401
WAC-AS1	ALS2	WAC-AS1\ALS2	0,699999928	0,043253969
WAC-AS1	ANKFY1	WAC-AS1\ANKFY1	0,73333329	0,031123236
WAC-AS1	AP2B1	WAC-AS1\AP2B1	0,74999994	0,025490521
WAC-AS1	ARL6IP1	WAC-AS1\ARL6IP1	0,73333329	0,031123236
WAC-AS1	ARV1	WAC-AS1\ARV1	0,833333313	0,008267196
WAC-AS1	ATP2C1	WAC-AS1\ATP2C1	0,716666639	0,036866181
WAC-AS1	ATP6V1C1	WAC-AS1\ATP6V1C1	0,716666639	0,036866181
WAC-AS1	ATPIF1	WAC-AS1\ATPIF1	0,74999994	0,025490521
WAC-AS1	BBS4	WAC-AS1\BBS4	0,73333329	0,031123236
WAC-AS1	BTBD3	WAC-AS1\BTBD3	0,783333302	0,017223325
WAC-AS1	C1orf109	WAC-AS1\C1orf109	0,799999952	0,013828263
WAC-AS1	C5orf15	WAC-AS1\C5orf15	0,73333329	0,031123236
WAC-AS1	CBS	WAC-AS1\CBS	0,833333313	0,008267196
WAC-AS1	CCDC30	WAC-AS1\CCDC30	0,870300472	0,003681658
WAC-AS1	CCZ1	WAC-AS1\CCZ1	0,866666615	0,004508378
WAC-AS1	CD274	WAC-AS1\CD274	0,866666615	0,004508378
WAC-AS1	CLDN12	WAC-AS1\CLDN12	0,849999964	0,006073633
WAC-AS1	CLINT1	WAC-AS1\CLINT1	0,766666651	0,021389991
WAC-AS1	CLTC	WAC-AS1\CLTC	0,833333313	0,008267196
WAC-AS1	COG5	WAC-AS1\COG5	0,716666639	0,036866181
WAC-AS1	COG8	WAC-AS1\COG8	0,870300472	0,003681658
WAC-AS1	CSRP2BP	WAC-AS1\CSRP2BP	0,73333329	0,031123236
WAC-AS1	CUX1	WAC-AS1\CUX1	0,783333302	0,017223325
WAC-AS1	CYB5B	WAC-AS1\CYB5B	0,783333302	0,017223325

WAC-AS1	CYP26B1	WAC-AS1\CYP26B1	0,699999928	0,043253969
WAC-AS1	DHTKD1	WAC-AS1\DHTKD1	0,799999952	0,013828263
WAC-AS1	DNAI1	WAC-AS1\DNAI1	0,916666567	0,001311728
WAC-AS1	EIF2AK1	WAC-AS1\EIF2AK1	0,699999928	0,043253969
WAC-AS1	EIF3B	WAC-AS1\EIF3B	0,816666603	0,010769401
WAC-AS1	ELOVL6	WAC-AS1\ELOVL6	0,799999952	0,013828263
WAC-AS1	ENPEP	WAC-AS1\ENPEP	0,783333302	0,017223325
WAC-AS1	ERAP1	WAC-AS1\ERAP1	0,716666639	0,036866181
WAC-AS1	EXOSC10	WAC-AS1\EXOSC10	0,716666639	0,036866181
WAC-AS1	FAM120A	WAC-AS1\FAM120A	0,699999928	0,043253969
WAC-AS1	FAM199X	WAC-AS1\FAM199X	0,716666639	0,036866181
WAC-AS1	FAM45A	WAC-AS1\FAM45A	0,783333302	0,017223325
WAC-AS1	FBXO22	WAC-AS1\FBXO22	0,711303294	0,037125219
WAC-AS1	FKTN	WAC-AS1\FKTN	0,74999994	0,025490521
WAC-AS1	FLVCR1	WAC-AS1\FLVCR1	0,883333266	0,003075397
WAC-AS1	FSTL4	WAC-AS1\FSTL4	0,799999952	0,013828263
WAC-AS1	GEMIN5	WAC-AS1\GEMIN5	0,916666567	0,001311728
WAC-AS1	GGCT	WAC-AS1\GGCT	0,799999952	0,013828263
WAC-AS1	GINS3	WAC-AS1\GINS3	0,883333266	0,003075397
WAC-AS1	GNPDA2	WAC-AS1\GNPDA2	0,73333329	0,031123236
WAC-AS1	GPHN	WAC-AS1\GPHN	0,716666639	0,036866181
WAC-AS1	GPS1	WAC-AS1\GPS1	0,799999952	0,013828263
WAC-AS1	GTF2H3	WAC-AS1\GTF2H3	0,799999952	0,013828263
WAC-AS1	GTF2H4	WAC-AS1\GTF2H4	0,695015132	0,044808201
WAC-AS1	HDAC4	WAC-AS1\HDAC4	0,711303294	0,037125219
WAC-AS1	HOMER2	WAC-AS1\HOMER2	0,799999952	0,013828263
WAC-AS1	HPSE	WAC-AS1\HPSE	0,783333302	0,017223325
WAC-AS1	IFI44	WAC-AS1\IFI44	0,74999994	0,025490521
WAC-AS1	IKZF5	WAC-AS1\IKZF5	0,699999928	0,043253969
WAC-AS1	IQCH	WAC-AS1\IQCH	0,73333329	0,031123236
WAC-AS1	KIAA1549	WAC-AS1\KIAA1549	0,716666639	0,036866181
WAC-AS1	KIF3A	WAC-AS1\KIF3A	0,799999952	0,013828263
WAC-AS1	KLHDC10	WAC-AS1\KLHDC10	0,833333313	0,008267196
WAC-AS1	KTN1	WAC-AS1\KTN1	0,816666603	0,010769401

WAC-AS1	LARS	WAC-AS1\LARS	0,73333329	0,031123236
WAC-AS1	LGI3	WAC-AS1\LGI3	0,699999928	0,043253969
WAC-AS1	LOC285556	WAC-AS1\LOC285556	0,916666567	0,001311728
WAC-AS1	LRPPRC	WAC-AS1\LRPPRC	0,799999952	0,013828263
WAC-AS1	LZIC	WAC-AS1\LZIC	0,74999994	0,025490521
WAC-AS1	MAP10	WAC-AS1\MAP10	0,716666639	0,036866181
WAC-AS1	MAP3K2	WAC-AS1\MAP3K2	0,74999994	0,025490521
WAC-AS1	MCOLN3	WAC-AS1\MCOLN3	0,73333329	0,031123236
WAC-AS1	MLEC	WAC-AS1\MLEC	0,816666603	0,010769401
WAC-AS1	MPP5	WAC-AS1\MPP5	0,783333302	0,017223325
WAC-AS1	MTPAP	WAC-AS1\MTPAP	0,699999928	0,043253969
WAC-AS1	MTPN	WAC-AS1\MTPN	0,686198473	0,046968695
WAC-AS1	MTSS1L	WAC-AS1\MTSS1L	0,766666651	0,021389991
WAC-AS1	NDUFB5	WAC-AS1\NDUFB5	0,699999928	0,043253969
WAC-AS1	NIF3L1	WAC-AS1\NIF3L1	0,716666639	0,036866181
WAC-AS1	NPTX1	WAC-AS1\NPTX1	0,766666651	0,021389991
WAC-AS1	NUDT5	WAC-AS1\NUDT5	0,766666651	0,021389991
WAC-AS1	NVL	WAC-AS1\NVL	0,933333278	0,000749559
WAC-AS1	OPA1	WAC-AS1\OPA1	0,73333329	0,031123236
WAC-AS1	PANK1	WAC-AS1\PANK1	0,716666639	0,036866181
WAC-AS1	PASK	WAC-AS1\PASK	0,74999994	0,025490521
WAC-AS1	PCBD2	WAC-AS1\PCBD2	0,899999917	0,002028219
WAC-AS1	PCCB	WAC-AS1\PCCB	0,816666603	0,010769401
WAC-AS1	PFN4	WAC-AS1\PFN4	0,916666567	0,001311728
WAC-AS1	PLA2G12A	WAC-AS1\PLA2G12A	0,699999928	0,043253969
WAC-AS1	PLEK2	WAC-AS1\PLEK2	0,766666651	0,021389991
WAC-AS1	PNMAL2	WAC-AS1\PNMAL2	0,849999964	0,006073633
WAC-AS1	PNPT1	WAC-AS1\PNPT1	0,883333266	0,003075397
WAC-AS1	POLR1A	WAC-AS1\POLR1A	0,899999917	0,002028219
WAC-AS1	PPIP5K1	WAC-AS1\PPIP5K1	0,799999952	0,013828263
WAC-AS1	PPP5C	WAC-AS1\PPP5C	0,74999994	0,025490521
WAC-AS1	PRMT5	WAC-AS1\PRMT5	0,883333266	0,003075397
WAC-AS1	PSMC2	WAC-AS1\PSMC2	0,783333302	0,017223325
WAC-AS1	PSMD12	WAC-AS1\PSMD12	0,73333329	0,031123236

WAC-AS1	PTPRT	WAC-AS1\PTPRT	0,74999994	0,025490521
WAC-AS1	RAD50	WAC-AS1\RAD50	0,74999994	0,025490521
WAC-AS1	RAI1	WAC-AS1\RAI1	0,786617756	0,015288801
WAC-AS1	RBM23	WAC-AS1\RBM23	0,849999964	0,006073633
WAC-AS1	RFESD	WAC-AS1\RFESD	0,799999952	0,013828263
WAC-AS1	RPGRIP1L	WAC-AS1\RPGRIP1L	0,716666639	0,036866181
WAC-AS1	RRAGD	WAC-AS1\RRAGD	0,73333329	0,031123236
WAC-AS1	RTF1	WAC-AS1\RTF1	0,799999952	0,013828263
WAC-AS1	SCCPDH	WAC-AS1\SCCPDH	0,866666615	0,004508378
WAC-AS1	SERBP1	WAC-AS1\SERBP1	0,866666615	0,004508378
WAC-AS1	SFXN5	WAC-AS1\SFXN5	0,686198473	0,046968695
WAC-AS1	SLC22A5	WAC-AS1\SLC22A5	0,73333329	0,031123236
WAC-AS1	SLC30A6	WAC-AS1\SLC30A6	0,816666603	0,010769401
WAC-AS1	SNUPN	WAC-AS1\SNUPN	0,816666603	0,010769401
WAC-AS1	SNX1	WAC-AS1\SNX1	0,833333313	0,008267196
WAC-AS1	SORCS1	WAC-AS1\SORCS1	0,73333329	0,031123236
WAC-AS1	SPATA2	WAC-AS1\SPATA2	0,949999988	0,000352734
WAC-AS1	ST7L	WAC-AS1\ST7L	0,816666603	0,010769401
WAC-AS1	STX6	WAC-AS1\STX6	0,783333302	0,017223325
WAC-AS1	SUSD4	WAC-AS1\SUSD4	0,849999964	0,006073633
WAC-AS1	TCEB3	WAC-AS1\TCEB3	0,866666615	0,004508378
WAC-AS1	TDGF1	WAC-AS1\TDGF1	0,716666639	0,036866181
WAC-AS1	THAP9	WAC-AS1\THAP9	0,74999994	0,025490521
WAC-AS1	THSD7B	WAC-AS1\THSD7B	0,933333278	0,000749559
WAC-AS1	TMEM129	WAC-AS1\TMEM129	0,853563964	0,005202822
WAC-AS1	TMEM26	WAC-AS1\TMEM26	0,74999994	0,025490521
WAC-AS1	TRIM52	WAC-AS1\TRIM52	0,74999994	0,025490521
WAC-AS1	TRIM61	WAC-AS1\TRIM61	0,716666639	0,036866181
WAC-AS1	TRMT10B	WAC-AS1\TRMT10B	0,73333329	0,031123236
WAC-AS1	TRUB1	WAC-AS1\TRUB1	0,73333329	0,031123236
WAC-AS1	TTC26	WAC-AS1\TTC26	0,766666651	0,021389991
WAC-AS1	TYW5	WAC-AS1\TYW5	0,833333313	0,008267196
WAC-AS1	UBE2V2	WAC-AS1\UBE2V2	0,786617756	0,015608465
WAC-AS1	UBE3A	WAC-AS1\UBE3A	0,816666603	0,010769401

WAC-AS1	UBE3C	WAC-AS1\UBE3C	0,73333329	0,031123236
WAC-AS1	UBFD1	WAC-AS1\UBFD1	0,783333302	0,017223325
WAC-AS1	URB2	WAC-AS1\URB2	0,766666651	0,021389991
WAC-AS1	WDR3	WAC-AS1\WDR3	0,916666567	0,001311728
WAC-AS1	WDR91	WAC-AS1\WDR91	0,783333302	0,017223325
WAC-AS1	WTH3DI	WAC-AS1\WTH3DI	0,816666603	0,010769401
WAC-AS1	WWOX	WAC-AS1\WWOX	0,845195651	0,006205908
WAC-AS1	XPO7	WAC-AS1\XPO7	0,766666651	0,021389991
WAC-AS1	XYLT2	WAC-AS1\XYLT2	0,833333313	0,008267196
WAC-AS1	ZC3HC1	WAC-AS1\ZC3HC1	0,866666615	0,004508378
WAC-AS1	ZMYM3	WAC-AS1\ZMYM3	0,73333329	0,031123236
WAC-AS1	ZMYM6	WAC-AS1\ZMYM6	0,799999952	0,013828263
WAC-AS1	ZNF543	WAC-AS1\ZNF543	0,73333329	0,031123236
WAC-AS1	ZNF544	WAC-AS1\ZNF544	0,699999928	0,043253969
WAC-AS1	ZNF554	WAC-AS1\ZNF554	0,84757942	0,00598545
WAC-AS1	ZNF558	WAC-AS1\ZNF558	0,849999964	0,006073633
WAC-AS1	ZNF572	WAC-AS1\ZNF572	0,799999952	0,013828263
WAC-AS1	ZNF782	WAC-AS1\ZNF782	0,79498601	0,013833774
WAC-AS1	ZRANB3	WAC-AS1\ZRANB3	0,849999964	0,006073633
WAC-AS1	ZSCAN31	WAC-AS1\ZSCAN31	0,716666639	0,036866181
