# Supplementary material for: LINC00174 is a novel prognostic factor in thymic epithelial tumors involved in cell migration and lipid metabolism
Source: Cell Death Dis. 2020 Nov 7;11(11):959. doi: 10.1038/s41419-020-03171-9 (PMC7648846; doi:10.1038/s41419-020-03171-9)
Supplement: Supplementary file 15 — Supplementary Table 3_Sheet 1 [file 41419_2020_3171_MOESM15_ESM.pdf]

List of 393 genes and 12 lncRNAs positively correlated in IRE cohort and validated in TCGA cohort (all of them are predicted to be target of miR145-5p by miRwalk 2.0 tool)

lncRNAs	lncRNAs ID	mRNAs	lncRNA\gene	R Spearman	pval
C17orf102	400591	ELOVL6	C17orf102\ELOVL6	0,187349229	0,041323513
C17orf102	400591	SHROOM2	C17orf102\SHROOM2	0,202008834	0,027583337
CDKN2B-AS1	100048912	NIF3L1	CDKN2B-AS1\NIF3L1	0,283998006	0,001814157
CDKN2B-AS1	100048912	PLCXD1	CDKN2B-AS1\PLCXD1	0,435080473	9,94233E-07
CDKN2B-AS1	100048912	RCC2	CDKN2B-AS1\RCC2	0,282502493	0,001921413
CDKN2B-AS1	100048912	UNG	CDKN2B-AS1\UNG	0,235507762	0,010071077
CDKN2B-AS1	100048912	YY1AP1	CDKN2B-AS1\YY1AP1	0,200263495	0,029137406
CTBP1-AS2	92070	ARV1	CTBP1-AS2\ARV1	0,208139866	0,023276743
CTBP1-AS2	92070	C15orf41	CTBP1-AS2\C15orf41	0,268793619	0,003208359
CTBP1-AS2	92070	COG5	CTBP1-AS2\COG5	0,503774391	8,34822E-09
CTBP1-AS2	92070	COG8	CTBP1-AS2\COG8	0,507605754	6,13749E-09
CTBP1-AS2	92070	CROT	CTBP1-AS2\CROT	0,482609315	4,12875E-08
CTBP1-AS2	92070	GPHN	CTBP1-AS2\GPHN	0,281270474	0,002014066
CTBP1-AS2	92070	PLD2	CTBP1-AS2\PLD2	0,287188435	0,001603325
CTBP1-AS2	92070	RNF170	CTBP1-AS2\RNF170	0,274163225	0,0026324
CTBP1-AS2	92070	SGPP2	CTBP1-AS2\SGPP2	0,340571144	0,000164465
CTBP1-AS2	92070	SIX1	CTBP1-AS2\SIX1	0,320217918	0,000411053
CTBP1-AS2	92070	SYBU	CTBP1-AS2\SYBU	0,304173195	0,000811472
CTBP1-AS2	92070	ZNF132	CTBP1-AS2\ZNF132	0,34575559	0,00012897
CTBP1-AS2	92070	GLI2	CTBP1-AS2\GLI2	0,283150548	0,001874247
CTBP1-AS2	92070	GRHL2	CTBP1-AS2\GRHL2	0,326731053	0,000287119
CTBP1-AS2	92070	GRTP1	CTBP1-AS2\GRTP1	0,307057399	0,000720007
CTBP1-AS2	92070	GTF2I	CTBP1-AS2\GTF2I	0,535799744	4,29224E-10
CTBP1-AS2	92070	HIST2H2BF	CTBP1-AS2\HIST2H2BF	0,282174904	0,001945663
CTBP1-AS2	92070	MAML3	CTBP1-AS2\MAML3	0,558973081	0
CTBP1-AS2	92070	MARVELD2	CTBP1-AS2\MARVELD2	0,386704173	1,63357E-05
CTBP1-AS2	92070	RABGAP1	CTBP1-AS2\RABGAP1	0,481840194	4,36443E-08
CTBP1-AS2	92070	RTF1	CTBP1-AS2\RTF1	0,46364478	1,55625E-07
CTBP1-AS2	92070	TSPAN13	CTBP1-AS2\TSPAN13	0,248219627	0,006615131
CTBP1-AS2	92070	UEVLD	CTBP1-AS2\UEVLD	0,484909557	3,49387E-08
CTBP1-AS2	92070	ZNF221	CTBP1-AS2\ZNF221	0,361494089	6,01325E-05
CTBP1-AS2	92070	ALDH7A1	CTBP1-AS2\ALDH7A1	0,385785501	1,7161E-05

CTBP1-AS2	92070	ALS2	CTBP1-AS2\ALS2	0,45410198	2,94432E-07
CTBP1-AS2	92070	ANK3	CTBP1-AS2\ANK3	0,43630537	9,21208E-07
CTBP1-AS2	92070	ANKFY1	CTBP1-AS2\ANKFY1	0,507456203	6,21247E-09
CTBP1-AS2	92070	ARL6IP1	CTBP1-AS2\ARL6IP1	0,413281584	3,6917E-06
CTBP1-AS2	92070	ATP6V1C1	CTBP1-AS2\ATP6V1C1	0,388968808	1,4458E-05
CTBP1-AS2	92070	ATRNL1	CTBP1-AS2\ATRNL1	0,307538648	0,000668252
CTBP1-AS2	92070	BRD1	CTBP1-AS2\BRD1	0,456188577	2,56527E-07
CTBP1-AS2	92070	C1orf109	CTBP1-AS2\C1orf109	0,509136875	5,41632E-09
CTBP1-AS2	92070	CAMSAP1	CTBP1-AS2\CAMSAP1	0,446617291	4,79257E-07
CTBP1-AS2	92070	CCDC30	CTBP1-AS2\CCDC30	0,287380715	0,001591362
CTBP1-AS2	92070	CDH1	CTBP1-AS2\CDH1	0,370922945	3,73756E-05
CTBP1-AS2	92070	CDS1	CTBP1-AS2\CDS1	0,358894744	6,83881E-05
CTBP1-AS2	92070	CETN3	CTBP1-AS2\CETN3	0,288192565	0,001541743
CTBP1-AS2	92070	CLCN5	CTBP1-AS2\CLCN5	0,227218345	0,013104923
CTBP1-AS2	92070	CLINT1	CTBP1-AS2\CLINT1	0,529874662	8,28399E-10
CTBP1-AS2	92070	CLTC	CTBP1-AS2\CLTC	0,38163367	2,14062E-05
CTBP1-AS2	92070	CNTNAP3	CTBP1-AS2\CNTNAP3	0,423258795	2,04678E-06
CTBP1-AS2	92070	DCAKD	CTBP1-AS2\DCAKD	0,269619712	0,003112931
CTBP1-AS2	92070	DDX31	CTBP1-AS2\DDX31	0,246595927	0,006987837
CTBP1-AS2	92070	DHTKD1	CTBP1-AS2\DHTKD1	0,375950719	2,88379E-05
CTBP1-AS2	92070	DMRT2	CTBP1-AS2\DMRT2	0,228236718	0,012693515
CTBP1-AS2	92070	DNAJC16	CTBP1-AS2\DNAJC16	0,470096852	1,00021E-07
CTBP1-AS2	92070	ELOVL6	CTBP1-AS2\ELOVL6	0,408168352	4,96089E-06
CTBP1-AS2	92070	EPN3	CTBP1-AS2\EPN3	0,205412334	0,025179857
CTBP1-AS2	92070	FAM120A	CTBP1-AS2\FAM120A	0,290101125	0,001430602
CTBP1-AS2	92070	FAM154B	CTBP1-AS2\FAM154B	0,479348525	3,48268E-08
CTBP1-AS2	92070	FAM160A1	CTBP1-AS2\FAM160A1	0,394046432	1,09616E-05
CTBP1-AS2	92070	FAM199X	CTBP1-AS2\FAM199X	0,369199544	4,08123E-05
CTBP1-AS2	92070	FBXO22	CTBP1-AS2\FBXO22	0,346339553	0,000125455
CTBP1-AS2	92070	FEM1B	CTBP1-AS2\FEM1B	0,498910412	1,22218E-08
CTBP1-AS2	92070	FKTN	CTBP1-AS2\FKTN	0,434795613	1,01199E-06
CTBP1-AS2	92070	FLVCR1	CTBP1-AS2\FLVCR1	0,25395955	0,005435368
CTBP1-AS2	92070	GRIP1	CTBP1-AS2\GRIP1	0,498589944	1,25284E-08
CTBP1-AS2	92070	HPSE	CTBP1-AS2\HPSE	0,26078194	0,004280183

CTBP1-AS2	92070	IFT140	CTBP1-AS2\IFT140	0,268850591	0,003201694
CTBP1-AS2	92070	IGSF3	CTBP1-AS2\IGSF3	0,292137872	0,001320099
CTBP1-AS2	92070	IQCH	CTBP1-AS2\IQCH	0,260041305	0,004393924
CTBP1-AS2	92070	ITGA2	CTBP1-AS2\ITGA2	0,461408631	1,81011E-07
CTBP1-AS2	92070	KLHDC10	CTBP1-AS2\KLHDC10	0,486611594	3,08494E-08
CTBP1-AS2	92070	LARS	CTBP1-AS2\LARS	0,272482552	0,00280172
CTBP1-AS2	92070	LMBR1	CTBP1-AS2\LMBR1	0,445947871	5,00333E-07
CTBP1-AS2	92070	LRPPRC	CTBP1-AS2\LRPPRC	0,358901866	6,83641E-05
CTBP1-AS2	92070	MAP3K2	CTBP1-AS2\MAP3K2	0,494730095	1,68362E-08
CTBP1-AS2	92070	MAST4	CTBP1-AS2\MAST4	0,362718986	5,65748E-05
CTBP1-AS2	92070	MCTP2	CTBP1-AS2\MCTP2	0,18506623	0,044035214
CTBP1-AS2	92070	METTL8	CTBP1-AS2\METTL8	0,310846033	0,00061425
CTBP1-AS2	92070	MYO10	CTBP1-AS2\MYO10	0,346275459	0,000125836
CTBP1-AS2	92070	NEO1	CTBP1-AS2\NEO1	0,227261074	0,01308743
CTBP1-AS2	92070	NETO2	CTBP1-AS2\NETO2	0,233264492	0,010823921
CTBP1-AS2	92070	NHLRC3	CTBP1-AS2\NHLRC3	0,475252813	6,97809E-08
CTBP1-AS2	92070	NPNT	CTBP1-AS2\NPNT	0,351545364	9,78358E-05
CTBP1-AS2	92070	NRCAM	CTBP1-AS2\NRCAM	0,214093434	0,019547728
CTBP1-AS2	92070	OSBPL3	CTBP1-AS2\OSBPL3	0,432267483	1,18335E-06
CTBP1-AS2	92070	OXCT1	CTBP1-AS2\OXCT1	0,375131748	3,00905E-05
CTBP1-AS2	92070	PAX9	CTBP1-AS2\PAX9	0,311964108	0,000585892
CTBP1-AS2	92070	PGAP1	CTBP1-AS2\PGAP1	0,364891041	5,07464E-05
CTBP1-AS2	92070	PIAS2	CTBP1-AS2\PIAS2	0,531505484	6,97586E-10
CTBP1-AS2	92070	PKP4	CTBP1-AS2\PKP4	0,352172055	9,49242E-05
CTBP1-AS2	92070	PLA2G12A	CTBP1-AS2\PLA2G12A	0,419078479	2,62621E-06
CTBP1-AS2	92070	PMPCB	CTBP1-AS2\PMPCB	0,278891896	0,002204512
CTBP1-AS2	92070	PRRG4	CTBP1-AS2\PRRG4	0,422760291	2,10888E-06
CTBP1-AS2	92070	PSD3	CTBP1-AS2\PSD3	0,397272468	9,173E-06
CTBP1-AS2	92070	PTPRK	CTBP1-AS2\PTPRK	0,192572283	0,036029608
CTBP1-AS2	92070	RAB14	CTBP1-AS2\RAB14	0,364955135	5,05833E-05
CTBP1-AS2	92070	RAD50	CTBP1-AS2\RAD50	0,515510611	3,17117E-09
CTBP1-AS2	92070	RPGRIP1L	CTBP1-AS2\RPGRIP1L	0,476434981	6,41954E-08
CTBP1-AS2	92070	RPS6KA6	CTBP1-AS2\RPS6KA6	0,364243695	4,65524E-05
CTBP1-AS2	92070	SDR42E1	CTBP1-AS2\SDR42E1	0,24842615	0,006569017

CTBP1-AS2	92070	SGPL1	CTBP1-AS2\SGPL1	0,411814556	4,02021E-06
CTBP1-AS2	92070	SIX4	CTBP1-AS2\SIX4	0,307164222	0,000716809
CTBP1-AS2	92070	SLC22A23	CTBP1-AS2\SLC22A23	0,231135166	0,011583894
CTBP1-AS2	92070	SLC30A6	CTBP1-AS2\SLC30A6	0,49427432	1,74281E-08
CTBP1-AS2	92070	SLC46A1	CTBP1-AS2\SLC46A1	0,255846745	0,005090774
CTBP1-AS2	92070	SNX1	CTBP1-AS2\SNX1	0,442679106	6,16578E-07
CTBP1-AS2	92070	SORBS2	CTBP1-AS2\SORBS2	0,353838485	8,75711E-05
CTBP1-AS2	92070	SORCS2	CTBP1-AS2\SORCS2	0,266635807	0,003470194
CTBP1-AS2	92070	SPATA6	CTBP1-AS2\SPATA6	0,48234582	4,2081E-08
CTBP1-AS2	92070	SPIN1	CTBP1-AS2\SPIN1	0,496190001	1,50651E-08
CTBP1-AS2	92070	STEAP2	CTBP1-AS2\STEAP2	0,430878792	1,28889E-06
CTBP1-AS2	92070	STX6	CTBP1-AS2\STX6	0,218145563	0,017315707
CTBP1-AS2	92070	SYT1	CTBP1-AS2\SYT1	0,225053411	0,014018494
CTBP1-AS2	92070	TNPO1	CTBP1-AS2\TNPO1	0,445392394	5,1849E-07
CTBP1-AS2	92070	TP63	CTBP1-AS2\TP63	0,439317761	7,62714E-07
CTBP1-AS2	92070	TRMT5	CTBP1-AS2\TRMT5	0,23425438	0,010485815
CTBP1-AS2	92070	TRPM7	CTBP1-AS2\TRPM7	0,504052129	8,16601E-09
CTBP1-AS2	92070	TRUB1	CTBP1-AS2\TRUB1	0,560197977	0
CTBP1-AS2	92070	TTC22	CTBP1-AS2\TTC22	0,496184592	9,55532E-09
CTBP1-AS2	92070	UBAP2	CTBP1-AS2\UBAP2	0,505269905	7,40979E-09
CTBP1-AS2	92070	UBFD1	CTBP1-AS2\UBFD1	0,516707022	2,8591E-09
CTBP1-AS2	92070	UNC5B	CTBP1-AS2\UNC5B	0,404393961	6,1522E-06
CTBP1-AS2	92070	UPF1	CTBP1-AS2\UPF1	0,357662726	7,26612E-05
CTBP1-AS2	92070	USP40	CTBP1-AS2\USP40	0,311358781	0,000601092
CTBP1-AS2	92070	VMA21	CTBP1-AS2\VMA21	0,231690642	0,011381252
CTBP1-AS2	92070	WDR91	CTBP1-AS2\WDR91	0,26574562	0,003583709
CTBP1-AS2	92070	XPR1	CTBP1-AS2\XPR1	0,403176186	6,59115E-06
CTBP1-AS2	92070	ZBTB41	CTBP1-AS2\ZBTB41	0,510176613	4,9719E-09
CTBP1-AS2	92070	ZMYM3	CTBP1-AS2\ZMYM3	0,374440963	3,11866E-05
CTBP1-AS2	92070	ZNF257	CTBP1-AS2\ZNF257	0,318010255	0,000452355
CTBP1-AS2	92070	ZNF280B	CTBP1-AS2\ZNF280B	0,488990172	2,58878E-08
CTBP1-AS2	92070	ZNF449	CTBP1-AS2\ZNF449	0,394295684	1,08124E-05
CTBP1-AS2	92070	ZNF543	CTBP1-AS2\ZNF543	0,487921948	2,80145E-08
CTBP1-AS2	92070	ZNF562	CTBP1-AS2\ZNF562	0,506081755	6,94228E-09

CTBP1-AS2	92070	ZNF626	CTBP1-AS2\ZNF626	0,374604757	3,09233E-05
CTBP1-AS2	92070	ZRANB3	CTBP1-AS2\ZRANB3	0,526627261	1,14877E-09
CTBP1-AS2	92070	AUTS2	CTBP1-AS2\AUTS2	0,390357499	1,34094E-05
CTBP1-AS2	92070	BBS4	CTBP1-AS2\BBS4	0,388484546	1,48416E-05
CTBP1-AS2	92070	CDC42BPG	CTBP1-AS2\CDC42BPG	0,459806295	2,01581E-07
CTBP1-AS2	92070	CLDN12	CTBP1-AS2\CLDN12	0,393519442	1,12834E-05
CTBP1-AS2	92070	DET1	CTBP1-AS2\DET1	0,337993163	0,000185321
CTBP1-AS2	92070	ESRP1	CTBP1-AS2\ESRP1	0,199772112	0,029541397
CTBP1-AS2	92070	EYA2	CTBP1-AS2\EYA2	0,190969947	0,037626746
CTBP1-AS2	92070	FBXW11	CTBP1-AS2\FBXW11	0,463473864	1,57439E-07
CTBP1-AS2	92070	GTF3C4	CTBP1-AS2\GTF3C4	0,478172625	5,67517E-08
CTBP1-AS2	92070	HPS5	CTBP1-AS2\HPS5	0,456480558	2,5161E-07
CTBP1-AS2	92070	KDM4D	CTBP1-AS2\KDM4D	0,370588235	3,8021E-05
CTBP1-AS2	92070	LAMP2	CTBP1-AS2\LAMP2	0,364841191	5,08736E-05
CTBP1-AS2	92070	LIMK1	CTBP1-AS2\LIMK1	0,264421023	0,003758814
CTBP1-AS2	92070	LRIG3	CTBP1-AS2\LRIG3	0,443220339	5,95699E-07
CTBP1-AS2	92070	MCOLN3	CTBP1-AS2\MCOLN3	0,26078194	0,004280183
CTBP1-AS2	92070	MPP5	CTBP1-AS2\MPP5	0,355953568	7,90043E-05
CTBP1-AS2	92070	N4BP1	CTBP1-AS2\N4BP1	0,349259365	0,000109179
CTBP1-AS2	92070	NUDT12	CTBP1-AS2\NUDT12	0,267732517	0,003334803
CTBP1-AS2	92070	PABPC4L	CTBP1-AS2\PABPC4L	0,383150548	1,97519E-05
CTBP1-AS2	92070	PANK1	CTBP1-AS2\PANK1	0,375829654	2,90199E-05
CTBP1-AS2	92070	PRRC1	CTBP1-AS2\PRRC1	0,461472725	1,80232E-07
CTBP1-AS2	92070	PSMD12	CTBP1-AS2\PSMD12	0,202086597	0,027679414
CTBP1-AS2	92070	RAB23	CTBP1-AS2\RAB23	0,398575701	8,53186E-06
CTBP1-AS2	92070	RANBP17	CTBP1-AS2\RANBP17	0,238990172	0,008993701
CTBP1-AS2	92070	RRAGB	CTBP1-AS2\RRAGB	0,458552913	2,19205E-07
CTBP1-AS2	92070	SLC22A5	CTBP1-AS2\SLC22A5	0,32337986	0,000357942
CTBP1-AS2	92070	SLC30A1	CTBP1-AS2\SLC30A1	0,331042587	0,000254446
CTBP1-AS2	92070	TM7SF3	CTBP1-AS2\TM7SF3	0,331612306	0,000247986
CTBP1-AS2	92070	WASL	CTBP1-AS2\WASL	0,408118502	4,97509E-06
FAM66E	100132103	ALDH7A1	FAM66E\ALDH7A1	0,351158635	9,01382E-05
FAM66E	100132103	ALS2	FAM66E\ALS2	0,531082847	5,18152E-10
FAM66E	100132103	ANK3	FAM66E\ANK3	0,570226184	1,29397E-11

FAM66E	100132103	AP2B1	FAM66E\AP2B1	0,479863374	3,35106E-08
FAM66E	100132103	ATP6AP2	FAM66E\ATP6AP2	0,397333945	7,6671E-06
FAM66E	100132103	AUTS2	FAM66E\AUTS2	0,404365917	5,09361E-06
FAM66E	100132103	BBS4	FAM66E\BBS4	0,300497005	0,000898485
FAM66E	100132103	BRD1	FAM66E\BRD1	0,273351234	0,002630244
FAM66E	100132103	BTBD3	FAM66E\BTBD3	0,488207457	1,77888E-08
FAM66E	100132103	C15orf41	FAM66E\C15orf41	0,251084631	0,005880565
FAM66E	100132103	CCDC122	FAM66E\CCDC122	0,551825202	7,78254E-11
FAM66E	100132103	CCDC148	FAM66E\CCDC148	0,475727535	4,55838E-08
FAM66E	100132103	CCDC30	FAM66E\CCDC30	0,37421879	2,75854E-05
FAM66E	100132103	CCDC8	FAM66E\CCDC8	0,301840544	0,000849621
FAM66E	100132103	CDH1	FAM66E\CDH1	0,544950368	1,47995E-10
FAM66E	100132103	CDKL3	FAM66E\CDKL3	0,276116882	0,002368869
FAM66E	100132103	CDS1	FAM66E\CDS1	0,502569966	5,74337E-09
FAM66E	100132103	CETN3	FAM66E\CETN3	0,308047536	0,00065392
FAM66E	100132103	CLDN12	FAM66E\CLDN12	0,575003211	7,9739E-12
FAM66E	100132103	CLTC	FAM66E\CLTC	0,516712481	1,79073E-09
FAM66E	100132103	CNTNAP3	FAM66E\CNTNAP3	0,502640679	5,71074E-09
FAM66E	100132103	COG5	FAM66E\COG5	0,57451608	8,38043E-12
FAM66E	100132103	DDX31	FAM66E\DDX31	0,398976048	6,97457E-06
FAM66E	100132103	DET1	FAM66E\DET1	0,222414611	0,015049699
FAM66E	100132103	DHTKD1	FAM66E\DHTKD1	0,209521352	0,022199998
FAM66E	100132103	DHX32	FAM66E\DHX32	0,325419258	0,000304733
FAM66E	100132103	DMRTA1	FAM66E\DMRTA1	0,41715215	2,36365E-06
FAM66E	100132103	DNAJC16	FAM66E\DNAJC16	0,525850117	8,19381E-10
FAM66E	100132103	DUOX1	FAM66E\DUOX1	0,327430638	0,000278115
FAM66E	100132103	DUOXA1	FAM66E\DUOXA1	0,325794336	0,000299597
FAM66E	100132103	EDA2R	FAM66E\EDA2R	0,403800217	5,26578E-06
FAM66E	100132103	EIF2AK4	FAM66E\EIF2AK4	0,409802928	3,68904E-06
FAM66E	100132103	ENPEP	FAM66E\ENPEP	0,414650668	2,7536E-06
FAM66E	100132103	EPN3	FAM66E\EPN3	0,330007719	0,000247153
FAM66E	100132103	EPS15L1	FAM66E\EPS15L1	0,195920967	0,032726138
FAM66E	100132103	ESRP1	FAM66E\ESRP1	0,461627387	1,26284E-07
FAM66E	100132103	FAM154B	FAM66E\FAM154B	0,404508062	5,05119E-06

FAM66E	100132103	FAM160A1	FAM66E\FAM160A1	0,559910006	3,58626E-11
FAM66E	100132103	FAM199X	FAM66E\FAM199X	0,612567301	1,32789E-13
FAM66E	100132103	FEM1B	FAM66E\FEM1B	0,402016688	5,84533E-06
FAM66E	100132103	FKTN	FAM66E\FKTN	0,667668109	1,10486E-16
FAM66E	100132103	FNBP1L	FAM66E\FNBP1L	0,492937343	1,23295E-08
FAM66E	100132103	FOXE1	FAM66E\FOXE1	0,301709124	0,000854291
FAM66E	100132103	GLI2	FAM66E\GLI2	0,466655836	8,82691E-08
FAM66E	100132103	GNPDA1	FAM66E\GNPDA1	0,393782602	9,39297E-06
FAM66E	100132103	GRHL2	FAM66E\GRHL2	0,436025768	7,17287E-07
FAM66E	100132103	GRIP1	FAM66E\GRIP1	0,423914015	1,55463E-06
FAM66E	100132103	GRTP1	FAM66E\GRTP1	0,353217156	8,13931E-05
FAM66E	100132103	GTF3C4	FAM66E\GTF3C4	0,50276639	5,65319E-09
FAM66E	100132103	HIST2H2BF	FAM66E\HIST2H2BF	0,477671284	3,94665E-08
FAM66E	100132103	HOMER2	FAM66E\HOMER2	0,370227459	3,40809E-05
FAM66E	100132103	HPSE	FAM66E\HPSE	0,474363507	5,04077E-08
FAM66E	100132103	IFI44	FAM66E\IFI44	0,237099255	0,009423975
FAM66E	100132103	IGSF3	FAM66E\IGSF3	0,459623864	1,45422E-07
FAM66E	100132103	IQCH	FAM66E\IQCH	0,28259101	0,001846379
FAM66E	100132103	IRF2BP2	FAM66E\IRF2BP2	0,539269791	2,48981E-10
FAM66E	100132103	ITGA2	FAM66E\ITGA2	0,561017836	3,21979E-11
FAM66E	100132103	KDM4D	FAM66E\KDM4D	0,573353251	9,43352E-12
FAM66E	100132103	KLHDC10	FAM66E\KLHDC10	0,590646404	1,54472E-12
FAM66E	100132103	LARS	FAM66E\LARS	0,270970578	0,002875775
FAM66E	100132103	LEPREL1	FAM66E\LEPREL1	0,181393462	0,048348429
FAM66E	100132103	LIMK1	FAM66E\LIMK1	0,394662581	8,93407E-06
FAM66E	100132103	LRIG3	FAM66E\LRIG3	0,567224828	1,74706E-11
FAM66E	100132103	LRPPRC	FAM66E\LRPPRC	0,428062486	1,19682E-06
FAM66E	100132103	MAML3	FAM66E\MAML3	0,568749077	1,50057E-11
FAM66E	100132103	MAP3K2	FAM66E\MAP3K2	0,615325092	9,62083E-14
FAM66E	100132103	MARVELD2	FAM66E\MARVELD2	0,544478951	1,54581E-10
FAM66E	100132103	MCOLN3	FAM66E\MCOLN3	0,450525513	2,72925E-07
FAM66E	100132103	MCTP2	FAM66E\MCTP2	0,251108202	0,005875765
FAM66E	100132103	MPP5	FAM66E\MPP5	0,508423396	3,56844E-09
FAM66E	100132103	MYO10	FAM66E\MYO10	0,469806474	7,03191E-08

FAM66E	100132103	N4BP1	FAM66E\N4BP1	0,486895346	1,96736E-08
FAM66E	100132103	NEO1	FAM66E\NEO1	0,488600305	1,7259E-08
FAM66E	100132103	NETO2	FAM66E\NETO2	0,269304904	0,003059648
FAM66E	100132103	NHLRC3	FAM66E\NHLRC3	0,590701402	1,5356E-12
FAM66E	100132103	NPNT	FAM66E\NPNT	0,306279722	0,000704954
FAM66E	100132103	NRCAM	FAM66E\NRCAM	0,290055115	0,001375181
FAM66E	100132103	NUDT12	FAM66E\NUDT12	0,458076044	1,62071E-07
FAM66E	100132103	PABPC4L	FAM66E\PABPC4L	0,601426143	4,73081E-13
FAM66E	100132103	PANK1	FAM66E\PANK1	0,423859016	1,55999E-06
FAM66E	100132103	PAX1	FAM66E\PAX1	0,28413883	0,001738075
FAM66E	100132103	PAX9	FAM66E\PAX9	0,607342428	2,42441E-13
FAM66E	100132103	PDGFA	FAM66E\PDGFA	0,196777375	0,031957009
FAM66E	100132103	PGAP1	FAM66E\PGAP1	0,555840104	5,31073E-11
FAM66E	100132103	PKP4	FAM66E\PKP4	0,461093114	1,31138E-07
FAM66E	100132103	PLA2G12A	FAM66E\PLA2G12A	0,510301208	3,05732E-09
FAM66E	100132103	PLD2	FAM66E\PLD2	0,397443942	7,61876E-06
FAM66E	100132103	POMT2	FAM66E\POMT2	0,2794718	0,002083434
FAM66E	100132103	PRKAR1A	FAM66E\PRKAR1A	0,584297986	3,0389E-12
FAM66E	100132103	PRRC1	FAM66E\PRRC1	0,49278806	1,24741E-08
FAM66E	100132103	PSD3	FAM66E\PSD3	0,525229417	8,6471E-10
FAM66E	100132103	RAB14	FAM66E\RAB14	0,496905104	9,02662E-09
FAM66E	100132103	RAB23	FAM66E\RAB23	0,536794851	3,11377E-10
FAM66E	100132103	RABGAP1	FAM66E\RABGAP1	0,58101378	4,28806E-12
FAM66E	100132103	RAD50	FAM66E\RAD50	0,581862331	3,92448E-12
FAM66E	100132103	RANBP17	FAM66E\RANBP17	0,445795628	3,75902E-07
FAM66E	100132103	RNF170	FAM66E\RNF170	0,567319112	1,73074E-11
FAM66E	100132103	RPGRIP1L	FAM66E\RPGRIP1L	0,505233473	4,63019E-09
FAM66E	100132103	RPS6KA6	FAM66E\RPS6KA6	0,59233971	1,28642E-12
FAM66E	100132103	RRM2B	FAM66E\RRM2B	0,506270591	4,25548E-09
FAM66E	100132103	RTF1	FAM66E\RTF1	0,274066217	0,00256029
FAM66E	100132103	SDR42E1	FAM66E\SDR42E1	0,351158635	9,01382E-05
FAM66E	100132103	SGPL1	FAM66E\SGPL1	0,579528816	5,00417E-12
FAM66E	100132103	SIX1	FAM66E\SIX1	0,530603573	5,4054E-10
FAM66E	100132103	SIX4	FAM66E\SIX4	0,48874173	1,7072E-08

FAM66E	100132103	SLC22A5	FAM66E\SLC22A5	0,449472681	2,93205E-07
FAM66E	100132103	SLC30A1	FAM66E\SLC30A1	0,509106951	3,37354E-09
FAM66E	100132103	SLC46A1	FAM66E\SLC46A1	0,285348801	0,001657462
FAM66E	100132103	SMO	FAM66E\SMO	0,303922636	0,000778684
FAM66E	100132103	SORBS2	FAM66E\SORBS2	0,427206078	1,26358E-06
FAM66E	100132103	SORCS2	FAM66E\SORCS2	0,314584521	0,000493263
FAM66E	100132103	SPATA6	FAM66E\SPATA6	0,60749171	2,38344E-13
FAM66E	100132103	SPIN1	FAM66E\SPIN1	0,456088235	1,86132E-07
FAM66E	100132103	SPINT1	FAM66E\SPINT1	0,310412479	0,000590947
FAM66E	100132103	STARD7	FAM66E\STARD7	0,280681771	0,001988388
FAM66E	100132103	STEAP2	FAM66E\STEAP2	0,542467571	1,85998E-10
FAM66E	100132103	SYBU	FAM66E\SYBU	0,352258608	8,53618E-05
FAM66E	100132103	SYT1	FAM66E\SYT1	0,278764675	0,002140867
FAM66E	100132103	TMEM185A	FAM66E\TMEM185A	0,187066182	0,041636896
FAM66E	100132103	TMEM30B	FAM66E\TMEM30B	0,480256221	3,25385E-08
FAM66E	100132103	TP63	FAM66E\TP63	0,504848483	4,7772E-09
FAM66E	100132103	TRMT5	FAM66E\TRMT5	0,44421638	4,1786E-07
FAM66E	100132103	TRPM7	FAM66E\TRPM7	0,523681598	9,88505E-10
FAM66E	100132103	TSPAN13	FAM66E\TSPAN13	0,306531145	0,000697479
FAM66E	100132103	TTC22	FAM66E\TTC22	0,557515704	4,52098E-11
FAM66E	100132103	UBTD2	FAM66E\UBTD2	0,429162459	1,11597E-06
FAM66E	100132103	UEVLD	FAM66E\UEVLD	0,563995621	2,40509E-11
FAM66E	100132103	UNC5B	FAM66E\UNC5B	0,503999932	5,1172E-09
FAM66E	100132103	USP46	FAM66E\USP46	0,509342659	3,30874E-09
FAM66E	100132103	WASL	FAM66E\WASL	0,562494943	2,78701E-11
FAM66E	100132103	WDR91	FAM66E\WDR91	0,277971122	0,002207022
FAM66E	100132103	XPR1	FAM66E\XPR1	0,523123754	1,03717E-09
FAM66E	100132103	ZBTB41	FAM66E\ZBTB41	0,579670241	4,93127E-12
FAM66E	100132103	ZNF280B	FAM66E\ZNF280B	0,618326447	6,75063E-14
FAM66E	100132103	ZNF449	FAM66E\ZNF449	0,532992086	4,37511E-10
FAM66E	100132103	ZNF626	FAM66E\ZNF626	0,539206936	2,50404E-10
FAM95B1	100133036	AP1S1	FAM95B1\AP1S1	0,252200541	0,005775171
FAM95B1	100133036	ARV1	FAM95B1\ARV1	0,24918815	0,006401337
FAM95B1	100133036	ATP6AP2	FAM95B1\ATP6AP2	0,394430993	1,07323E-05

FAM95B1	100133036	C15orf41	FAM95B1\C15orf41	0,326577411	0,000310752
FAM95B1	100133036	DMRTA1	FAM95B1\DMRTA1	0,244304546	0,007414769
FAM95B1	100133036	DUOX1	FAM95B1\DUOX1	0,363367042	5,47732E-05
FAM95B1	100133036	EPS15L1	FAM95B1\EPS15L1	0,237323743	0,00949584
FAM95B1	100133036	ESRP1	FAM95B1\ESRP1	0,342643498	0,000149306
FAM95B1	100133036	EYA2	FAM95B1\EYA2	0,231697764	0,011378675
FAM95B1	100133036	GPHN	FAM95B1\GPHN	0,259621137	0,004459649
FAM95B1	100133036	KRTAP5-8	FAM95B1\KRTAP5-8	0,265940249	0,003463688
FAM95B1	100133036	LAMP2	FAM95B1\LAMP2	0,200940037	0,028588894
FAM95B1	100133036	LIMK1	FAM95B1\LIMK1	0,481576698	4,44807E-08
FAM95B1	100133036	MCOLN3	FAM95B1\MCOLN3	0,342622134	0,000149456
FAM95B1	100133036	MFSD3	FAM95B1\MFSD3	0,256266914	0,005016767
FAM95B1	100133036	NAGK	FAM95B1\NAGK	0,364762854	5,10741E-05
FAM95B1	100133036	NUDT12	FAM95B1\NUDT12	0,244430993	0,007513775
FAM95B1	100133036	PABPC4L	FAM95B1\PABPC4L	0,258802165	0,004590297
FAM95B1	100133036	PLD2	FAM95B1\PLD2	0,353453924	8,92191E-05
FAM95B1	100133036	PLEK2	FAM95B1\PLEK2	0,20300527	0,026968636
FAM95B1	100133036	RFESD	FAM95B1\RFESD	0,283378436	0,001857913
FAM95B1	100133036	RNF170	FAM95B1\RNF170	0,231270474	0,011534243
FAM95B1	100133036	SGPP2	FAM95B1\SGPP2	0,24957271	0,00631817
FAM95B1	100133036	SLC22A5	FAM95B1\SLC22A5	0,220338983	0,016202472
FAM95B1	100133036	SLC44A3	FAM95B1\SLC44A3	0,357669848	7,26358E-05
FAM95B1	100133036	SPINT1	FAM95B1\SPINT1	0,268786498	0,003209193
FAM95B1	100133036	SYBU	FAM95B1\SYBU	0,209521436	0,022360867
FAM95B1	100133036	TMEM129	FAM95B1\TMEM129	0,358025922	7,13762E-05
FAM95B1	100133036	UBAC1	FAM95B1\UBAC1	0,351751887	9,68672E-05
FAM95B1	100133036	VAC14	FAM95B1\VAC14	0,244188862	0,007574728
FAM95B1	100133036	WNK2	FAM95B1\WNK2	0,349779234	0,000106496
FAM95B1	100133036	ACAD10	FAM95B1\ACAD10	0,344473722	0,000137012
FAM95B1	100133036	ANK3	FAM95B1\ANK3	0,235187295	0,010175718
FAM95B1	100133036	ANKFY1	FAM95B1\ANKFY1	0,210126763	0,021969459
FAM95B1	100133036	AP2B1	FAM95B1\AP2B1	0,250968523	0,006024334
FAM95B1	100133036	APTX	FAM95B1\APTX	0,37900584	2,45848E-05
FAM95B1	100133036	ATP2C1	FAM95B1\ATP2C1	0,33034468	0,000262572

FAM95B1	100133036	ATP6V0B	FAM95B1\ATP6V0B	0,253738784	0,005477012
FAM95B1	100133036	C19orf54	FAM95B1\C19orf54	0,31207093	0,000583247
FAM95B1	100133036	CAMSAP1	FAM95B1\CAMSAP1	0,287480416	0,001585191
FAM95B1	100133036	CBLN3	FAM95B1\CBLN3	0,229105541	0,012351515
FAM95B1	100133036	CCDC30	FAM95B1\CCDC30	0,18258795	0,046985024
FAM95B1	100133036	CDH1	FAM95B1\CDH1	0,241561031	0,008265025
FAM95B1	100133036	CDKL3	FAM95B1\CDKL3	0,327268195	0,000301346
FAM95B1	100133036	CDS1	FAM95B1\CDS1	0,331498362	0,000249265
FAM95B1	100133036	CNTNAP3	FAM95B1\CNTNAP3	0,233143427	0,010865924
FAM95B1	100133036	DDX31	FAM95B1\DDX31	0,281014101	0,002033849
FAM95B1	100133036	DENND1A	FAM95B1\DENND1A	0,459642501	2,03804E-07
FAM95B1	100133036	DNAJC16	FAM95B1\DNAJC16	0,198931776	0,030243336
FAM95B1	100133036	ELOVL6	FAM95B1\ELOVL6	0,322112235	0,000378421
FAM95B1	100133036	FAHD1	FAM95B1\FAHD1	0,357093007	7,47206E-05
FAM95B1	100133036	FAM120A	FAM95B1\FAM120A	0,488413331	2,70165E-08
FAM95B1	100133036	FAM154B	FAM95B1\FAM154B	0,303333399	0,000798188
FAM95B1	100133036	FAM199X	FAM95B1\FAM199X	0,330052699	0,000266042
FAM95B1	100133036	FKTN	FAM95B1\FKTN	0,263082182	0,003943586
FAM95B1	100133036	FLVCR1	FAM95B1\FLVCR1	0,410945734	4,22763E-06
FAM95B1	100133036	FSTL4	FAM95B1\FSTL4	0,366415041	4,69984E-05
FAM95B1	100133036	GGA2	FAM95B1\GGA2	0,399558467	8,07665E-06
FAM95B1	100133036	GGCT	FAM95B1\GGCT	0,312256089	0,000578688
FAM95B1	100133036	GLI2	FAM95B1\GLI2	0,247671272	0,006738973
FAM95B1	100133036	GRHL2	FAM95B1\GRHL2	0,204684986	0,025550366
FAM95B1	100133036	GRIP1	FAM95B1\GRIP1	0,338541518	0,000180689
FAM95B1	100133036	GRTP1	FAM95B1\GRTP1	0,207384988	0,023790658
FAM95B1	100133036	HIST2H2BF	FAM95B1\HIST2H2BF	0,415339695	3,2735E-06
FAM95B1	100133036	HPSE	FAM95B1\HPSE	0,264705882	0,00372052
FAM95B1	100133036	KIAA1549	FAM95B1\KIAA1549	0,25362484	0,005498616
FAM95B1	100133036	LARS	FAM95B1\LARS	0,225160234	0,013972145
FAM95B1	100133036	LMBR1	FAM95B1\LMBR1	0,189510041	0,039133442
FAM95B1	100133036	MAML3	FAM95B1\MAML3	0,206943455	0,024095744
FAM95B1	100133036	MARVELD2	FAM95B1\MARVELD2	0,219384703	0,016678909
FAM95B1	100133036	MTSS1L	FAM95B1\MTSS1L	0,220865974	0,015944474

FAM95B1	100133036	NCS1	FAM95B1\NCS1	0,315083321	0,000513031
FAM95B1	100133036	NDUFA4	FAM95B1\NDUFA4	0,216336704	0,01828315
FAM95B1	100133036	NETO2	FAM95B1\NETO2	0,251203532	0,005976079
FAM95B1	100133036	NRCAM	FAM95B1\NRCAM	0,19828372	0,030794307
FAM95B1	100133036	OSBPL3	FAM95B1\OSBPL3	0,295627404	0,00114874
FAM95B1	100133036	PKP1	FAM95B1\PKP1	0,26741205	0,003373864
FAM95B1	100133036	PRSS8	FAM95B1\PRSS8	0,40329013	6,54885E-06
FAM95B1	100133036	S100A14	FAM95B1\S100A14	0,32576556	0,000322154
FAM95B1	100133036	SLC6A8	FAM95B1\SLC6A8	0,478799316	5,42744E-08
FAM95B1	100133036	SORBS2	FAM95B1\SORBS2	0,218181171	0,017297118
FAM95B1	100133036	SPIRE2	FAM95B1\SPIRE2	0,558545791	0
FAM95B1	100133036	STARD7	FAM95B1\STARD7	0,242479704	0,008017614
FAM95B1	100133036	STX6	FAM95B1\STX6	0,443647629	5,79694E-07
FAM95B1	100133036	TMEM180	FAM95B1\TMEM180	0,260888762	0,004263998
FAM95B1	100133036	TP63	FAM95B1\TP63	0,187665575	0,041109257
FAM95B1	100133036	TRIM7	FAM95B1\TRIM7	0,22423444	0,014378327
FAM95B1	100133036	VMA21	FAM95B1\VMA21	0,191247686	0,037345711
FAM95B1	100133036	WDR91	FAM95B1\WDR91	0,317048853	0,000471513
FAM95B1	100133036	XYLT2	FAM95B1\XYLT2	0,343854152	0,000141064
KCNQ1OT1	10984	APP	KCNQ1OT1\APP	0,546353796	8,11494E-11
KCNQ1OT1	10984	ATP6AP2	KCNQ1OT1\ATP6AP2	0,393291554	1,14253E-05
KCNQ1OT1	10984	AUTS2	KCNQ1OT1\AUTS2	0,607078764	0
KCNQ1OT1	10984	C14orf39	KCNQ1OT1\C14orf39	0,622006563	4,34936E-14
KCNQ1OT1	10984	C15orf41	KCNQ1OT1\C15orf41	0,451103831	3,58358E-07
KCNQ1OT1	10984	C5orf15	KCNQ1OT1\C5orf15	0,524077767	1,46785E-09
KCNQ1OT1	10984	CBS	KCNQ1OT1\CBS	0,215724256	0,01862116
KCNQ1OT1	10984	CCDC148	KCNQ1OT1\CCDC148	0,579480451	5,02934E-12
KCNQ1OT1	10984	CDC42BPG	KCNQ1OT1\CDC42BPG	0,548020225	5,20003E-11
KCNQ1OT1	10984	CLDN12	KCNQ1OT1\CLDN12	0,648532973	0
KCNQ1OT1	10984	CLSTN1	KCNQ1OT1\CLSTN1	0,454778522	2,81593E-07
KCNQ1OT1	10984	COG5	KCNQ1OT1\COG5	0,50081185	1,0542E-08
KCNQ1OT1	10984	DET1	KCNQ1OT1\DET1	0,223778664	0,014582053
KCNQ1OT1	10984	DHX32	KCNQ1OT1\DHX32	0,473899729	7,67413E-08
KCNQ1OT1	10984	DMRTA1	KCNQ1OT1\DMRTA1	0,321544805	0,000362754

KCNQ1OT1	10984	DSG2	KCNQ1OT1\DSG2	0,547892038	5,40679E-11
KCNQ1OT1	10984	DUOX1	KCNQ1OT1\DUOX1	0,484852585	3,50841E-08
KCNQ1OT1	10984	EIF2AK4	KCNQ1OT1\EIF2AK4	0,581370175	0
KCNQ1OT1	10984	EPS15L1	KCNQ1OT1\EPS15L1	0,18139154	0,048466935
KCNQ1OT1	10984	ESRP1	KCNQ1OT1\ESRP1	0,373864122	3,21305E-05
KCNQ1OT1	10984	FAM83B	KCNQ1OT1\FAM83B	0,623244552	0
KCNQ1OT1	10984	GTF3C4	KCNQ1OT1\GTF3C4	0,486476285	3,11571E-08
KCNQ1OT1	10984	HPS5	KCNQ1OT1\HPS5	0,673928215	0
KCNQ1OT1	10984	KDM4D	KCNQ1OT1\KDM4D	0,441639368	6,5866E-07
KCNQ1OT1	10984	KTN1	KCNQ1OT1\KTN1	0,615994873	0
KCNQ1OT1	10984	LEPREL1	KCNQ1OT1\LEPREL1	0,407883492	5,04256E-06
KCNQ1OT1	10984	LRIG3	KCNQ1OT1\LRIG3	0,616493377	0
KCNQ1OT1	10984	MBLAC2	KCNQ1OT1\MBLAC2	0,584282866	0
KCNQ1OT1	10984	MPP5	KCNQ1OT1\MPP5	0,306509044	0,000736631
KCNQ1OT1	10984	N4BP1	KCNQ1OT1\N4BP1	0,585101837	0
KCNQ1OT1	10984	NET1	KCNQ1OT1\NET1	0,575267056	0
KCNQ1OT1	10984	NTF4	KCNQ1OT1\NTF4	0,285764136	0,001694523
KCNQ1OT1	10984	NUDT12	KCNQ1OT1\NUDT12	0,518138442	2,52204E-09
KCNQ1OT1	10984	NUDT9	KCNQ1OT1\NUDT9	0,497443384	1,36857E-08
KCNQ1OT1	10984	PABPC4L	KCNQ1OT1\PABPC4L	0,642301666	0
KCNQ1OT1	10984	PCSK6	KCNQ1OT1\PCSK6	0,19809144	0,030959414
KCNQ1OT1	10984	PLD2	KCNQ1OT1\PLD2	0,323415468	0,000357382
KCNQ1OT1	10984	PRKAR1A	KCNQ1OT1\PRKAR1A	0,697941889	0
KCNQ1OT1	10984	PRRC1	KCNQ1OT1\PRRC1	0,579112662	0
KCNQ1OT1	10984	RAB23	KCNQ1OT1\RAB23	0,59789916	0
KCNQ1OT1	10984	RANBP17	KCNQ1OT1\RANBP17	0,700384561	0
KCNQ1OT1	10984	RNF170	KCNQ1OT1\RNF170	0,696140151	0
KCNQ1OT1	10984	RRM2B	KCNQ1OT1\RRM2B	0,568758012	0
KCNQ1OT1	10984	SERPINB5	KCNQ1OT1\SERPINB5	0,49322746	1,88626E-08
KCNQ1OT1	10984	SHROOM2	KCNQ1OT1\SHROOM2	0,230301951	0,0118938
KCNQ1OT1	10984	SIX1	KCNQ1OT1\SIX1	0,611956986	0
KCNQ1OT1	10984	SLC22A5	KCNQ1OT1\SLC22A5	0,522532403	1,69599E-09
KCNQ1OT1	10984	SLC30A1	KCNQ1OT1\SLC30A1	0,535051987	4,69034E-10
KCNQ1OT1	10984	SLC45A4	KCNQ1OT1\SLC45A4	0,303567868	0,000831975

KCNQ1OT1	10984	SPIN4	KCNQ1OT1\SPIN4	0,462199117	1,71617E-07
KCNQ1OT1	10984	SPINT1	KCNQ1OT1\SPINT1	0,370346105	3,84944E-05
KCNQ1OT1	10984	STK36	KCNQ1OT1\STK36	0,710361772	0
KCNQ1OT1	10984	SUPT3H	KCNQ1OT1\SUPT3H	0,398212505	8,70614E-06
KCNQ1OT1	10984	TM7SF3	KCNQ1OT1\TM7SF3	0,293327161	0,001259237
KCNQ1OT1	10984	TMEM133	KCNQ1OT1\TMEM133	0,363103547	5,54991E-05
KCNQ1OT1	10984	UBTD2	KCNQ1OT1\UBTD2	0,471976926	8,77783E-08
KCNQ1OT1	10984	VPS35	KCNQ1OT1\VPS35	0,461665005	1,77912E-07
KCNQ1OT1	10984	WASL	KCNQ1OT1\WASL	0,744922376	0
KCNQ1OT1	10984	ZNF211	KCNQ1OT1\ZNF211	0,514292836	3,51994E-09
KCNQ1OT1	10984	ZNF540	KCNQ1OT1\ZNF540	0,562163509	0
KCNQ1OT1	10984	ALDH7A1	KCNQ1OT1\ALDH7A1	0,432659165	1,15511E-06
KCNQ1OT1	10984	ALS2	KCNQ1OT1\ALS2	0,645506338	0
KCNQ1OT1	10984	ANK3	KCNQ1OT1\ANK3	0,688513032	0
KCNQ1OT1	10984	AP2B1	KCNQ1OT1\AP2B1	0,302022504	0,000886492
KCNQ1OT1	10984	ATP6V0A1	KCNQ1OT1\ATP6V0A1	0,213317191	0,020002589
KCNQ1OT1	10984	AZIN1	KCNQ1OT1\AZIN1	0,399487253	8,10885E-06
KCNQ1OT1	10984	BAIAP2	KCNQ1OT1\BAIAP2	0,271435693	0,002912101
KCNQ1OT1	10984	BRD1	KCNQ1OT1\BRD1	0,385678678	1,72595E-05
KCNQ1OT1	10984	C19orf44	KCNQ1OT1\C19orf44	0,180757727	0,049267614
KCNQ1OT1	10984	C1orf109	KCNQ1OT1\C1orf109	0,534054978	5,26352E-10
KCNQ1OT1	10984	CCDC122	KCNQ1OT1\CCDC122	0,587437687	0
KCNQ1OT1	10984	CCDC30	KCNQ1OT1\CCDC30	0,678564307	0
KCNQ1OT1	10984	CCDC8	KCNQ1OT1\CCDC8	0,412519584	3,85902E-06
KCNQ1OT1	10984	CDH1	KCNQ1OT1\CDH1	0,562156388	0
KCNQ1OT1	10984	CDKL3	KCNQ1OT1\CDKL3	0,252043868	0,005806337
KCNQ1OT1	10984	CDS1	KCNQ1OT1\CDS1	0,393804301	1,11083E-05
KCNQ1OT1	10984	CHID1	KCNQ1OT1\CHID1	0,187316622	0,04149232
KCNQ1OT1	10984	CNTNAP2	KCNQ1OT1\CNTNAP2	0,288783649	0,001506509
KCNQ1OT1	10984	CNTNAP3	KCNQ1OT1\CNTNAP3	0,472454066	8,49064E-08
KCNQ1OT1	10984	COL8A2	KCNQ1OT1\COL8A2	0,20095428	0,028577442
KCNQ1OT1	10984	CRTC1	KCNQ1OT1\CRTC1	0,449722262	3,92083E-07
KCNQ1OT1	10984	DCAKD	KCNQ1OT1\DCAKD	0,419049993	2,63065E-06
KCNQ1OT1	10984	DDX31	KCNQ1OT1\DDX31	0,355540521	8,06131E-05

KCNQ1OT1	10984	DENND1A	KCNQ1OT1\DENND1A	0,240250677	0,00862962
KCNQ1OT1	10984	DNAJC16	KCNQ1OT1\DNAJC16	0,610981342	0
KCNQ1OT1	10984	DNAJC21	KCNQ1OT1\DNAJC21	0,277695485	0,002306353
KCNQ1OT1	10984	DUOXA1	KCNQ1OT1\DUOXA1	0,559844749	3,60906E-11
KCNQ1OT1	10984	EDA2R	KCNQ1OT1\EDA2R	0,450861701	3,64061E-07
KCNQ1OT1	10984	EFNB2	KCNQ1OT1\EFNB2	0,520616721	2,02105E-09
KCNQ1OT1	10984	EXOC7	KCNQ1OT1\EXOC7	0,370103974	3,89734E-05
KCNQ1OT1	10984	FAM154B	KCNQ1OT1\FAM154B	0,401955565	5,86622E-06
KCNQ1OT1	10984	FAM160A1	KCNQ1OT1\FAM160A1	0,698269477	0
KCNQ1OT1	10984	FBXL16	KCNQ1OT1\FBXL16	0,230138157	0,01195557
KCNQ1OT1	10984	FEM1B	KCNQ1OT1\FEM1B	0,573985187	0
KCNQ1OT1	10984	FNBP1L	KCNQ1OT1\FNBP1L	0,712441248	0
KCNQ1OT1	10984	FOXE1	KCNQ1OT1\FOXE1	0,220390403	0,016018607
KCNQ1OT1	10984	GLI2	KCNQ1OT1\GLI2	0,380408774	2,28363E-05
KCNQ1OT1	10984	GNPDA1	KCNQ1OT1\GNPDA1	0,529803447	8,34533E-10
KCNQ1OT1	10984	GOLM1	KCNQ1OT1\GOLM1	0,372247543	3,4921E-05
KCNQ1OT1	10984	GPC4	KCNQ1OT1\GPC4	0,433955277	1,06614E-06
KCNQ1OT1	10984	GPR107	KCNQ1OT1\GPR107	0,610226463	0
KCNQ1OT1	10984	GRHL2	KCNQ1OT1\GRHL2	0,371073888	3,25939E-05
KCNQ1OT1	10984	GRIP1	KCNQ1OT1\GRIP1	0,535051987	4,69034E-10
KCNQ1OT1	10984	GRTP1	KCNQ1OT1\GRTP1	0,353988036	8,69379E-05
KCNQ1OT1	10984	GTF2IRD2	KCNQ1OT1\GTF2IRD2	0,356017661	7,87574E-05
KCNQ1OT1	10984	HDGFRP3	KCNQ1OT1\HDGFRP3	0,292963965	0,001277546
KCNQ1OT1	10984	HIST2H2BF	KCNQ1OT1\HIST2H2BF	0,381890044	2,11177E-05
KCNQ1OT1	10984	HN1L	KCNQ1OT1\HN1L	0,223792907	0,014575649
KCNQ1OT1	10984	HPSE	KCNQ1OT1\HPSE	0,440606751	7,03151E-07
KCNQ1OT1	10984	IFT140	KCNQ1OT1\IFT140	0,45558325	2,67015E-07
KCNQ1OT1	10984	IGF1R	KCNQ1OT1\IGF1R	0,609243697	0
KCNQ1OT1	10984	IGSF3	KCNQ1OT1\IGSF3	0,446218487	4,91709E-07
KCNQ1OT1	10984	IKBIP	KCNQ1OT1\IKBIP	0,211764706	0,020939811
KCNQ1OT1	10984	IQCE	KCNQ1OT1\IQCE	0,514157527	3,56076E-09
KCNQ1OT1	10984	IQCH	KCNQ1OT1\IQCH	0,288911836	0,001498965
KCNQ1OT1	10984	IRF2BP2	KCNQ1OT1\IRF2BP2	0,67543085	0
KCNQ1OT1	10984	ITGA2	KCNQ1OT1\ITGA2	0,617825096	0

KCNQ1OT1	10984	ITGAV	KCNQ1OT1\ITGAV	0,551452785	6,01382E-12
KCNQ1OT1	10984	KAL1	KCNQ1OT1\KAL1	0,579554195	0
KCNQ1OT1	10984	KCTD1	KCNQ1OT1\KCTD1	0,432687651	1,15308E-06
KCNQ1OT1	10984	KDM4B	KCNQ1OT1\KDM4B	0,220388833	0,016177912
KCNQ1OT1	10984	KIAA0319L	KCNQ1OT1\KIAA0319L	0,619156815	0
KCNQ1OT1	10984	KLHDC10	KCNQ1OT1\KLHDC10	0,596538955	0
KCNQ1OT1	10984	LAMP1	KCNQ1OT1\LAMP1	0,267120068	0,003409812
KCNQ1OT1	10984	LARS	KCNQ1OT1\LARS	0,372674833	3,41621E-05
KCNQ1OT1	10984	LPHN3	KCNQ1OT1\LPHN3	0,413367518	2,97655E-06
KCNQ1OT1	10984	LPIN1	KCNQ1OT1\LPIN1	0,523515169	1,54763E-09
KCNQ1OT1	10984	LRRK1	KCNQ1OT1\LRRK1	0,639559892	0
KCNQ1OT1	10984	MAML3	KCNQ1OT1\MAML3	0,592308788	0
KCNQ1OT1	10984	MARVELD2	KCNQ1OT1\MARVELD2	0,516094573	3,01523E-09
KCNQ1OT1	10984	MAST4	KCNQ1OT1\MAST4	0,64270047	0
KCNQ1OT1	10984	MCTP2	KCNQ1OT1\MCTP2	0,575794046	0
KCNQ1OT1	10984	MTPAP	KCNQ1OT1\MTPAP	0,247692636	0,00673411
KCNQ1OT1	10984	MYLK4	KCNQ1OT1\MYLK4	0,506430708	6,74985E-09
KCNQ1OT1	10984	MYO10	KCNQ1OT1\MYO10	0,565852443	0
KCNQ1OT1	10984	MYO5B	KCNQ1OT1\MYO5B	0,527588663	1,04476E-09
KCNQ1OT1	10984	NAA25	KCNQ1OT1\NAA25	0,523002421	1,62357E-09
KCNQ1OT1	10984	NDRG3	KCNQ1OT1\NDRG3	0,276434981	0,002418248
KCNQ1OT1	10984	NEO1	KCNQ1OT1\NEO1	0,593106395	0
KCNQ1OT1	10984	NHLRC3	KCNQ1OT1\NHLRC3	0,713901154	0
KCNQ1OT1	10984	NPNT	KCNQ1OT1\NPNT	0,585087594	0
KCNQ1OT1	10984	NRCAM	KCNQ1OT1\NRCAM	0,346816693	0,000122649
KCNQ1OT1	10984	NXN	KCNQ1OT1\NXN	0,301324598	0,000912171
KCNQ1OT1	10984	OSBPL3	KCNQ1OT1\OSBPL3	0,600932916	0
KCNQ1OT1	10984	PARD6B	KCNQ1OT1\PARD6B	0,587936191	0
KCNQ1OT1	10984	PAX1	KCNQ1OT1\PAX1	0,388840621	1,45586E-05
KCNQ1OT1	10984	PAX9	KCNQ1OT1\PAX9	0,581882923	0
KCNQ1OT1	10984	PDGFA	KCNQ1OT1\PDGFA	0,373479561	3,27746E-05
KCNQ1OT1	10984	PDPK1	KCNQ1OT1\PDPK1	0,595136021	0
KCNQ1OT1	10984	PGAP1	KCNQ1OT1\PGAP1	0,75491383	0
KCNQ1OT1	10984	PIAS2	KCNQ1OT1\PIAS2	0,254116223	0,005405987

KCNQ1OT1	10984	PKP4	KCNQ1OT1\PKP4	0,565133172	0
KCNQ1OT1	10984	PLA2G12A	KCNQ1OT1\PLA2G12A	0,689716565	0
KCNQ1OT1	10984	POGZ	KCNQ1OT1\POGZ	0,611373024	0
KCNQ1OT1	10984	POMT2	KCNQ1OT1\POMT2	0,30524854	0,000776191
KCNQ1OT1	10984	POTEE	KCNQ1OT1\POTEE	0,412032946	3,2265E-06
KCNQ1OT1	10984	PRKAA2	KCNQ1OT1\PRKAA2	0,564111694	2,37776E-11
KCNQ1OT1	10984	PRRG4	KCNQ1OT1\PRRG4	0,544238712	1,26002E-10
KCNQ1OT1	10984	PSD3	KCNQ1OT1\PSD3	0,584204529	0
KCNQ1OT1	10984	RAB14	KCNQ1OT1\RAB14	0,537850734	3,32693E-10
KCNQ1OT1	10984	RABGAP1	KCNQ1OT1\RABGAP1	0,702763139	0
KCNQ1OT1	10984	RAI1	KCNQ1OT1\RAI1	0,499430281	1,17392E-08
KCNQ1OT1	10984	RAVER2	KCNQ1OT1\RAVER2	0,410653753	4,29959E-06
KCNQ1OT1	10984	RNF212	KCNQ1OT1\RNF212	0,401374448	7,2953E-06
KCNQ1OT1	10984	RPGRIP1L	KCNQ1OT1\RPGRIP1L	0,5293904	8,70843E-10
KCNQ1OT1	10984	RPS6KA6	KCNQ1OT1\RPS6KA6	0,61322166	1,23049E-13
KCNQ1OT1	10984	SCAMP1	KCNQ1OT1\SCAMP1	0,617070218	0
KCNQ1OT1	10984	SDR42E1	KCNQ1OT1\SDR42E1	0,396389403	9,63304E-06
KCNQ1OT1	10984	SEC61A1	KCNQ1OT1\SEC61A1	0,187131463	0,04169679
KCNQ1OT1	10984	SGPL1	KCNQ1OT1\SGPL1	0,572033898	0
KCNQ1OT1	10984	SIX4	KCNQ1OT1\SIX4	0,635329725	0
KCNQ1OT1	10984	SLC46A1	KCNQ1OT1\SLC46A1	0,18821393	0,040513298
KCNQ1OT1	10984	SMO	KCNQ1OT1\SMO	0,433620567	1,08847E-06
KCNQ1OT1	10984	SORBS2	KCNQ1OT1\SORBS2	0,309094146	0,000661236
KCNQ1OT1	10984	SORCS2	KCNQ1OT1\SORCS2	0,275452215	0,002508875
KCNQ1OT1	10984	SPATA6	KCNQ1OT1\SPATA6	0,634332716	0
KCNQ1OT1	10984	SPIN1	KCNQ1OT1\SPIN1	0,558068651	0
KCNQ1OT1	10984	ST7L	KCNQ1OT1\ST7L	0,459037174	2,12228E-07
KCNQ1OT1	10984	STEAP2	KCNQ1OT1\STEAP2	0,588463182	0
KCNQ1OT1	10984	STON2	KCNQ1OT1\STON2	0,466721265	1,26192E-07
KCNQ1OT1	10984	SYT1	KCNQ1OT1\SYT1	0,527602906	1,04328E-09
KCNQ1OT1	10984	TAB3	KCNQ1OT1\TAB3	0,649843327	0
KCNQ1OT1	10984	TDGF1	KCNQ1OT1\TDGF1	0,298259577	0,000985596
KCNQ1OT1	10984	TMEM180	KCNQ1OT1\TMEM180	0,192850021	0,035758674
KCNQ1OT1	10984	TMEM185A	KCNQ1OT1\TMEM185A	0,352442672	9,36922E-05

KCNQ1OT1	10984	TMEM30B	KCNQ1OT1\TMEM30B	0,63186868	0
KCNQ1OT1	10984	TNPO1	KCNQ1OT1\TNPO1	0,644694488	0
KCNQ1OT1	10984	TOMM20	KCNQ1OT1\TOMM20	0,479945877	5,00059E-08
KCNQ1OT1	10984	TP63	KCNQ1OT1\TP63	0,531427147	7,03464E-10
KCNQ1OT1	10984	TRMT5	KCNQ1OT1\TRMT5	0,433691782	1,08368E-06
KCNQ1OT1	10984	TRPM7	KCNQ1OT1\TRPM7	0,619249395	0
KCNQ1OT1	10984	TSPAN13	KCNQ1OT1\TSPAN13	0,557676969	0
KCNQ1OT1	10984	TTC22	KCNQ1OT1\TTC22	0,444004487	4,23818E-07
KCNQ1OT1	10984	TTC26	KCNQ1OT1\TTC26	0,44713716	4,63476E-07
KCNQ1OT1	10984	UEVLD	KCNQ1OT1\UEVLD	0,522760291	1,66053E-09
KCNQ1OT1	10984	UNC5B	KCNQ1OT1\UNC5B	0,616044723	0
KCNQ1OT1	10984	UPF1	KCNQ1OT1\UPF1	0,456466315	2,51848E-07
KCNQ1OT1	10984	USP28	KCNQ1OT1\USP28	0,703389831	0
KCNQ1OT1	10984	USP46	KCNQ1OT1\USP46	0,600633813	0
KCNQ1OT1	10984	WDR91	KCNQ1OT1\WDR91	0,516585956	2,88938E-09
KCNQ1OT1	10984	XPO7	KCNQ1OT1\XPO7	0,583741632	0
KCNQ1OT1	10984	XPR1	KCNQ1OT1\XPR1	0,615004985	0
KCNQ1OT1	10984	ZBTB41	KCNQ1OT1\ZBTB41	0,544573423	1,1826E-10
KCNQ1OT1	10984	ZNF221	KCNQ1OT1\ZNF221	0,572966814	0
KCNQ1OT1	10984	ZNF229	KCNQ1OT1\ZNF229	0,479561316	5,14007E-08
KCNQ1OT1	10984	ZNF257	KCNQ1OT1\ZNF257	0,387046005	1,60382E-05
KCNQ1OT1	10984	ZNF280B	KCNQ1OT1\ZNF280B	0,653389831	0
KCNQ1OT1	10984	ZNF449	KCNQ1OT1\ZNF449	0,593063666	0
KCNQ1OT1	10984	ZNF543	KCNQ1OT1\ZNF543	0,407904857	5,03639E-06
KCNQ1OT1	10984	ZNF562	KCNQ1OT1\ZNF562	0,564584817	0
KCNQ1OT1	10984	ZNF572	KCNQ1OT1\ZNF572	0,318038741	0,000451799
LINC00174	285908	ATP6AP2	LINC00174\ATP6AP2	0,418565731	2,70717E-06
LINC00174	285908	C15orf41	LINC00174\C15orf41	0,495933628	1,53628E-08
LINC00174	285908	C5orf15	LINC00174\C5orf15	0,324070645	0,000347218
LINC00174	285908	CLDN12	LINC00174\CLDN12	0,532630679	6,17361E-10
LINC00174	285908	COG5	LINC00174\COG5	0,413181883	3,7132E-06
LINC00174	285908	CROT	LINC00174\CROT	0,533236006	5,77307E-10
LINC00174	285908	DNAI1	LINC00174\DNAI1	0,316912958	0,000445438
LINC00174	285908	DUOX1	LINC00174\DUOX1	0,453916821	2,98042E-07

LINC00174	285908	EPS15L1	LINC00174\EPS15L1	0,18778664	0,040977053
LINC00174	285908	ESRP1	LINC00174\ESRP1	0,463616294	1,55926E-07
LINC00174	285908	FBXO2	LINC00174\FBXO2	0,1889332	0,039742595
LINC00174	285908	FBXW11	LINC00174\FBXW11	0,342209087	0,000152372
LINC00174	285908	GPHN	LINC00174\GPHN	0,315304088	0,000508205
LINC00174	285908	GTF3C4	LINC00174\GTF3C4	0,201467027	0,028167756
LINC00174	285908	KDM4D	LINC00174\KDM4D	0,340371742	0,000165996
LINC00174	285908	LAMP2	LINC00174\LAMP2	0,282609315	0,001913565
LINC00174	285908	LIMK1	LINC00174\LIMK1	0,595221478	0
LINC00174	285908	LRIG3	LINC00174\LRIG3	0,33473152	0,000215235
LINC00174	285908	MCOLN3	LINC00174\MCOLN3	0,406815268	5,36032E-06
LINC00174	285908	MOV10	LINC00174\MOV10	0,258574277	0,004627256
LINC00174	285908	MPP5	LINC00174\MPP5	0,234795613	0,010304913
LINC00174	285908	N4BP1	LINC00174\N4BP1	0,469491525	1,04297E-07
LINC00174	285908	NUDT12	LINC00174\NUDT12	0,528571429	9,46664E-10
LINC00174	285908	PABPC4L	LINC00174\PABPC4L	0,550056972	2,26637E-11
LINC00174	285908	PANK1	LINC00174\PANK1	0,295556189	0,001152023
LINC00174	285908	PLD2	LINC00174\PLD2	0,434852585	1,00841E-06
LINC00174	285908	RAB23	LINC00174\RAB23	0,228806438	0,012468329
LINC00174	285908	RNF170	LINC00174\RNF170	0,588278023	0
LINC00174	285908	SGPP2	LINC00174\SGPP2	0,330658026	0,000258894
LINC00174	285908	SHROOM2	LINC00174\SHROOM2	0,4190856	2,6251E-06
LINC00174	285908	SLC22A5	LINC00174\SLC22A5	0,452229027	3,32953E-07
LINC00174	285908	SLC30A1	LINC00174\SLC30A1	0,306217063	0,000745626
LINC00174	285908	SLC44A3	LINC00174\SLC44A3	0,214079191	0,019555993
LINC00174	285908	SPINT1	LINC00174\SPINT1	0,415304088	3,28033E-06
LINC00174	285908	SYBU	LINC00174\SYBU	0,259606894	0,004461892
LINC00174	285908	TM7SF3	LINC00174\TM7SF3	0,223451075	0,014730034
LINC00174	285908	TMEM129	LINC00174\TMEM129	0,275153112	0,002537061
LINC00174	285908	UBAC1	LINC00174\UBAC1	0,240471443	0,008567214
LINC00174	285908	WNK2	LINC00174\WNK2	0,223600627	0,014662316
LINC00174	285908	ZNF132	LINC00174\ZNF132	0,354757157	8,37487E-05
LINC00174	285908	AGPAT3	LINC00174\AGPAT3	0,27175616	0,002877901
LINC00174	285908	ALDH7A1	LINC00174\ALDH7A1	0,21526848	0,018876202

LINC00174	285908	ANK3	LINC00174\ANK3	0,608438969	0
LINC00174	285908	ANKFY1	LINC00174\ANKFY1	0,443576414	5,82333E-07
LINC00174	285908	AP2B1	LINC00174\AP2B1	0,232709016	0,011017822
LINC00174	285908	ATHL1	LINC00174\ATHL1	0,188021649	0,04072144
LINC00174	285908	ATP2C1	LINC00174\ATP2C1	0,519918815	2,15235E-09
LINC00174	285908	ATP6V1C1	LINC00174\ATP6V1C1	0,33315767	0,000231222
LINC00174	285908	ATRNL1	LINC00174\ATRNL1	0,232773524	0,010848166
LINC00174	285908	BRD1	LINC00174\BRD1	0,319790628	0,000418764
LINC00174	285908	C1orf109	LINC00174\C1orf109	0,3610668	6,14221E-05
LINC00174	285908	CAMSAP1	LINC00174\CAMSAP1	0,449992879	3,85247E-07
LINC00174	285908	CBLN3	LINC00174\CBLN3	0,383641931	1,92423E-05
LINC00174	285908	CCDC30	LINC00174\CCDC30	0,606387979	0
LINC00174	285908	CCDC8	LINC00174\CCDC8	0,250220766	0,006180194
LINC00174	285908	CDH1	LINC00174\CDH1	0,510226463	4,95145E-09
LINC00174	285908	CDS1	LINC00174\CDS1	0,47600057	6,61965E-08
LINC00174	285908	CETN3	LINC00174\CETN3	0,235863837	0,009955923
LINC00174	285908	CLINT1	LINC00174\CLINT1	0,212590799	0,020436495
LINC00174	285908	CLTC	LINC00174\CLTC	0,304379718	0,000804584
LINC00174	285908	CNTNAP3	LINC00174\CNTNAP3	0,472247543	8,61383E-08
LINC00174	285908	DDX31	LINC00174\DDX31	0,382053838	2,09353E-05
LINC00174	285908	DENND1A	LINC00174\DENND1A	0,36097422	6,1705E-05
LINC00174	285908	DHTKD1	LINC00174\DHTKD1	0,180650904	0,049403637
LINC00174	285908	DNAJC16	LINC00174\DNAJC16	0,538221051	3,17083E-10
LINC00174	285908	DTNB	LINC00174\DTNB	0,234888193	0,010274246
LINC00174	285908	DUOXA1	LINC00174\DUOXA1	0,334769527	0,00019818
LINC00174	285908	ELOVL6	LINC00174\ELOVL6	0,421692067	2,24804E-06
LINC00174	285908	EPN3	LINC00174\EPN3	0,208225324	0,02321917
LINC00174	285908	EXOC7	LINC00174\EXOC7	0,209770688	0,022198978
LINC00174	285908	FAM154B	LINC00174\FAM154B	0,473273881	5,46082E-08
LINC00174	285908	FAM199X	LINC00174\FAM199X	0,427560177	1,57853E-06
LINC00174	285908	FEM1B	LINC00174\FEM1B	0,308688221	0,000672584
LINC00174	285908	FKTN	LINC00174\FKTN	0,602136448	0
LINC00174	285908	FLVCR1	LINC00174\FLVCR1	0,434126193	1,05491E-06
LINC00174	285908	FSTL4	LINC00174\FSTL4	0,31798889	0,000452773

LINC00174	285908	GGA2	LINC00174\GGA2	0,490015667	2,39906E-08
LINC00174	285908	GLI2	LINC00174\GLI2	0,362191995	5,80805E-05
LINC00174	285908	GPR107	LINC00174\GPR107	0,372689076	3,4137E-05
LINC00174	285908	GRHL2	LINC00174\GRHL2	0,398772134	7,05724E-06
LINC00174	285908	GRIP1	LINC00174\GRIP1	0,489182453	2,55216E-08
LINC00174	285908	GRTP1	LINC00174\GRTP1	0,4062954	5,52173E-06
LINC00174	285908	GTF2I	LINC00174\GTF2I	0,263630537	0,003866943
LINC00174	285908	HIST2H2BF	LINC00174\HIST2H2BF	0,432167782	1,19065E-06
LINC00174	285908	HOMER2	LINC00174\HOMER2	0,272197693	0,002831375
LINC00174	285908	HPSE	LINC00174\HPSE	0,355618858	8,03056E-05
LINC00174	285908	IQCH	LINC00174\IQCH	0,275195841	0,002533017
LINC00174	285908	ITGA2	LINC00174\ITGA2	0,408438969	4,88447E-06
LINC00174	285908	KDM4B	LINC00174\KDM4B	0,208275174	0,023185642
LINC00174	285908	KIAA1549	LINC00174\KIAA1549	0,220851731	0,0159514
LINC00174	285908	KLHDC10	LINC00174\KLHDC10	0,346845179	0,000122483
LINC00174	285908	LARS	LINC00174\LARS	0,312149266	0,000581314
LINC00174	285908	LMBR1	LINC00174\LMBR1	0,37590799	2,8902E-05
LINC00174	285908	MAML3	LINC00174\MAML3	0,538683948	2,98294E-10
LINC00174	285908	MAP3K2	LINC00174\MAP3K2	0,346432132	0,000124906
LINC00174	285908	MARVELD2	LINC00174\MARVELD2	0,527531691	1,0507E-09
LINC00174	285908	MAST4	LINC00174\MAST4	0,496773964	1,44069E-08
LINC00174	285908	MCTP2	LINC00174\MCTP2	0,429774961	1,3791E-06
LINC00174	285908	METTL8	LINC00174\METTL8	0,560667996	0
LINC00174	285908	MTSS1L	LINC00174\MTSS1L	0,248554337	0,006540538
LINC00174	285908	MYO10	LINC00174\MYO10	0,42456915	1,89172E-06
LINC00174	285908	NCS1	LINC00174\NCS1	0,290948583	0,001383636
LINC00174	285908	NETO2	LINC00174\NETO2	0,300783364	0,000932553
LINC00174	285908	NRCAM	LINC00174\NRCAM	0,355255662	8,17403E-05
LINC00174	285908	NXN	LINC00174\NXN	0,280864549	0,002045471
LINC00174	285908	OSBPL3	LINC00174\OSBPL3	0,543312918	1,48783E-10
LINC00174	285908	PAX1	LINC00174\PAX1	0,274896738	0,002561448
LINC00174	285908	PAX9	LINC00174\PAX9	0,482502493	4,16075E-08
LINC00174	285908	PCTP	LINC00174\PCTP	0,357420595	7,35299E-05
LINC00174	285908	PDPK1	LINC00174\PDPK1	0,374747187	3,06961E-05

LINC00174	285908	PGAP1	LINC00174\PGAP1	0,519121208	2,31157E-09
LINC00174	285908	PKP4	LINC00174\PKP4	0,471122347	9,31546E-08
LINC00174	285908	PLA2G12A	LINC00174\PLA2G12A	0,555996297	0
LINC00174	285908	POLR1A	LINC00174\POLR1A	0,199594075	0,029688944
LINC00174	285908	PRRG4	LINC00174\PRRG4	0,308916109	0,000666191
LINC00174	285908	PRSS8	LINC00174\PRSS8	0,306950577	0,000723218
LINC00174	285908	PSD3	LINC00174\PSD3	0,506979063	6,45739E-09
LINC00174	285908	RAB14	LINC00174\RAB14	0,38129896	2,17884E-05
LINC00174	285908	RAD50	LINC00174\RAD50	0,387544509	1,56136E-05
LINC00174	285908	RPS6KA6	LINC00174\RPS6KA6	0,455977667	1,87566E-07
LINC00174	285908	S100A14	LINC00174\S100A14	0,189609742	0,039028956
LINC00174	285908	SCD5	LINC00174\SCD5	0,319605469	0,000422147
LINC00174	285908	SGPL1	LINC00174\SGPL1	0,497849309	1,3265E-08
LINC00174	285908	SLC46A1	LINC00174\SLC46A1	0,193398376	0,035228803
LINC00174	285908	SLC6A8	LINC00174\SLC6A8	0,381548213	2,15031E-05
LINC00174	285908	SMO	LINC00174\SMO	0,241674975	0,008233975
LINC00174	285908	SNX1	LINC00174\SNX1	0,273123487	0,002736021
LINC00174	285908	SORBS2	LINC00174\SORBS2	0,37170631	3,59051E-05
LINC00174	285908	SORCS2	LINC00174\SORCS2	0,283613445	0,001841204
LINC00174	285908	SPATA6	LINC00174\SPATA6	0,518651189	2,4102E-09
LINC00174	285908	SPIRE2	LINC00174\SPIRE2	0,362248967	5,7916E-05
LINC00174	285908	STEAP2	LINC00174\STEAP2	0,484959407	3,48119E-08
LINC00174	285908	STX6	LINC00174\STX6	0,322916963	0,000365298
LINC00174	285908	SUSD4	LINC00174\SUSD4	0,280309073	0,002089166
LINC00174	285908	TMEM180	LINC00174\TMEM180	0,388370602	1,49332E-05
LINC00174	285908	TP63	LINC00174\TP63	0,52194844	1,78985E-09
LINC00174	285908	TRPM7	LINC00174\TRPM7	0,453446802	3,07397E-07
LINC00174	285908	TTC22	LINC00174\TTC22	0,347716631	0,000106743
LINC00174	285908	UBE3C	LINC00174\UBE3C	0,425345392	1,8052E-06
LINC00174	285908	UBFD1	LINC00174\UBFD1	0,333698903	0,000225604
LINC00174	285908	UNC5B	LINC00174\UNC5B	0,384482267	1,83996E-05
LINC00174	285908	UPF1	LINC00174\UPF1	0,259557043	0,004469752
LINC00174	285908	USP40	LINC00174\USP40	0,617483264	0
LINC00174	285908	WDR91	LINC00174\WDR91	0,566628685	0

LINC00174	285908	XYLT2	LINC00174\XYLT2	0,361494089	6,01325E-05
LINC00174	285908	ZBTB41	LINC00174\ZBTB41	0,363210369	5,52038E-05
LINC00174	285908	ZNF280B	LINC00174\ZNF280B	0,431270474	1,25825E-06
LINC00174	285908	ZNF449	LINC00174\ZNF449	0,531028344	7,34005E-10
LINC00882	100302640	AP1S1	LINC00882\AP1S1	0,295710374	0,001094209
LINC00882	100302640	ATP6V0A1	LINC00882\ATP6V0A1	0,37058233	3,34499E-05
LINC00882	100302640	BAIAP2	LINC00882\BAIAP2	0,429467823	1,09446E-06
LINC00882	100302640	C14orf39	LINC00882\C14orf39	0,226467788	0,013261736
LINC00882	100302640	CBLN3	LINC00882\CBLN3	0,202852755	0,026927872
LINC00882	100302640	CDKL3	LINC00882\CDKL3	0,431174804	9,81308E-07
LINC00882	100302640	COG5	LINC00882\COG5	0,373875128	2,80953E-05
LINC00882	100302640	DHX32	LINC00882\DHX32	0,200019666	0,029182242
LINC00882	100302640	DUOXA1	LINC00882\DUOXA1	0,308398839	0,000644192
LINC00882	100302640	GLI2	LINC00882\GLI2	0,461408678	1,2825E-07
LINC00882	100302640	GRHL2	LINC00882\GRHL2	0,259857613	0,004317391
LINC00882	100302640	GRTP1	LINC00882\GRTP1	0,339318567	0,000159948
LINC00882	100302640	HIST2H2BF	LINC00882\HIST2H2BF	0,364923279	4,49464E-05
LINC00882	100302640	HOMER2	LINC00882\HOMER2	0,330117263	0,00024591
LINC00882	100302640	KCTD1	LINC00882\KCTD1	0,223389979	0,014601306
LINC00882	100302640	MAML3	LINC00882\MAML3	0,460845623	1,33446E-07
LINC00882	100302640	MARVELD2	LINC00882\MARVELD2	0,481867502	2,88264E-08
LINC00882	100302640	MRPL40	LINC00882\MRPL40	0,226992814	0,013044269
LINC00882	100302640	NAGK	LINC00882\NAGK	0,205650208	0,024849212
LINC00882	100302640	NET1	LINC00882\NET1	0,327195083	0,000281117
LINC00882	100302640	NTF4	LINC00882\NTF4	0,208077043	0,023158592
LINC00882	100302640	PLD2	LINC00882\PLD2	0,444406578	4,1258E-07
LINC00882	100302640	PRSS8	LINC00882\PRSS8	0,340907948	0,000148292
LINC00882	100302640	PSAT1	LINC00882\PSAT1	0,327512246	0,000277082
LINC00882	100302640	RABGAP1	LINC00882\RABGAP1	0,359135937	6,04569E-05
LINC00882	100302640	RNF170	LINC00882\RNF170	0,424835099	1,46735E-06
LINC00882	100302640	SEC61A1	LINC00882\SEC61A1	0,243955713	0,007502512
LINC00882	100302640	SIX1	LINC00882\SIX1	0,415819104	2,56442E-06
LINC00882	100302640	SORCS1	LINC00882\SORCS1	0,291724745	0,001286066
LINC00882	100302640	SPINT1	LINC00882\SPINT1	0,326072538	0,00029584

LINC00882	100302640	SYBU	LINC00882\SYBU	0,352404232	8,47475E-05
LINC00882	100302640	ALDH7A1	LINC00882\ALDH7A1	0,399779186	6,65778E-06
LINC00882	100302640	ANK3	LINC00882\ANK3	0,369153313	3,60594E-05
LINC00882	100302640	AP2B1	LINC00882\AP2B1	0,418937284	2,11801E-06
LINC00882	100302640	APP	LINC00882\APP	0,239408516	0,008733269
LINC00882	100302640	ATP6AP2	LINC00882\ATP6AP2	0,463033695	1,14314E-07
LINC00882	100302640	ATP6V1A	LINC00882\ATP6V1A	0,280205001	0,00202536
LINC00882	100302640	ATP6V1C1	LINC00882\ATP6V1C1	0,30305146	0,000807677
LINC00882	100302640	ATRNL1	LINC00882\ATRNL1	0,399291679	6,84841E-06
LINC00882	100302640	AUTS2	LINC00882\AUTS2	0,351816232	8,72536E-05
LINC00882	100302640	BBS4	LINC00882\BBS4	0,204046572	0,026023346
LINC00882	100302640	BRD1	LINC00882\BRD1	0,252369454	0,005623981
LINC00882	100302640	CAMSAP1	LINC00882\CAMSAP1	0,389533738	1,1939E-05
LINC00882	100302640	CCDC122	LINC00882\CCDC122	0,316062289	0,000462392
LINC00882	100302640	CCDC148	LINC00882\CCDC148	0,388221229	1,28485E-05
LINC00882	100302640	CCDC30	LINC00882\CCDC30	0,417964412	2,24871E-06
LINC00882	100302640	CCDC8	LINC00882\CCDC8	0,362097317	5,19844E-05
LINC00882	100302640	CDC42BPG	LINC00882\CDC42BPG	0,320010796	0,000388422
LINC00882	100302640	CDH1	LINC00882\CDH1	0,533835981	4,05854E-10
LINC00882	100302640	CDKL5	LINC00882\CDKL5	0,358016141	6,39844E-05
LINC00882	100302640	CDS1	LINC00882\CDS1	0,498776947	7,78103E-09
LINC00882	100302640	CETN3	LINC00882\CETN3	0,399601004	6,72687E-06
LINC00882	100302640	CLDN12	LINC00882\CLDN12	0,463967367	1,06974E-07
LINC00882	100302640	CLSTN1	LINC00882\CLSTN1	0,258509595	0,004530356
LINC00882	100302640	CLTC	LINC00882\CLTC	0,215464457	0,018605491
LINC00882	100302640	CNTNAP3	LINC00882\CNTNAP3	0,4539393	2,15966E-07
LINC00882	100302640	COX19	LINC00882\COX19	0,200468684	0,028814601
LINC00882	100302640	CRTC1	LINC00882\CRTC1	0,286238234	0,001600387
LINC00882	100302640	DCAKD	LINC00882\DCAKD	0,338317185	0,000167722
LINC00882	100302640	DDX31	LINC00882\DDX31	0,258156796	0,004587622
LINC00882	100302640	DENND1A	LINC00882\DENND1A	0,288273069	0,001476468
LINC00882	100302640	DMRTA1	LINC00882\DMRTA1	0,462089059	1,22228E-07
LINC00882	100302640	DNAL4	LINC00882\DNAL4	0,284916126	0,00168589
LINC00882	100302640	DSG2	LINC00882\DSG2	0,325203011	0,00030773

LINC00882	100302640	DUOX1	LINC00882\DUOX1	0,320196105	0,000385235
LINC00882	100302640	EFNB2	LINC00882\EFNB2	0,375329091	2,59967E-05
LINC00882	100302640	ENPEP	LINC00882\ENPEP	0,358665537	6,1916E-05
LINC00882	100302640	EPN3	LINC00882\EPN3	0,227791068	0,012719595
LINC00882	100302640	ESRP1	LINC00882\ESRP1	0,349956015	9,56447E-05
LINC00882	100302640	EYA2	LINC00882\EYA2	0,180989859	0,048858302
LINC00882	100302640	FAM154B	LINC00882\FAM154B	0,309327571	0,000619109
LINC00882	100302640	FAM160A1	LINC00882\FAM160A1	0,351969468	8,65939E-05
LINC00882	100302640	FAM199X	LINC00882\FAM199X	0,391768136	1,05285E-05
LINC00882	100302640	FAM83B	LINC00882\FAM83B	0,250470036	0,006006944
LINC00882	100302640	FKTN	LINC00882\FKTN	0,396671697	7,96435E-06
LINC00882	100302640	FNBP1L	LINC00882\FNBP1L	0,310766729	0,000582009
LINC00882	100302640	GNPDA1	LINC00882\GNPDA1	0,46440213	1,03712E-07
LINC00882	100302640	GOLM1	LINC00882\GOLM1	0,188020909	0,040587729
LINC00882	100302640	GPR107	LINC00882\GPR107	0,224754851	0,013993389
LINC00882	100302640	GPR64	LINC00882\GPR64	0,31870351	0,000411608
LINC00882	100302640	GRIP1	LINC00882\GRIP1	0,340348457	0,000152302
LINC00882	100302640	HPS5	LINC00882\HPS5	0,181788113	0,04785414
LINC00882	100302640	HPSE	LINC00882\HPSE	0,415505504	2,61395E-06
LINC00882	100302640	IGSF3	LINC00882\IGSF3	0,205204754	0,025170727
LINC00882	100302640	ITGA2	LINC00882\ITGA2	0,385734903	1,47532E-05
LINC00882	100302640	KAL1	LINC00882\KAL1	0,311340474	0,000567796
LINC00882	100302640	KDM4D	LINC00882\KDM4D	0,30118768	0,000873053
LINC00882	100302640	KIAA0319L	LINC00882\KIAA0319L	0,436121129	7,12847E-07
LINC00882	100302640	KIAA1549	LINC00882\KIAA1549	0,347753689	0,00010655
LINC00882	100302640	KLHDC10	LINC00882\KLHDC10	0,327832973	0,000273056
LINC00882	100302640	LAMP1	LINC00882\LAMP1	0,414443541	2,78848E-06
LINC00882	100302640	LAMP2	LINC00882\LAMP2	0,416955903	2,39224E-06
LINC00882	100302640	LEPREL1	LINC00882\LEPREL1	0,204670208	0,02556123
LINC00882	100302640	LIMK1	LINC00882\LIMK1	0,40811809	4,07938E-06
LINC00882	100302640	LMBR1	LINC00882\LMBR1	0,410626889	3,51127E-06
LINC00882	100302640	LRIG3	LINC00882\LRIG3	0,332084389	0,000224559
LINC00882	100302640	MAP3K2	LINC00882\MAP3K2	0,274631477	0,002506178
LINC00882	100302640	MAPK1	LINC00882\MAPK1	0,231625538	0,011256484

LINC00882	100302640	MAST4	LINC00882\MAST4	0,397623187	7,54059E-06
LINC00882	100302640	MCOLN3	LINC00882\MCOLN3	0,532827473	4,43958E-10
LINC00882	100302640	MED22	LINC00882\MED22	0,235859136	0,009814393
LINC00882	100302640	MPP5	LINC00882\MPP5	0,33572286	0,000189527
LINC00882	100302640	MYLK4	LINC00882\MYLK4	0,185483601	0,043425907
LINC00882	100302640	MYO10	LINC00882\MYO10	0,533101872	4,33262E-10
LINC00882	100302640	N4BP1	LINC00882\N4BP1	0,501079055	6,47429E-09
LINC00882	100302640	NCS1	LINC00882\NCS1	0,333449261	0,000210769
LINC00882	100302640	NEO1	LINC00882\NEO1	0,376291272	2,46899E-05
LINC00882	100302640	NETO2	LINC00882\NETO2	0,332807807	0,000217149
LINC00882	100302640	NHLRC3	LINC00882\NHLRC3	0,364880515	4,50459E-05
LINC00882	100302640	NISCH	LINC00882\NISCH	0,289064196	0,001430693
LINC00882	100302640	NPNT	LINC00882\NPNT	0,31981836	0,000391758
LINC00882	100302640	NRCAM	LINC00882\NRCAM	0,343142346	0,000133236
LINC00882	100302640	NUDT12	LINC00882\NUDT12	0,438533709	6,08823E-07
LINC00882	100302640	NUDT9	LINC00882\NUDT9	0,307245858	0,000676628
LINC00882	100302640	NXN	LINC00882\NXN	0,272037151	0,002763327
LINC00882	100302640	OAT	LINC00882\OAT	0,21219304	0,020516365
LINC00882	100302640	OSBPL3	LINC00882\OSBPL3	0,225624378	0,013617704
LINC00882	100302640	OXCT1	LINC00882\OXCT1	0,20666228	0,024131756
LINC00882	100302640	PABPC4L	LINC00882\PABPC4L	0,513876065	2,27196E-09
LINC00882	100302640	PANK1	LINC00882\PANK1	0,418573793	2,16598E-06
LINC00882	100302640	PAX1	LINC00882\PAX1	0,296644046	0,00105322
LINC00882	100302640	PAX9	LINC00882\PAX9	0,563496109	2,52623E-11
LINC00882	100302640	PDGFA	LINC00882\PDGFA	0,188783527	0,039765651
LINC00882	100302640	PGAP1	LINC00882\PGAP1	0,324440393	0,000318521
LINC00882	100302640	PIAS2	LINC00882\PIAS2	0,196669849	0,032052729
LINC00882	100302640	PKP1	LINC00882\PKP1	0,270176934	0,002962105
LINC00882	100302640	PKP4	LINC00882\PKP4	0,463439949	1,11063E-07
LINC00882	100302640	PLA2G12A	LINC00882\PLA2G12A	0,440137345	5,47852E-07
LINC00882	100302640	POGZ	LINC00882\POGZ	0,181973422	0,047623496
LINC00882	100302640	POMT2	LINC00882\POMT2	0,346613326	0,000112641
LINC00882	100302640	PRKAA2	LINC00882\PRKAA2	0,315936248	0,000464954
LINC00882	100302640	PRKAR1A	LINC00882\PRKAR1A	0,335615951	0,000190479

LINC00882	100302640	PRRC1	LINC00882\PRRC1	0,205482717	0,024969687
LINC00882	100302640	PRRG4	LINC00882\PRRG4	0,362510699	5,08942E-05
LINC00882	100302640	PSD3	LINC00882\PSD3	0,229907867	0,011892582
LINC00882	100302640	RAB14	LINC00882\RAB14	0,214862203	0,018945265
LINC00882	100302640	RAB23	LINC00882\RAB23	0,257208869	0,004744705
LINC00882	100302640	RAD50	LINC00882\RAD50	0,293105357	0,001216387
LINC00882	100302640	RANBP17	LINC00882\RANBP17	0,320174723	0,000385602
LINC00882	100302640	RASEF	LINC00882\RASEF	0,21591667	0,018353838
LINC00882	100302640	RHBDD2	LINC00882\RHBDD2	0,225891651	0,013504008
LINC00882	100302640	RNF212	LINC00882\RNF212	0,278387548	0,002172079
LINC00882	100302640	RRM2B	LINC00882\RRM2B	0,238186189	0,009093096
LINC00882	100302640	S100A14	LINC00882\S100A14	0,378226326	2,22462E-05
LINC00882	100302640	SCAMP1	LINC00882\SCAMP1	0,232969028	0,010779939
LINC00882	100302640	SCD5	LINC00882\SCD5	0,423459536	1,59949E-06
LINC00882	100302640	SDR42E1	LINC00882\SDR42E1	0,248214256	0,006491755
LINC00882	100302640	SERPINB5	LINC00882\SERPINB5	0,359282046	6,00103E-05
LINC00882	100302640	SGPL1	LINC00882\SGPL1	0,424995462	1,45264E-06
LINC00882	100302640	SHROOM2	LINC00882\SHROOM2	0,214537912	0,019130429
LINC00882	100302640	SIX4	LINC00882\SIX4	0,265679628	0,0034969
LINC00882	100302640	SLC22A5	LINC00882\SLC22A5	0,470328454	6,77048E-08
LINC00882	100302640	SLC44A3	LINC00882\SLC44A3	0,418331466	2,19853E-06
LINC00882	100302640	SLC46A1	LINC00882\SLC46A1	0,24084466	0,008326594
LINC00882	100302640	SORBS2	LINC00882\SORBS2	0,476187069	4,40606E-08
LINC00882	100302640	SPATA6	LINC00882\SPATA6	0,416652995	2,43702E-06
LINC00882	100302640	SPIN1	LINC00882\SPIN1	0,231643356	0,011250044
LINC00882	100302640	SPIRE2	LINC00882\SPIRE2	0,245748221	0,007061222
LINC00882	100302640	STEAP2	LINC00882\STEAP2	0,486436081	2,03774E-08
LINC00882	100302640	STON2	LINC00882\STON2	0,402868857	5,56129E-06
LINC00882	100302640	SUFU	LINC00882\SUFU	0,279741729	0,002061881
LINC00882	100302640	TMEM185A	LINC00882\TMEM185A	0,273398459	0,002625571
LINC00882	100302640	TMEM30B	LINC00882\TMEM30B	0,334529042	0,000200419
LINC00882	100302640	TNPO1	LINC00882\TNPO1	0,300432189	0,000900906
LINC00882	100302640	TOMM20	LINC00882\TOMM20	0,269018753	0,003092281
LINC00882	100302640	TP63	LINC00882\TP63	0,40933329	3,79413E-06

LINC00882	100302640	TRPM7	LINC00882\TRPM7	0,237523353	0,009293633
LINC00882	100302640	TTC22	LINC00882\TTC22	0,28579074	0,001628876
LINC00882	100302640	UBTD2	LINC00882\UBTD2	0,285318816	0,001659418
LINC00882	100302640	UNC5B	LINC00882\UNC5B	0,422379755	1,71104E-06
LINC00882	100302640	UPF1	LINC00882\UPF1	0,299156408	0,000949792
LINC00882	100302640	USP28	LINC00882\USP28	0,362567717	5,07455E-05
LINC00882	100302640	USP46	LINC00882\USP46	0,261442466	0,0040785
LINC00882	100302640	VMA21	LINC00882\VMA21	0,331011735	0,000235979
LINC00882	100302640	WASL	LINC00882\WASL	0,41466805	2,75069E-06
LINC00882	100302640	WDR91	LINC00882\WDR91	0,308935021	0,0006296
LINC00882	100302640	XPR1	LINC00882\XPR1	0,334382933	0,000201792
LINC00882	100302640	ZBTB41	LINC00882\ZBTB41	0,278939911	0,002126502
LINC00882	100302640	ZNF280B	LINC00882\ZNF280B	0,456205772	1,8462E-07
LINC00882	100302640	ZNF449	LINC00882\ZNF449	0,449584539	2,90984E-07
OIP5-AS1	729082	FAM45A	OIP5-AS1\FAM45A	0,272632104	0,002786264
OIP5-AS1	729082	LZIC	OIP5-AS1\LZIC	0,55758439	0
OIP5-AS1	729082	NDUFB5	OIP5-AS1\NDUFB5	0,222781655	0,015036509
OIP5-AS1	729082	NIF3L1	OIP5-AS1\NIF3L1	0,376591654	2,7892E-05
OIP5-AS1	729082	RTF1	OIP5-AS1\RTF1	0,289303518	0,001476128
OIP5-AS1	729082	URB2	OIP5-AS1\URB2	0,208624128	0,02295211
OIP5-AS1	729082	WDR3	OIP5-AS1\WDR3	0,187679818	0,041093685
OIP5-AS1	729082	ARL6IP1	OIP5-AS1\ARL6IP1	0,283706025	0,00183466
OIP5-AS1	729082	ATPAF1	OIP5-AS1\ATPAF1	0,328087167	0,000290536
OIP5-AS1	729082	CSRP2BP	OIP5-AS1\CSRP2BP	0,274013673	0,002647082
OIP5-AS1	729082	EIF2AK1	OIP5-AS1\EIF2AK1	0,273102122	0,002738188
OIP5-AS1	729082	GGCT	OIP5-AS1\GGCT	0,244979348	0,00737734
OIP5-AS1	729082	GINS3	OIP5-AS1\GINS3	0,238342117	0,009186118
OIP5-AS1	729082	GTF2H4	OIP5-AS1\GTF2H4	0,282217633	0,001942484
OIP5-AS1	729082	HIST1H2BC	OIP5-AS1\HIST1H2BC	0,249964393	0,006234454
OIP5-AS1	729082	LRPPRC	OIP5-AS1\LRPPRC	0,284959407	0,001748113
OIP5-AS1	729082	PMPCB	OIP5-AS1\PMPCB	0,264292836	0,003776161
OIP5-AS1	729082	PNPT1	OIP5-AS1\PNPT1	0,19193847	0,036654388
OIP5-AS1	729082	PSMC2	OIP5-AS1\PSMC2	0,26249822	0,004026705
OIP5-AS1	729082	PSMD12	OIP5-AS1\PSMD12	0,420581114	2,40199E-06

OIP5-AS1	729082	SCCPDH	OIP5-AS1\SCCPDH	0,266137302	0,003533357
OIP5-AS1	729082	TRIM14	OIP5-AS1\TRIM14	0,196218487	0,03260741
OIP5-AS1	729082	VANGL1	OIP5-AS1\VANGL1	0,307641362	0,000702684
PARD6G-AS1	100130522	ACAD10	PARD6G-AS1\ACAD10	0,264321322	0,0037723
PARD6G-AS1	100130522	AFG3L2	PARD6G-AS1\AFG3L2	0,408859137	4,76801E-06
PARD6G-AS1	100130522	ANKFY1	PARD6G-AS1\ANKFY1	0,456865119	2,45271E-07
PARD6G-AS1	100130522	AP1S1	PARD6G-AS1\AP1S1	0,288513032	0,001522548
PARD6G-AS1	100130522	APOBEC2	PARD6G-AS1\APOBEC2	0,361489542	5,3627E-05
PARD6G-AS1	100130522	APTX	PARD6G-AS1\APTX	0,338477425	0,000181225
PARD6G-AS1	100130522	ARV1	PARD6G-AS1\ARV1	0,298597066	0,001019209
PARD6G-AS1	100130522	ATP2C1	PARD6G-AS1\ATP2C1	0,319114086	0,000431247
PARD6G-AS1	100130522	ATP6V1C1	PARD6G-AS1\ATP6V1C1	0,203190429	0,026827283
PARD6G-AS1	100130522	C15orf41	PARD6G-AS1\C15orf41	0,246403646	0,007033187
PARD6G-AS1	100130522	CAMSAP1	PARD6G-AS1\CAMSAP1	0,477061672	6,14097E-08
PARD6G-AS1	100130522	CBLN3	PARD6G-AS1\CBLN3	0,474975075	7,11586E-08
PARD6G-AS1	100130522	CBS	PARD6G-AS1\CBS	0,261835921	0,004122876
PARD6G-AS1	100130522	CETN3	PARD6G-AS1\CETN3	0,315902293	0,000495338
PARD6G-AS1	100130522	CLDN12	PARD6G-AS1\CLDN12	0,245876656	0,007158824
PARD6G-AS1	100130522	COG5	PARD6G-AS1\COG5	0,303040877	0,00085021
PARD6G-AS1	100130522	COG8	PARD6G-AS1\COG8	0,237416322	0,009467307
PARD6G-AS1	100130522	CROT	PARD6G-AS1\CROT	0,274839766	0,002566896
PARD6G-AS1	100130522	CSRP2BP	PARD6G-AS1\CSRP2BP	0,302357214	0,000874414
PARD6G-AS1	100130522	CUX1	PARD6G-AS1\CUX1	0,378023074	2,58839E-05
PARD6G-AS1	100130522	DNAI1	PARD6G-AS1\DNAI1	0,316457437	0,000454444
PARD6G-AS1	100130522	DNAJC16	PARD6G-AS1\DNAJC16	0,239766415	0,008767921
PARD6G-AS1	100130522	DUOX1	PARD6G-AS1\DUOX1	0,504571998	7,83483E-09
PARD6G-AS1	100130522	EIF2AK1	PARD6G-AS1\EIF2AK1	0,209884632	0,022125308
PARD6G-AS1	100130522	ELOVL6	PARD6G-AS1\ELOVL6	0,302200541	0,000880049
PARD6G-AS1	100130522	ENPEP	PARD6G-AS1\ENPEP	0,308289417	0,000683908
PARD6G-AS1	100130522	FAM120A	PARD6G-AS1\FAM120A	0,431498362	1,24074E-06
PARD6G-AS1	100130522	FAM163A	PARD6G-AS1\FAM163A	0,453646204	3,03394E-07
PARD6G-AS1	100130522	FAM199X	PARD6G-AS1\FAM199X	0,263260219	0,003918553
PARD6G-AS1	100130522	FBXO22	PARD6G-AS1\FBXO22	0,261764706	0,004133339
PARD6G-AS1	100130522	FKTN	PARD6G-AS1\FKTN	0,272097992	0,002841821

PARD6G-AS1	100130522	FLVCR1	PARD6G-AS1\FLVCR1	0,363915397	5,3291E-05
PARD6G-AS1	100130522	FSTL4	PARD6G-AS1\FSTL4	0,425089019	1,83335E-06
PARD6G-AS1	100130522	GGA1	PARD6G-AS1\GGA1	0,312348668	0,000576421
PARD6G-AS1	100130522	GGA2	PARD6G-AS1\GGA2	0,505512035	7,2674E-09
PARD6G-AS1	100130522	GGCT	PARD6G-AS1\GGCT	0,205668708	0,024995469
PARD6G-AS1	100130522	GINS3	PARD6G-AS1\GINS3	0,214356929	0,019395373
PARD6G-AS1	100130522	GLI2	PARD6G-AS1\GLI2	0,544616152	1,1729E-10
PARD6G-AS1	100130522	HDAC4	PARD6G-AS1\HDAC4	0,299202393	0,000994506
PARD6G-AS1	100130522	HIST1H2BC	PARD6G-AS1\HIST1H2BC	0,260319043	0,004350957
PARD6G-AS1	100130522	HIST2H2BF	PARD6G-AS1\HIST2H2BF	0,410319043	4,38351E-06
PARD6G-AS1	100130522	HN1L	PARD6G-AS1\HN1L	0,263651901	0,003863984
PARD6G-AS1	100130522	HOMER2	PARD6G-AS1\HOMER2	0,374077767	3,17778E-05
PARD6G-AS1	100130522	KDM4D	PARD6G-AS1\KDM4D	0,271485543	0,002906757
PARD6G-AS1	100130522	KIAA1549	PARD6G-AS1\KIAA1549	0,477054551	6,14407E-08
PARD6G-AS1	100130522	LARS	PARD6G-AS1\LARS	0,245584675	0,007229289
PARD6G-AS1	100130522	LIMK1	PARD6G-AS1\LIMK1	0,317832218	0,000455848
PARD6G-AS1	100130522	MAML3	PARD6G-AS1\MAML3	0,313381285	0,000551681
PARD6G-AS1	100130522	MAP3K2	PARD6G-AS1\MAP3K2	0,228372027	0,012639713
PARD6G-AS1	100130522	MCOLN3	PARD6G-AS1\MCOLN3	0,508702464	5,61257E-09
PARD6G-AS1	100130522	MPP5	PARD6G-AS1\MPP5	0,407206951	5,24167E-06
PARD6G-AS1	100130522	MTSS1L	PARD6G-AS1\MTSS1L	0,260254949	0,004360839
PARD6G-AS1	100130522	N4BP1	PARD6G-AS1\N4BP1	0,266756872	0,003455009
PARD6G-AS1	100130522	NAGK	PARD6G-AS1\NAGK	0,47290272	8,22878E-08
PARD6G-AS1	100130522	NDUFA4	PARD6G-AS1\NDUFA4	0,204920951	0,025536526
PARD6G-AS1	100130522	NETO2	PARD6G-AS1\NETO2	0,497016095	1,4142E-08
PARD6G-AS1	100130522	NPTX1	PARD6G-AS1\NPTX1	0,246581684	0,006991187
PARD6G-AS1	100130522	NRCAM	PARD6G-AS1\NRCAM	0,462961117	1,63002E-07
PARD6G-AS1	100130522	NUDT12	PARD6G-AS1\NUDT12	0,27967526	0,002140058
PARD6G-AS1	100130522	NVL	PARD6G-AS1\NVL	0,262697621	0,003998147
PARD6G-AS1	100130522	OSBPL3	PARD6G-AS1\OSBPL3	0,327125766	0,000303263
PARD6G-AS1	100130522	OXCT1	PARD6G-AS1\OXCT1	0,540820396	2,21214E-10
PARD6G-AS1	100130522	PASK	PARD6G-AS1\PASK	0,45820396	2,24367E-07
PARD6G-AS1	100130522	PCBD2	PARD6G-AS1\PCBD2	0,255590372	0,00513641
PARD6G-AS1	100130522	PFN4	PARD6G-AS1\PFN4	0,478793047	3,63023E-08

PARD6G-AS1	100130522	PLA2G12A	PARD6G-AS1\PLA2G12A	0,257662726	0,004777775
PARD6G-AS1	100130522	PLEK2	PARD6G-AS1\PLEK2	0,565774106	0
PARD6G-AS1	100130522	PRSS8	PARD6G-AS1\PRSS8	0,59432417	0
PARD6G-AS1	100130522	RFESD	PARD6G-AS1\RFESD	0,307121493	0,000718087
PARD6G-AS1	100130522	RRAGB	PARD6G-AS1\RRAGB	0,377360775	2,67955E-05
PARD6G-AS1	100130522	SCCPDH	PARD6G-AS1\SCCPDH	0,209435978	0,022416605
PARD6G-AS1	100130522	SCD5	PARD6G-AS1\SCD5	0,297735365	0,001055344
PARD6G-AS1	100130522	SLC44A3	PARD6G-AS1\SLC44A3	0,509813417	5,12319E-09
PARD6G-AS1	100130522	SLC6A8	PARD6G-AS1\SLC6A8	0,538947443	2,87948E-10
PARD6G-AS1	100130522	SNX1	PARD6G-AS1\SNX1	0,347101552	0,000121002
PARD6G-AS1	100130522	SPIRE2	PARD6G-AS1\SPIRE2	0,508453212	5,72811E-09
PARD6G-AS1	100130522	SPRY3	PARD6G-AS1\SPRY3	0,225167355	0,013969059
PARD6G-AS1	100130522	STX6	PARD6G-AS1\STX6	0,545691497	9,4187E-11
PARD6G-AS1	100130522	TRIM14	PARD6G-AS1\TRIM14	0,207655605	0,023605315
PARD6G-AS1	100130522	TRIM61	PARD6G-AS1\TRIM61	0,246255742	0,00694053
PARD6G-AS1	100130522	TRIM7	PARD6G-AS1\TRIM7	0,423728814	1,98982E-06
PARD6G-AS1	100130522	TTC22	PARD6G-AS1\TTC22	0,33891787	0,000163018
PARD6G-AS1	100130522	UBE3C	PARD6G-AS1\UBE3C	0,228678251	0,012518689
PARD6G-AS1	100130522	UBFD1	PARD6G-AS1\UBFD1	0,21574562	0,018609278
PARD6G-AS1	100130522	VAC14	PARD6G-AS1\VAC14	0,475957841	6,63965E-08
PARD6G-AS1	100130522	VANGL1	PARD6G-AS1\VANGL1	0,266201396	0,003525178
PARD6G-AS1	100130522	VMA21	PARD6G-AS1\VMA21	0,310981342	0,000610752
PARD6G-AS1	100130522	WDR61	PARD6G-AS1\WDR61	0,201666429	0,028009793
PARD6G-AS1	100130522	WNK2	PARD6G-AS1\WNK2	0,494487965	1,71483E-08
PARD6G-AS1	100130522	WWOX	PARD6G-AS1\WWOX	0,255490671	0,005154255
PARD6G-AS1	100130522	XYLT2	PARD6G-AS1\XYLT2	0,434318473	1,04241E-06
PARD6G-AS1	100130522	ZNF132	PARD6G-AS1\ZNF132	0,28778664	0,001566374
PARD6G-AS1	100130522	ZNF544	PARD6G-AS1\ZNF544	0,391240564	1,27804E-05
PVT1	5820	ARV1	PVT1\ARV1	0,294366899	0,001208141
PVT1	5820	ATP6V0D1	PVT1\ATP6V0D1	0,232025353	0,011260657
PVT1	5820	CBS	PVT1\CBS	0,208809286	0,022829018
PVT1	5820	CCL25	PVT1\CCL25	0,392778806	1,17507E-05
PVT1	5820	ESRP1	PVT1\ESRP1	0,215738499	0,018613238
PVT1	5820	EYA2	PVT1\EYA2	0,268095713	0,003291027

PVT1	5820	FBXO2	PVT1\FBXO2	0,493284432	1,87818E-08
PVT1	5820	GPHN	PVT1\GPHN	0,236917818	0,009621842
PVT1	5820	MFSD3	PVT1\MFSD3	0,571855861	0
PVT1	5820	MRPL40	PVT1\MRPL40	0,508289417	5,80521E-09
PVT1	5820	NAGK	PVT1\NAGK	0,510596781	4,80198E-09
PVT1	5820	NVL	PVT1\NVL	0,354443811	8,50346E-05
PVT1	5820	PFN4	PVT1\PFN4	0,346888525	0,000111142
PVT1	5820	PLEK2	PVT1\PLEK2	0,350470019	0,000103027
PVT1	5820	PSMC2	PVT1\PSMC2	0,182281726	0,047360682
PVT1	5820	RASL10A	PVT1\RASL10A	0,260689825	0,004190431
PVT1	5820	RFESD	PVT1\RFESD	0,244345535	0,007535238
PVT1	5820	SCCPDH	PVT1\SCCPDH	0,30710725	0,000718513
PVT1	5820	SHROOM2	PVT1\SHROOM2	0,329995727	0,000266724
PVT1	5820	SNUPN	PVT1\SNUPN	0,218216778	0,017278546
PVT1	5820	SPINT1	PVT1\SPINT1	0,189096995	0,039568821
PVT1	5820	TMEM129	PVT1\TMEM129	0,436390828	9,16309E-07
PVT1	5820	UBAC1	PVT1\UBAC1	0,469049993	1,07526E-07
PVT1	5820	AGPAT3	PVT1\AGPAT3	0,378307933	2,55009E-05
PVT1	5820	ATHL1	PVT1\ATHL1	0,439061387	7,7512E-07
PVT1	5820	CALML3	PVT1\CALML3	0,556893605	0
PVT1	5820	DENND1A	PVT1\DENND1A	0,407427717	5,17589E-06
PVT1	5820	EIF3B	PVT1\EIF3B	0,523515169	1,54763E-09
PVT1	5820	FAHD1	PVT1\FAHD1	0,477453354	5,97274E-08
PVT1	5820	FBXL16	PVT1\FBXL16	0,327510326	0,000298111
PVT1	5820	GGA2	PVT1\GGA2	0,181006979	0,048951439
PVT1	5820	GGCT	PVT1\GGCT	0,582972511	0
PVT1	5820	GPS1	PVT1\GPS1	0,430237858	1,34056E-06
PVT1	5820	IFI44	PVT1\IFI44	0,247721122	0,00672763
PVT1	5820	LMOD3	PVT1\LMOD3	0,343681562	0,000129822
PVT1	5820	NCS1	PVT1\NCS1	0,307527418	0,000706033
PVT1	5820	NDUFA4	PVT1\NDUFA4	0,428023074	1,53471E-06
PVT1	5820	NETO2	PVT1\NETO2	0,206850876	0,024160138
PVT1	5820	PCBD2	PVT1\PCBD2	0,298739496	0,001013346
PVT1	5820	PCCB	PVT1\PCCB	0,429475858	1,40456E-06

PVT1	5820	PKP1	PVT1\PKP1	0,351182168	9,95612E-05
PVT1	5820	PRMT5	PVT1\PRMT5	0,213659023	0,019801169
PVT1	5820	PRSS8	PVT1\PRSS8	0,342992451	0,000146886
PVT1	5820	S100A14	PVT1\S100A14	0,347521721	0,00011861
PVT1	5820	SEC61A1	PVT1\SEC61A1	0,195000712	0,033718352
PVT1	5820	SLCO5A1	PVT1\SLCO5A1	0,263075061	0,00394459
PVT1	5820	SUSD4	PVT1\SUSD4	0,555604615	0
PVT1	5820	TMEM180	PVT1\TMEM180	0,547585814	5,91211E-11
PVT1	5820	XYLT2	PVT1\XYLT2	0,187950434	0,040798757
PVT1	5820	ZC3HC1	PVT1\ZC3HC1	0,266358069	0,003505258
TTC28-AS1	284900	AP1S1	TTC28-AS1\AP1S1	0,260347529	0,004346571
TTC28-AS1	284900	DNAL4	TTC28-AS1\DNAL4	0,302556616	0,00086729
TTC28-AS1	284900	RPL37	TTC28-AS1\RPL37	0,682238997	0
TTC28-AS1	284900	TOMM40	TTC28-AS1\TOMM40	0,535265632	4,57391E-10
TTC28-AS1	284900	TXNL1	TTC28-AS1\TXNL1	0,363822817	5,35386E-05
TTC28-AS1	284900	CCDC73	TTC28-AS1\CCDC73	0,306694203	0,000730979
TTC28-AS1	284900	DNAJC19	TTC28-AS1\DNAJC19	0,211515454	0,021093759
TTC28-AS1	284900	EXOSC10	TTC28-AS1\EXOSC10	0,293206096	0,001265313
TTC28-AS1	284900	GGCT	TTC28-AS1\GGCT	0,322432702	0,000373144
TTC28-AS1	284900	GPR35	TTC28-AS1\GPR35	0,286675687	0,001635628
TTC28-AS1	284900	GTF2H4	TTC28-AS1\GTF2H4	0,493255946	1,88222E-08
TTC28-AS1	284900	IDH3G	TTC28-AS1\IDH3G	0,608880501	0
TTC28-AS1	284900	LRRC27	TTC28-AS1\LRRC27	0,412754593	3,80666E-06
TTC28-AS1	284900	MED22	TTC28-AS1\MED22	0,188363481	0,040352028
TTC28-AS1	284900	NDUFA4	TTC28-AS1\NDUFA4	0,459535679	2,05267E-07
TTC28-AS1	284900	RAB40C	TTC28-AS1\RAB40C	0,238513032	0,009135021
TTC28-AS1	284900	RHBDD2	TTC28-AS1\RHBDD2	0,202421308	0,027418618
TTC28-AS1	284900	RP9	TTC28-AS1\RP9	0,643547928	0
TTC28-AS1	284900	SARS2	TTC28-AS1\SARS2	0,41173622	4,03851E-06
TTC28-AS1	284900	SLC15A1	TTC28-AS1\SLC15A1	0,200931585	0,02843972
