# Supplementary material for: LINC00174 is a novel prognostic factor in thymic epithelial tumors involved in cell migration and lipid metabolism
Source: Cell Death Dis. 2020 Nov 7;11(11):959. doi: 10.1038/s41419-020-03171-9 (PMC7648846; doi:10.1038/s41419-020-03171-9)
Supplement: Supplementary file 16 — Supplementary Table 3_Sheet 2 [file 41419_2020_3171_MOESM16_ESM.pdf]

Prognostic value of the 12 lncRNAs previosly selected

gene	HR[CI95%]	pval	logrank(zscore)
LINC00174	1.8876[1.0089-3.5313]	0,046828497	0,018416651
FAM95B1	1.4962[0.98443-2.2741]	0,0592259	0,065966222
FAM66E	1.7783[0.98543-3.0812]	0,06009544	0,082391672
PVT1	2.3066[0.9863-4.1656]	0,055793092	0,142871792
OIP5-AS1	0.32754[0.073344-1.4627]	0,143773065	0,243335218
TTC28-AS1	0.41122[0.15454-1.0943]	0,075139194	0,436700297
CTBP1-AS2	0.65722[0.12242-3.5283]	0,624461076	0,630981844
KCNQ1OT1	1.0063[0.5563-1.8202]	0,983516914	0,733157067
CDKN2B-AS1	0.95039[0.49894-1.8103]	0,877003579	0,773751283
PARD6G-AS1	0.93359[0.53872-1.6179]	0,806484951	0,858826501
LINC00882	1.2854[0.76608-2.1569]	0,341661748	0,882077273
C17orf102	4.6941e-46[0-Inf]	0,996954836	0,934250841
