# Supplementary material for: LINC00174 is a novel prognostic factor in thymic epithelial tumors involved in cell migration and lipid metabolism
Source: Cell Death Dis. 2020 Nov 7;11(11):959. doi: 10.1038/s41419-020-03171-9 (PMC7648846; doi:10.1038/s41419-020-03171-9)
Supplement: Supplementary file 17 — Supplementary Table 3_Sheet 3 [file 41419_2020_3171_MOESM17_ESM.pdf]

List of 128 genes positively correlated to LINC00174 in IRE cohort and validated in TCGA cohort					
**lncRNAs**	**lncRNAs ID**	**mRNAs**	**lncRNA\gene**	**R Spearman**	**pval**
LINC00174	285908	USP40	LINC00174\USP40	0,617483264	0
LINC00174	285908	ANK3	LINC00174\ANK3	0,608438969	0
LINC00174	285908	CCDC30	LINC00174\CCDC30	0,606387979	0
LINC00174	285908	FKTN	LINC00174\FKTN	0,602136448	0
LINC00174	285908	LIMK1	LINC00174\LIMK1	0,595221478	0
LINC00174	285908	RNF170	LINC00174\RNF170	0,588278023	0
LINC00174	285908	WDR91	LINC00174\WDR91	0,566628685	0
LINC00174	285908	METTL8	LINC00174\METTL8	0,560667996	0
LINC00174	285908	PLA2G12A	LINC00174\PLA2G12A	0,555996297	0
LINC00174	285908	PABPC4L	LINC00174\PABPC4L	0,550056972	2,26637E-11
LINC00174	285908	OSBPL3	LINC00174\OSBPL3	0,543312918	1,48783E-10
LINC00174	285908	MAML3	LINC00174\MAML3	0,538683948	2,98294E-10
LINC00174	285908	DNAJC16	LINC00174\DNAJC16	0,538221051	3,17083E-10
LINC00174	285908	CROT	LINC00174\CROT	0,533236006	5,77307E-10
LINC00174	285908	CLDN12	LINC00174\CLDN12	0,532630679	6,17361E-10
LINC00174	285908	ZNF449	LINC00174\ZNF449	0,531028344	7,34005E-10
LINC00174	285908	NUDT12	LINC00174\NUDT12	0,528571429	9,46664E-10
LINC00174	285908	MARVELD2	LINC00174\MARVELD2	0,527531691	1,0507E-09
LINC00174	285908	TP63	LINC00174\TP63	0,52194844	1,78985E-09
LINC00174	285908	ATP2C1	LINC00174\ATP2C1	0,519918815	2,15235E-09
LINC00174	285908	PGAP1	LINC00174\PGAP1	0,519121208	2,31157E-09
LINC00174	285908	SPATA6	LINC00174\SPATA6	0,518651189	2,4102E-09
LINC00174	285908	CDH1	LINC00174\CDH1	0,510226463	4,95145E-09
LINC00174	285908	PSD3	LINC00174\PSD3	0,506979063	6,45739E-09
LINC00174	285908	SGPL1	LINC00174\SGPL1	0,497849309	1,3265E-08
LINC00174	285908	MAST4	LINC00174\MAST4	0,496773964	1,44069E-08
LINC00174	285908	C15orf41	LINC00174\C15orf41	0,495933628	1,53628E-08
LINC00174	285908	GGA2	LINC00174\GGA2	0,490015667	2,39906E-08
LINC00174	285908	GRIP1	LINC00174\GRIP1	0,489182453	2,55216E-08
LINC00174	285908	STEAP2	LINC00174\STEAP2	0,484959407	3,48119E-08
LINC00174	285908	PAX9	LINC00174\PAX9	0,482502493	4,16075E-08
LINC00174	285908	CDS1	LINC00174\CDS1	0,47600057	6,61965E-08

LINC00174	285908	FAM154B	LINC00174\FAM154B	0,473273881	5,46082E-08
LINC00174	285908	CNTNAP3	LINC00174\CNTNAP3	0,472247543	8,61383E-08
LINC00174	285908	PKP4	LINC00174\PKP4	0,471122347	9,31546E-08
LINC00174	285908	N4BP1	LINC00174\N4BP1	0,469491525	1,04297E-07
LINC00174	285908	ESRP1	LINC00174\ESRP1	0,463616294	1,55926E-07
LINC00174	285908	RPS6KA6	LINC00174\RPS6KA6	0,455977667	1,87566E-07
LINC00174	285908	DUOX1	LINC00174\DUOX1	0,453916821	2,98042E-07
LINC00174	285908	TRPM7	LINC00174\TRPM7	0,453446802	3,07397E-07
LINC00174	285908	SLC22A5	LINC00174\SLC22A5	0,452229027	3,32953E-07
LINC00174	285908	CAMSAP1	LINC00174\CAMSAP1	0,449992879	3,85247E-07
LINC00174	285908	ANKFY1	LINC00174\ANKFY1	0,443576414	5,82333E-07
LINC00174	285908	PLD2	LINC00174\PLD2	0,434852585	1,00841E-06
LINC00174	285908	FLVCR1	LINC00174\FLVCR1	0,434126193	1,05491E-06
LINC00174	285908	HIST2H2BF	LINC00174\HIST2H2BF	0,432167782	1,19065E-06
LINC00174	285908	ZNF280B	LINC00174\ZNF280B	0,431270474	1,25825E-06
LINC00174	285908	MCTP2	LINC00174\MCTP2	0,429774961	1,3791E-06
LINC00174	285908	FAM199X	LINC00174\FAM199X	0,427560177	1,57853E-06
LINC00174	285908	UBE3C	LINC00174\UBE3C	0,425345392	1,8052E-06
LINC00174	285908	MYO10	LINC00174\MYO10	0,42456915	1,89172E-06
LINC00174	285908	ELOVL6	LINC00174\ELOVL6	0,421692067	2,24804E-06
LINC00174	285908	SHROOM2	LINC00174\SHROOM2	0,4190856	2,6251E-06
LINC00174	285908	ATP6AP2	LINC00174\ATP6AP2	0,418565731	2,70717E-06
LINC00174	285908	SPINT1	LINC00174\SPINT1	0,415304088	3,28033E-06
LINC00174	285908	COG5	LINC00174\COG5	0,413181883	3,7132E-06
LINC00174	285908	ITGA2	LINC00174\ITGA2	0,408438969	4,88447E-06
LINC00174	285908	MCOLN3	LINC00174\MCOLN3	0,406815268	5,36032E-06
LINC00174	285908	GRTP1	LINC00174\GRTP1	0,4062954	5,52173E-06
LINC00174	285908	GRHL2	LINC00174\GRHL2	0,398772134	7,05724E-06
LINC00174	285908	TMEM180	LINC00174\TMEM180	0,388370602	1,49332E-05
LINC00174	285908	RAD50	LINC00174\RAD50	0,387544509	1,56136E-05
LINC00174	285908	UNC5B	LINC00174\UNC5B	0,384482267	1,83996E-05
LINC00174	285908	CBLN3	LINC00174\CBLN3	0,383641931	1,92423E-05
LINC00174	285908	DDX31	LINC00174\DDX31	0,382053838	2,09353E-05
LINC00174	285908	SLC6A8	LINC00174\SLC6A8	0,381548213	2,15031E-05

LINC00174	285908	RAB14	LINC00174\RAB14	0,38129896	2,17884E-05
LINC00174	285908	LMBR1	LINC00174\LMBR1	0,37590799	2,8902E-05
LINC00174	285908	PDPK1	LINC00174\PDPK1	0,374747187	3,06961E-05
LINC00174	285908	GPR107	LINC00174\GPR107	0,372689076	3,4137E-05
LINC00174	285908	SORBS2	LINC00174\SORBS2	0,37170631	3,59051E-05
LINC00174	285908	ZBTB41	LINC00174\ZBTB41	0,363210369	5,52038E-05
LINC00174	285908	SPIRE2	LINC00174\SPIRE2	0,362248967	5,7916E-05
LINC00174	285908	GLI2	LINC00174\GLI2	0,362191995	5,80805E-05
LINC00174	285908	XYLT2	LINC00174\XYLT2	0,361494089	6,01325E-05
LINC00174	285908	C1orf109	LINC00174\C1orf109	0,3610668	6,14221E-05
LINC00174	285908	DENND1A	LINC00174\DENND1A	0,36097422	6,1705E-05
LINC00174	285908	PCTP	LINC00174\PCTP	0,357420595	7,35299E-05
LINC00174	285908	HPSE	LINC00174\HPSE	0,355618858	8,03056E-05
LINC00174	285908	NRCAM	LINC00174\NRCAM	0,355255662	8,17403E-05
LINC00174	285908	ZNF132	LINC00174\ZNF132	0,354757157	8,37487E-05
LINC00174	285908	TTC22	LINC00174\TTC22	0,347716631	0,000106743
LINC00174	285908	KLHDC10	LINC00174\KLHDC10	0,346845179	0,000122483
LINC00174	285908	MAP3K2	LINC00174\MAP3K2	0,346432132	0,000124906
LINC00174	285908	FBXW11	LINC00174\FBXW11	0,342209087	0,000152372
LINC00174	285908	KDM4D	LINC00174\KDM4D	0,340371742	0,000165996
LINC00174	285908	DUOXA1	LINC00174\DUOXA1	0,334769527	0,00019818
LINC00174	285908	LRIG3	LINC00174\LRIG3	0,33473152	0,000215235
LINC00174	285908	UBFD1	LINC00174\UBFD1	0,333698903	0,000225604
LINC00174	285908	ATP6V1C1	LINC00174\ATP6V1C1	0,33315767	0,000231222
LINC00174	285908	SGPP2	LINC00174\SGPP2	0,330658026	0,000258894
LINC00174	285908	C5orf15	LINC00174\C5orf15	0,324070645	0,000347218
LINC00174	285908	STX6	LINC00174\STX6	0,322916963	0,000365298
LINC00174	285908	BRD1	LINC00174\BRD1	0,319790628	0,000418764
LINC00174	285908	SCD5	LINC00174\SCD5	0,319605469	0,000422147
LINC00174	285908	FSTL4	LINC00174\FSTL4	0,31798889	0,000452773
LINC00174	285908	DNAI1	LINC00174\DNAI1	0,316912958	0,000445438
LINC00174	285908	GPHN	LINC00174\GPHN	0,315304088	0,000508205
LINC00174	285908	LARS	LINC00174\LARS	0,312149266	0,000581314
LINC00174	285908	PRRG4	LINC00174\PRRG4	0,308916109	0,000666191

LINC00174	285908	FEM1B	LINC00174\FEM1B	0,308688221	0,000672584
LINC00174	285908	PRSS8	LINC00174\PRSS8	0,306950577	0,000723218
LINC00174	285908	SLC30A1	LINC00174\SLC30A1	0,306217063	0,000745626
LINC00174	285908	CLTC	LINC00174\CLTC	0,304379718	0,000804584
LINC00174	285908	NETO2	LINC00174\NETO2	0,300783364	0,000932553
LINC00174	285908	PANK1	LINC00174\PANK1	0,295556189	0,001152023
LINC00174	285908	NCS1	LINC00174\NCS1	0,290948583	0,001383636
LINC00174	285908	SORCS2	LINC00174\SORCS2	0,283613445	0,001841204
LINC00174	285908	LAMP2	LINC00174\LAMP2	0,282609315	0,001913565
LINC00174	285908	NXN	LINC00174\NXN	0,280864549	0,002045471
LINC00174	285908	SUSD4	LINC00174\SUSD4	0,280309073	0,002089166
LINC00174	285908	IQCH	LINC00174\IQCH	0,275195841	0,002533017
LINC00174	285908	TMEM129	LINC00174\TMEM129	0,275153112	0,002537061
LINC00174	285908	PAX1	LINC00174\PAX1	0,274896738	0,002561448
LINC00174	285908	SNX1	LINC00174\SNX1	0,273123487	0,002736021
LINC00174	285908	HOMER2	LINC00174\HOMER2	0,272197693	0,002831375
LINC00174	285908	AGPAT3	LINC00174\AGPAT3	0,27175616	0,002877901
LINC00174	285908	GTF2I	LINC00174\GTF2I	0,263630537	0,003866943
LINC00174	285908	SYBU	LINC00174\SYBU	0,259606894	0,004461892
LINC00174	285908	UPF1	LINC00174\UPF1	0,259557043	0,004469752
LINC00174	285908	MOV10	LINC00174\MOV10	0,258574277	0,004627256
LINC00174	285908	CCDC8	LINC00174\CCDC8	0,250220766	0,006180194
LINC00174	285908	MTSS1L	LINC00174\MTSS1L	0,248554337	0,006540538
LINC00174	285908	SMO	LINC00174\SMO	0,241674975	0,008233975
LINC00174	285908	UBAC1	LINC00174\UBAC1	0,240471443	0,008567214
LINC00174	285908	CETN3	LINC00174\CETN3	0,235863837	0,009955923
LINC00174	285908	DTNB	LINC00174\DTNB	0,234888193	0,010274246
LINC00174	285908	MPP5	LINC00174\MPP5	0,234795613	0,010304913
LINC00174	285908	ATRNL1	LINC00174\ATRNL1	0,232773524	0,010848166
LINC00174	285908	AP2B1	LINC00174\AP2B1	0,232709016	0,011017822
LINC00174	285908	RAB23	LINC00174\RAB23	0,228806438	0,012468329
LINC00174	285908	WNK2	LINC00174\WNK2	0,223600627	0,014662316
LINC00174	285908	TM7SF3	LINC00174\TM7SF3	0,223451075	0,014730034
LINC00174	285908	KIAA1549	LINC00174\KIAA1549	0,220851731	0,0159514

LINC00174	285908	ALDH7A1	LINC00174\ALDH7A1	0,21526848	0,018876202
LINC00174	285908	SLC44A3	LINC00174\SLC44A3	0,214079191	0,019555993
LINC00174	285908	CLINT1	LINC00174\CLINT1	0,212590799	0,020436495
LINC00174	285908	EXOC7	LINC00174\EXOC7	0,209770688	0,022198978
LINC00174	285908	KDM4B	LINC00174\KDM4B	0,208275174	0,023185642
LINC00174	285908	EPN3	LINC00174\EPN3	0,208225324	0,02321917
LINC00174	285908	GTF3C4	LINC00174\GTF3C4	0,201467027	0,028167756
LINC00174	285908	POLR1A	LINC00174\POLR1A	0,199594075	0,029688944
LINC00174	285908	SLC46A1	LINC00174\SLC46A1	0,193398376	0,035228803
LINC00174	285908	S100A14	LINC00174\S100A14	0,189609742	0,039028956
LINC00174	285908	FBXO2	LINC00174\FBXO2	0,1889332	0,039742595
LINC00174	285908	ATHL1	LINC00174\ATHL1	0,188021649	0,04072144
LINC00174	285908	EPS15L1	LINC00174\EPS15L1	0,18778664	0,040977053
LINC00174	285908	DHTKD1	LINC00174\DHTKD1	0,180650904	0,049403637
