# Supplementary material for: LINC00174 is a novel prognostic factor in thymic epithelial tumors involved in cell migration and lipid metabolism
Source: Cell Death Dis. 2020 Nov 7;11(11):959. doi: 10.1038/s41419-020-03171-9 (PMC7648846; doi:10.1038/s41419-020-03171-9)
Supplement: Supplementary file 18 — Supplementary Table 3_Sheet 4 [file 41419_2020_3171_MOESM18_ESM.pdf]

Prognostic value of LINC00174/mRNAs pairs by multivatiate analysis

LNC	HR	p	logrank(zsc0)	GENE	HR	p	ogrank(zsc0	HR(gene+lnc)	p	logrank(zsc0)	HRmultiv(gene+lnc)	p
LINC00174	1.8876[1.0089-3.5313]	0,046828	0,018416651	UBAC1	2.1639[1.1297-4.1447]	0,01992	0,00556	2.8367[1.3702-5.8726]	0,004979	0,005857774	2.8192[1.2982-6.1222]	0,0088
LINC00174	1.8876[1.0089-3.5313]	0,046828	0,018416651	CCDC30	2.4756[1.1626-5.2716]	0,018744	0,204203	2.621[1.2116-5.6698]	0,01438	0,047089404	2.6233[1.1719-5.8723]	0,01898
LINC00174	1.8876[1.0089-3.5313]	0,046828	0,018416651	SYBU	1.984[0.84991-4.6313]	0,113192	0,051599	2.2552[1.0192-4.9903]	0,044761	0,031745698	3.2164[1.1776-8.7851]	0,02268
LINC00174	1.8876[1.0089-3.5313]	0,046828	0,018416651	FEM1B	0.90729[0.46441-1.7725]	0,775844	0,843092	2.0175[1.0143-4.06]	0,047918	0,035263086	2.068[1.0765-3.9676]	0,02905
LINC00174	1.8876[1.0089-3.5313]	0,046828	0,018416651	SCD5	1.5726[0.85418-2.8953]	0,14599	0,185694	1.9972[1.0428-3.8252]	0,036948	0,049106	2.0448[1.0344-4.0422]	0,03966
LINC00174	1.8876[1.0089-3.5313]	0,046828	0,018416651	CDS1	2.0131[0.98783-4.1026]	0,054069	0,368912	2.1556[1.0738-4.3272]	0,030746	0,339183145	2.0644[0.99751-4.2723]	0,05179
LINC00174	1.8876[1.0089-3.5313]	0,046828	0,018416651	SGPP2	2.1729[1.1139-4.2387]	0,022821	0,083884	2.6481[1.2545-5.5896]	0,01062	0,128896019	2.5267[0.983-5.071]	0,05257
LINC00174	1.8876[1.0089-3.5313]	0,046828	0,018416651	HOMER2	1.4839[0.63004-3.4952]	0,366502	0,054446	2.0829[0.94288-4.6013]	0,069592	0,021175072	2.2121[0.98547-4.9655]	0,05429
LINC00174	1.8876[1.0089-3.5313]	0,046828	0,018416651	CETN3	1.5692[0.64847-3.7974]	0,317627	0,866992	1.9862[1.0279-3.8379]	0,041156	0,925229555	1.9899[0.98677-4.013]	0,05451
LINC00174	1.8876[1.0089-3.5313]	0,046828	0,018416651	ATRNL1	1.1827[0.56062-2.4949]	0,659576	0,510332	1.5819[0.68609-3.6475]	0,281894	0,352376558	2.1953[0.96207-5.0093]	0,06175
LINC00174	1.8876[1.0089-3.5313]	0,046828	0,018416651	NUDT12	2.0659[0.96765-4.4105]	0,06079	0,012903	2.0737[1.0816-3.976]	0,028081	0,02424628	1.959[0.96568-3.9742]	0,06243
LINC00174	1.8876[1.0089-3.5313]	0,046828	0,018416651	ANK3	1.9391[1.0317-3.6446]	0,039682	0,038688	2.0678[1.0775-3.9686]	0,028941	0,025598171	1.9644[0.94957-4.0638]	0,06869
LINC00174	1.8876[1.0089-3.5313]	0,046828	0,018416651	KDM4D	1.6992[0.77559-3.7228]	0,185201	0,490837	2.0951[1.0276-4.2713]	0,04185	0,333442019	1.977[0.93745-4.1694]	0,0734
LINC00174	1.8876[1.0089-3.5313]	0,046828	0,018416651	GRIP1	1.9096[1.0332-3.5296]	0,039007	0,014456	1.9045[1.0724-3.3822]	0,027899	0,013325126	1.8331[0.94325-3.5624]	0,07383
LINC00174	1.8876[1.0089-3.5313]	0,046828	0,018416651	ALDH7A1	1.5189[0.69154-3.3362]	0,297773	0,839644	2.1079[1.0075-4.4101]	0,047711	0,249043086	2.0022[0.93293-4.297]	0,07478
LINC00174	1.8876[1.0089-3.5313]	0,046828	0,018416651	SPIRE2	1.6163[0.82037-3.1844]	0,165223	0,273858	1.8761[0.94887-3.7093]	0,070437	0,373012997	1.9906[0.92259-4.2949]	0,07932
LINC00174	1.8876[1.0089-3.5313]	0,046828	0,018416651	GPHN	1.4926[0.75035-2.9692]	0,253668	0,135062	1.8481[0.97568-3.5005]	0,059506	0,187441582	1.877[0.92529-3.8077]	0,08101
LINC00174	1.8876[1.0089-3.5313]	0,046828	0,018416651	GRHL2	3.3804[0.91633-12.4703]	0,06743	0,112575	3.1852[1.0473-9.6876]	0,041209	0,108466505	2.8936[0.87675-9.5503]	0,08114
LINC00174	1.8876[1.0089-3.5313]	0,046828	0,018416651	ATP6AP2	1.5885[0.8002-3.1533]	0,18588	0,114258	1.9677[1.0245-3.7793]	0,042083	0,176409639	1.8761[0.92275-3.8144]	0,08222
LINC00174	1.8876[1.0089-3.5313]	0,046828	0,018416651	ANKFY1	1.7201[0.86013-3.4398]	0,125057	0,404434	1.9541[1.0217-3.7377]	0,042892	0,112047196	1.8401[0.92433-3.6633]	0,08256
LINC00174	1.8876[1.0089-3.5313]	0,046828	0,018416651	SHROOM2	1.9036[0.9493-3.8171]	0,069768	0,546863	2.0751[1.0605-4.0603]	0,033056	0,197747751	1.9552[0.91508-4.1775]	0,08347
LINC00174	1.8876[1.0089-3.5313]	0,046828	0,018416651	SLC44A3	1.3507[0.72081-2.5311]	0,34811	0,062055	1.6629[0.86782-3.1864]	0,125342	0,0982179	2.0028[0.90393-4.4377]	0,08705
LINC00174	1.8876[1.0089-3.5313]	0,046828	0,018416651	HIST2H2BF	1.5383[0.85132-2.7797]	0,153653	0,258303	1.7524[0.96795-3.1727]	0,063963	0,276825403	1.7212[0.91351-3.243]	0,09294
LINC00174	1.8876[1.0089-3.5313]	0,046828	0,018416651	RPS6KA6	1.9233[0.92927-3.9807]	0,078013	0,113705	2.1047[1.0067-4.4003]	0,047944	0,155330136	1.9332[0.8938-4.1813]	0,09398
LINC00174	1.8876[1.0089-3.5313]	0,046828	0,018416651	FAM199X	1.8374[0.82944-4.0701]	0,133837	0,000946	2.0183[1.0033-4.06]	0,048918	0,034133263	1.8914[0.89016-4.0187]	0,09744
LINC00174	1.8876[1.0089-3.5313]	0,046828	0,018416651	MCOLN3	1.2932[0.52455-3.1881]	0,576517	0,059884	1.7092[0.76529-3.8172]	0,191042	0,05294092	2.1062[0.87262-5.0834]	0,09754
LINC00174	1.8876[1.0089-3.5313]	0,046828	0,018416651	SNX1	1.3499[0.56243-3.2397]	0,501827	0,031791	1.9571[0.95899-3.9939]	0,065046	0,029953162	1.853[0.88538-3.8782]	0,10164
LINC00174	1.8876[1.0089-3.5313]	0,046828	0,018416651	PANK1	1.4114[0.77335-2.5757]	0,261614	0,637255	1.7138[0.96773-3.0351]	0,064675	0,181835426	1.6652[0.90215-3.0738]	0,10295
LINC00174	1.8876[1.0089-3.5313]	0,046828	0,018416651	FSTL4	1.6162[0.78188-3.3407]	0,195033	0,781448	1.791[0.92868-3.4539]	0,082005	0,678281437	1.7699[0.89076-3.5168]	0,10315
LINC00174	1.8876[1.0089-3.5313]	0,046828	0,018416651	UBFD1	1.6576[0.79064-3.4753]	0,180882	0,150102	1.9802[1.0212-3.8397]	0,04317	0,010675252	1.8417[0.87706-3.8674]	0,10665
LINC00174	1.8876[1.0089-3.5313]	0,046828	0,018416651	SORBS2	1.5873[0.76078-3.3118]	0,218196	0,384213	1.8055[0.91987-3.5438]	0,085932	0,260959785	1.7804[0.88001-3.602]	0,10861
LINC00174	1.8876[1.0089-3.5313]	0,046828	0,018416651	STX6	1.2072[0.65025-2.2411]	0,550843	0,211725	1.6854[0.92876-3.0583]	0,085999	0,016418311	1.7147[0.88079-3.3383]	0,11262
LINC00174	1.8876[1.0089-3.5313]	0,046828	0,018416651	PABPC4L	1.7829[0.72954-4.3573]	0,204682	0,313678	2.0375[0.89697-4.6284]	0,089085	0,093680213	1.9376[0.8415-4.4616]	0,12007
LINC00174	1.8876[1.0089-3.5313]	0,046828	0,018416651	CLDN12	1.5923[0.77989-3.2509]	0,201485	0,337164	1.9319[0.96175-3.8805]	0,064255	0,127466333	1.7875[0.85616-3.7321]	0,12198
LINC00174	1.8876[1.0089-3.5313]	0,046828	0,018416651	USP40	1.5285[0.73975-3.1583]	0,251841	0,301128	1.8636[0.95287-3.6448]	0,068921	0,027523916	1.7424[0.85323-3.5583]	0,12744
LINC00174	1.8876[1.0089-3.5313]	0,046828	0,018416651	RNF170	1.587[0.77213-3.262]	0,208938	0,125052	1.9052[0.96571-3.7588]	0,062976	0,408010719	1.7553[0.83693-3.6813]	0,13651
LINC00174	1.8876[1.0089-3.5313]	0,046828	0,018416651	DNAI1	1.5262[0.89491-2.6027]	0,120584	0,056288	1.6769[0.98972-2.8412]	0,054656	0,069396661	1.5404[0.86781-2.7343]	0,14001
LINC00174	1.8876[1.0089-3.5313]	0,046828	0,018416651	TP63	1.655[0.76776-3.5677]	0,198576	0,957911	1.8572[0.91672-3.7627]	0,085686	0,498546746	1.7237[0.82215-3.6138]	0,14944
LINC00174	1.8876[1.0089-3.5313]	0,046828	0,018416651	CNTNAP3	1.1028[0.55254-2.2009]	0,781431	0,036995	1.4033[0.71192-2.7663]	0,327747	0,024763568	1.7352[0.80556-3.7376]	0,15921
LINC00174	1.8876[1.0089-3.5313]	0,046828	0,018416651	TMEM180	1.4553[0.78269-2.706]	0,235728	0,753432	1.8443[0.96779-3.5146]	0,062817	0,062430361	1.6966[0.81001-3.5534]	0,1611
LINC00174	1.8876[1.0089-3.5313]	0,046828	0,018416651	PKP4	1.1652[0.60286-2.2522]	0,64922	0,224098	1.7142[0.87171-3.371]	0,118274	0,3464401	1.6569[0.81764-3.3578]	0,16112
LINC00174	1.8876[1.0089-3.5313]	0,046828	0,018416651	RAB14	1.3331[0.65869-2.6979]	0,424138	0,18919	1.7778[0.95625-3.3053]	0,068972	0,135319939	1.6368[0.81919-3.2706]	0,16293
LINC00174	1.8876[1.0089-3.5313]	0,046828	0,018416651	COG5	1.3509[0.69059-2.6424]	0,379644	0,381228	1.7229[0.93095-3.1887]	0,083241	0,255168347	1.6037[0.82157-3.1303]	0,16634
LINC00174	1.8876[1.0089-3.5313]	0,046828	0,018416651	TRPM7	1.4696[0.6842-3.1565]	0,323633	0,482989	1.8668[0.91117-3.8248]	0,088047	0,126004683	1.7032[0.79763-3.6371]	0,16888
LINC00174	1.8876[1.0089-3.5313]	0,046828	0,018416651	LMBR1	1.3202[0.64066-2.7205]	0,451443	0,291103	1.7315[0.90942-3.2968]	0,094719	0,455245516	1.6177[0.81307-3.2187]	0,17055
LINC00174	1.8876[1.0089-3.5313]	0,046828	0,018416651	KIAA1549	1.1831[0.6276-2.2305]	0,603139	0,056692	1.4879[0.79102-2.7986]	0,217681	0,035268253	1.5998[0.81553-3.1382]	0,17168
LINC00174	1.8876[1.0089-3.5313]	0,046828	0,018416651	MPP5	1.0149[0.52646-1.9567]	0,964681	0,375025	1.474[0.80216-2.7085]	0,211355	0,479652626	1.5736[0.81189-3.0498]	0,17934
LINC00174	1.8876[1.0089-3.5313]	0,046828	0,018416651	TM7SF3	1.2352[0.62102-2.4567]	0,547137	0,903303	1.799[0.92699-3.4914]	0,082582	0,146782993	1.6374[0.79526-3.3714]	0,1808
LINC00174	1.8876[1.0089-3.5313]	0,046828	0,018416651	CDH1	1.4269[0.71682-2.8404]	0,311475	0,911191	1.7885[0.87174-3.6695]	0,112825	0,372116575	1.6554[0.78433-3.4941]	0,18597
LINC00174	1.8876[1.0089-3.5313]	0,046828	0,018416651	EXOC7	0.94215[0.45166-1.9653]	0,873774	0,750138	1.7972[0.92438-3.4941]	0,083952	0,431597454	1.6311[0.78665-3.3821]	0,18851
LINC00174	1.8876[1.0089-3.5313]	0,046828	0,018416651	MAP3K2	1.4113[0.70814-2.8125]	0,327537	0,473249	1.6841[0.8941-3.1722]	0,106632	0,331463182	1.5811[0.79854-3.1304]	0,18869
LINC00174	1.8876[1.0089-3.5313]	0,046828	0,018416651	XYLT2	1.0847[0.57765-2.0369]	0,800271	0,099534	1.6863[0.86864-3.2735]	0,122613	0,238272813	1.5724[0.78947-3.1318]	0,1979
LINC00174	1.8876[1.0089-3.5313]	0,046828	0,018416651	BRD1	0.88751[0.45284-1.7394]	0,728142	0,240955	1.4976[0.71718-3.1274]	0,282333	0,931287812	1.6784[0.74377-3.7873]	0,21237
LINC00174	1.8876[1.0089-3.5313]	0,046828	0,018416651	PSD3	1.6145[0.83787-3.1108]	0,152319	0,181556	1.7476[0.94756-3.2232]	0,073854	0,167754587	1.5847[0.7663-3.277]	0,21426
LINC00174	1.8876[1.0089-3.5313]	0,046828	0,018416651	RAD50	1.3647[0.68064-2.7364]	0,380989	0,99662	1.7088[0.91233-3.2005]	0,094243	0,120828683	1.5533[0.76816-3.1409]	0,22027
LINC00174	1.8876[1.0089-3.5313]	0,046828	0,018416651	CLTC	1.1946[0.61518-2.3197]	0,599501	0,306588	1.6877[0.91812-3.1023]	0,091994	0,324921211	1.524[0.76437-3.0385]	0,2314
LINC00174	1.8876[1.0089-3.5313]	0,046828	0,018416651	GPR107	1.2835[0.65689-2.5078]	0,465191	0,177156	1.7233[0.91612-3.2418]	0,091366	0,072421876	1.5534[0.74936-3.2203]	0,23632
LINC00174	1.8876[1.0089-3.5313]	0,046828	0,018416651	CAMSAP1	1.2122[0.6634-2.2149]	0,531557	0,857104	1.5717[0.87683-2.8172]	0,128878	0,014771681	1.4488[0.76555-2.7418]	0,25465
LINC00174	1.8876[1.0089-3.5313]	0,046828	0,018416651	CLINT1	1.1065[0.51063-2.3976]	0,797592	0,849858	1.6881[0.8386-3.398]	0,142414	0,376117405	1.5425[0.72997-3.2595]	0,2562
LINC00174	1.8876[1.0089-3.5313]	0,046828	0,018416651	SGPL1	1.2738[0.68906-2.3547]	0,440122	0,024469	1.6035[0.88326-2.9111]	0,120661	0,02270741	1.4489[0.75613-2.7763]	0,26378
LINC00174	1.8876[1.0089-3.5313]	0,046828	0,018416651	MARVELD2	1.3239[0.68233-2.5686]	0,406745	0,776494	1.6016[0.84065-3.0512]	0,152092	0,431506382	1.4669[0.74776-2.8775]	0,26508
LINC00174	1.8876[1.0089-3.5313]	0,046828	0,018416651	ZBTB41	1.2644[0.60877-2.6259]	0,529325	0,825331	1.6129[0.86546-3.006]	0,132301	0,169039084	1.4711[0.74293-2.9131]	0,26808
LINC00174	1.8876[1.0089-3.5313]	0,046828	0,018416651	FKTN	1.2661[0.61075-2.6247]	0,52585	0,37314	1.6048[0.85462-3.0135]	0,141203	0,283687727	1.4631[0.73748-2.9027]	0,27625
LINC00174	1.8876[1.0089-3.5313]	0,046828	0,018416651	MYO10	1.346[0.67037-2.7024]	0,403457	0,308711	1.6409[0.84116-3.201]	0,146326	0,176798994	1.4794[0.72892-3.0027]	0,27816
LINC00174	1.8876[1.0089-3.5313]	0,046828	0,018416651	UPF1	0.79454[0.38004-1.6612]	0,541049	0,637989	1.6007[0.84159-3.0445]	0,151511	0,979540445	1.4594[0.73534-2.8965]	0,2797
LINC00174	1.8876[1.0089-3.5313]	0,046828	0,018416651	MAML3	1.3171[0.67105-2.5851]	0,423398	0,651118	1.6092[0.87105-2.9728]	0,128728	0,2912809	1.4312[0.73126-2.801]	0,29536
LINC00174	1.8876[1.0089-3.5313]	0,046828	0,018416651	EPN3	1.2311[0.5463-2.7745]	0,615945	0,415496	1.6268[0.74369-3.5587]	0,223037	0,217066647	1.4774[0.69268-3.1512]	0,31255
LINC00174	1.8876[1.0089-3.5313]	0,046828	0,018416651	ZNF449	1.0329[0.52019-2.0508]	0,926365	0,179648	1.5084[0.79307-2.8689]	0,210148	0,219796192	1.407[0.7238-2.7352]	0,31399
LINC00174	1.8876[1.0089-3.5313]	0,046828	0,018416651	PAX9	1.1327[0.54504-2.3539]	0,738528	0,052857	1.5034[0.74129-3.0491]	0,258393	0,035637567	1.4147[0.71176-2.8118]	0,32225
LINC00174	1.8876[1.0089-3.5313]	0,046828	0,018416651	KDM4B	0.72664[0.3652-1.4458]	0,362968	0,360456	1.3905[0.67788-2.8524]	0,368451	0,298341771	1.4595[0.68865-3.0931]	0,32384
LINC00174	1.8876[1.0089-3.5313]	0,046828	0,018416651	PDPK1	1.2182[0.58083-2.555]	0,601441	0,816415	1.6748[0.79777-3.5161]	0,172916	0,185038319	1.4793[0.67636-3.2355]	0,32675
LINC00174	1.8876[1.0089-3.5313]	0,046828	0,018416651	CCDC8	0.92236[0.47168-1.8037]	0,813267	0,867481	1.4507[0.7052-2.9842]	0,312049	0,331373868	1.4801[0.66891-3.275]	0,33321
LINC00174	1.8876[1.0089-3.5313]	0,046828	0,018416651	ZNF280B	1.1851[0.49152-2.8574]	0,705281	0,671234	1.6881[0.78164-3.646]	0,182564	0,583939057	1.4586[0.66478-3.2004]	0,34641
LINC00174	1.8876[1.0089-3.5313]	0,046828	0,018416651	C1orf109	1.1486[0.46801-2.8187]	0,762352	0,817266	1.7665[0.7676-4.0651]	0,180886	0,265164031	1.5092[0.62551-3.6412]	0,35973
LINC00174	1.8876[1.0089-3.5313]	0,046828	0,018416651	PGAP1	1.3191[0.67891-2.5631]	0,413771	0,646277	1.6105[0.85283-3.0413]	0,141776	0,707778269	1.4177[0.66705-3.0133]	0,36418
LINC00174	1.8876[1.0089-3.5313]	0,046828	0,018416651	UNC5B	1.3047[0.71754-2.3722]	0,383297	0,815558	1.5362[0.87681-2.6913]	0,133492	0,60819766	1.345[0.68111-2.6561]	0,39321
LINC00174	1.8876[1.0089-3.5313]	0,046828	0,018416651	GLI2	1.0086[0.50219-2.0258]	0,98074	0,040163	1.3118[0.66698-2.5801]	0,431609	0,024437373	1.2946[0.67823-2.4709]	0,43376
LINC00174	1.8876[1.0089-3.5313]	0,046828	0,018416651	MTSS1L	0.6906[0.38088-1.2521]	0,222705	0,897792	1.1814[0.6031-2.3144]	0,626948	0,216493701	1.3078[0.64442-2.6542]	0,45737
LINC00174	1.8876[1.0089-3.5313]	0,046828	0,018416651	GTF2I	1.1454[0.5722-2.293]	0,701365	0,48068	1.4714[0.70668-3.0636]	0,301996	0,375975141	1.2856[0.6109-2.7056]	0,50807
LINC00174	1.8876[1.0089-3.5313]	0,046828	0,018416651	MAST4	1.125[0.57841-2.1881]	0,728602	0,711683	1.4134[0.73842-2.7053]	0,29624	0,828448268	1.2434[0.62056-2.4914]	0,53895
LINC00174	1.8876[1.0089-3.5313]	0,046828	0,018416651	LRIG3	1.1674[0.59738-2.2815]	0,650623	0,792924	1.446[0.75562-2.767]	0,265396	0,744673042	1.2509[0.61036-2.5637]	0,54087
LINC00174	1.8876[1.0089-3.5313]	0,046828	0,018416651	ZNF132	0.74781[0.38251-1.462]	0,395515	0,742212	1.2997[0.62688-2.6948]	0,481	0,446852999	1.2505[0.61001-2.5635]	0,54161
LINC00174	1.8876[1.0089-3.5313]	0,046828	0,018416651	SMO	0.65375[0.32836-1.3016]	0,226375	0,674968	1.1586[0.53494-2.5092]	0,708922	0,616748833	1.2234[0.54945-2.7241]	0,62149
LINC00174	1.8876[1.0089-3.5313]	0,046828	0,018416651	KLHDC10	0.99995[0.5153-1.9404]	0,999889	0,869921	1.3301[0.71067-2.4894]	0,37241	0,297060764	1.1488[0.58598-2.252]	0,68636
LINC00174	1.8876[1.0089-3.5313]	0,046828	0,018416651	RAB23	0.88987[0.45802-1.7289]	0,730599	0,528184	1.3477[0.73158-2.4828]	0,338384	0,900806452	1.1422[0.57875-2.2543]	0,70143
LINC00174	1.8876[1.0089-3.5313]	0,046828	0,018416651	PAX1	0.8109[0.56039-1.1734]	0,266215	0,910322	0.89956[0.5207-1.5541]	0,704333	0,259267757	1.108[0.62153-1.9754]	0,728
LINC00174	1.8876[1.0089-3.5313]	0,046828	0,018416651	SLC30A1	0.89139[0.4609-1.724]	0,732617	0,258919	1.2976[0.68045-2.4745]	0,428946	0,979559101	1.1313[0.54701-2.3397]	0,73933
LINC00174	1.8876[1.0089-3.5313]	0,046828	0,018416651	NRCAM	0.72519[0.40398-1.3018]	0,281718	0,198645	0.95895[0.48472-1.8971]	0,904153	0,12654266	1.0613[0.54406-2.0704]	0,86141
LINC00174	1.8876[1.0089-3.5313]	0,046828	0,018416651	SORCS2	0.6968[0.36595-1.3268]	0,271561	0,524021	0.94761[0.45538-1.9719]	0,885558	0,939469177	0.98378[0.50379-1.9211]	0,96181
LINC00174	1.8876[1.0089-3.5313]	0,046828	0,018416651	GTF3C4	0.78869[0.40242-1.5457]	0,48927	0,408837	1.1926[0.64654-2.1997]	0,572905	0,880099215	0.99865[0.50572-1.972]	0,99689
