# Supplementary material for: LINC00174 is a novel prognostic factor in thymic epithelial tumors involved in cell migration and lipid metabolism
Source: Cell Death Dis. 2020 Nov 7;11(11):959. doi: 10.1038/s41419-020-03171-9 (PMC7648846; doi:10.1038/s41419-020-03171-9)
Supplement: Supplementary file 19 — Supplementary Table 3_Sheet 5 [file 41419_2020_3171_MOESM19_ESM.pdf]

List of the five genes positively correlated to LINC00174 in IRE cohort and TCGA cohort selected for their prognostic value in combination with LINC00174 by multivariate analysis (see sheet 4)

**TCGA thymoma cohort**			**IRE Thymoma cohort**		
**mRNA/LINC00174 pairs**	**R Spearman**	**p**	**mRNA/LINC00174 pairs**	**R Spearman**	**p**
UBAC1\LINC00174	0,240471443	0,008567	UBAC1\LINC00174	0,866666615	0,004508
CCDC30\LINC00174	0,606387979	0	CCDC30\LINC00174	0,74999994	0,025491
SYBU\LINC00174	0,259606894	0,004462	SYBU\LINC00174	0,966666639	0,000165
FEM1B\LINC00174	0,308688221	0,000673	FEM1B\LINC00174	0,766666651	0,02139
SCD5\LINC00174	0,319605469	0,000422	SCD5\LINC00174	0,699999928	0,043254
