# Supplementary material for: LINC00174 is a novel prognostic factor in thymic epithelial tumors involved in cell migration and lipid metabolism
Source: Cell Death Dis. 2020 Nov 7;11(11):959. doi: 10.1038/s41419-020-03171-9 (PMC7648846; doi:10.1038/s41419-020-03171-9)
Supplement: Supplementary file 20 — Supplementary Table 3_Sheet 6 [file 41419_2020_3171_MOESM20_ESM.pdf]

List of compounds able to downregulate LINC00174-associated 128-gene signature.

Compound	Description	Score
catechin	beta secretase inhibitor, fatty acid synthase inhibitor, free radical scavenger,	-99.08
CO-101244	ionotropic glutamate receptor antagonist	-98.46
mitomycin-c	DNA alkylating drug, DNA inhibitor, DNA synthesis inhibitor	-98.43
YC-1	activator of soluble guanylyl cyclase, guanylate cyclase activator, hypoxia in	-95.58
IRL-2500	endothelin receptor antagonist	-94.67
retinol	RAR receptor binder	-94.65
olanzapine	dopamine receptor antagonist, serotonin receptor antagonist	-93.09
cinacalcet	calcimimetic that activates calcium-sensing receptors	-92.30
testosterone	androgen receptor (AR) agonist, androgen receptor agonist, testosterone rec	-92.23
CAY-10415	insulin sensitizer	-91.97
amisulpride	dopamine receptor antagonist	-91.64
loreclezole	GABA receptor agonist	-91.57
lenalidomide	angiogenesis inhibitor, cereblon inhibitor, tumor apoptosis inducer, tumor	-91.42
1,2-dichlorobenzene	hepatotoxicant that induces oxidative stress and inflammatory response	-91.40
psoromic-acid	Rab-Prenylation Inhibitor	-90.91
importazole	importin-beta transport receptor inhibitor	-90.60
alfaxalone	11-beta hydroxysteroid dehydrogenase inhibitor, chloride channel agonist,	-90.58
damnacanthal	src inhibitor	-90.47
immethridine	histamine receptor agonist	-90.43
felbamate	carbonic anhydrase inhibitor, GABA receptor modulator, glutamate recepto	-90.16
